# Synthesis, structure and redox properties of single-atom bridged diuranium complexes supported by aryloxides†

**DOI:** 10.1039/d4dt01819b

**Published:** 2024-07-23

**Authors:** Fang-Che Hsueh, Luciano Barluzzi, Thayalan Rajeshkumar, Rosario Scopelliti, Ivica Zivkovic, Laurent Maron, Marinella Mazzanti

**Affiliations:** a Group of Coordination Chemistry, Institut des Sciences et Ingénierie Chimiques, École Polytechnique Fédérale de Lausanne (EPFL) 1015 Lausanne Switzerland marinella.mazzanti@epfl.ch; b Laboratoire de Physique et Chimie des Nano-objets, Institut National des Sciences Appliquées 31077 Toulouse Cedex 4 France; c Institut des Sciences et Ingénierie Chimiques, École Polytechnique Fédérale de Lausanne (EPFL) 1015 Lausanne Switzerland; d Laboratory for Quantum Magnetism, Institute of Physics, École Polytechnique Fédérale de Lausanne (EPFL) CH-1015 Lausanne Switzerland

## Abstract

Single-atom (group 15 and group 16 anions) bridged dimetallic complexes of low oxidation state uranium provide a convenient route to implement multielectron transfer and promote magnetic communication in uranium chemistry, but remain extremely rare. Here we report the synthesis, redox and magnetic properties of N^3−^, O^2−^, and S^2−^ bridged diuranium complexes supported by bulky aryloxide ligands. The U(iv)/U(iv) nitride [Cs(THF)_8_][(U(OAr)_3_)_2_(μ-N)], 1 could be prepared and characterized but could not be reduced. Reduction of the neutral U(iv)/U(iv) complexes [(U(OAr)_3_)_2_(μ-X)] A (X = O) and B (X = S) led to the isolation and characterization of the U(iv)/U(iii) and U(iii)/U(iii) analogues. Complexes [(K(THF)_4_)_2_(U(OAr)_2_)_2_(μ-S)_2_], 5 and [K(2.2.2-cryptand)]_2_[(U(OAr)_3_)_2_(μ-S)], 6 are the first examples of U(iii) sulphide bridged complexes. Computational studies and redox properties allow the reactivity of the dimetallic complexes to be related to their electronic structure.

## Abstract 2

Reduction of the neutral U^IV^–X–U^IV^ (X = O or S) complexes supported by aryloxide ligands yielded the U^IV^/U^III^ and U^III^/U^III^ analogues. Notably, U^III^–S–U^III^ is the first example of a U(iii) sulphide-bridged complex.

## Graphical abstract

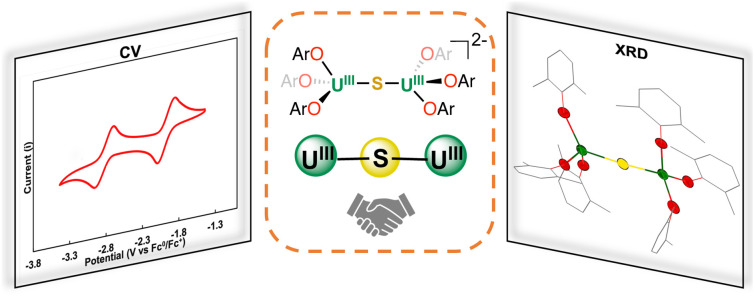

## Introduction

Single-atom (group 15 and group 16 anions) bridged dimetallic complexes of uranium have attracted a large number of studies motivated by the need for a better understanding of uranium–ligand bonding interactions^1–18^ but also because they provide a convenient route for implementing multielectron transfer in uranium chemistry,^16,19–28^ as they show unexpected catalytic activity^29,30^ and promote magnetic communication.^2,23,31–35^ Most of the reported single-atom bridged uranium complexes contain uranium in high oxidation states (+iv to +vi), with complexes of U(iv) being by far the most studied. Until recently, only one single-atom bridged complex containing uranium in the +iii oxidation state, [(U(C_5_H_5_)_2_)_2_(μ-O)], had been crystallographically characterized,^36^ but a synthetic route could not be identified. In contrast, oxide, sulphide, or nitride bridged diuranium(iv) complexes were conveniently synthesized by reacting a U(iii) precursor with stoichiometric amounts of carefully chosen oxidizing atom transfer reagents.^7,13,23^ Alternative routes to oxide and sulphide bridged diuranium(iv) complexes include abstraction of sulphide or oxide during the reduction of CO_2_ or CS_2_ by U(iii) complexes.^16,37^

**Fig. 1:**
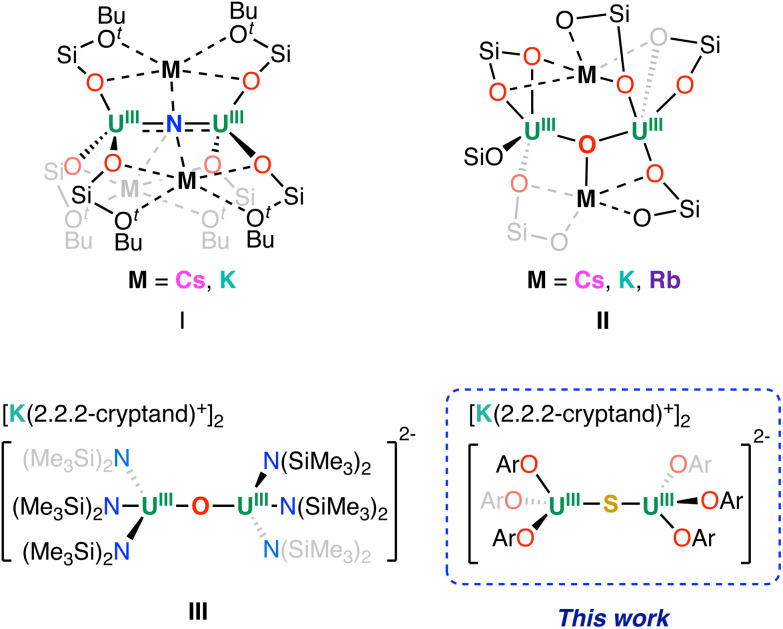
Previously reported U(iii) bridging complexes.

We reported the synthesis of diuranium(iv) oxide and nitride complexes supported by tris(*tert*-butoxy)siloxide ligands, which were prepared by the reaction of the U(iii) complex [U(OSi(O^*t*^Bu)_3_)_3_]_2_ with atom transfer reagents.^12,23^ We showed that these complexes can be reduced with KC_8_, leading to the rational synthesis of nitride- and oxide-bridged diuranium(iii) complexes I and II (Fig. 1).^19,23,38–40^

Attempts to prepare the analogous sulphide bridged diuranium(iii) complex have so far only led to U(iv) decomposition products.^41^ Complexes I and II showed high reactivity towards N_2_ resulting in the first examples of four electron reduction and cleavage of N_2_ effected by a uranium complex in the absence of external reducing agents.^19,23,39,40^ In contrast, the two- and three-electron reduction of the triphenylsiloxide oxide-bridged diuranium(iv) complex [(U(OSiPh_3_)_3_(DME))_2_(μ-O)] yielded formal “U(ii)/U(iv)”, and “U(i)/U(iv)” complexes *via* ligand migration and formation of uranium–arene δ-bond interactions.^27^ Remarkably, these complexes can promote the reduction of substrates restoring the original ligand arrangement. Recently, we also investigated the reduction of diuranium(iv) complexes supported by the –N(SiMe_3_)_2_ ligand and found that, while the nitride-bridged U(iv) complex [NBu_4_][(U(N(SiMe_3_)_2_)_3_)_2_(μ-N)]^32^ could not be reduced further, reduction of the oxide-bridged complex [(U(N(SiMe_3_)_2_)_3_)_2_(μ-O)] allowed the isolation of the diuranium(iii) analogue (complex III in Fig. 1).^21^

Surprisingly this complex did not reduce N_2_, but showed unprecedented reactivity, including four electron reduction of azobenzene by a single metal centre through the delivery of “masked U(ii)”.^20^ Overall, these results highlighted that the attractive and diverse reactivity demonstrated by the single-atom bridged diuranium(iii) complexes drastically depend on the steric and electronic properties of the supporting ligand. Hence, we set out to investigate the possibility of accessing U(iii)–X–U(iii) complexes with X = N, O, S, using the 2,6-di-*tert*-butylphenoxide supporting ligand. Taking advantage of the reported diuranium(iv) complexes [(U(OAr)_3_)_2_(μ-X)], (X = O or S, complex A and B; OAr = 2,6-di-*tert*-butylphenoxide),^7^ we were able to prepare the U(iii)–O–U(iii) and U(iii)–O–U(iv) derivatives and compare their redox properties with those of the amide and siloxide analogues and crystallographically characterize the first example of a diuranium(iii) sulphide-bridged complex.

## Results and discussion

### U(iv)/U(iv) complexes

At first, we synthesised the previously reported chalcogenide bridged diuranium(iv) complexes with aryloxide ligands, [(U(OAr)_3_)_2_(μ-X)] (X = O or S, complex A and B; OAr = 2,6-di-*tert*-butylphenoxide).^7^

**Scheme 1:**
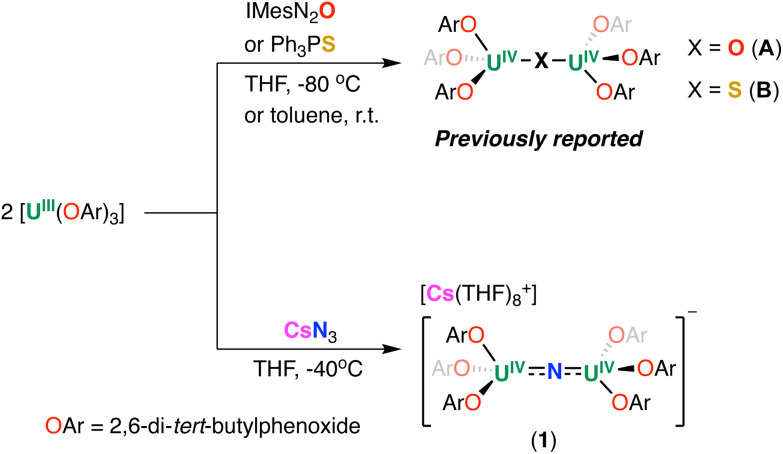
Synthesis of the U(iv)/U(iv) bridging complexes.

The complex [(U(OAr)_3_)_2_(μ-O)] (A) was prepared in 80% yield using a modified literature procedure^7^ by reacting [U(OAr)_3_] in THF at −80 °C with the N_2_O adduct of the N-heterocyclic carbene 1,3-dimesitylimidazol 2-ylidene (IMes), IMesN_2_O^37,42^ (Scheme 1). The sulphide complex [(U(OAr)_3_)_2_(μ-S)] (B) was prepared in 91% by reacting [U(OAr)_3_] with 0.5 equiv. PPh_3_S according to a slightly modified version of the previously reported procedure^7^ (Scheme 1).

To compare the redox reactivity and magnetic properties of compounds presenting different linkers we also pursued the synthesis of the nitride-bridged analogous complex that had not been reported previously. The synthesis of the nitride bridged diuranium(iv) complex [Cs(THF)_8_][(U(OAr)_3_)_2_(μ-N)] (1) (Scheme 1) was achieved by reacting [U(OAr)_3_] with alkali azide, a method that had previously led to the isolation of several nitride-bridged complexes supported by amide or siloxide ligands.^5,12,28,32,40,43,44^

**Fig. 2:**
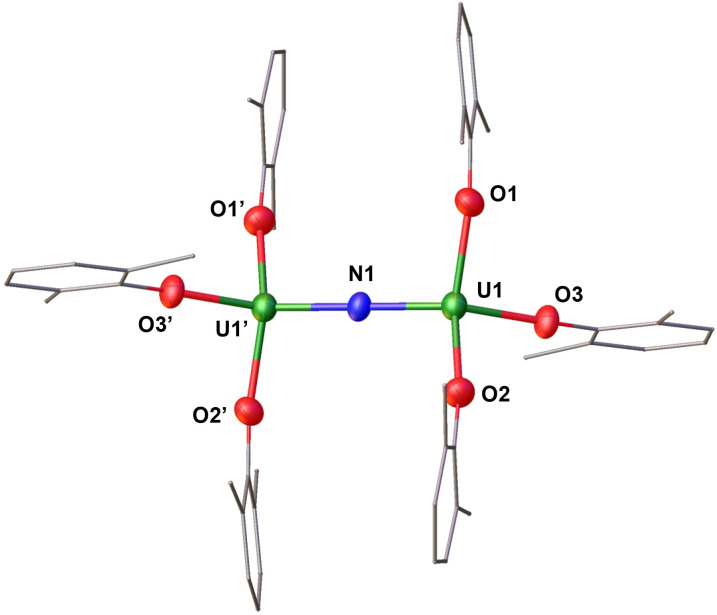
Molecular structure of the anionic species [(U(OAr)_3_)_2_(μ-N)]^−^ in (1) with portions of the aryloxide ligands depicted as wire frames for clarity. Thermal ellipsoids are drawn at the 50% probability level. [Cs(THF)_8_]^+^ cation, hydrogen atoms, and methyl groups on the –OAr ligands have been omitted for clarity.

The addition of 0.5 equiv. of CsN_3_ to 1.0 equiv. of [U(OAr)_3_] in THF at −40 °C for 4 days, resulted in the formation of a dark orange solution. Analysis of the reaction mixture by ^1^H NMR spectroscopy in THF-*d*_8_ showed the full consumption of the U(iii) precursor and appearance of a new species (Fig. S5†). Single crystals suitable for X-ray diffraction studies were obtained in 88% yield from a concentrated THF/*n*-hexane solution at −40 °C and were identified as the dimeric complex, [Cs(THF)_8_][(U(OAr)_3_)_2_(μ-N)] (1). The ^1^H NMR spectrum of isolated complex 1 in THF-*d*_8_ shows broad resonances at *δ* 5.74 and −8.54 ppm at room temperature (Fig. S7†), while at lower temperatures (−40 °C), three resonances at *δ* 96.95, −19.72, and −39.15 ppm are observed, suggesting fluxional behaviour (Fig. S7†). Complex 1 crystallizes in the space group *P*2_1_/*c*, with the full molecule generated by symmetry. The solid-state molecular structure of 1 (Fig. 2) shows an ion pair, consisting of an outer-sphere [Cs(THF)_8_]^+^ cation and the [(U(OAr)_3_)_2_(μ-N)]^−^ anion. Each uranium centre is tetra-coordinated in a distorted tetrahedral geometry. The two uranium(iv) ions are bridged by a nitride (N^3−^) anion and are each bound by three –OAr ligands. The U–O_Ar_ bond distances (2.178(4)–2.226(4) Å) are elongated compared to the U(iii) precursor, [U(OAr)_3_] (2.149(4)–2.165(3) Å).^45^ This elongation could be due to reduced *tert*-butyl interactions between the aryloxides upon the formation of the bridging complex. The O_Ar_–U–O_Ar_ and N–U–O_Ar_ bond angles are in the range of 94.21(17)–138.43(12)°. The values of the U–N–U bond distances (2.0612(5) Å) and angle (180.0°) are consistent with the previously reported anionic U^IV^–N–U^IV^ complexes supported by –OSi(O^*t*^Bu)_3_ and –N(SiMe_3_)_2_ ligands (2.032(12)–2.083(5) Å; 168.4(3)–179(1)°).^32,46^

### U(iii)/U(iii) complexes

With the bridging U(iv)/U(iv) complexes A, B and 1 in hand, we next investigate their redox reactivity.

At first, we explored the reduction of complex 1. Attempts to reduce complex 1 with excess KC_8_ at −80 °C proved unsuccessful and led to a complex mixture of unreacted 1 and unidentified species (Fig. S8†). The reaction of 1 with 1.0 equiv. of KC_8_ in THF-*d*_8_ at −40 °C resulted in a mixture of 1 and a new species, as observed by ^1^H NMR spectroscopy (Fig. S9†). The addition of a second equiv. of KC_8_ to the reaction mixture at −40 °C led to the full consumption of 1 and formation of a new species, as indicated by ^1^H NMR spectroscopy (Fig. S9†). Unfortunately, multiple attempts to isolate single crystals of this species for X-ray diffraction studies proved unsuccessful.

#### Oxide-bridged complexes

^1^H NMR studies showed that the reaction of A with 2.0 equiv. of KC_8_ in THF-*d*_8_ at −80 °C resulted in a mixture of species that were assigned as the U(iii)/U(iv), “[K(THF)_*x*_][(U(OAr)_3_)_2_(μ-O)]” and U(iii)/U(iii), “[K(THF)_*x*_]_2_[(U(OAr)_3_)_2_(μ-O)]” complexes (Fig. S10†). The addition of 3.0 equiv. of KC_8_ (5.0 equiv. in total) to the reaction mixture at −80 °C led to a full consumption of the U(iii)/U(iv) species and to the formation of a U(iii)/U(iii) complex, as suggested by ^1^H NMR spectroscopy (Fig. S10†). However, the putative U(iii)/U(iii), “[K(THF)_*x*_]_2_[(U(OAr)_3_)_2_(μ-O)]” complex is extremely sensitive to temperature in solution. Analysis by variable temperature ^1^H NMR spectroscopy revealed complete decomposition of “[K(THF)_*x*_]_2_[(U(OAr)_3_)_2_(μ-O)]” within 10 min at −40 °C, resulting in an intractable mixture (Fig. S11†).

**Fig. 3:**
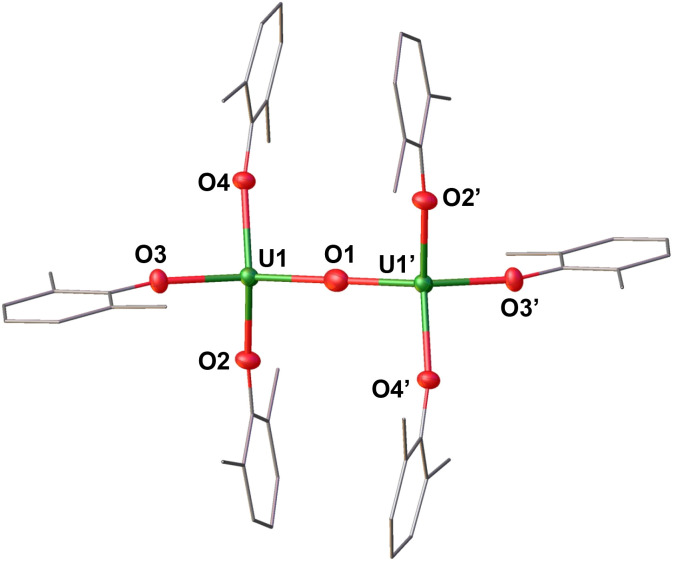
Molecular structure of the anionic species of [(U(OAr)_3_)_2_(μ-O)]^−^ (2) with portions of the aryloxide ligands depicted as wire frames for clarity. Thermal ellipsoids are drawn at the 50% probability level. [Li(THF)_4_]^+^ cation, hydrogen atoms, solvent molecules, and methyl groups on the –OAr ligands have been omitted for clarity.

When the reduction of A was performed with 5.0 equiv. of KC_8_ in presence of 5.0 equiv. of LiI, a putative U(iii)/U(iii) was also formed that decomposes over time at −40 °C (Fig. S13†). The product formed from the decomposition of the U(iii)/U(iii) complex at −40 °C could be crystallographically characterized as the mix-valent diuranium(iii)/(iv) complex, [Li(THF)_4_][U(OAr)_3_(μ-O)] (2) (Fig. 3). The ^1^H NMR spectrum of complex 2 is practically the same as that observed for the putative [K(THF)_*x*_][(U(OAr)_3_)_2_(μ-O)] analogue suggesting that cations are outer-sphere.

Complex 2 crystallizes in the space group *P*1̄, with the full molecule generated by symmetry. The solid-state molecular structure of complex 2 (Fig. 3) displays an anionic dinuclear complex, [(U(OAr)_3_)_2_(μ-O)]^−^ where the two uranium ions are bridged by an oxide (O^2−^), and each uranium is tetra-coordinated by three –OAr ligands and one bridging oxide in a distorted tetrahedral environment. The structure is completed by one outer-sphere [Li(THF)_4_]^+^ cation. The U–O_Ar_ distances (2.208(3)–2.246(3) Å) are longer compared to the U–O_Ar_ lengths in the U(iii) precursor, [U(OAr)_3_] (2.149(4)–2.165(3) Å) and U(iv) complex, [U(OAr)_4_] (2.135(4) Å).^47^ This elongation is attributed to the release of steric repulsion upon the formation of the bridging complex. The O_Ar_–U–O_Ar_ and O_oxide_–U–O_Ar_ bond angles (93.16(12)–137.63(12)°) are comparable to those found in 1. The U–O_oxide_ distances (2.15427(12) Å) are in the range of the previously reported U(iii)/U(iv) complexes, [K(2.2.2-cryptand)][(U(N(SiMe_3_)_2_)_3_)_2_(μ-O)] (2.067(6)–2.273(6) Å)^21^ and [K(DME)_4_][(K(DME)U(calix[4]tetrapyrrole))_2_(μ-O)] (2.017(10)–2.226(10) Å).^48^

The U–O–U angle (180.0°) is slightly more linear compared to those found in the U(iii)/U(iv) analogues supported by –N(SiMe_3_)_2_ ^21^ and calix[4]tetrapyrrole^48^ ligands (177.8(4)–179.0(6)°).

**Scheme 2:**
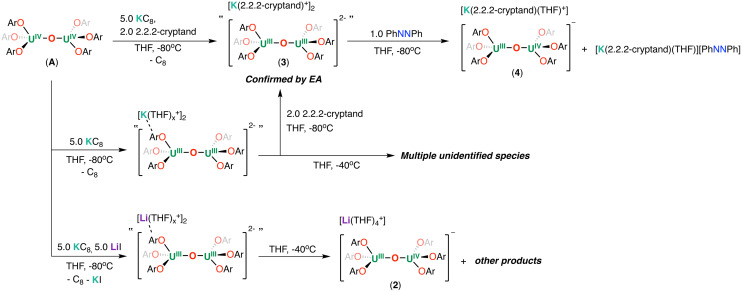
Reduction of A and reactivity of 3 with azobenzene.

Lastly, to isolate the U(iii)–O–U(iii) product, we performed the reduction of A in presence of 2.2.2-cryptand. The addition of 2.0 equiv. of 2.2.2-cryptand and 5.0 equivalents of KC_8_ to A at −80 °C, resulted in the formation of a new set of resonances in the ^1^H NMR spectrum (Fig. S15†) recorded at −80 °C that were assigned to the U(iii)/U(iii) [K(2.2.2-cryptand)]_2_[(U(OAr)_3_)_2_(μ-O)] (3) (Scheme 2). Decomposition of complex 3 was observed by ^1^H NMR spectroscopy after a few hours at −40 °C (Fig. S17†), but it could be isolated analytically pure in 80% yield from washing with cold toluene and *n*-hexane at −80 °C.

The formulation of complex 3 was confirmed by the isolation of the U(iii)–O–U(iv) analogue from redox reactivity studies. The reaction of complex 3 with PhNNPh resulted in the isolation of the U(iii)–O–U(iv) analogue oxide-bridged complex while the reduced azobenzene radical remains outer-sphere. The addition of 1.0 equiv. of PhNNPh to a solution of 3 in THF-*d*_8_ at −80 °C, led immediately to a colour change from dark red-brown to dark yellow-brown, full consumption of the starting material and the formation of a new species as indicated by ^1^H NMR spectroscopy (Fig. S18†). Single crystals suitable for X-ray diffraction studies were obtained from concentrated THF solution at −40 °C, and identified as the complex [K(2.2.2-cryptand)(THF)][(U(OAr)_3_)_2_(μ-O)] (4), co-crystallized with singly reduced [K(2.2.2-cryptand)(THF)][PhNNPh] in 52% yield (Scheme 2).

**Fig. 4:**
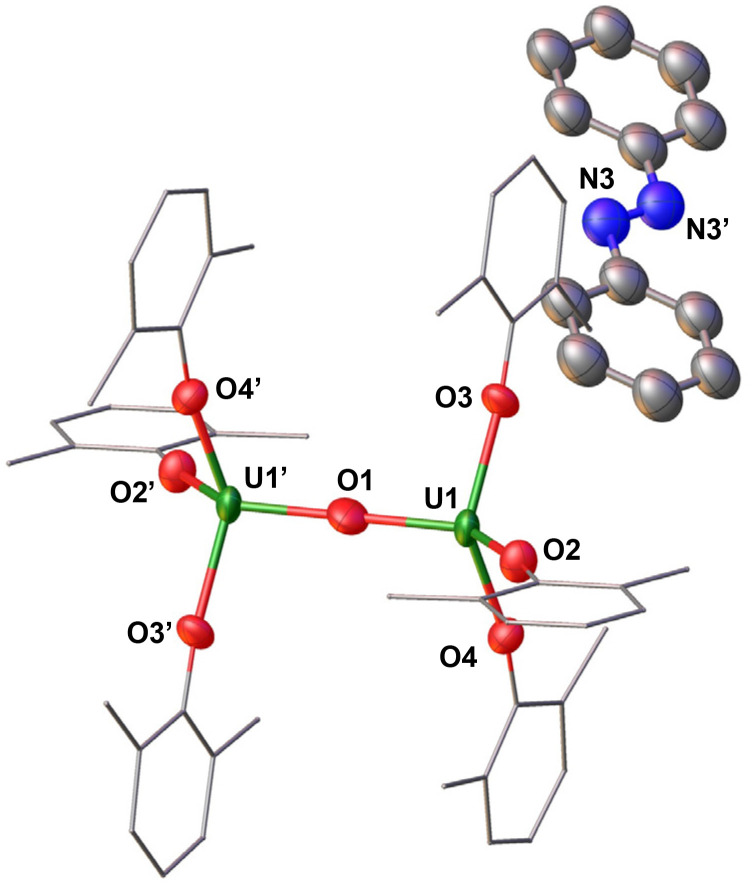
Molecular structure of the anionic species [(U(OAr)_3_)_2_(μ-O)]^−^ in (4), co-crystallized with [PhNNPh]^−^, with portions of the aryloxide ligands depicted as wire frames for clarity. Thermal ellipsoids are drawn at the 50% probability level. Two [K(2.2.2-cryptand)(THF)]^+^ cations, hydrogen atoms, and the methyl groups on the –OAr ligands have been omitted for clarity.

Complex 4 crystallizes in the space group *P*1̄, with the full molecule generated by symmetry. The solid-state structure of 4 (Fig. 4) was found to be very similar to the lithium analogue (2), with one outer-sphere [K(2.2.2-cryptand)(THF)]^+^ cations instead of [Li(THF)_4_]^+^ cation. The U–O_Ar_ distances 2.204(6)–2.223(6) and the U–O_oxide_ distances 2.1421(7) are comparable to those observed in U(iii)/U(iv) complex 2. The O_Ar_–U–O_Ar_ and O_ox_ide__–U–O_Ar_ bond angles (95.5(2)–137.6(2)°) are comparable to those found in 2. The U–O–U angle (180.0°) remains consistent with the linear arrangement observed in 2. Additionally, the N–N bond length (1.3(1) Å) of singly reduced [K(2.2.2-cryptand)(THF)][PhNNPh] is equivalent to that reported for the anionic compound, [K(2.2.2-cryptand)][PhNNPh] (1.34(3) Å).^49^ The N–N bond distance, being longer than neutral PhNNPh (1.25 Å) and shorter than the dianionic analogue [K(18-c-6)]_2_[PhNNPh] (1.40(3) Å),^27^ is diagnostic of the radical monoanionic nature of the anion [PhNNPh]^−^.

The reactivity of the U(iii)–O–U(iii) complex 3 is different from that previously reported for the analogous amide complex [K(2.2.2-cryptand)]_2_[(U(N(SiMe_3_)_2_)_3_)_2_(μ-O)],^20^ in which the reaction with azobenzene leads to cleavage of a U–O bond followed by four-electron reduction and cleavage of the N–N bond (yielding a U(vi) bis-imido complex) and the release of a U(iv) terminal oxide complex. Considering the very similar redox properties measured for complex 3 and the amide-supported U(iii)–O–U(iii) complex (see Electrochemistry section), the difference in reactivity is probably determined by the high stability of the terminal oxide complex [U(O)(N(SiMe_3_)_2_)_3_] compared to the aryloxide analogue.

Additionally, complex 3, similarly to what observed for [K(2.2.2-cryptand)]_2_[(U(N(SiMe_3_)_2_)_3_)_2_(μ-O)],^20^ shows no reactivity towards N_2_ reduction. This lack of reactivity contrasts with what was previously reported for the analogous oxide-bridged diuranium(iii) complex supported by siloxides ligands [(U(OSi(O^*t*^Bu)_3_)_3_)_2_(μ-O)]^23,39,40^ and can be explained by the lower reducing ability of 3 compared to the siloxide complex (see Electrochemistry section).

#### Sulphide-bridged complexes

The reduction of the sulphide complex B was also pursued and led to the isolation of the first example of a U(iii)/U(iii) sulphide-bridged complex.

Similar to the reduction of the oxide complex A, the addition of 2.0 equiv. of KC_8_ to B at −80 °C in THF-*d*_8_ resulted in a mixture of U(iii)/U(iv), “[K(THF)_*x*_][(U(OAr)_3_)_2_(μ-S)]” and U(iii)/U(iii), “[K(THF)_*x*_]_2_[(U(OAr)_3_)_2_(μ-S)]” complexes as indicated by ^1^H NMR spectroscopy (Fig. S20†). The addition of excess 3.0 equiv. of KC_8_ (5.0 equiv. in total) to B at −80 °C in THF-*d*_8_ led to the full consumption of U(iii)/U(iv), “[K(THF)_*x*_][(U(OAr)_3_)_2_(μ-S)], and the concomitant formation of a putative U(iii)/U(iii), complex “[K(THF)_*x*_]_2_[(U(OAr)_3_)_2_(μ-S)]” and KOAr, as observed by ^1^H NMR spectroscopy (Fig. S20†). Attempts to isolate single crystals of the putative “[K(THF)_*x*_]_2_[(U(OAr)_3_)_2_(μ-S)]” for XRD analysis were not successful. In addition, storing the reaction mixture in THF-*d*_8_ at −40 °C for 3 weeks resulted in the full decomposition of “[K(THF)_*x*_]_2_[(U(OAr)_3_)_2_(μ-S)]” and the formation of [KU(OAr)_4_], KOAr, and other species, as observed by ^1^H NMR spectroscopy (Fig. S21†).

**Scheme 3:**
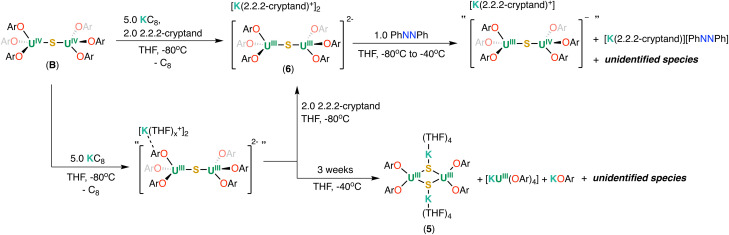
Reduction of B and reactivity of 6 with azobenzene.

Only a few dark crystals suitable for X-ray crystallography analysis of the complex [(K(THF)_4_)_2_(U(OAr)_2_)_2_(μ-S)_2_] (5) (Scheme 3) were obtained from a concentrated THF/*n*-hexane mixture at −40 °C. Attempts to isolate analytically pure complex 5 by changing different recrystallization conditions proved unsuccessful due to the similar solubility of the 5, [KU(OAr)_4_] and KOAr.

**Fig. 5:**
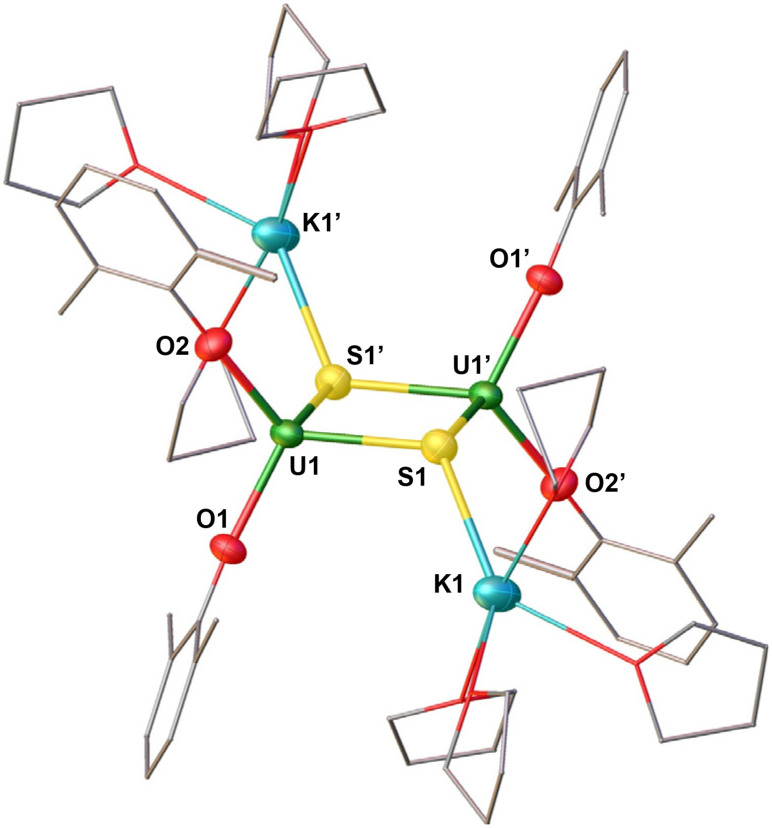
Molecular structure of [(K(THF)_4_)_2_(U(OAr)_2_)_2_(μ-S)_2_] (5) with portions of the aryloxide ligands and THF ligands depicted as wire frames for clarity. Thermal ellipsoids are drawn at the 50% probability level. Hydrogen atoms and methyl groups on the –OAr ligands have been omitted for clarity.

Complex 5 crystallizes in the space group *P*1̄, with the full molecule generated by inversion symmetry. The solid-state structure of 5 (Fig. 5) shows a dinuclear complex consisting of two equivalent U(iii) moieties, bridged by two sulphide ligands. Each uranium centre is tetra-coordinated in a distorted tetrahedral geometry and is bound by two –OAr ligands and the two bridging sulphides. Two K^+^ cations are bound by one sulphide ligand and four THF molecules. Notably, one K^+^ ion is above the U_2_S_2_ plane, while the other one is below the plane. The U–O_Ar_ bond distances (2.2191(19)–2.220(2) Å) are longer than those found in the U(iv) precursor, [(U(OAr)_3_)_2_(μ-S)] (U–O_Ar_: 2.079(9)–2.125(8) Å).^7^ The U–S distances (2.6946(7)–2.6988(8) Å) are longer than those reported for uranium(iv) sulphides dimers supported by cyclopentadienyl ligands and –OSi(O^*t*^Bu)_3_ ligands, [(η^5^-1,3-R_2_C_5_H_3_)_2_U_2_(μ-S)_2_] (R = Me_3_C or Me_3_Si) (2.605(2)–2.612(1) Å)^50,51^ and [Cs(THF)_2_(U(OSi(O^*t*^Bu)_3_)_3_)_2_(μ-S)_2_] (2.639(3)–2.677(3) Å).^38^ The values of the U–S–U angles are equivalent in 5 (91.52(2)°) and are smaller than those reported for the bis-sulphide U(iv) complexes (95.19(7)–98.34(10)°).^38,50,51^ Moreover, the O_Ar_–U–O_Ar_, S–U–O_Ar_ and S–U–S bond angles are in the range of 88.48(2)–119.24(6)°.

As with the oxide system, performing the reduction of B in the presence of 2.2.2-cryptand allowed the isolation of the desired U(iii) sulphide complex. Upon addition of 2.0 equiv. of 2.2.2-cryptand and 5.0 equivalents of KC_8_ to B at −80 °C, a new set of resonances appeared in the ^1^H NMR spectrum (Fig. S23†) recorded at −80 °C. Eventually, dark single crystals suitable for X-ray diffraction studies were obtained in 81% yield from a concentrated THF/*n*-hexane mixture at −40 °C, and were identified as the complex [K(2.2.2-cryptand)]_2_[(U(OAr)_3_)_2_(μ-S)] (6) (Scheme 3).

Complex 6 is stable at −40 °C in THF-*d*_8_ for up to 1 week as showed by ^1^H NMR spectroscopy (Fig. S25†), suggesting that the encapsulation of the cations in the cryptand prevents their binding to the ligands and rearrangement processes from occurring.

**Fig. 6:**
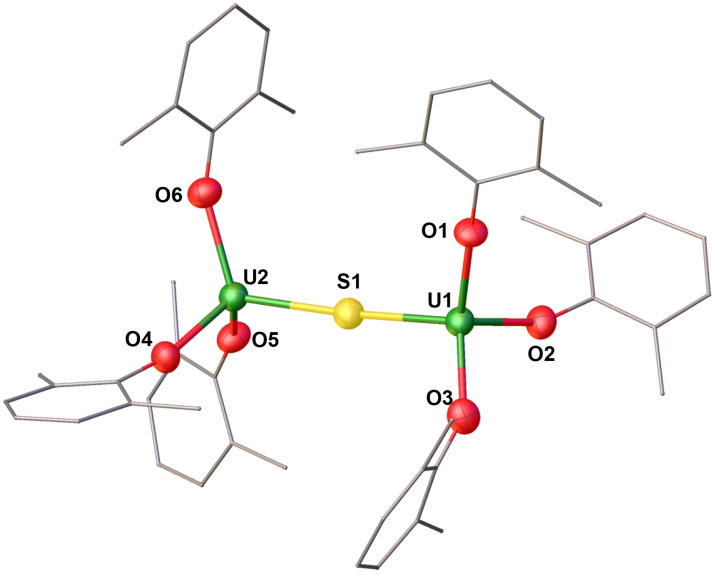
Molecular structure of the dianionic species [(U(OAr)_3_)_2_(μ-S)]^2−^ in (6) with portions of the aryloxide ligands depicted as wire frames for clarity. Thermal ellipsoids are drawn at the 50% probability level. Two [K(2.2.2-cryptand)]^+^ cations, hydrogen atoms, and methyl groups on the –OAr ligands have been omitted for clarity.

Complex 6 crystallizes in the triclinic space group *P*1̄. The solid-state molecular structure of complex 6 (Fig. 6 and Table 1) shows an ion pair consisting of two [K(2.2.2-cryptand)]^+^ cations and the [(U(OAr)_3_)_2_(μ-S)]^2−^ dianion. The two uranium(iii) ions in 6 are bridged by a sulphide (S^2−^) ligand, and each is bound by three –OAr ligands. The U–O_Ar_ bond distances (2.236(4)–2.250(4) Å) are longer compared to the U(iv) precursor, [(U(OAr)_3_)_2_(μ-S)] (2.079(9)–2.125(8) Å)^7^ and the previously reported U(iv) [(((^*t*-Bu^ArO)_3_tacn)U)_2_(μ-S)] (2.203(4) Å)^33^ supported by polydentate aryloxide ligand, but are comparable to complex 5 (2.2191(19)–2.220(2) Å). The U–S bond distances (2.6612(16)–2.6668(16) Å) are longer than those found in [(U(OAr)_3_)_2_(μ-S)] (2.5881 Å) and [(((^*t*-Bu^ArO)_3_tacn)U)_2_(μ-S)] (2.592(6) Å), but slightly shorter than those in complex 5 (2.6946(7)–2.6988(8) Å). The U–S–U bond angle (173.56(7)°) is slightly smaller compared to those found in the precursor (180.0(1)°) and the reported U(iv)[(((^*t*-Bu^ArO)_3_tacn)U)_2_(μ-S)] (180.0°). The range of O_Ar_–U–O_Ar_ and S–U–O_Ar_ bond angles (105.93(16)–117.50(15)°) is smaller than that found in the precursor, B (92.1(2)–156.2(4)°).

**Table 1:** Selected bond lengths (Å) and angles (°) for complexes 1, 2, 4, 5 and 6; X = N, O, S

Complex	1	2	4	5	6
U–O_Ar_ (Å)	2.178(4)–2.226(4)	2.208(3)–2.246(3)	2.204(6)–2.223(6)	2.2191(19)–2.220(2)	2.236(4)–2.250(4)
U–X (Å)	2.0612(5)	2.15427(12)	2.1421(7)	2.6946(7)–2.6988(8)	2.6612(16)–2.6668(16)
U–X–U (°)	180.0	180.0	180.0	91.52(2)	173.56(7)

Remarkably, complexes 5 and 6 are the first examples of diuranium(iii) bridged by sulphide or bis-sulphide moieties. Previously, only diuranium(iv) bridged by sulphide or bis-sulphide ligands have been reported. Complex 6 showed similar reactivity with azobenzene as 3. The reaction of 6 with 1.0 equiv. of PhNNPh in THF at −80 °C, resulted in a mixture of unreacted 6 and a new species, as observed by ^1^H NMR spectroscopy at −80 °C (Fig. S26†). The full consumption of the starting material was observed when the reaction mixture was warmed up to −40 °C after 1 hour as indicated by ^1^H NMR spectroscopy (Fig. S26†). The ^1^H NMR spectrum of the reaction mixture showed the formation of a major species that was assigned as the mono-reduced U(iii)–S–U(iv), species suggesting that complex 6 had transferred one electron to the PhNNPh species as found for the U(iii)–O–U(iii) complex. No reaction with N_2_ was observed for complex 6.

### Electrochemistry

**Fig. 7:**
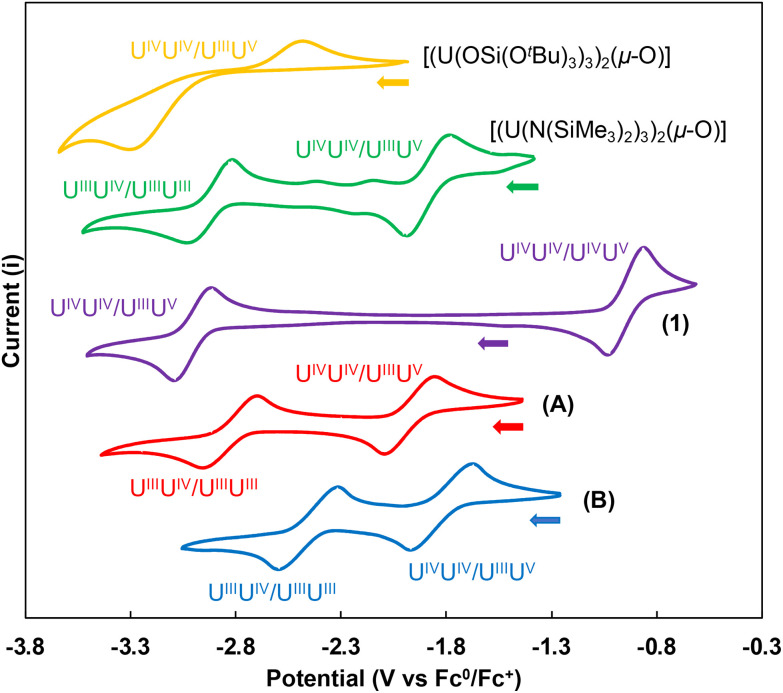
Cyclic voltammograms for complexes, A (red), B (blue), 1 (purple), [(U(N(SiMe_3_)_2_)_3_)_2_(μ-O)] (green) and [(U(OSi(O^*t*^Bu)_3_)_3_)_2_(μ-O)] (yellow) in THF. Conditions: Pt disk working electrode, referenced to the Fc^+/0^ couple; 0.1 M [NBu_4_][BPh_4_] electrolyte in THF. Scan rate (100 mV s^−1^). Arrows indicate the scan direction.

Cyclic voltammograms were measured for 3 mM THF solutions of complexes A, B, 1, and of previously reported [(U(OSi(O^*t*^Bu)_3_)_3_)_2_(μ-O)]^39^ and [(U(N(SiMe_3_)_2_)_3_)_2_(μ-O)]^8^ (Fig. 7) complexes (Table 2).

Electrochemical data in V *vs*. Fc^0^/Fc^+^ for the [(U(OSi(O^*t*^Bu)_3_)_3_)_2_(μ-O)], [(U(N(SiMe_3_)_2_)_3_)_2_(μ-O)], 1, A, and B complexes

**Table:** 

*E*_pc_	[(U(OSi(O^*t*^Bu)_3_)_3_)_2_(μ-O)]	[(U(N(SiMe_3_)_2_)_3_)_2_(μ-O)]	1	A	B
U^IV^U^IV^/U^III^U^IV^	−3.32	−1.99	−3.09	−2.09	−1.98
U^III^U^IV^/U^III^U^III^	—	−3.03	—	−2.96	−2.60
U^IV^U^V^/U^IV^U^IV^	—	—	−1.03	—	—

**Table:** 

*E*_pa_	[(U(OSi(O^*t*^Bu)_3_)_3_)_2_(μ-O)]	[(U(N(SiMe_3_)_2_)_3_)_2_(μ-O)]	1	A	B
U^III^U^III^/U^III^U^IV^	−2.48	−2.81	—	−2.70	−2.29
U^III^U^IV^/U^IV^U^IV^	—	−1.78	−2.90	−1.85	−1.66
U^IV^U^IV^/U^IV^U^V^	—	—	−0.84	—	—

The cyclic voltammogram for complex A in THF revealed two quasi-reversible redox events at values of *E*_1/2_ = −2.83, and −1.97 V *vs.* Fc^+/0^. The two measured waves display reduction events with *E*_pc_ values of −2.09 V and −2.96 V *vs.* Fc^+/0^, respectively. The event at −2.09 V *vs.* Fc^+/0^ is assigned to the reduction of the U(iv)–O–U(iv) complex to the U(iii)–O–U(iv) analogue, while the more negative event is assigned to the reduction of the mixed valent species to the U(iii)–O–U(iii) species with both events being in the range of values reported in the literature for the U(iv)/U(iii) couple.^52^ The large difference (0.86 V) between the redox potential measured for the two uranium centres indicate a strong degree of electronic communication between them. Large separation between the U(iv)/U(v), U(v)/U(v), and U(v)/U(vi) redox couples have been previously reported for bis-oxo bridged complexes supported by polydentate tris-aryloxide ligands [(((^*n*P,Me^ArO)_3_tacn)U^V^)_2_(μ-O)_2_] and were explained in terms of highly covalent U–O bonds in the bis(μ-oxo) diamond core that supports the electronic coupling.^53^

The values of reduction potential measured for A are significantly more positive than the value reported for the U(iii)/U(iv) (*E*_pc_ value at −3.32 V *vs.* Fc^+/0^) couple in the bridging oxide complexes supported by –OSi(O^*t*^Bu)_3_ ligands,^39^ indicating a lower reducing power of the U(iii)–O–U(iii) moiety when supported by aryloxide ligands compared to siloxides. However, these values are comparable to the two different U(iii)/U(iv) couples (*E*_pc_ values at −1.99 V and −3.03 V *vs.* Fc^+/0^) measured for the bridging oxide complex supported by –N(SiMe_3_)_2_ ligands.

Furthermore, the values of the reduction potentials of A (*E*_pc_ values at −2.09 V and −2.96 *vs.* Fc^+/0^) are more negative than those reported for the U(iii) monomeric complex, [U(OAr)_3_](OAr = 2,6-di-*tert*-butylphenoxide) (*E*_pc_ value at −1.30 V *vs.* Fc^+/0^),^7^ which, unlike 3, can reduce N_2_ in non-polar solvents. Unfortunately, the reactivity towards N_2_ could not be probed for complex 3 in non-polar solvents due to the low solubility. However, the lack of reactivity with N_2_ may also be due to the presence of aryloxide sterically hindering the U(iii)–O–U(iii) cavity, which prevents N_2_ side-on binding.

The voltammogram of B exhibits similar redox events, which can be assigned to the two U(iii)/U(iv) couples. The two reduction potentials for B (*E*_pc_ values at −1.98 and −2.60 V *vs.* Fc^+/0^) are more positive than those of A with a larger peak separation of 0.86 V for A compared to B (0.62 V) suggesting that the sulphide (S^2−^) linker decreases the reducing power of the complex compared to the oxide linker and promotes a weaker electronic communication.

In contrast, the voltammogram of 1 shows two distinct redox processes that are further apart compared to those observed for A and B. The first cathodic redox event (*E*_1/2_ = −3.0 V *vs.* Fc^+/0^) is assigned to the U(iii)/U(iv) couple while the second event (*E*_1/2_ = −0.94 V *vs.* Fc^+/0^) is assigned to the U(iv)/U(v) couple. Only the one-electron reduction to U(iii)/U(iv) is observable for 1 and is significantly shifted to more negative potentials compared to the oxide A and sulphide B complexes. These results indicate that the nature of both supporting ligands and single-atom linker affect significantly the redox potential of single-atom bridged dinuclear uranium complexes. Nitride linkers shift significantly the first reduction potential towards very negative values compared to sulphide and oxide which prevents the isolation of U(iii)/U(iii) complexes. The presence of siloxide ligands also shifts to very negative potentials the U(iv)/U(iii) reduction potential compared to amide and aryloxide ligands, but chemical reduction with KC_8_ allowed to isolate U(iii)–O–U(iii) complexes that showed the ability to reduce and even cleave dinitrogen,^23,35^ suggesting electronic stabilization provided by the multiple binding modes available to the siloxide ligands.

### Magnetic properties

**Fig. 8:**
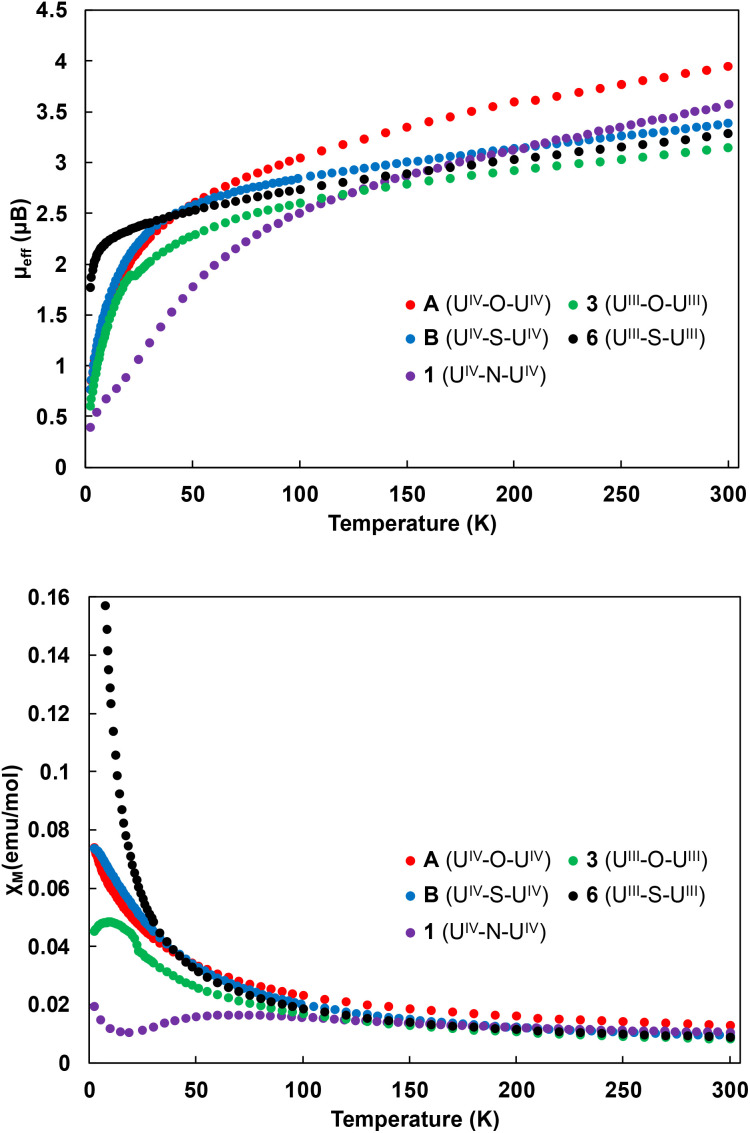
Temperature dependent SQUID magnetization data plotted as a function of *μ*_eff_*vs.* temperature (top), and *χ*_M_*vs.* temperature (bottom) for complexes A (red), B (blue), 1 (purple), 3 (green) and 6 (black) (measured under an applied field of 1 T).

To further investigate the electronic structures and bonding in the U(iv) oxide (A), sulphide (B), nitride (1), U(iii) oxide (3) and sulphide (6) complexes, variable temperature magnetometry experiments were performed for complexes A, B, 1, 3 and 6 under an applied magnetic field of 1 T (Fig. 8). The measured values of the magnetic moment per ion at 300 K are 3.94*μ*_B_ for A, 3.39*μ*_B_ for B, 3.57 for 1 and decrease smoothly to a low value of 0.77*μ*_B_ for A and B and 0.40*μ*_B_ for 1 at 2 K and tending to zero, which is typical of the magnetic singlet 5f^2^ uranium(iv) ions subject to modest temperature-independent paramagnetism.^54–56^ In the case of 6, the magnetic moment per ion is 3.29*μ*_B_ at 300 K. The temperature dependence shows a smooth decrease until 10 K, and a steeper one below that temperature, reaching a value of 1.77*μ*_B_ at 2 K. This trend is characteristic of uranium(iii) ions, owing to the ^4^I_9/2_ ground state of the f^3^ ion.^55,57^ On the other hand, complex 3 possesses a magnetic moment per ion at 300 K of 3.15*μ*_B_, which decreases to a low value of 0.60*μ*_B_ at 2 K. Similar low values at 2 K have previously been observed even in U(iii) complexes.^55^

The temperature dependence of the magnetic susceptibility (Fig. 8), for complexes A, B and 6 shows its continuous increase when decreasing the temperature, as expected for isolated uranium(iv) (A, B)^33,53,58,59^ and uranium(iii)^55,57^ (6) ions. On the other hand, the U(iv) ions in complex 1 are antiferromagnetically coupled, as evidenced by the maximum at 70 K in the *χ*_M_*vs. T* plot. Unambiguous antiferromagnetic coupling is very rare for U(iv)–U(iv) complexes^2,33^ and is usually weaker, but we recently reported a similar behaviour with a maximum at 90 K in the *χ*_M_*vs. T* plot for the uranium(iv) nitride-bridged complex [NBu_4_][(U(N(SiMe_3_)_2_)_3_)_2_(μ-N)].^32^ Interestingly, complex 3 also exhibits a maximum at 9 K in the *χ*_M_*vs. T* plot (Fig. 8), this maximum and downturn suggest the presence of antiferromagnetic exchange coupling, but the contribution of the single-ion crystal field effect cannot be ruled out.^2^

Dinuclear complexes of uranium(iii) are rare and only a few examples of magnetic communication between U(iii) centres have been reported.^19,39,60^ These include the diuranium(iii) bridging nitride and oxide complexes, [K_3_(U^III^(OSi(O^*t*^Bu)_3_)_3_)_2_(μ-N)] and [Cs_2_(U^III^(OSi(O^*t*^Bu)_3_)_3_)_2_(μ-O)],^19,39^ which showed antiferromagnetic coupling with a maximum at 23 K and 20 K, respectively. Stronger antiferromagnetic coupling with the highest value of *χ*_M_ at 110 K was reported for an arene-bridged U(iii) dimer.^25^

### Computational studies

**Fig. 9:**
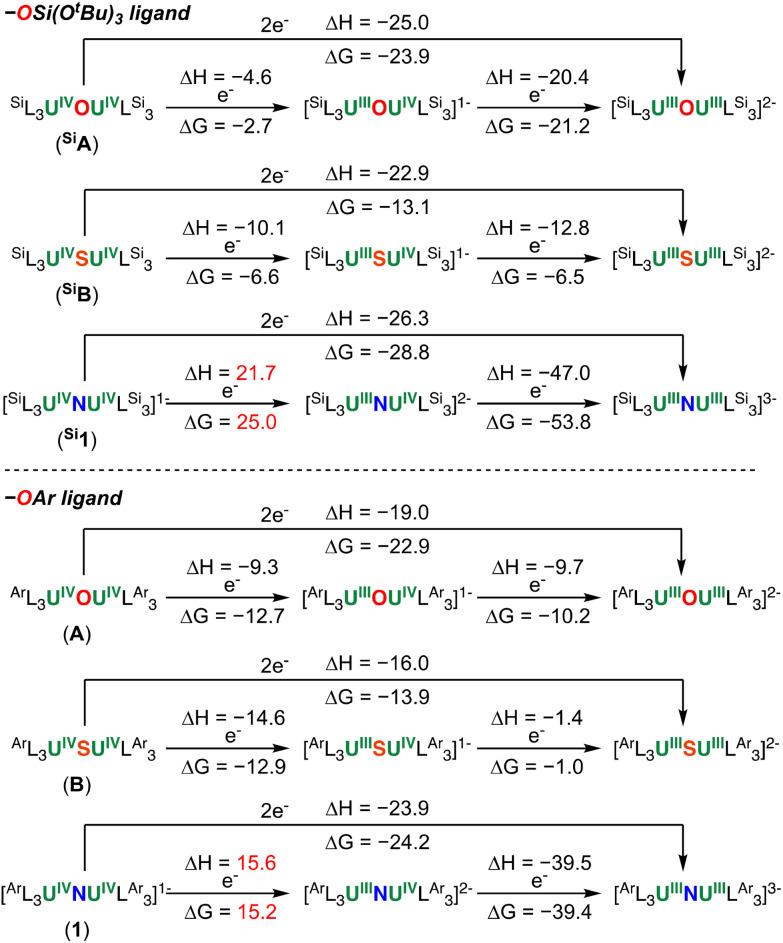
Computed reduction reactions of complexes ^Si^A, ^Si^B, ^Si^1, A, B and 1 by KC_8_. The energies are given in kcal mol^−1^ at 298 K.

To gain some insights into the reduction behaviour of complexes A, B and 1, DFT calculations (B3PW91) including solvent and dispersion corrections were carried out. For the sake of comparison, the tris(*tert*-butoxy)siloxide equivalent of these three complexes were also computed (labelled ^Si^A, ^Si^B and ^Si^1). The reduction of the complexes with KC_8_ was computed as two successive single electron reduction with the formation of a mixed valence intermediate complex (Fig. 9).

The two-electron reduction is computed to be thermodynamically favourable for all complexes. However, a closer look at the results of Fig. 9 clearly shows that first the reductions are easier for aryloxide complexes than for the siloxide ones, in line with the experimentally observed higher reducing power of the siloxide complexes. Secondly, it appears that the reduction of the nitride complexes is more complicated than the oxides and sulphides, since the first electron transfer reaction is endothermic (endergonic), which is again in line with experiment. The latter can be easily explained by analysing the boding situation in the complexes A, B and 1 using Natural Bonding Orbital (NBO) analysis. In the starting complexes, NBO indicates presence of two single U–X bonds which are strongly polarized toward X (82 to 91%) so that bonding seems similar at first sight. However, at the second order donor–acceptor, the nitride complex is the only one to display large donation from nitrogen lone pairs to empty uranium orbitals, resulting in electron delocalization in between the nitride and the two uranium. This delocalization strengthens the U–N bonds as evidence by the Wiberg Bond Indexes (WBI) which are around 1 for O and S (0.79 and 1.03) while it grows to 1.22 for N. This multiple bond character of two U–N bonds involves more uranium orbital in bonding and therefore less prompted to accept electrons, making it less reducible. The difference of reducing power between the siloxide and aryloxide ligand was also analysed using bonding analysis. It is noteworthy that the nature of U–O_Ar_ and U–O_Si_ are quite different in the different U–O–U complexes. Indeed, as reflected in the Natural Charges (see ESI†), the U–O_Si_ bond appears to much more ionic than the U–O_Ar_ ones. Indeed, the oxygen charges in the former is around −1.2 while is it only −0.8 in the latter complexes. Since the charge of uranium centres are similar with the two sets of ligands, it clearly means that the empty f orbitals at uranium are higher in energy in the U(iv)–O–U(iv) siloxide compared to the U(iv)–O–U(iv) aryloxide case making this complex more difficult to reduce as observed experimentally.

## Conclusions

In summary, we reported the synthesis, redox and magnetic properties of a series of N^3−^, O^2−^, and S^2−^ bridged diuranium complexes supported by bulky aryloxide ligands and compared their properties to analogous complexes supported by siloxides. The U(iv)/U(iv) nitride [Cs(THF)_8_][(U(OAr)_3_)_2_(μ-N)], 1 could be prepared and characterized but could not be chemically reduced. Reduction of the neutral U(iv)/U(iv) complexes [(U(OAr)_3_)_2_(μ-X)], A (X = O) and B (X = S) led to the isolation and characterization of rare U(iii)/U(iv) and U(iii)/U(iii) analogues. Notably, complexes [(K(THF)_4_)_2_(U(OAr)_2_)_2_(μ-S)_2_], 5 and [K(2.2.2-cryptand)]_2_[(U(OAr)_3_)_2_(μ-S)], 6 are the first examples of U(iii) sulphide bridged complexes. The redox properties of diuranium complexes are significantly different for siloxide and aryloxide supported complexes and indicate a significantly stronger reducing power for the siloxide complexes. In contrast, the easily accessible first U(iii)/U(iv) couple facilitate one-electron transfer reactions to substrates such as azobenzene for the aryloxide supported diuranium complexes. Computational studies show that the nature of U–O_Ar_ and U–O_Si_ are quite different in the U–O–U complexes with the U–O_Si_ bond more ionic than the U–O_Ar_ ones. As a result, the empty f uranium orbitals are found higher in energy in the U(iv)–O–U(iv) siloxide compared to the U(iv)–O–U(iv) aryloxide rendering the analogue U(iii)–O–U(iii) significantly more reducing. The redox behaviour of the oxide- and sulphide-bridged complexes is similar while reduction of the nitride bridged complexes is more complicated due to the significant multiple bond character of the U–N–U bridge. Such different interaction is corroborated by the observed magnetic communication for the U(iv)–N–U(iv) complex compared to the U(iv)–O–U(iv) and U(iv)–S–U(iv) systems, showing a magnetic behaviour typical of isolated U(iv) ions. Finally the data reported here show that both single-atom linkers and supporting ligands can be used to tune magnetic communication and redox reactivity in diuranium complexes.

## Author contributions

F.-C. H. designed and performed all the experiments, analysed the data, prepared all the figure and wrote the manuscript; L. B. isolated and characterised 1; T. R. and L. M. performed the computational studies, analysed them and wrote the computational section; I. Z. measured and analysed the magnetic data; R. S. measured and analysed the XRD data; M. M. conceived and supervised the project, analysed the data, wrote the manuscript.

## Data availability

Synthetic details, analytical data including depictions of all spectra and coordinate data of all computationally optimised species, are documented in the ESI.† Crystallographic data is made available *via* the CCDC. The data that support the findings of this study are openly available in the Zenodo repository at https://doi.org/10.5281/zenodo.12191401.

## Conflicts of interest

There are no conflicts to declare.

## Supplementary Material

DT-053-D4DT01819B-s001

DT-053-D4DT01819B-s002
