# Supplementary material for: Synthesis, structure and redox properties of single-atom bridged diuranium complexes supported by aryloxides
Source: Dalton Trans. 2024 Jul 23;53(32):13416–26. doi: 10.1039/d4dt01819b (PMC11320667; doi:10.1039/d4dt01819b)
Supplement: DT-053-D4DT01819B-s001 [file DT-053-D4DT01819B-s001.pdf]

Electronic Supplementary Material (ESI) for Dalton Transactions.
This journal is © The Royal Society of Chemistry 2024

Synthesis, structure and redox properties of single-atom bridged diuranium complexes supported by aryloxides

Fang-Che Hsueh,[a] Luciano Barluzzi,[a] T. Rajeshkumar[b], Rosario Scopelliti,[c] Ivica Zivcovic,[d] Laurent Maron[b*] and Marinella Mazzanti[a*]

[a] F.-C. Hsueh, L. Barluzzi, Prof. M. Mazzanti
Group of Coordination Chemistry, Institut des Sciences et Ingénierie Chimiques, École Polytechnique Fédérale de Lausanne (EPFL), 1015, Lausanne, Switzerland. Email: marinella.mazzanti@epfl.ch.

[b]. T. Rajeshkumar, Prof. L. Maron
Laboratoire de Physique et Chimie des Nano-objets, Institut National des Sciences Appliquées, 31077 Toulouse, Cedex 4, France.

[c] Dr. R. Scopelliti ,Institut des Sciences et Ingénierie Chimiques, École Polytechnique Fédérale de Lausanne (EPFL), 1015, Lausanne, Switzerland.

[d] Laboratory for Quantum Magnetism, Institute of Physics, Ecole Polytechnique Fédérale de Lausanne (EPFL), CH-1015 Lausanne, Switzerland.

*Email to whom correspondence should be addressed: marinella.mazzanti@epfl.ch

Table of Contents

S1. Materials and Physical Measurements 3

S2. Synthesis 5

S3. NMR Spectroscopic Data 10

S4. Electrochemistry Data 36

S5. SQUID Magnetometry Data 38

S6. X-ray Crystallography Data 46

S7. Computational Details 48

S8. References 139

Experimental Procedures

S1. Materials and Physical Measurements

General Considerations

Unless otherwise noted, all manipulations were carried out at ambient temperature under an inert argon or nitrogen atmosphere using Schlenk techniques and an MBraun glovebox equipped with a purifier unit. The water and oxygen levels were always kept at less than 0.1 ppm. Glassware was dried overnight at 140 °C before use.

NMR experiments were carried out using NMR tubes adapted with J-Young valves. NMR spectra were recorded on Bruker 400 MHz spectrometers and referenced to residual solvent resonances of THF (d_8-THF) and toluene (d_8-toluene).

Elemental analyses were performed under an inert atmosphere of nitrogen with a ThermoScientific Flash 2000 Organic Elemental Analyzer.

Cyclic voltammetry data were carried out at room temperature in an argon-filled glovebox described above. Data were collected using a Biologic SP-300 potentiostat connected to a personal computer. All samples were measured with 0.1 M $[NBu_4][BPh_4]$ supporting electrolyte in THF solution. The experiments were carried out with a platinum disk (d = 5 mm) working electrode, a platinum wire counter electrode, and an Ag/AgCl reference electrode. Potential calibration was performed at the end of each data collection cycle using the decamethylferrocene (Fc*) /decamethylferrocenium couple as an internal standard.

Magnetic measurements were performed using a Quantum Design MPMS3 superconducting quantum interference device (SQUID) magnetometer in a temperature range 2-300 K. The powder sample was enclosed in an evacuated quartz capsule and placed inside a plastic straw. The measurements were performed with applied magnetic field of 1T in the zero-field cooled (ZFC) regime. Diamagnetic corrections were applied using Pascal's constants[1]. The magnetic moment per uranium ion was calculated using the formula:

$$\mu_{eff} = \sqrt{\frac{8\chi T}{2}}$$

Starting materials. Unless otherwise noted, reagents were purchased from commercial suppliers and used without further purification. Anhydrous solvents were purchased from Aldrich and further distilled from K/benzophenone (THF, Et_2O and toluene), sodium sand/benzophenone (*n*-hexane). Deuterated solvents for NMR spectroscopy were purchased from Cortecnet, freeze-degassed and distilled over K/benzophenone (THF-d_8, toluene-d_8). Azobenzene (PhNNPh), triphenylphosphine sulfide (Ph_3PS), 2,6-di-*tert*-butylphenol (HOAr), 2.2.2-cryptand, and caesium azide (CsN_3) were purchased from Sigma-Aldrich and dried under high vacuum prior to use. Depleted uranium was purchased from Ibilabs, Florida, USA. KC_8[2], $IMesN_2O$[3], KOAr[4], $[U(OAr)_3)]$[5], and $[U(OAr)_4)]$[4] were prepared according to the published procedure. $[(U(OAr)_3)_2(\mu\text{-}O)]$ (**A**) and $[(U(OAr)_3)_2(\mu\text{-}S)]$ **(B)** were synthesized using a modified published procedure[6].

Caution: Depleted uranium (primary isotope ^{238}U) is a weak a-emitter (4.197 MeV) with a half-life of 4.47×10^9 years. Manipulations and reactions should be carried out in monitored fume hoods or in an inert atmosphere glovebox in a radiation laboratory equipped with α- and β-counting equipment.

X-ray crystallography data for the analyzed crystal structures were selected and mounted on various Rigaku diffractometers (XtaLAB Synergy R, DW system, HyPix-Arc 150 detector or SuperNova, Dual, Cu at home/near, AtlasS type detectors). The crystals were kept at a steady T = 140.00(10) K during data collection. Data were measured using ω scans with Cu K_α radiation. The diffraction patterns were indexed and the total number of runs and images were based on the strategy calculation from the program CrysAlisPro 1.171.42.72a (Rigaku OD, 2022)[7]. The unit cells were refined using CrysAlisPro 1.171.42.72a (Rigaku OD, 2022)[7]. Data reduction, scaling and absorption corrections were performed using CrysAlisPro 1.171.42.72a (Rigaku OD, 2022)[7]. The structures were solved with the **ShelXT** (Sheldrick, 2015)[8] solution program using dual methods and by using **Olex2** 1.5 (Dolomanov et al., 2009)[9] as the graphical interface. The models were refined with **ShelXL** 2018/3 (Sheldrick, 2015)[10] using full matrix least squares minimization on ***F*2**. All non-hydrogen atoms were refined anisotropically. The positions of the hydrogen atom were calculated geometrically and refined using the riding model. Several structures displayed problems dealing with disorder (disordered ligands or solvent) or twinning. The major employed technique was the split model combined with a series of restraints and constraints. The restraints and constraints are used in order to get acceptable bond lengths and angles and/or anisotropic behavior. In some cases, the twinning treatment has been used for real twins or for multi-crystals, in order to properly separate the different domains. Finally, in some structures the solvent molecules were difficult to handle and the mask algorithm (by Olex2) was used to squeeze them completely from the final model. **CCDC deposition numbers** (https://www.ccdc.cam.ac.uk/services/structures?id=doi:10.1002/anie.202317346) 2362157 (complex **1**), 2362158 (complex **2**), 2362159 (complex **4**), 2362160 (complex **5**), 2362161 (complex **6**), contain the supplementary crystallographic data for this paper. These data are provided free of charge by the joint Cambridge Crystallographic Data Centre and Fachinformationszentrum Karlsruhe (http://www.ccdc.cam.ac.uk/structures).

S2. Synthesis

Synthesis of [(U(OAr)$_3$)$_2$(μ-O)] (A)

A yellow suspension of IMesN$_2$O (44.0 mg, 0.126 mmol, 0.5 equiv.) in THF (2.5 mL) was added to a dark green solution of complex [U(OAr)$_3$] (215.8 mg, 0.2526 mmol, 1.0 equiv.) in THF (2.5 mL) at -80 °C. N$_2$ evolution was observed. The reaction mixture was allowed to react for 5 hours at -80 °C, yielding a dark yellow solution. The volatiles were removed under vacuum and the residue was dissolved in a mixture of toluene (5.0 mL) and Et$_2$O (1.0 mL). The resulting solution was stored at -40 °C overnight affording a yellow powder. The resulting powder was washed with cold Et$_2$O (1.0 mL), affording product **A** (175.3 mg, 80%). Multiple attempts to isolate single crystals of **A** for X-Ray diffraction studies proved unsuccessful due to the poor crystal quality. Analytical data matched those previously reported[6]. ^{1}H NMR (400 MHz, THF-d_8, 298 K): δ 14.20 ppm (s, 12H, –OAr), δ 12.17 ppm (s, 6H, –OAr), δ -11.26 (br s, 108H, –C(CH$_3$)$_3$)(**Figure S2**). *Anal. Calcd.* for **A**, $C_{84}H_{126}O_7U_2$: C: 58.52; H: 7.37; N: 0.00. *Found*: C: 58.13; H: 7.21; N: 0.00.

Synthesis of [(U(OAr)$_3$)$_2$(μ-S)] (B)

A suspension of Ph$_3$PS (35.4 mg, 0.120 mmol, 0.5 equiv.) in toluene (2.0 mL) was added to a dark green solution of complex [U(OAr)$_3$] (205.0 mg, 0.240 mmol, 1.0 equiv.) in toluene (1.0 mL) at room temperature. The reaction mixture was stirred for 3 hours at room temperature, yielding a dark orange solution. The volatiles were removed under vacuum and the residue was dissolved in a mixture of toluene (2.0 mL) and Et$_2$O (1.0 mL). The resulting solution was stored at -40 °C overnight affording orange powder. The resulting powder was washed with cold Et$_2$O (1.0 mL), affording product **B** (190.4 mg, 91%). Analytical data matched those previously reported[6]. ^{1}H NMR (400 MHz, toluene-d_8, 298 K): δ 15.34 ppm (d, J = 8.1 Hz, 12H, –OAr), δ 11.75 ppm (t, J = 7.9 Hz, 6H, –OAr), δ -8.67 (s, 108H, –C(CH$_3$)$_3$) (**Figure S4**). *Anal. Calcd.* for **B**, $C_{84}H_{126}O_6SU_2$: C: 57.98; H: 7.30; N: 0.00; S: 1.84. *Found*: C: 57.93; H: 7.06; N: 0.00; S: 1.78.

Synthesis of [Cs(THF)$_8$][(U(OAr)$_3$)$_2$(μ-N)] (1)

A dark green solution of complex [U(OAr)$_3$] (84.9 mg, 0.0994 mmol, 1.0 equiv.) in THF (1.0 mL) was added to a suspension of CsN$_3$ (8.7 mg, 0.050 mmol, 0.5 equiv.) in THF (1.0 mL) at -40 °C. The reaction mixture was stirred for 4 days at -40 °C, yielding a dark orange solution. The volatiles were removed under vacuum and the residue was dissolved in a mixture of THF (2.0 mL) and *n*-hexane (1.0 mL). The resulting solution was stored at -40 °C overnight affording orange powder of complex **1** (93.9 mg, 88%). X-ray quality crystals **1** were obtained by cooling a concentrated THF/*n*-hexane solution to -40 °C. ^{1}H NMR (400 MHz, THF-d_8, 298 K): δ 5.74 ppm, δ -8.54 ppm (**Figure S6**). ^{1}H NMR (400 MHz, THF-d_8, 233 K): δ 96.95 ppm, δ -19.72 ppm, δ -39.15 ppm (**Figure S7**). *Anal. Calcd.* for [Cs(THF)$_5$][(U(OAr)$_3$)$_2$(μ-N)], $C_{104}H_{166}CsNO_{11}U_2$: C: 56.38; H: 7.55; N: 0.63. *Found*: C: 56.70; H: 7.52; N: 0.84. The caesium bound THF was lost during the drying process.

Reduction of [Cs(THF)$_8$][(U(OAr)$_3$)$_2$(μ-N)] (1) with 5.0 equiv. of KC$_8$ at -80 °C (NMR test reaction)

A dark orange solution of complex **1** (10.1 mg, 0.00488 mmol, 1.0 equiv.) in THF-d_8 (0.25 mL) was added to a bronze suspension of KC$_8$ (3.3 mg, 0.024 mmol, 5.0 equiv.) in THF-d_8 (0.15 mL) at -80 °C. The reaction mixture was allowed to react for 6 hours at -80 °C. The ^{1}H NMR spectrum of the reaction mixture showed resonances assigned to **1** and unidentified species (**Figure S8**).

Reduction of $[Cs(THF)_8][(U(OAr)_3)_2(\mu\text{-}N)]$ (1) with 1.0-2.0 equiv. of KC_8 at -40 °C (NMR test reaction)

A dark orange solution of complex **1** (15.3 mg, 0.00739 mmol, 1.0 equiv.) in THF-d_8 (0.25 mL) was added to a bronze suspension of KC_8 (1.0 mg, 0.00739 mmol, 1.0 equiv.) in THF-d_8 (0.15 mL) at -40 °C. The reaction mixture was allowed to react for 30 minutes at -40 °C. The ^{1}H NMR spectrum of the reaction mixture showed resonances assigned to **1** and a new species (**Figure S9**). A bronze suspension of KC_8 (1.0 mg, 0.00739 mmol, 1.0 equiv.) in THF-d_8 (0.15 mL) was added to a stirring reaction mixture at -40 °C and stirred for 30 minutes at -40 °C. The ^{1}H NMR spectrum of the reaction mixture showed resonances assigned to a new species and the disappearance of those corresponding to complex **1** (**Figure S9**). The reaction mixture was filtered on a porosity 4 glass frit, yielding a dark brown solution. Attempts to isolate X-ray quality crystals were not successful preventing further characterization.

Reduction of $[Cs(THF)_8][(U(OAr)_3)_2(\mu\text{-}N)]$ (1) with 3.0 equiv. of KC_8 at -40 °C (NMR test reaction)

A dark orange solution of complex **1** (11.1 mg, 0.00536 mmol, 1.0 equiv.) in THF-d_8 (0.25 mL) was added to a bronze suspension of KC_8 (2.2 mg, 0.016 mmol, 3.0 equiv.) in THF-d_8 (0.15 mL) at -40 °C. The reaction mixture was allowed to react for 30 minutes at -40 °C. The ^{1}H NMR spectrum of the reaction mixture showed multiple unidentified resonances (**Figure S9**). Attempts to isolate X-ray quality crystals were not successful preventing further characterization.

Reduction of $[(U(OAr)_3)_2(\mu\text{-}O)]$ (A) with 1.0-5.0 equiv. of KC_8 at -80 °C (NMR test reaction)

A pale yellow solution of complex **A** (16.8 mg, 0.00974 mmol, 1.0 equiv.) in THF-d_8 (0.25 mL) was added to a bronze suspension of KC_8 (1.4 mg, 0.010 mmol, 1.0 equiv.) in THF-d_8 (0.15 mL) at -80 °C. The reaction mixture was allowed to react for 20 minutes at -80 °C. The ^{1}H NMR spectrum of the reaction mixture showed one set of resonances assigned to U^{III}/U^{IV} "$[K(THF)_x][(U(OAr)_3)_2(\mu\text{-}O)]$" (**Figure S10**). A bronze suspension of KC_8 (1.4 mg, 0.010 mmol, 1.0 equiv.) in THF-d_8 (0.15 mL) was added to reaction mixture and reacted for 20 minutes at -80 °C. The ^{1}H NMR spectrum of the reaction mixture showed two sets of resonances assigned to U^{III}/U^{IV} "$[K(THF)_x][(U(OAr)_3)_2(\mu\text{-}O)]$" and U^{III}/U^{III} "$[K(THF)_x]_2[(U(OAr)_3)_2(\mu\text{-}O)]$" (**Figure S10**). A bronze suspension of KC_8 (4.0 mg, 0.030 mmol, 3.0 equiv.) in THF-d_8 (0.15 mL) was added to reaction mixture and reacted for 20 minutes at -80 °C. The ^{1}H NMR spectrum of the reaction mixture showed only one set of resonances assigned to U^{III}/U^{III} "$[K(THF)_x]_2[(U(OAr)_3)_2(\mu\text{-}O)]$" (**Figure S10**). Multiple attempts to isolate single crystals for X-Ray diffraction studies proved unsuccessful due to the instability of U^{III}/U^{III} "$[K(THF)_x]_2[(U(OAr)_3)_2(\mu\text{-}O)]$" at -40 °C (**Figure S11**).

Reduction of $[(U(OAr)_3)_2(\mu\text{-}O)]$ (A) with 5.0 equiv. of KC_8 and 5.0 equiv. of LiI at -80 °C (NMR test reaction)

A pale yellow solution of complex **A** (9.6 mg, 0.0056 mmol, 1.0 equiv.) and lithium iodide (3.8 mg, 0.028 mmol, 5.0 equiv.) in THF-d_8 (0.25 mL) was added to a bronze suspension of KC_8 (3.9 mg, 0.029 mmol, 5.0 equiv.) in THF-d_8 (0.15 mL) at -80 °C. The reaction mixture was allowed to react for 20 minutes at -80 °C. The ^{1}H NMR spectrum of the reaction mixture showed one set of resonances assigned to U^{III}/U^{III} "$[Li(THF)_x]_2[(U(OAr)_3)_2(\mu\text{-}O)]$" (**Figure S12**). Multiple attempts to isolate single crystals for X-Ray diffraction studies proved unsuccessful due to the instability of U^{III}/U^{III} "$[Li(THF)_x]_2[(U(OAr)_3)_2(\mu\text{-}O)]$" at -40 °C (**Figure S13**). Only a few X-ray quality crystals of the decomposition products, U^{III}/U^{IV} $[Li(THF)_4][(U(OAr)_3)_2(\mu\text{-}O)]$(**2**) could be isolated from a concentrated THF/*n*-hexane solution after 1 day at -40 °C. Attempts to isolate complex **2** in higher yield were not successful preventing further characterization.

Reduction of [(U(OAr)$_3$)$_2$(μ-O)] (A) with 5.0 equiv. of KC$_8$, followed by the addition of 2.0 equiv. of 2.2.2-cryptand (NMR test reaction)

A pale yellow solution of complex **A** (8.4 mg, 0.0049 mmol, 1.0 equiv.) in THF-*d*$_8$ (0.25 mL) was added to a bronze suspension of KC$_8$ (3.6 mg, 0.027 mmol, 5.0 equiv.) in THF-*d*$_8$ (0.15 mL) at -80 °C. The reaction mixture was allowed to react for 20 minutes at -80 °C. The reaction mixture was filtered on a porosity 4 glass frit, yielding a dark red-brown solution. The ^{1}H NMR spectrum of the reaction mixture showed only one set of resonances assigned to U^{III}/U^{III} "[K(THF)$_x$]$_2$[(U(OAr)$_3$)$_2$(μ-O)]" (**Figure S14**). A 2.2.2-cryptand (3.8 mg, 0.010 mmol, 2.0 equiv.) solution in THF-*d*$_8$ (0.1 mL) was added to this dark red-brown solution at -80 °C. The ^{1}H NMR spectrum of the resulting solution showed the presence of complex **3**, with signals slightly shifted compared to U^{III}/U^{III} "[K(THF)$_x$]$_2$[(U(OAr)$_3$)$_2$(μ-O)]", indicating weak interactions between the K$^+$ ions and the ligands in "[K(THF)$_x$]$_2$[(U(OAr)$_3$)$_2$(μ-O)]" in THF solution (**Figure S14**).

Synthesis of [K(2.2.2-cryptand]$_2$[(U(OAr)$_3$)$_2$(μ-O)] (3)

A pale yellow solution of complex **A** (40.7 mg, 0.0236 mmol, 1.0 equiv.) and 2.2.2-cryptand (17.9 mg, 0.0475 mmol, 2.0 equiv.) in THF (2.0 mL) was added to a bronze suspension of KC$_8$ (16.8 mg, 0.0124 mmol, 5.0 equiv.) in THF (1.0 mL) at -80 °C. The reaction mixture was allowed to react for 20 minutes at -80 °C. The reaction mixture was filtered on a porosity 4 glass frit at -80 °C, yielding a dark red-brown solution. The filtrate was evaporated while maintaining the reaction flask at a -80 °C, and the resulting residue was washed with cold toluene (1.0 mL) and *n*-hexane (1.0 mL), affording analytically pure dark powder of complex **3** (48.2 mg, 80%). Complex **3** is only stable in the THF solution at -40 °C for 3 hours and fully decomposes after 6 hours at -40 °C (**Figure S17**). Multiple attempts to isolate single crystals for X-Ray diffraction studies proved unsuccessful due to the instability of **3** at -40 °C. ^{1}H NMR (400 MHz, THF-*d*$_8$, 193 K): δ 55.90 ppm, δ 9.48 ppm, δ -8.48 ppm, δ -27.17 ppm, δ -34.56 ppm (**Figure S16**). *Anal. Calcd.* for **2**, $C_{120}H_{198}K_2N_4O_{19}U_2$: C: 56.41; H: 7.81; N: 2.19. *Found*: C: 56.08; H: 7.60; N: 2.11.

Reactivity of 3 with azobenzene (PhNNPh)

Synthesis of [K(2.2.2-cryptand)(THF)][(U(OAr)$_3$)$_2$(μ-O)] · [K(2.2.2-cryptand)(THF)][PhNNPh], 4 · [K(2.2.2-cryptand)(THF)][PhNNPh]

An orange solution of PhNNPh (1.7 mg, 0.0093 mmol, 1.0 equiv.) in THF-*d*$_8$ (0.1 mL) was added to a dark red-brown solution of complex **3** (24.0 mg, 0.00939 mmol, 1.0 equiv.) in THF-*d*$_8$ (0.5 mL) at -80 °C, yielding a dark yellow-brown solution. The ^{1}H NMR spectrum of the reaction mixture showed the presence of new resonance assigned to **4.** The volatiles were removed under vacuum and the residue was dissolved in THF (0.5 mL). The resulting solution was stored at -40 °C overnight affording dark brown crystalline powder of complex **4** · [K(2.2.2-cryptand)(THF)][PhNNPh] (13.3 mg, 52%). X-ray quality crystals **4** · [K(2.2.2-cryptand)(THF)][PhNNPh] were obtained by cooling a concentrated THF solution to -40 °C. ^{1}H NMR (400 MHz, THF-*d*$_8$, 193 K): δ 66.63 ppm, δ 22.21 ppm, δ 18.90 ppm, δ 14.63 ppm, δ 13.27 ppm, δ -2.98 ppm, δ 59.02 ppm (**Figure S19**). The ^{1}H NMR spectrum is consistent with the *in-situ* formation of **4** from the mono-reduction of **A** with KC$_8$ and 2.2.2-cryptand (**Figure S18**). *Anal. Calcd.* for [K(2.2.2-cryptand)][(U(OAr)$_3$)$_2$(μ-O)] · [K(2.2.2-cryptand)][PhNNPh], $C_{132}H_{208}K_2N_6O_{19}U_2$: C: 57.92; H: 7.66; N: 3.07. *Found*: C: 57.47; H: 7.61; N: 2.89. The potassium bound THF was lost during the drying process.

Reduction of [(U(OAr)$_3$)$_2$(μ-O)] (A) with 1.0 equiv. of KC$_8$ and 1.0 equiv. of 2.2.2-cryptand at -80 °C (NMR test reaction)

A pale yellow solution of complex **A** (14.5 mg, 0.00841 mmol, 1.0 equiv.) and 2.2.2-cryptand (3.2 mg, 0.0085 mmol, 1.0 equiv.) in THF-*d*$_8$ (0.25 mL) was added to a bronze suspension of KC$_8$ (1.2 mg, 0.0089 mmol, 1.0 equiv.) in THF-*d*$_8$ (0.15 mL) at -80 °C. The reaction mixture was allowed to react for 20 minutes at -80 °C. The ^{1}H NMR spectrum of the reaction mixture showed one set of resonances assigned to U^{III}/U^{IV} "[K(2.2.2-cryptand)][(U(OAr)$_3$)$_2$(μ-O)]". Multiple attempts to isolate single crystals for X-Ray diffraction studies proved unsuccessful.

Reduction of [(U(OAr)$_3$)$_2$(μ-S)] (B) with 1.0-5.0 equiv. of KC$_8$ at -80 °C (NMR test reaction)

An orange solution of complex **B** (14.1 mg, 0.00810 mmol, 1.0 equiv.) in THF-*d*$_8$ (0.25 mL) was added to a bronze suspension of KC$_8$ (1.1 mg, 0.0081 mmol, 1.0 equiv.) in THF-*d*$_8$ (0.15 mL) at -80 °C. The reaction mixture was allowed to react for 20 minutes at -80 °C. The ^{1}H NMR spectrum of the reaction mixture showed one set of resonances assigned to U^{III}/U^{IV} "[K(THF)$_x$][(U(OAr)$_3$)$_2$(μ-S)]" (**Figure S20**). A bronze suspension of KC$_8$ (1.1 mg, 0.0081 mmol, 1.0 equiv.) in THF-*d*$_8$ (0.15 mL) was added to reaction mixture and reacted for 20 minutes at -80 °C. The ^{1}H NMR spectrum of the reaction mixture showed two sets of resonances assigned to U^{III}/U^{IV} "[K(THF)$_x$][(U(OAr)$_3$)$_2$(μ-S)]" and U^{III}/U^{III} "[K(THF)$_x$]$_2$[(U(OAr)$_3$)$_2$(μ-S)]" (**Figure S20**). A bronze suspension of KC$_8$ (3.3 mg, 0.024 mmol, 3.0 equiv.) in THF-*d*$_8$ (0.15 mL) was added to reaction mixture and reacted for 20 minutes at -80 °C. The ^{1}H NMR spectrum of the reaction mixture showed only one set of resonances assigned to U^{III}/U^{III} "[K(THF)$_x$]$_2$[(U(OAr)$_3$)$_2$(μ-S)]" (**Figure S20**). Multiple attempts to isolate single crystals for X-Ray diffraction studies proved unsuccessful due to the instability of U^{III}/U^{III} "[K(THF)$_x$]$_2$[(U(OAr)$_3$)$_2$(μ-S)]" at -40 °C (**Figure S21**).

^{1}H NMR spectroscopy studies of rearrangement of "[K(THF)$_x$]$_2$[(U(OAr)$_3$)$_2$(μ-S)]" in THF at -40 °C (NMR test reaction)

An orange solution of complex **B** (15.6 mg, 0.00897 mmol, 1.0 equiv.) in THF-*d*$_8$ (0.25 mL) was added to a bronze suspension of KC$_8$ (6.2 mg, 0.046 mmol, 5.0 equiv.) in THF-*d*$_8$ (0.15 mL) at -80 °C. The reaction mixture was allowed to react for 20 minutes at -80 °C. The reaction mixture was filtered on a porosity 4 glass frit at -80 °C, yielding a dark red-brown solution. The ^{1}H NMR spectroscopy studies of the reaction mixture showed that "[K(THF)$_x$]$_2$[(U(OAr)$_3$)$_2$(μ-S)]" was completely consumed after 3 weeks at -40 °C, resulting in new resonances assigned to [KU(OAr)$_4$], KOAr and new species (**Figure S21**). Only a few X-ray quality crystals of [(K(THF)$_4$)$_2$(U(OAr)$_2$)$_2$(μ-S)$_2$] (**5**) could be isolated from a concentrated THF/*n*-hexane solution at -40 °C. Attempts to isolate complex **5** in higher yield were not successful due to the similar solubility of **5**, [KU(OAr)$_4$] and KOAr, preventing further characterization. The formation of [KU(OAr)$_4$] was confirmed by the *in-situ* formation of [KU(OAr)$_4$] from the mono-reduction of [U(OAr)$_4$] with KC$_8$ (**Figure S21**).

Reduction of [U(OAr)$_4$] with 1.2 equiv. of KC$_8$ (NMR test reaction)

A green solution of complex **A** (10.6 mg, 0.0100 mmol, 1.0 equiv.) in THF-*d*$_8$ (0.25 mL) was added to a bronze suspension of KC$_8$ (1.6 mg, 0.012 mmol, 1.2 equiv.) in THF-*d*$_8$ (0.15 mL) at -80 °C, resulting in a dark red-brown solution. The reaction mixture was allowed to react for 20 minutes at -80 °C. The ^{1}H NMR spectrum of the reaction mixture showed one set of resonances assigned to [KU(OAr)$_4$] (**Figure S21**).

Reduction of [(U(OAr)$_3$)$_2$(μ-S)] (B) with 5.0 equiv. of KC$_8$, followed by the addition of 2.0 equiv. of 2.2.2-cryptand (NMR test reaction)

An orange solution of complex **B** (8.5 mg, 0.0049 mmol, 1.0 equiv.) in THF-d_8 (0.25 mL) was added to a bronze suspension of KC$_8$ (3.5 mg, 0.026 mmol, 5.0 equiv.) in THF-d_8 (0.15 mL) at -80 °C. The reaction mixture was allowed to react for 20 minutes at -80 °C. The reaction mixture was filtered on a porosity 4 glass frit, yielding a dark red-brown solution. The ^{1}H NMR spectrum of the reaction mixture showed only one set of resonances assigned to U^{III}/U^{III} "[K(THF)$_x$]$_2$[(U(OAr)$_3$)$_2$(μ-S)]" (**Figure S22**). A 2.2.2-cryptand (3.7 mg, 0.0098 mmol, 2.0 equiv.) solution in THF-d_8 (0.1 mL) was added to this dark red-brown solution at -80 °C. The ^{1}H NMR spectrum of the resulting solution showed the presence of complex **6**, with signals slightly shifted compared to U^{III}/U^{III} "[K(THF)$_x$]$_2$[(U(OAr)$_3$)$_2$(μ-S)]", indicating weak interaction between the K$^+$ ions and the ligands in "[K(THF)$_x$]$_2$[(U(OAr)$_3$)$_2$(μ-S)]" in THF solution (**Figure S22**).

Synthesis of [K(2.2.2-cryptand]$_2$[(U(OAr)$_3$)$_2$(μ-S)] (6)

An orange solution of complex **B** (51.7 mg, 0.0297 mmol, 1.0 equiv.) and 2.2.2-cryptand (22.4 mg, 0.0594 mmol, 2.0 equiv.) in THF (2.0 mL) was added to a bronze suspension of KC$_8$ (20.9 mg, 0.154 mmol, 5.0 equiv.) in THF (1.0 mL) at -80 °C. The reaction mixture was allowed to react for 20 minutes at -80 °C. The reaction mixture was filtered on a porosity 4 glass frit at -80 °C, yielding a dark red-brown solution. The filtrate was evaporated while maintaining the reaction flask at a -80 °C, and the resulting residue was dissolved in a mixture of cold THF (1.0 mL) and *n*-hexane (1.0 mL). The resulting solution was stored at -40 °C overnight affording dark powder of complex **6** (73.1 mg, 96%). X-ray quality crystals **6** were obtained by cooling a concentrated THF/*n*-hexane solution to -40 °C. Complex **6** is stable in the THF solution for up to 1 week at -40 °C (**Figure S25**). ^{1}H NMR (400 MHz, THF-d_8, 193 K): δ 12.06 ppm, δ 10.88 ppm, δ 4.24 ppm, δ 4.07 ppm, δ 3.91 ppm, δ 2.53 ppm, δ 1.38 ppm (**Figure S24**). *Anal. Calcd.* for **6**, $C_{120}H_{198}K_2N_4O_{19}U_2$: C: 56.06; H: 7.76; N: 2.18; S: 1.25. *Found*: C: 55.76; H: 7.55; N: 2.27; S: 1.34.

Reactivity of 6 with azobenzene (PhNNPh) (NMR test reaction)

An orange solution of PhNNPh (1.0 mg, 0.0055 mmol, 1.0 equiv.) in THF-d_8 (0.15 mL) was added to a dark red-brown solution of complex **6** (12.9 mg, 0.00502 mmol, 1.0 equiv.) in THF-d_8 (0.25 mL) at -80 °C, yielding a dark yellow-brown solution. The ^{1}H NMR spectrum of the reaction mixture showed the presence of two sets of resonance assigned to unreacted **6** and mono-reduced "[K(2.2.2-cryptand)][(U(OAr)$_3$)$_2$(μ-S)]" (**Figure S26**). The full consumption of **6** and the formation of "[K(2.2.2-cryptand)][(U(OAr)$_3$)$_2$(μ-S)]", KOAr ligand and unknown species were observed when the reaction mixture was warmed up to -40 °C after 1 hour (**Figure S26**). The major compound in the reaction mixture is consistent with the *in-situ* formation of mono-reduced "[K(2.2.2-cryptand)][(U(OAr)$_3$)$_2$(μ-S)]" from the mono-reduction of **B** with KC$_8$ and 2.2.2-cryptand (**Figure S26**).

Reduction of [(U(OAr)$_3$)$_2$(μ-S)] (B) with 1.0 equiv. of KC$_8$ and 1.0 equiv. of 2.2.2-cryptand at -80 °C (NMR test reaction)

An orange solution of complex **B** (11.8 mg, 0.00678 mmol, 1.0 equiv.) and 2.2.2-cryptand (2.7 mg, 0.0072 mmol, 1.0 equiv.) in THF-d_8 (0.25 mL) was added to a bronze suspension of KC$_8$ (1.0 mg, 0.0074 mmol, 1.0 equiv.) in THF-d_8 (0.15 mL) at -80 °C. The reaction mixture was allowed to react for 20 minutes at -80 °C. The ^{1}H NMR spectrum of the reaction mixture showed one set of resonances assigned to U^{III}/U^{IV} "[K(2.2.2-cryptand)][(U(OAr)$_3$)$_2$(μ-S)]" (**Figure S26**).

S3. NMR Spectroscopic Data

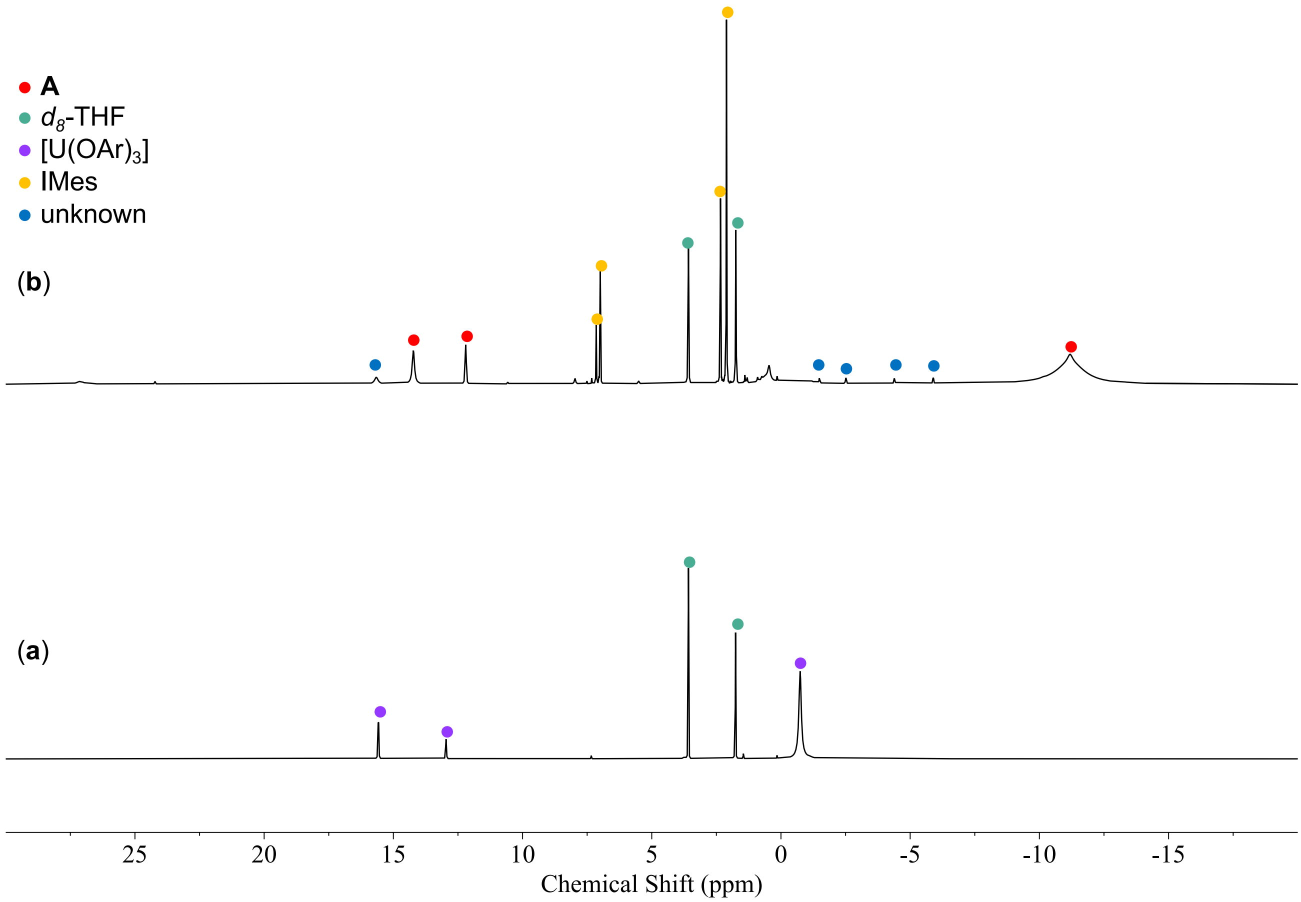

Figure S1. ^{1}H NMR spectra (400 MHz, THF-d_8, 298 K) of the reaction mixture obtained after addition of 0.5 equiv. of IMesN$_2$O to [U(OAr)$_3$] at -80 °C (a) before (b) [U(OAr)$_3$] and 0.5 equiv. of IMesN$_2$O after 5 h, resulted in complex **A**.

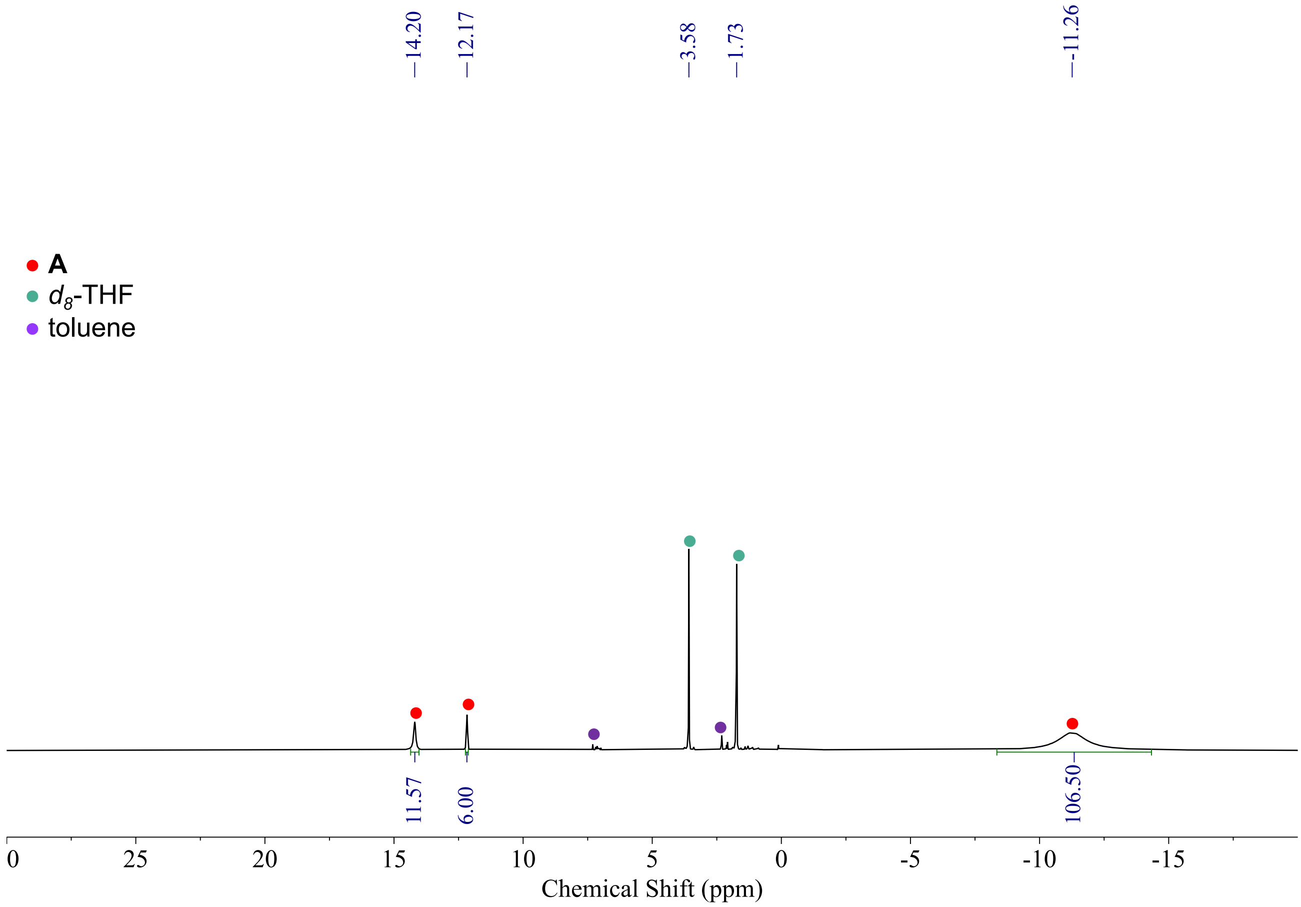

Figure S2. ^1H NMR spectrum (400 MHz, THF-d_8, 298 K) of isolated **A**.

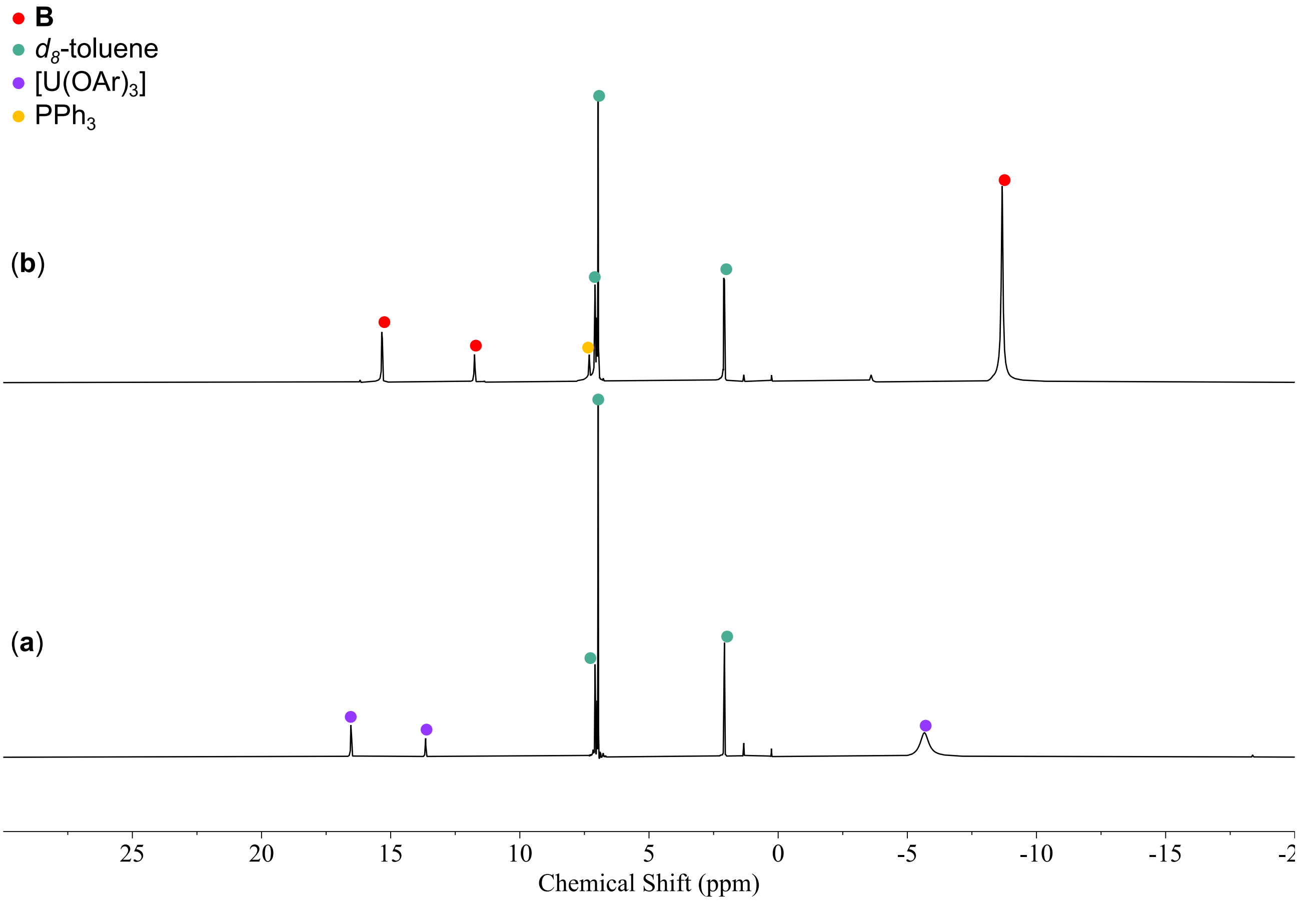

Figure S3. ^{1}H NMR spectra (400 MHz, THF-d_8, 298 K) of the reaction mixture obtained after addition of 0.5 equiv. of Ph_3PS to [U(OAr)$_3$] at room temperature (a) before (b) [U(OAr)$_3$] and 0.5 equiv. of Ph_3PS after 3 h, resulted in complex **B**.

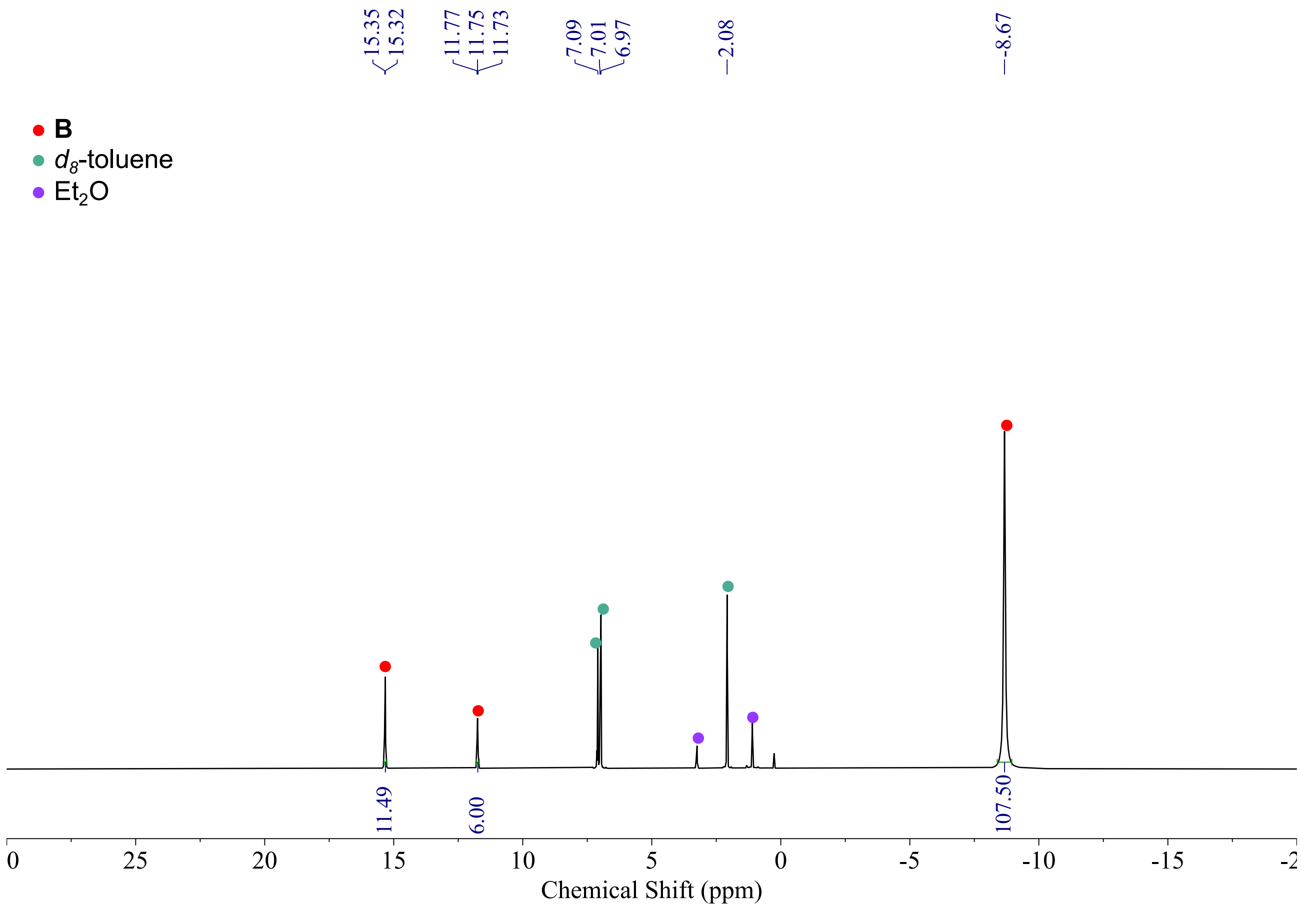

Figure S4. ^{1}H NMR spectrum (400 MHz, toluene-*d*8, 298 K) of isolated **B**.

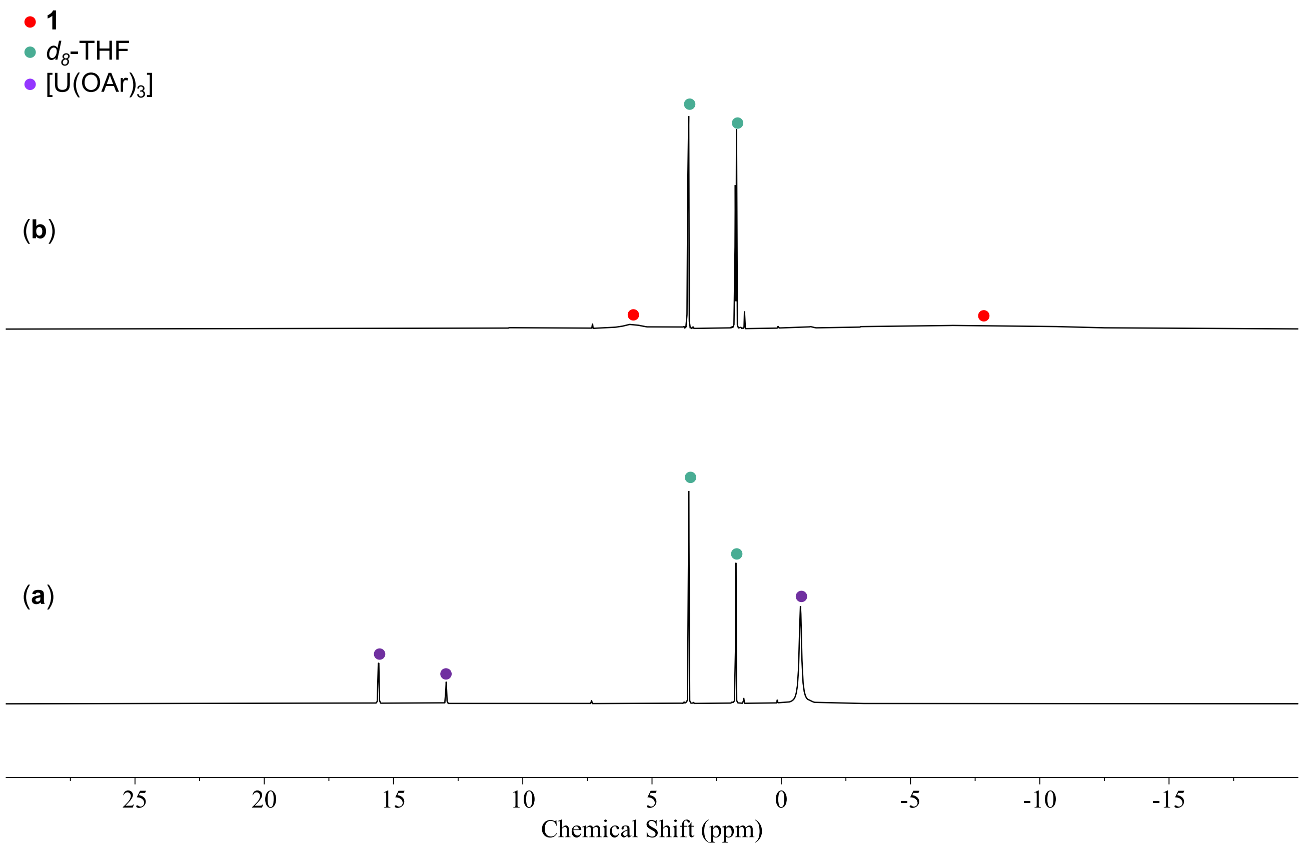

Figure S5. ^{1}H NMR spectra (400 MHz, THF-d_8, 298 K) of the reaction mixture obtained after addition of 0.5 equiv. of CsN_3 to [U(OAr)$_3$] at -40 °C (a) before (b) [U(OAr)$_3$] and 0.5 equiv. of CsN_3 after 4 days, resulted in complex **1**.

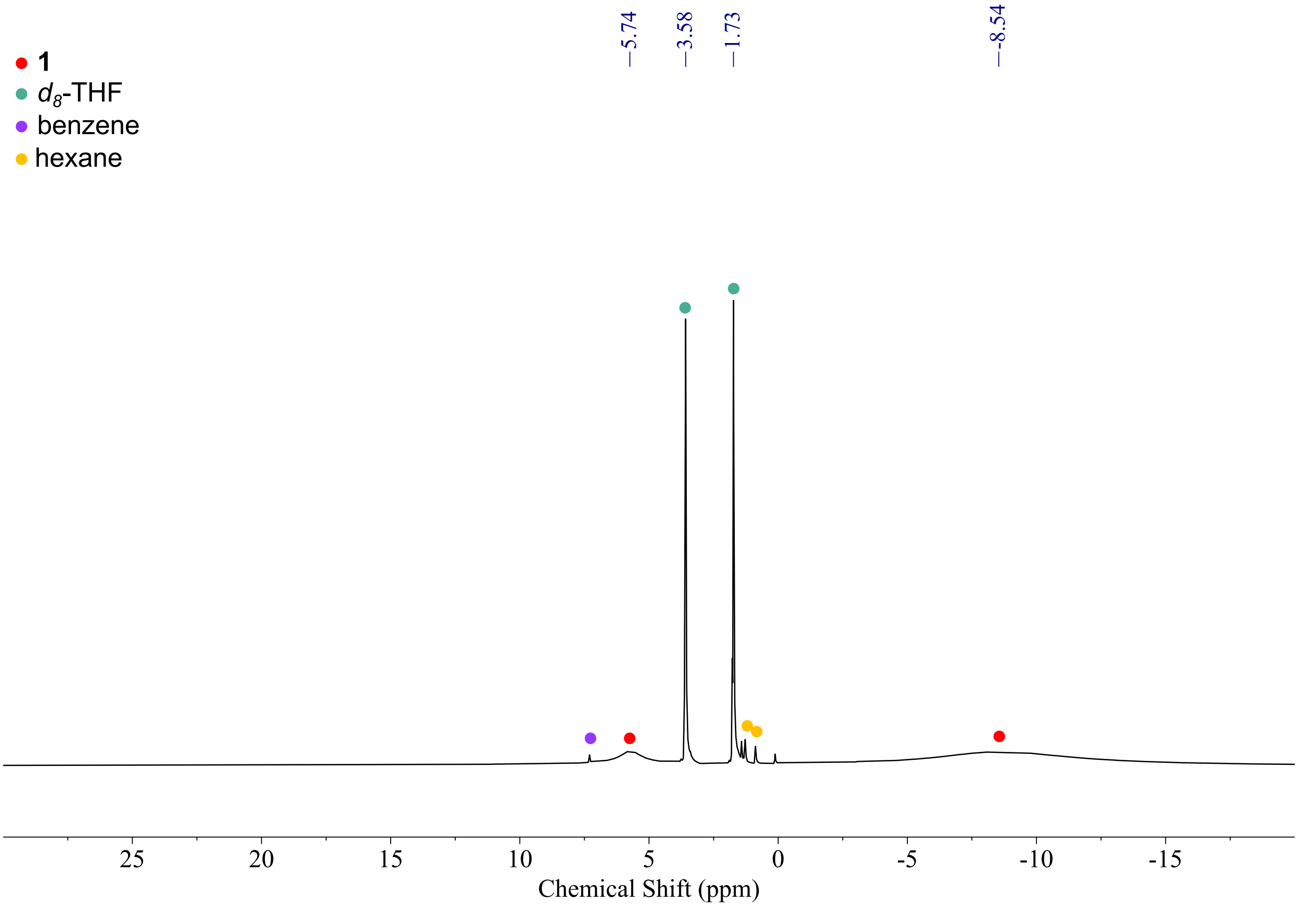

Figure S6. ^{1}H NMR spectrum (400 MHz, THF-d_8, 298 K) of isolated **1**.

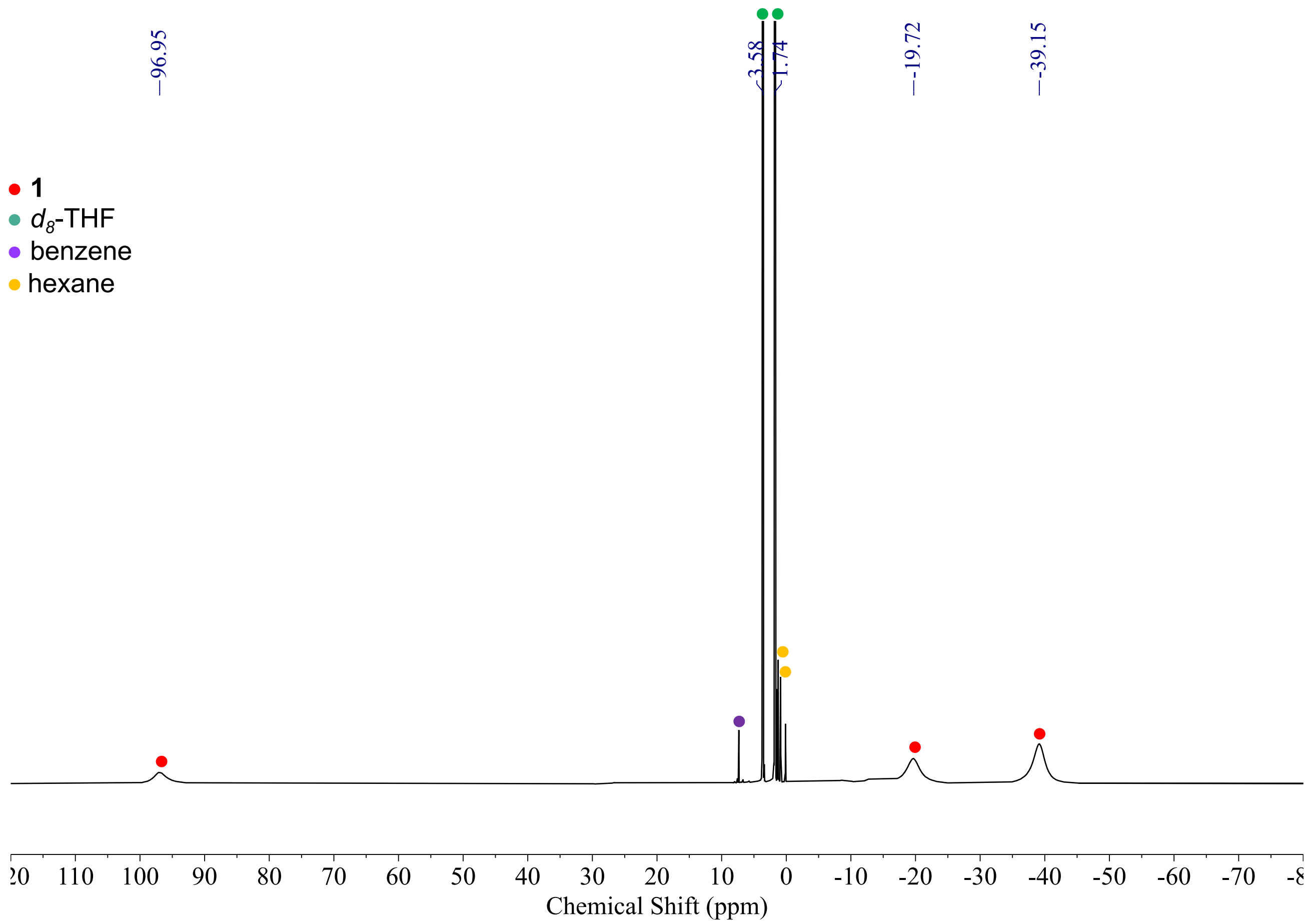

Figure S7. ^{1}H NMR spectrum (400 MHz, THF-d_8, 233 K) of isolated **1**.

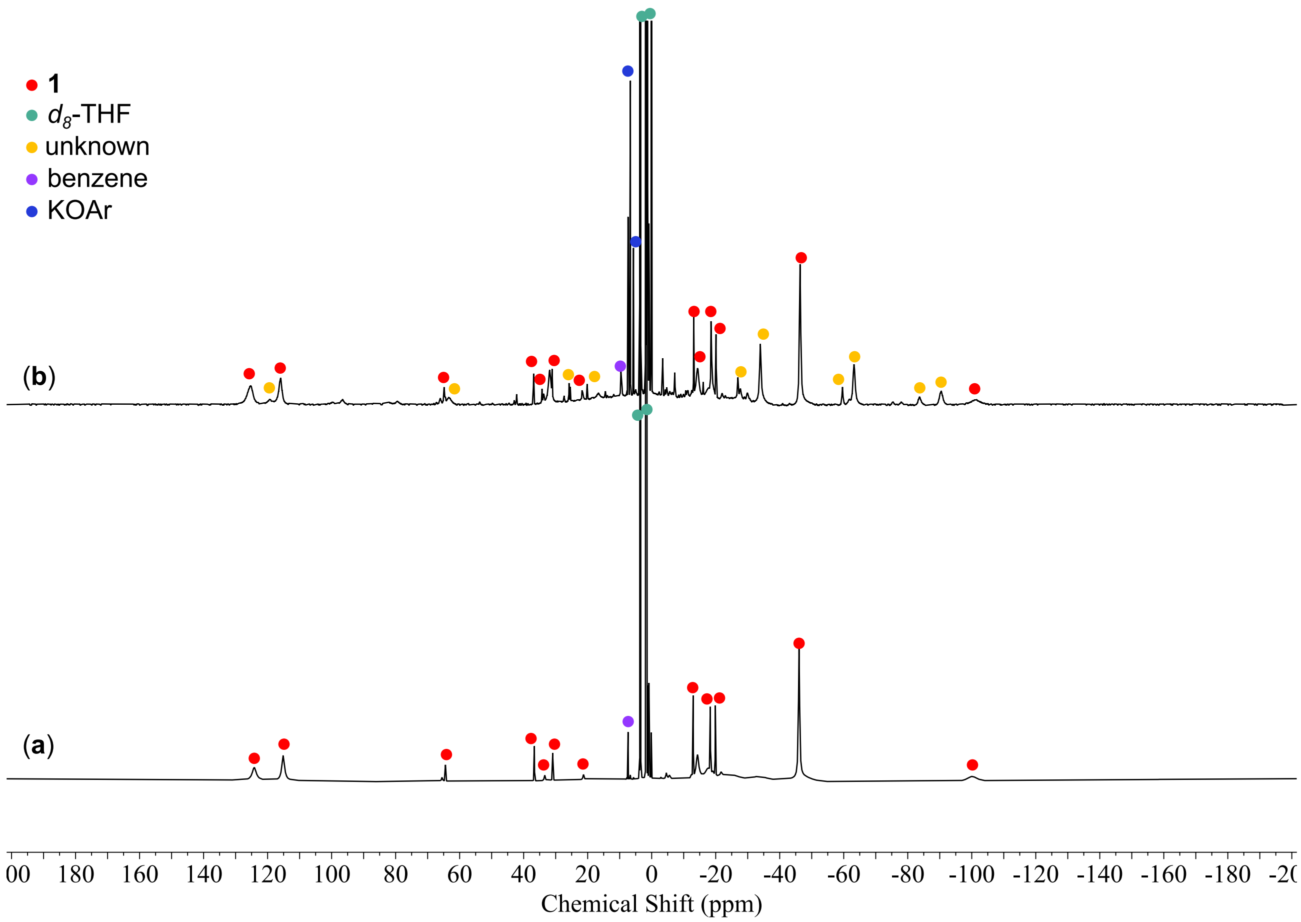

Figure S8. ^{1}H NMR spectra (400 MHz, THF-d_8, 193 K) of the reaction mixture obtained after addition of 5.0 equiv. of KC_8 to **1** at -80 °C (a) before (b) **1** and 5.0 equiv. of KC_8 after 6 h.

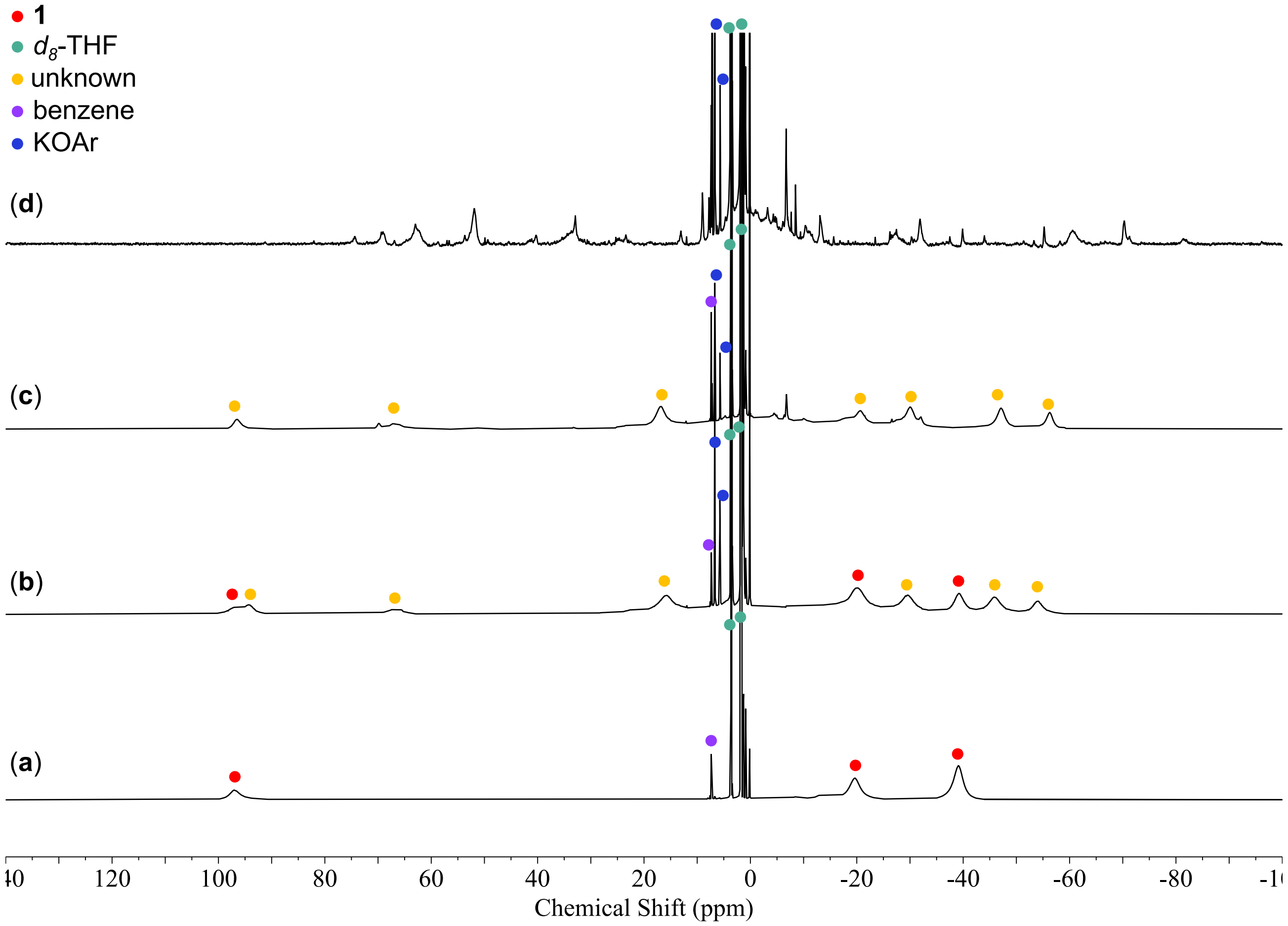

Figure S9. ^{1}H NMR spectra (400 MHz, THF-d_8, 233 K) of the reaction mixture obtained after addition of 1.0-3.0 equiv. of KC_8 to **1** at -40 °C (a) before (b) **1** and 1.0 equiv. of KC_8 after 30 mins (c) **1** and 2.0 equiv. of KC_8 after 30 mins (d) **1** and 3.0 equiv. of KC_8 after 30 mins.

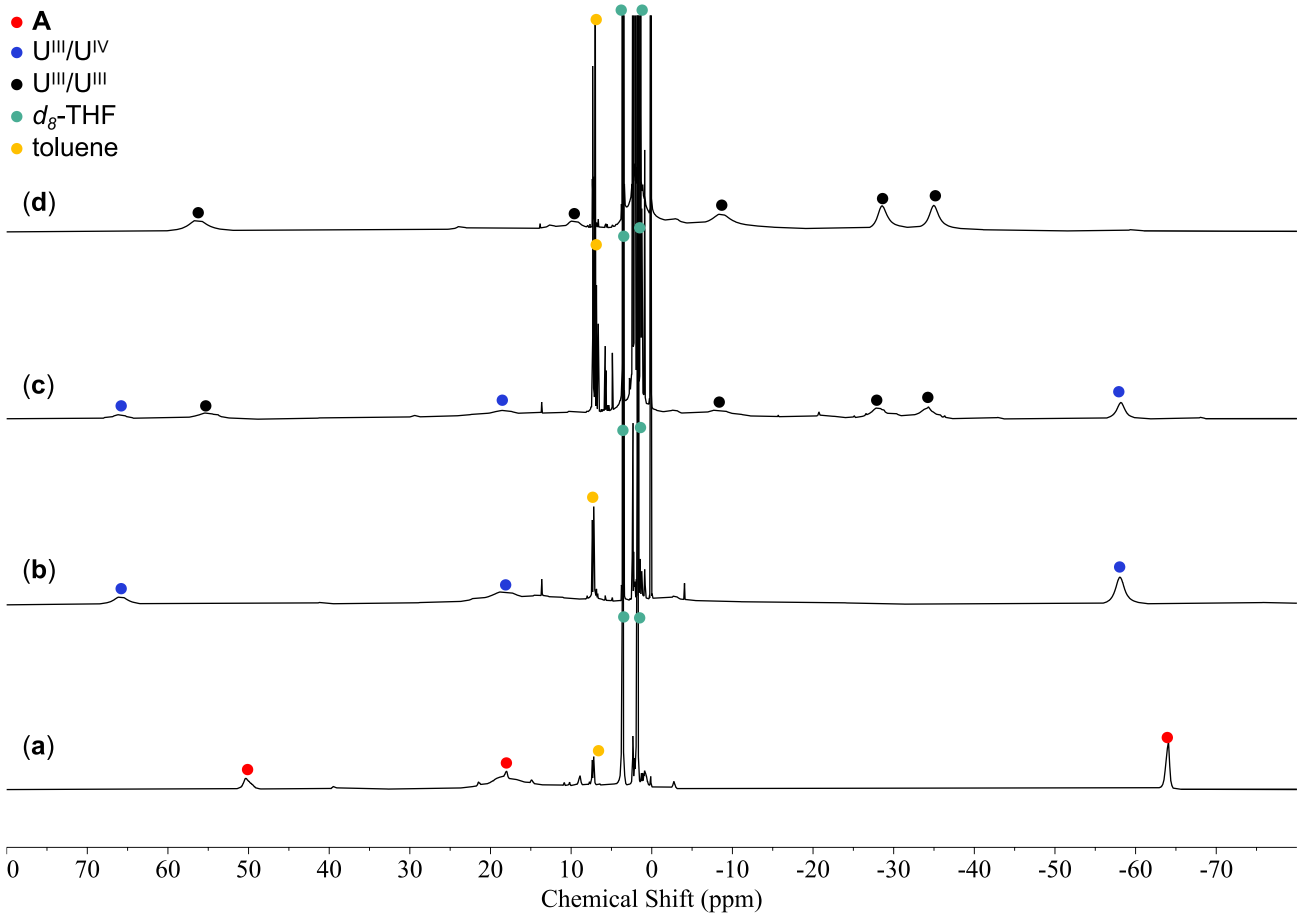

Figure S10. ^{1}H NMR spectra (400 MHz, THF-d_8, 193 K) of the reaction mixture obtained after addition of 1.0-5.0 equiv. of KC_8 to **A** at -80 °C (a) before (b) **A** and 1.0 equiv. of KC_8 after 20 mins (c) **1** and 2.0 equiv. of KC_8 after 20 mins (d) **1** and 5.0 equiv. of KC_8 after 20 mins (U^{III}/U^{IV} = "[K(THF)$_x$][(U(OAr)$_3$)$_2$(μ-O)]", U^{III}/U^{III} = "[K(THF)$_x$]$_2$[(U(OAr)$_3$)$_2$(μ-O)]").

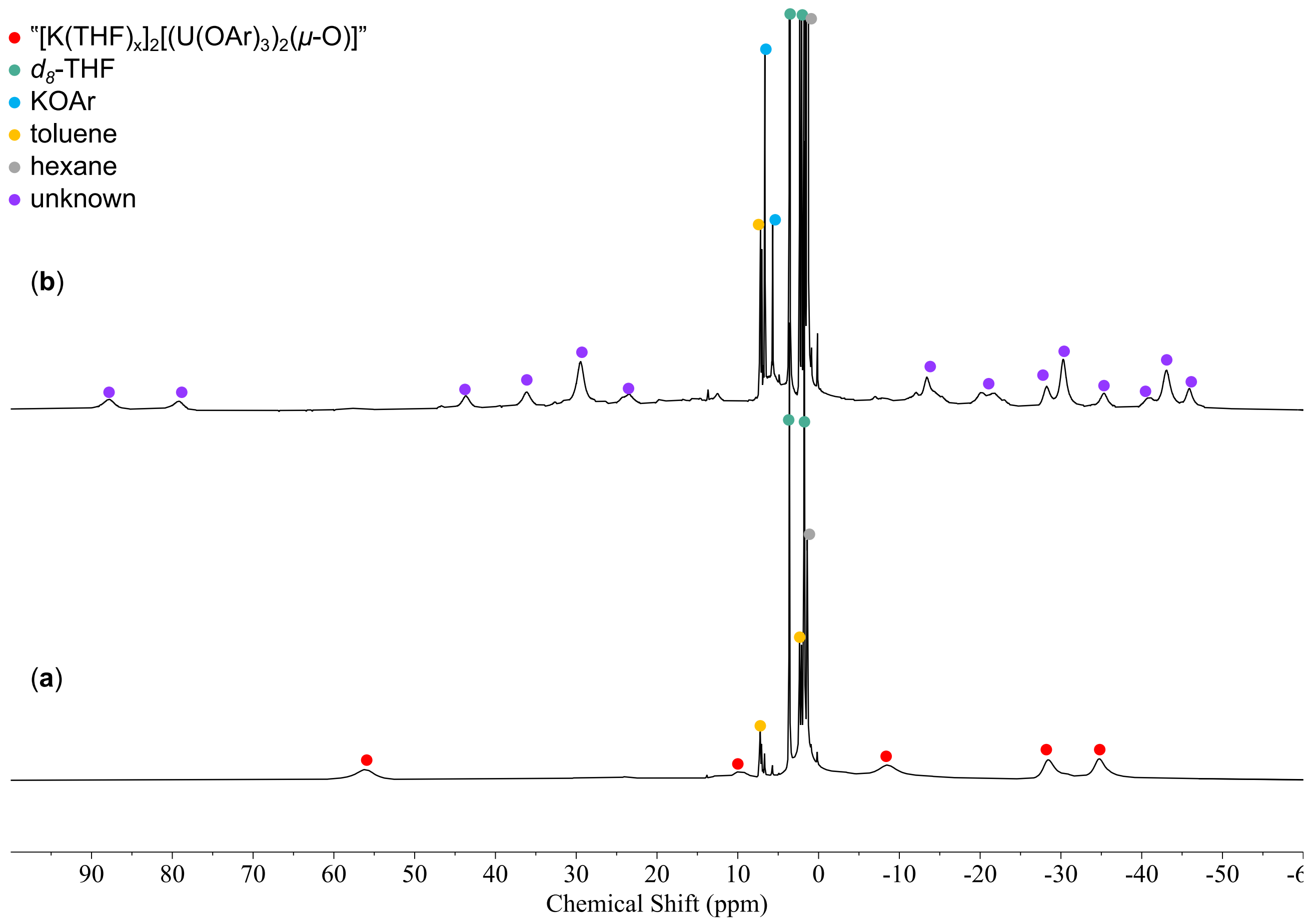

Figure S11. ^{1}H NMR spectra (400 MHz, THF-*d*$_8$, 193 K) of the reaction mixture obtained after addition of 5.0 equiv. of KC_8 to **A** at -80 °C (a) **A** and 5.0 equiv. of KC_8 after 20 mins (b) **A** and 5.0 equiv. of KC_8 after being warmed up to -40 °C for 10 mins, and then cooled back to -80 °C, showing decomposition.

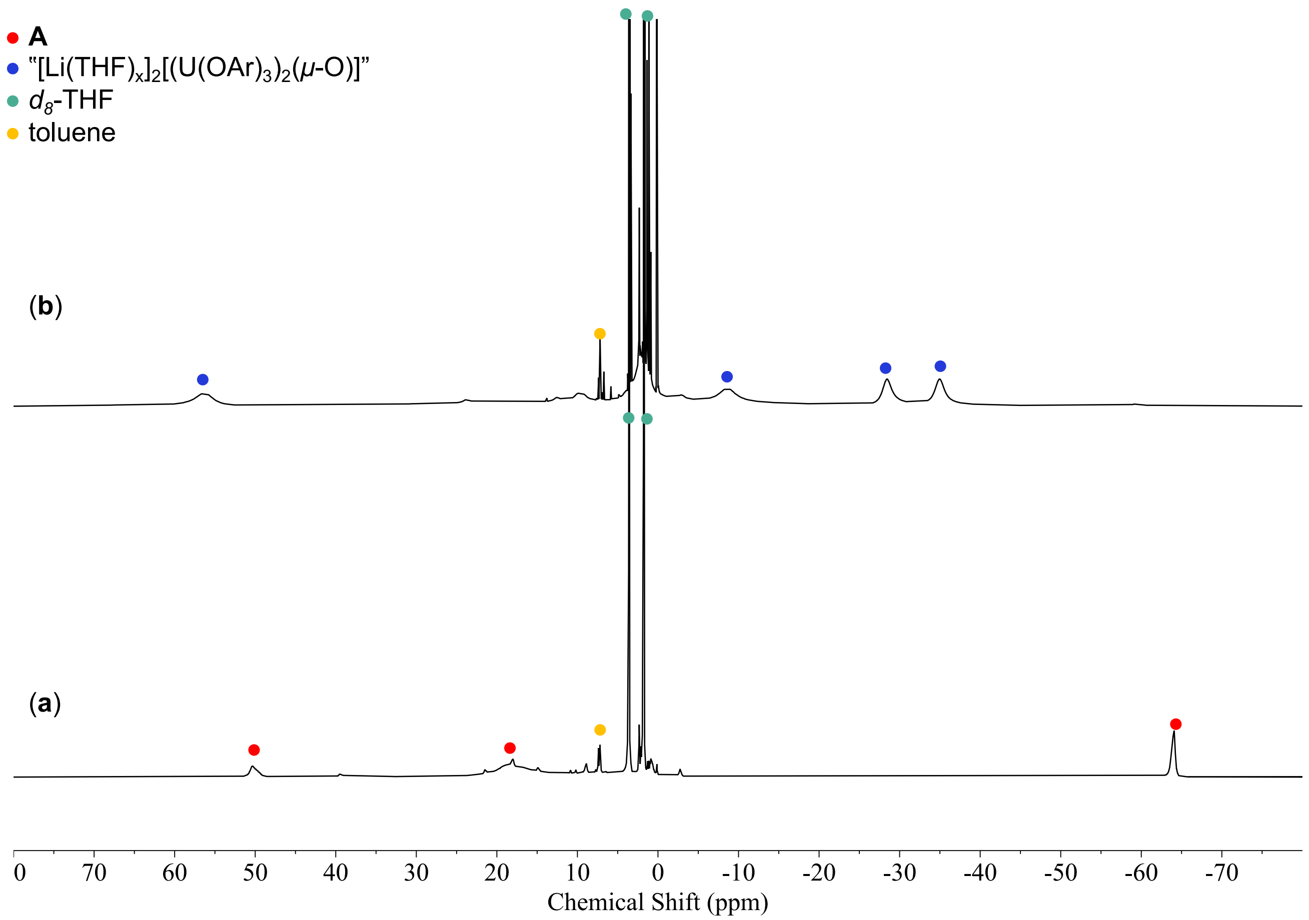

Figure S12. ^{1}H NMR spectra (400 MHz, THF-d_8, 193 K) of the reaction mixture obtained after addition of 5.0 equiv. of KC_8 and 5.0 equiv. of LiI to **A** at -80 °C (a) before (b) **A** and 5.0 equiv. of KC_8 and 5.0 equiv. of LiI after 20 mins.

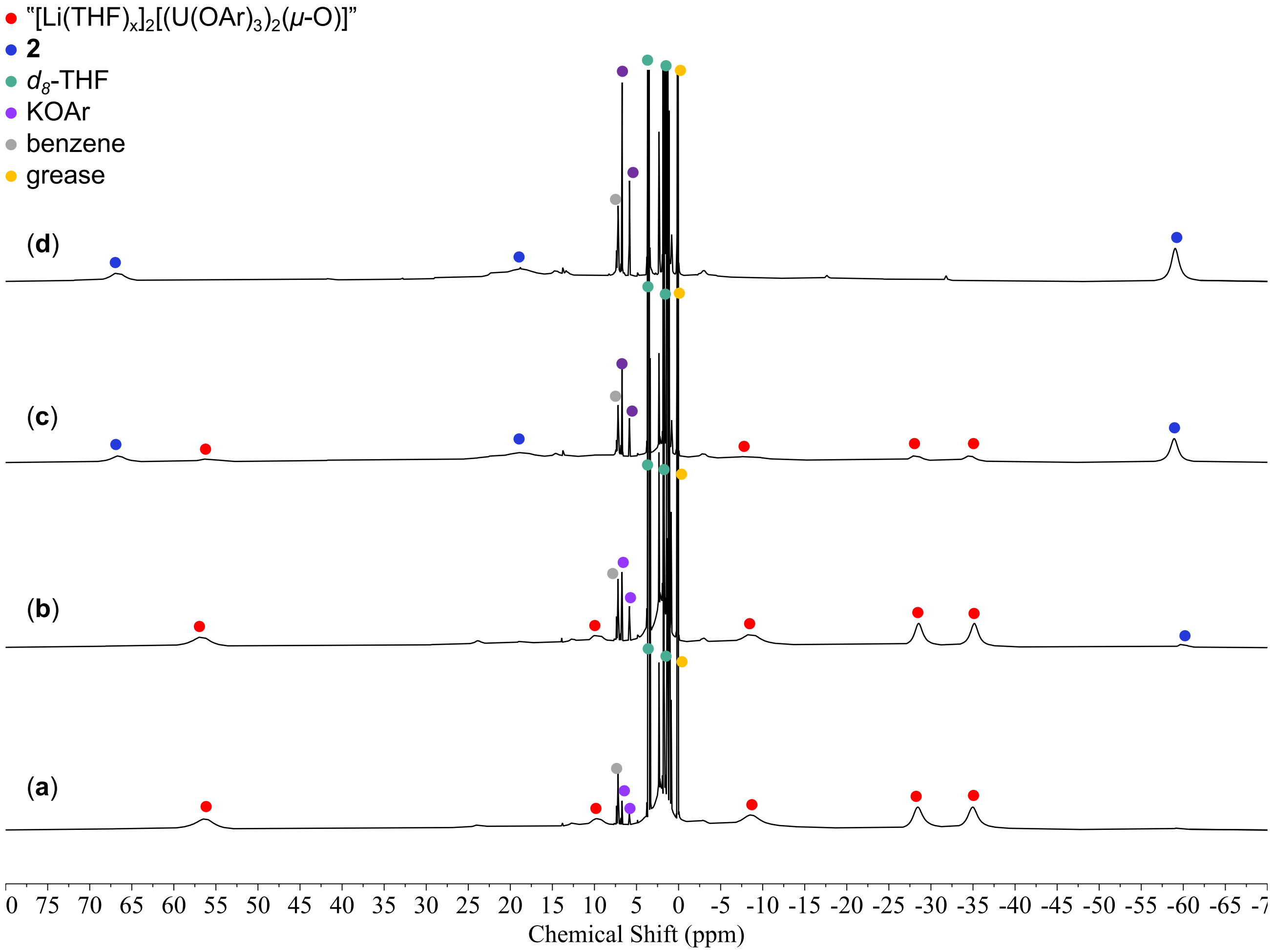

Figure S13. ^{1}H NMR spectra (400 MHz, THF-d_8, 193 K) of the reaction mixture obtained after addition of 5.0 equiv. of KC_8 and 5.0 equiv. of LiI to **A** at -80 °C (a) **A** and 5.0 equiv. of KC_8 and 5.0 equiv. of LiI after 20 mins (b) **A** and 5.0 equiv. of KC_8 and 5.0 equiv. of LiI after being warmed up to -40 °C for 2 h (c) **A** and 5.0 equiv. of KC_8 and 5.0 equiv. of LiI after being warmed up to -40 °C for 4 h (d) **A** and 5.0 equiv. of KC_8 and 5.0 equiv. of LiI after being warmed up to -40 °C for 6 h.

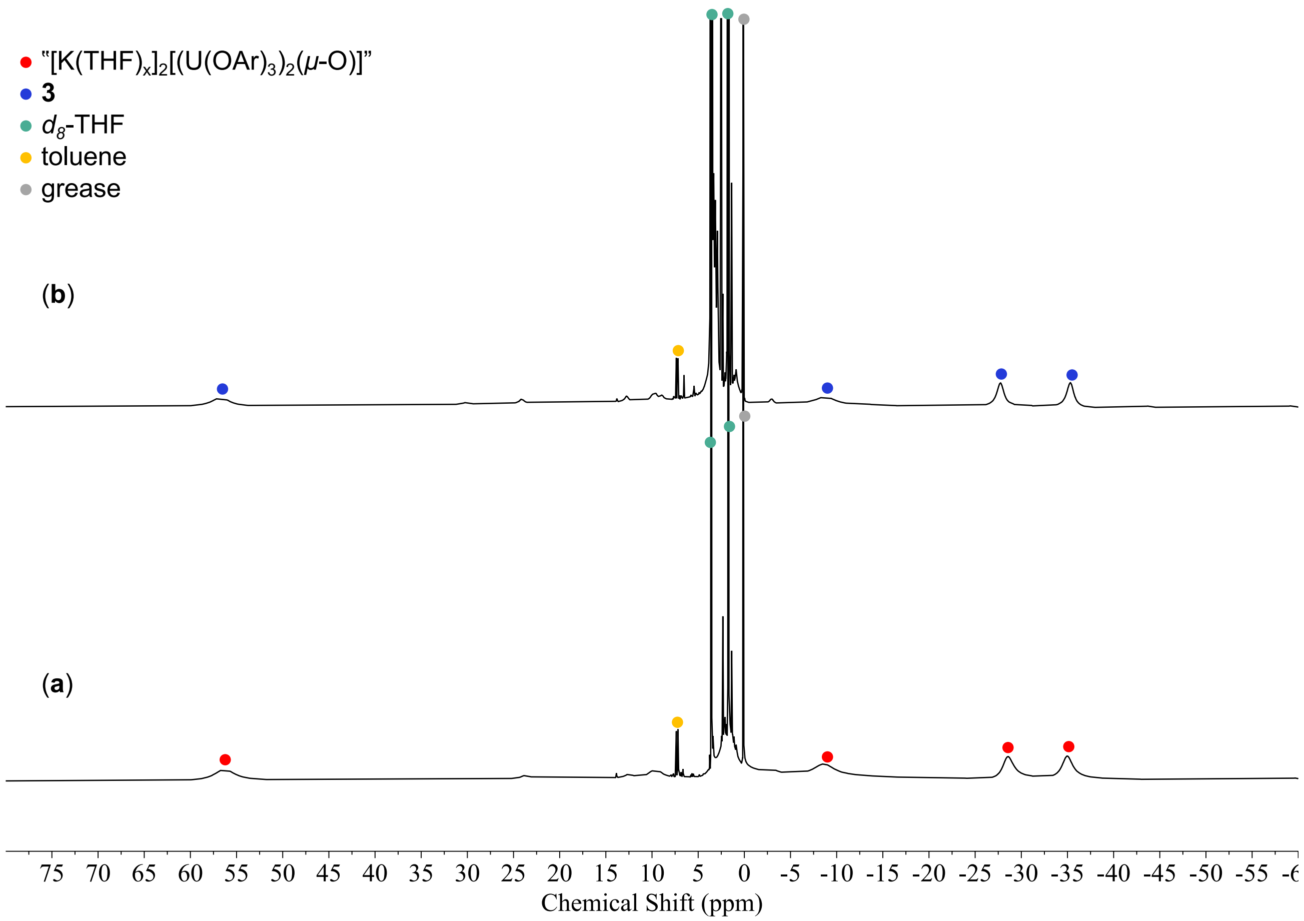

Figure S14. ^{1}H NMR spectra (400 MHz, THF-d_8, 193 K) of the reaction mixture obtained after addition of 2.0 equiv. of 2.2.2-cryptand to"$[K(THF)_x]_2[(U(OAr)_3)_2(\mu\text{-}O)]$" at -80 °C (a) before (b) "$[K(THF)_x]_2[(U(OAr)_3)_2(\mu\text{-}O)]$" and 2.0 equiv. of 2.2.2-cryptand immediately, showing the presence of complex **3**.

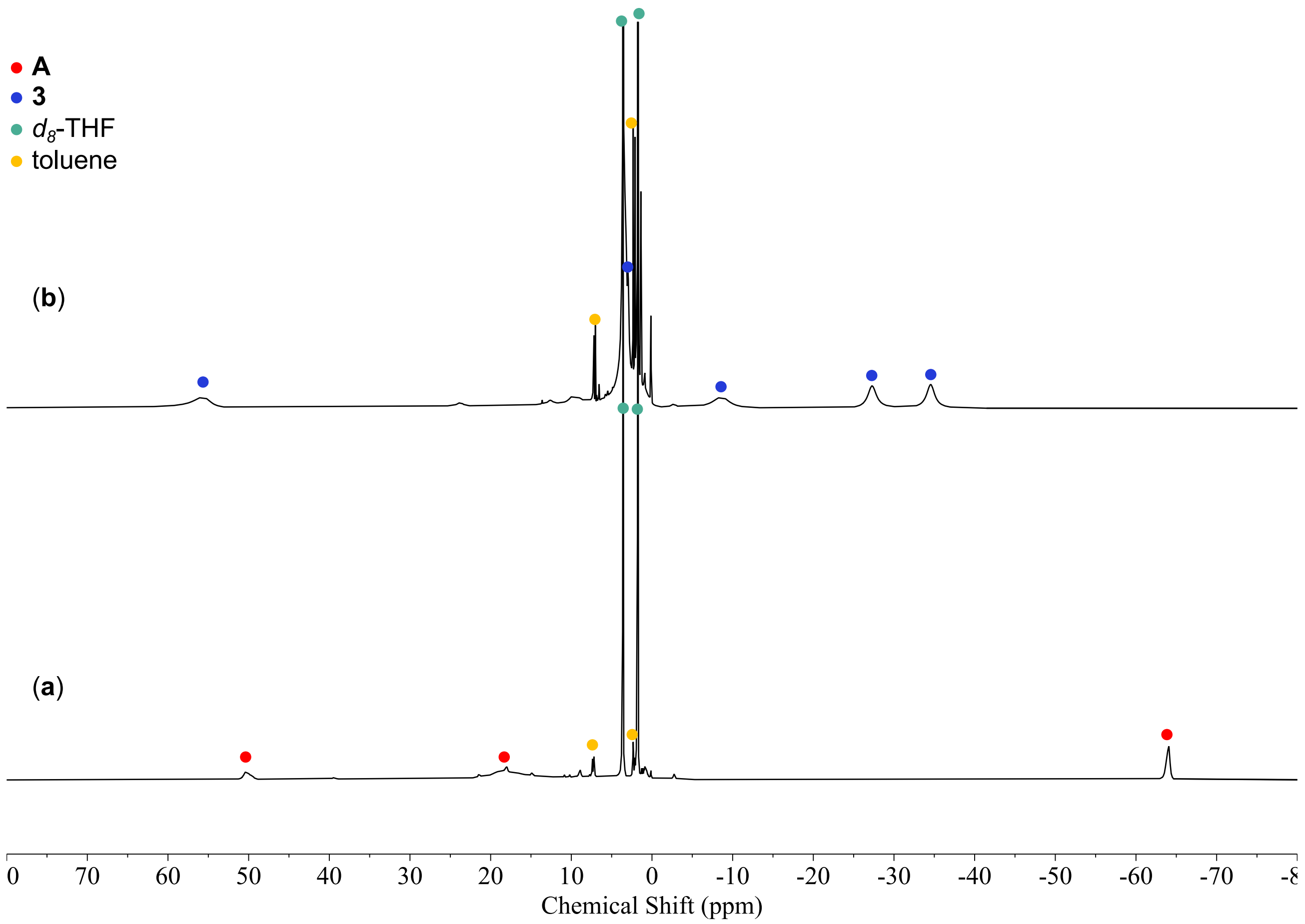

Figure S15. ^{1}H NMR spectra (400 MHz, THF-d_8, 193 K) of the reaction mixture obtained after addition of 5.0 equiv. of KC_8 and 2.0 equiv. of 2.2.2-cryptand to **A** at -80 °C (a) before (b) **A** and 5.0 equiv. of KC_8 and 2.0 equiv. of 2.2.2-cryptand after 20 mins, showing the presence of complex **3**.

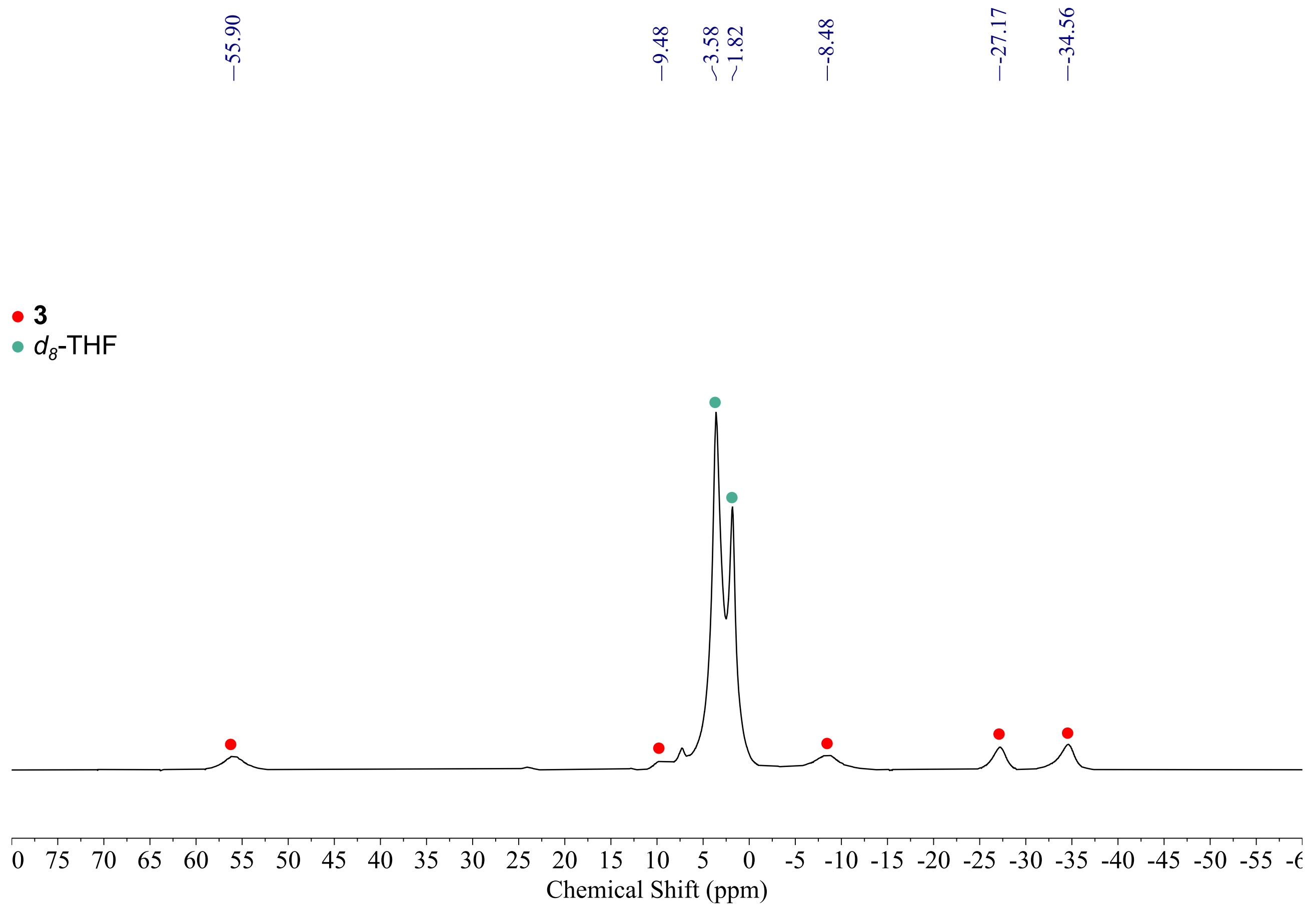

Figure S16. ^{1}H NMR spectrum (400 MHz, THF-d_8, 193 K) of isolated **3**.

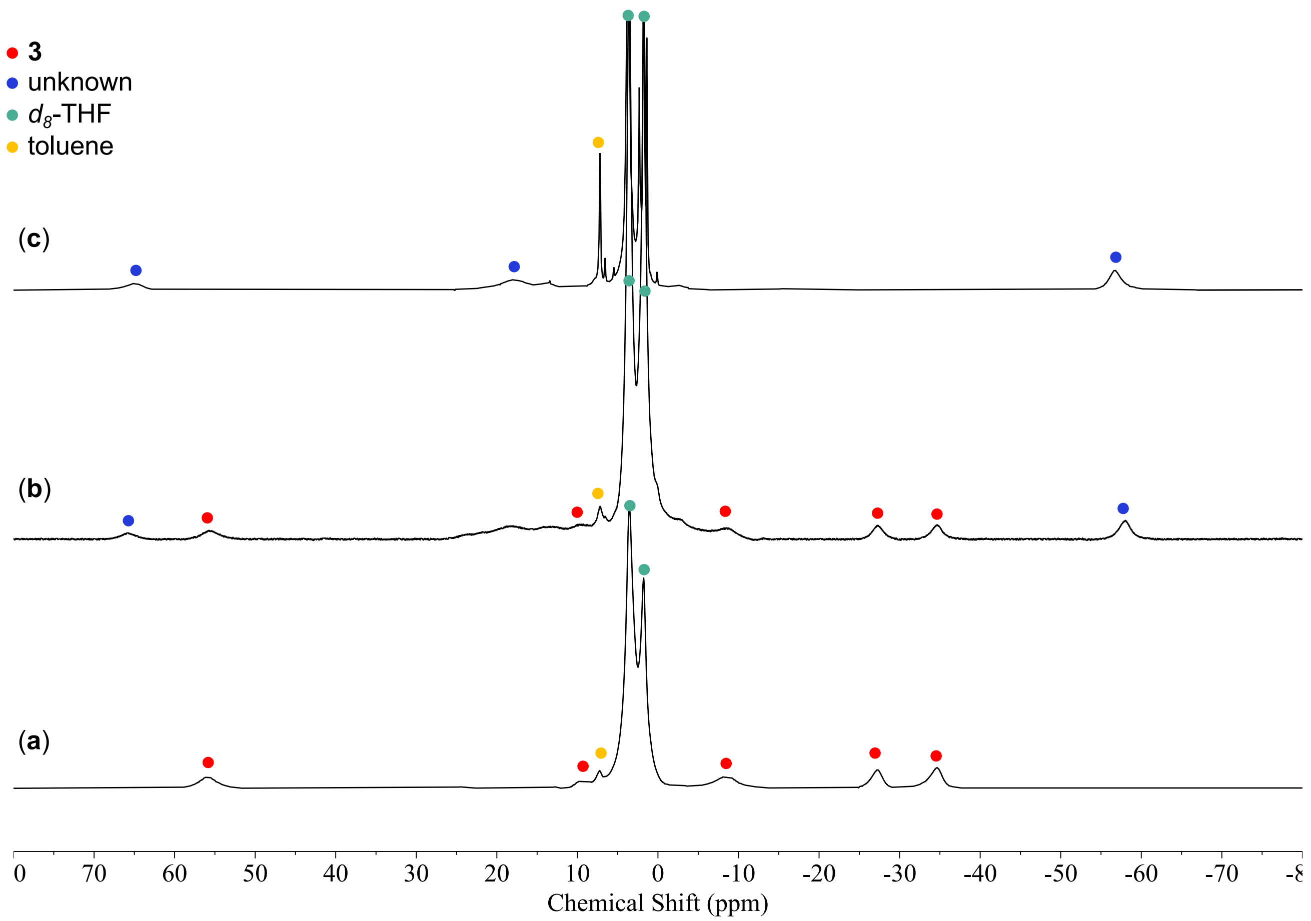

Figure S17. ^{1}H NMR spectra (400 MHz, THF-d_8, 193 K) of the isolated **3** at -80 °C (a) before (b) **3** after being warmed up to -40 °C for 3 h (c) **3** after being warmed up to -40 °C for 6 h.

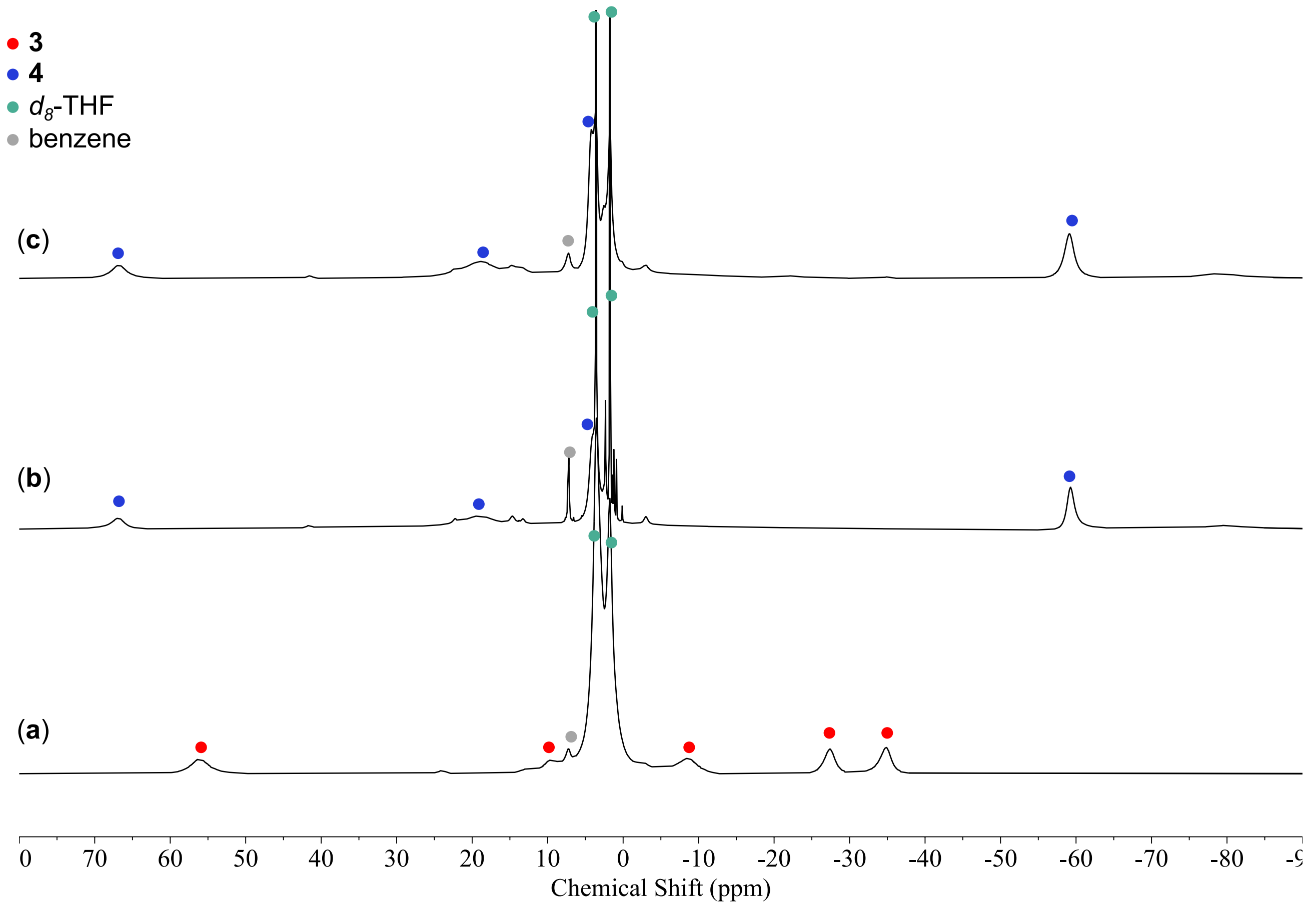

Figure S18. ^{1}H NMR spectra (400 MHz, THF-d_8, 193 K) of the reaction mixture obtained after addition of 1.0 equiv. of PhNNPh to **3** at -80 °C (a) before (b) **3** and 1.0 equiv. of PhNNPh immediately (c) **A** and 1.0 equiv. of KC_8 and 1.0 equiv. of 2.2.2-cryptand after 20 mins.

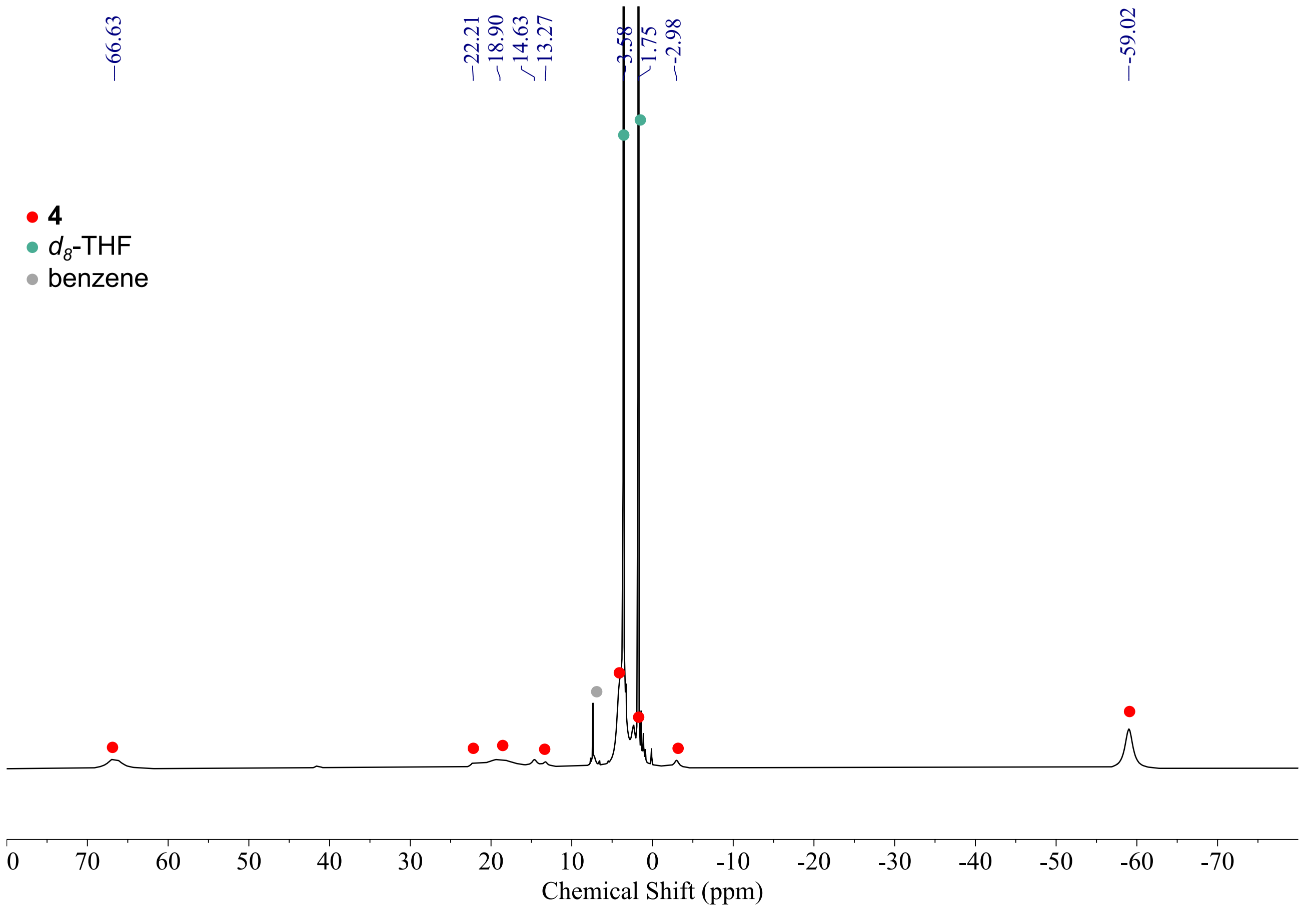

Figure S19. ^{1}H NMR spectrum (400 MHz, THF-d$_8$, 193 K) of isolated **4** • [K(2.2.2-cryptand)(THF)][PhNNPh].

.

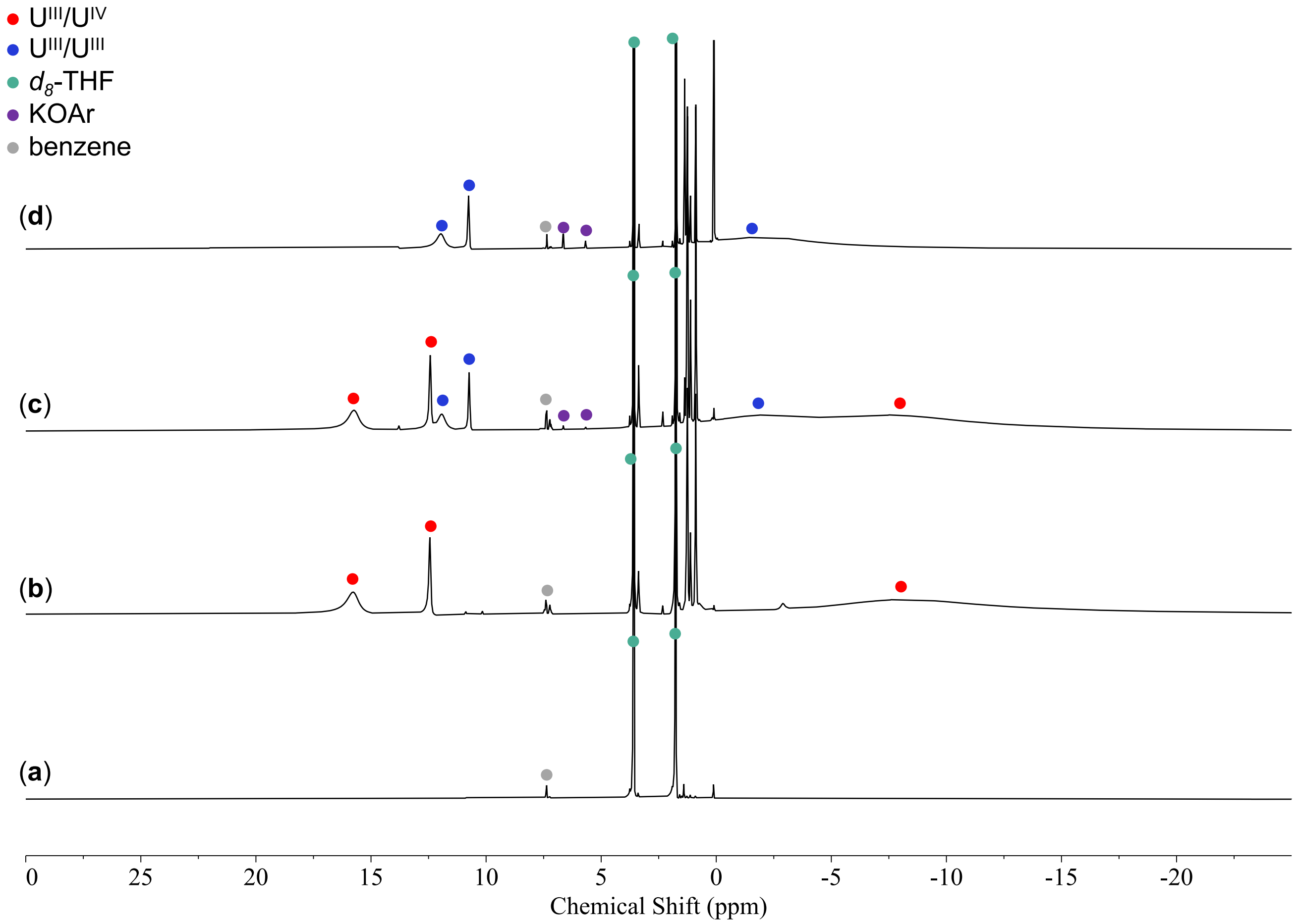

Figure S20. ^{1}H NMR spectra (400 MHz, THF-d_8, 193 K) of the reaction mixture obtained after addition of 1.0-5.0 equiv. of KC_8 to **B** at -80 °C (a) before (NMR silent) (b) **B** and 1.0 equiv. of KC_8 after 20 mins (c) **B** and 2.0 equiv. of KC_8 after 20 mins (d) **B** and 5.0 equiv. of KC_8 after 20 mins (U^{III}/U^{IV} = "$[K(THF)_x][(U(OAr)_3)_2(\mu\text{-}S)]$" and U^{III}/U^{III} = "$[K(THF)_x]_2[(U(OAr)_3)_2(\mu\text{-}S)]$").

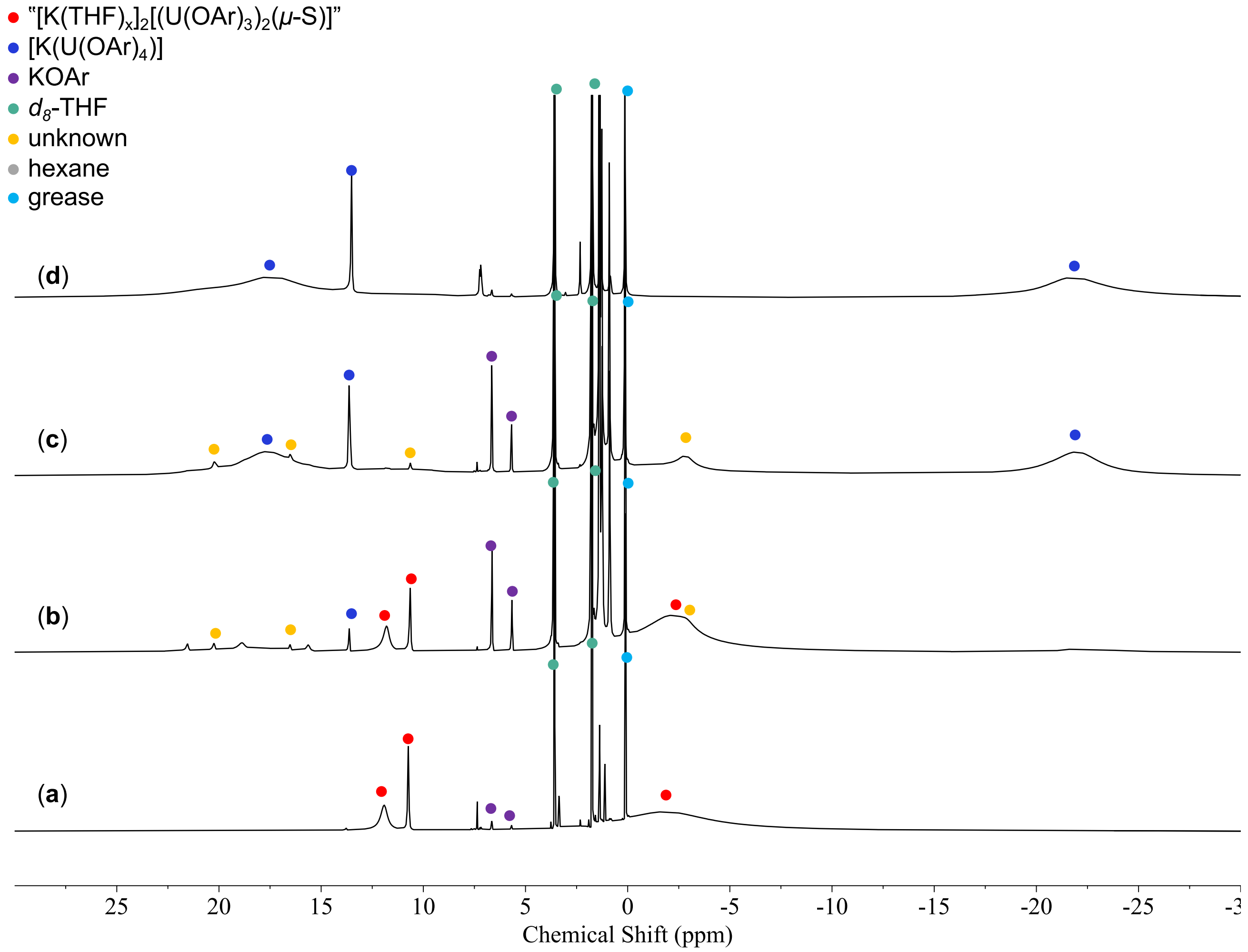

Figure S21. ^{1}H NMR spectra (400 MHz, THF-d_8, 193 K) of the reaction mixture obtained after addition of 5.0 equiv. of KC_8 to **B** at -80 °C (a) **B** and 5.0 equiv. of KC_8 after 20 mins at -80 °C (b) **B** and 5.0 equiv. of KC_8 after being warmed up to -40 °C for 1 week (c) **B** and 5.0 equiv. of KC_8 after being warmed up to -40 °C for 3 weeks (d) $[U(OAr)_4]$ and 1.2 equiv. of KC_8 after 20 mins, yielded $[KU(OAr)_4]$.

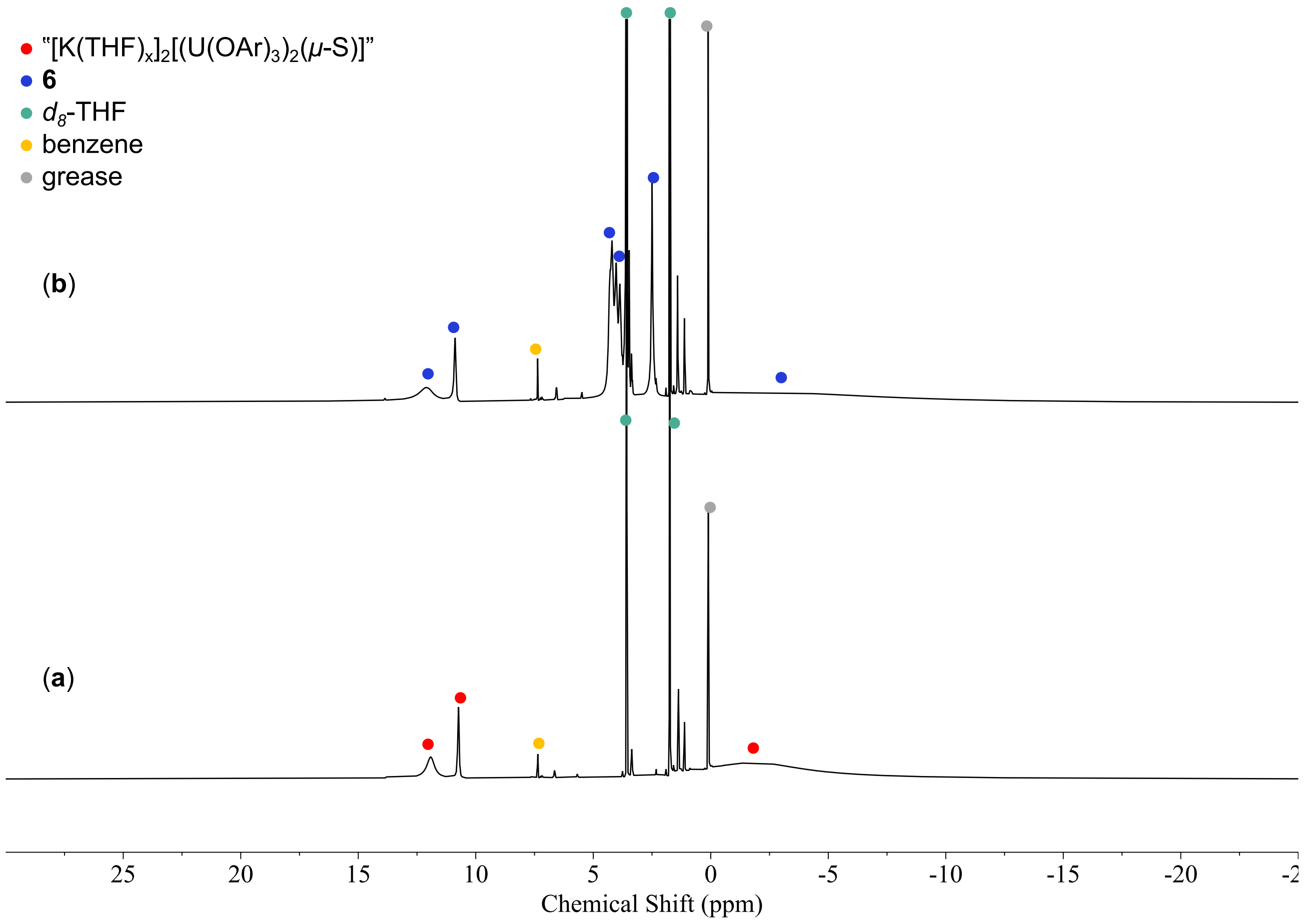

Figure S22. ^{1}H NMR spectra (400 MHz, THF-d_8, 193 K) of the reaction mixture obtained after addition of 2.0 equiv. of 2.2.2-cryptand to"[K(THF)$_x$]$_2$[(U(OAr)$_3$)$_2$(μ-S)]" at -80 °C (a) before (b) "[K(THF)$_x$]$_2$[(U(OAr)$_3$)$_2$(μ-S)]" and 2.0 equiv. of 2.2.2-cryptand immediately, showing the presence of complex **6**.

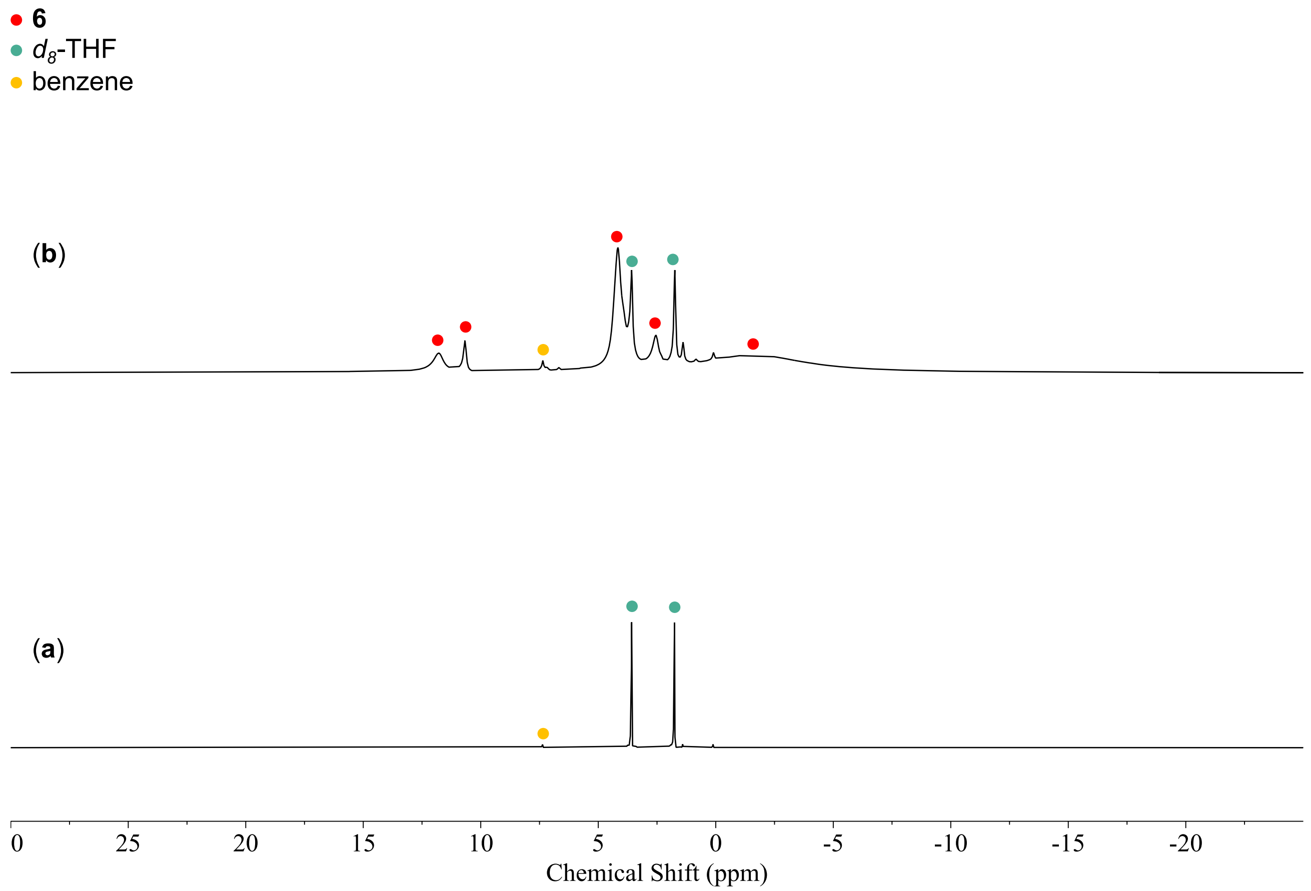

Figure S23. ^{1}H NMR spectra (400 MHz, THF-d_8, 193 K) of the reaction mixture obtained after addition of 5.0 equiv. of KC_8 and 2.0 equiv. of 2.2.2-cryptand to **B** at -80 °C (a) before (NMR silent) (b) **B** and 5.0 equiv. of KC_8 and 2.0 equiv. of 2.2.2-cryptand after 20 mins, showing the presence of complex **6**.

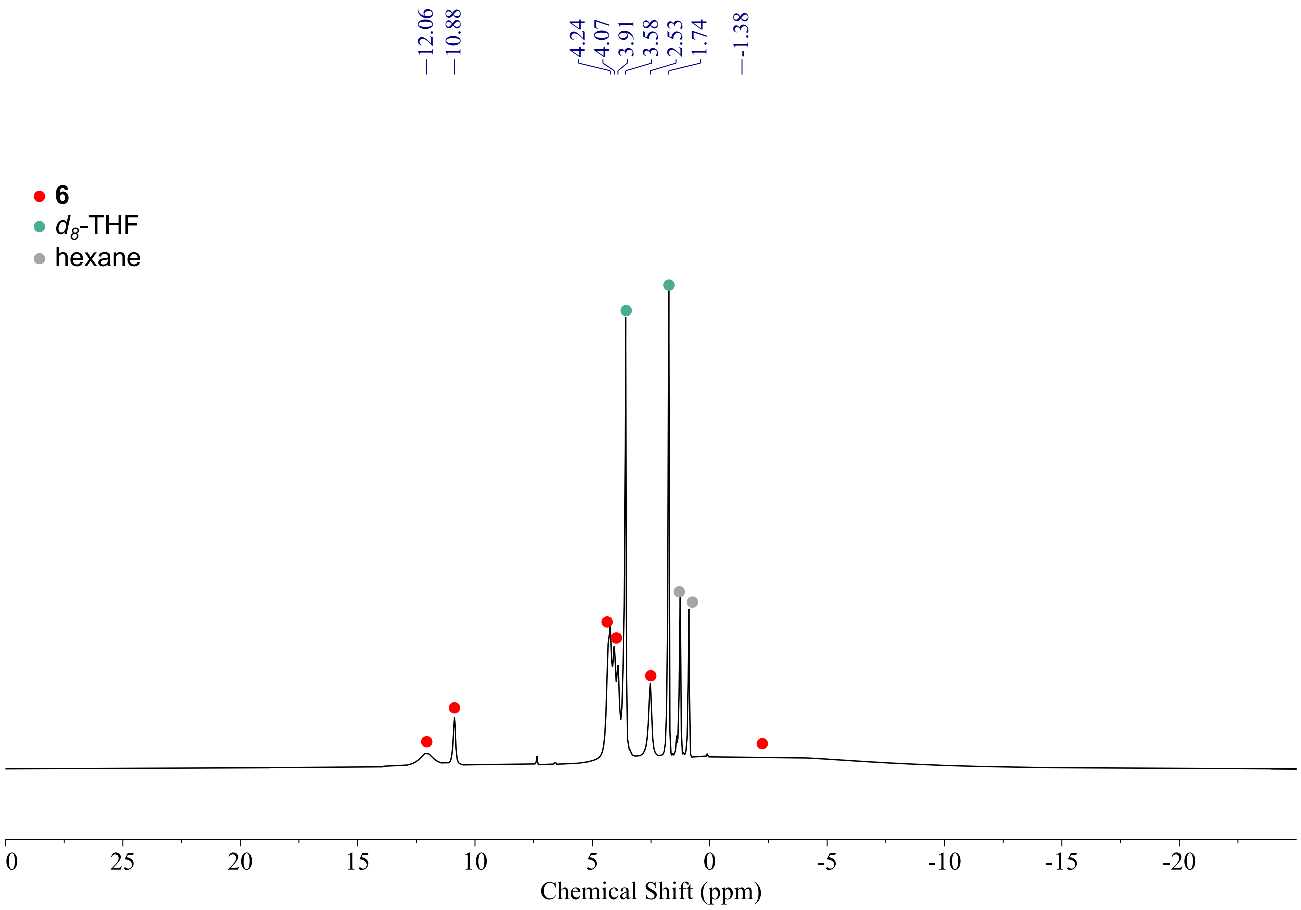

Figure S24. ^{1}H NMR spectrum (400 MHz, THF-d$_8$, 193 K) of isolated **6**.

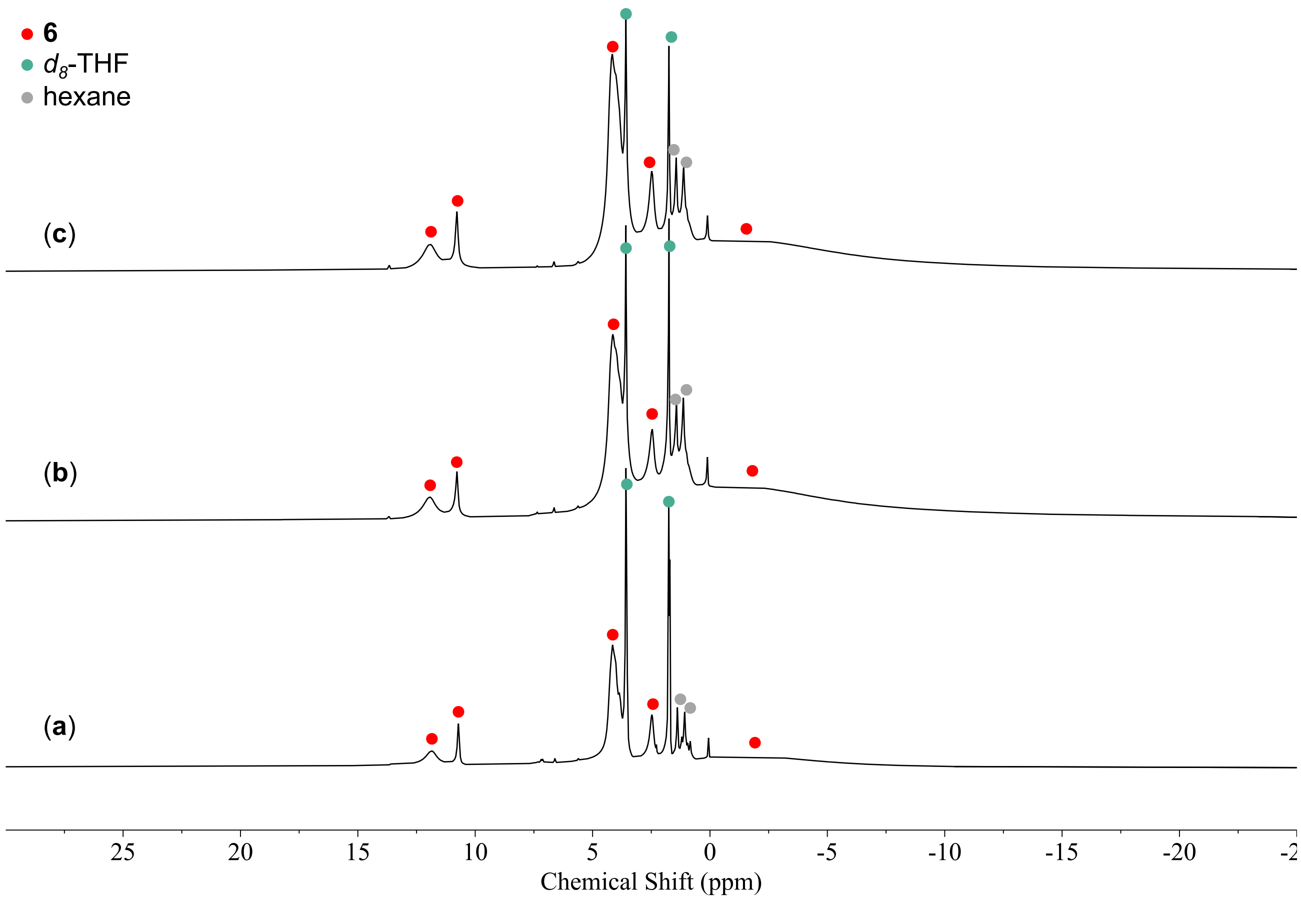

Figure S25. ^{1}H NMR spectra (400 MHz, THF-d_8, 193 K) of the isolated **6** (a) before (b) **3** after being warmed up to -40 °C for 3 days (c) **3** after being warmed up to -40 °C for 1 week.

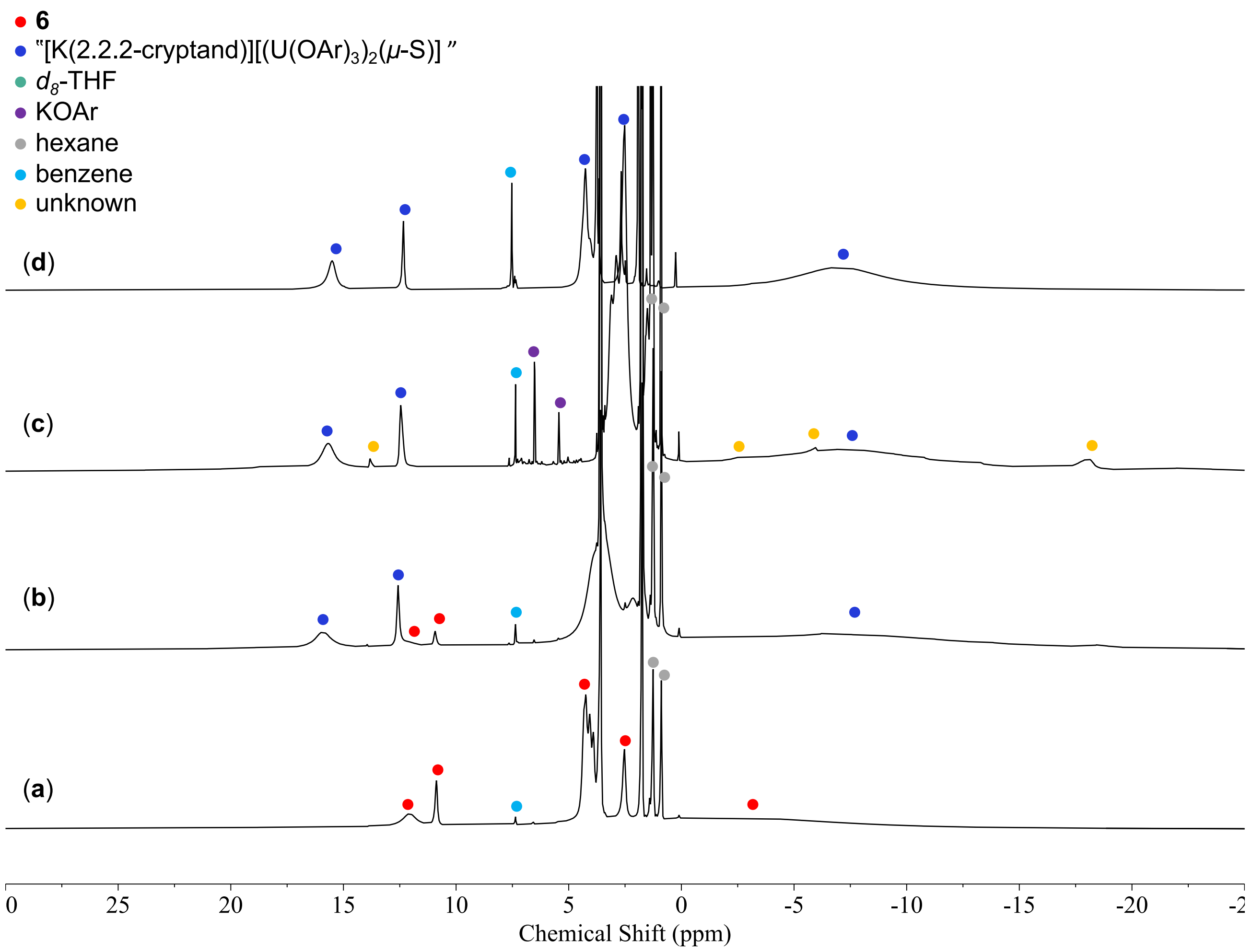

Figure S26. ^{1}H NMR spectra (400 MHz, THF-d_8, 193 K) of the reaction mixture obtained after addition of 1.0 equiv. of PhNNPh to **6** at -80 °C (a) before (b) **3** and 1.0 equiv. of PhNNPh immediately (c) **6** and 1.0 equiv. of PhNNPh after being warmed up to -40 °C for 1 h (d) **B** and 1.0 equiv. of KC_8 and 1.0 equiv. of 2.2.2-cryptand after 20 mins.

S4. Electrochemistry Data

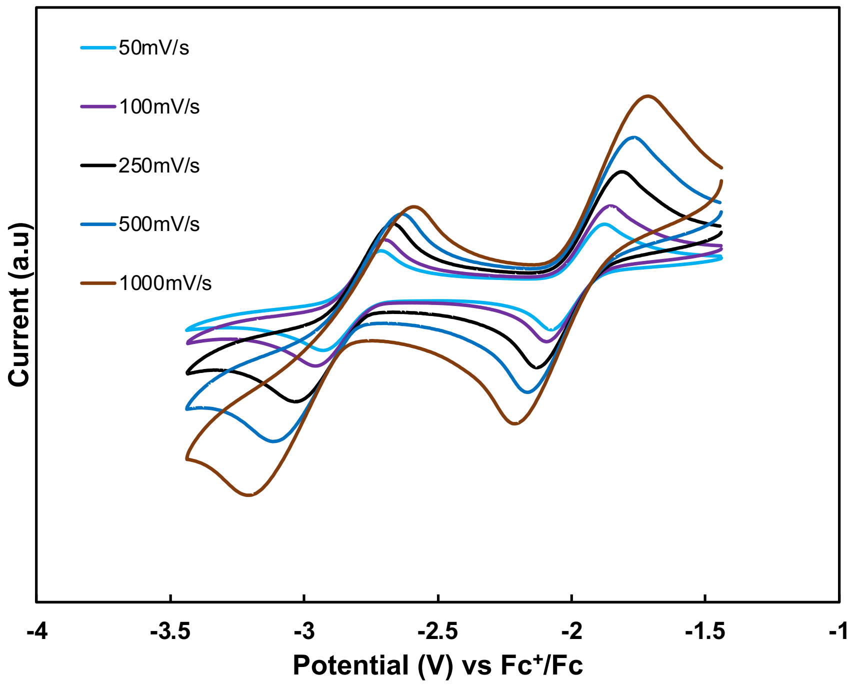

Figure S27. Cyclic voltammogram of **A** in THF at room temperature with varying scan rates.

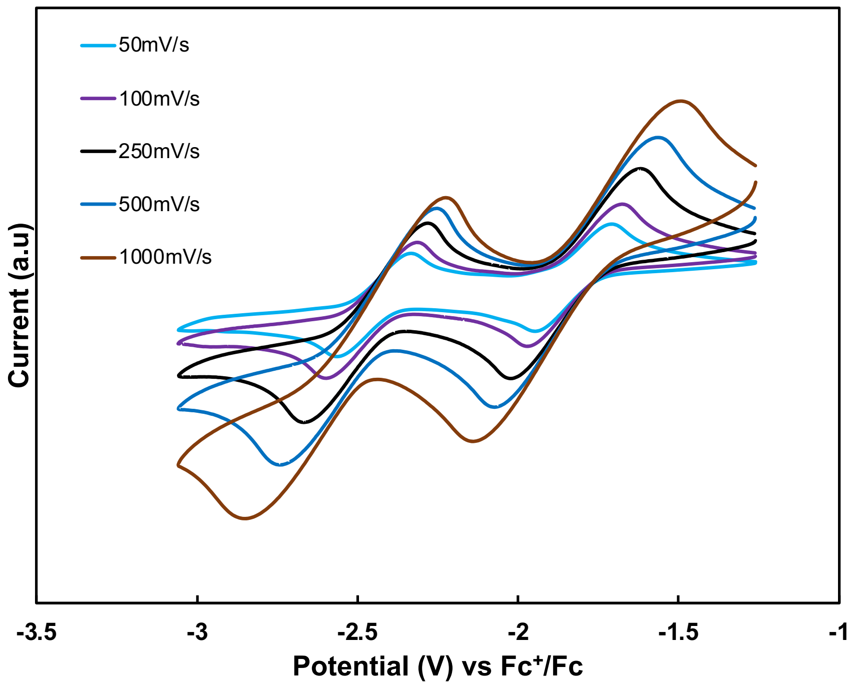

Figure S28. Cyclic voltammogram of **B** in THF at room temperature with varying scan rates.

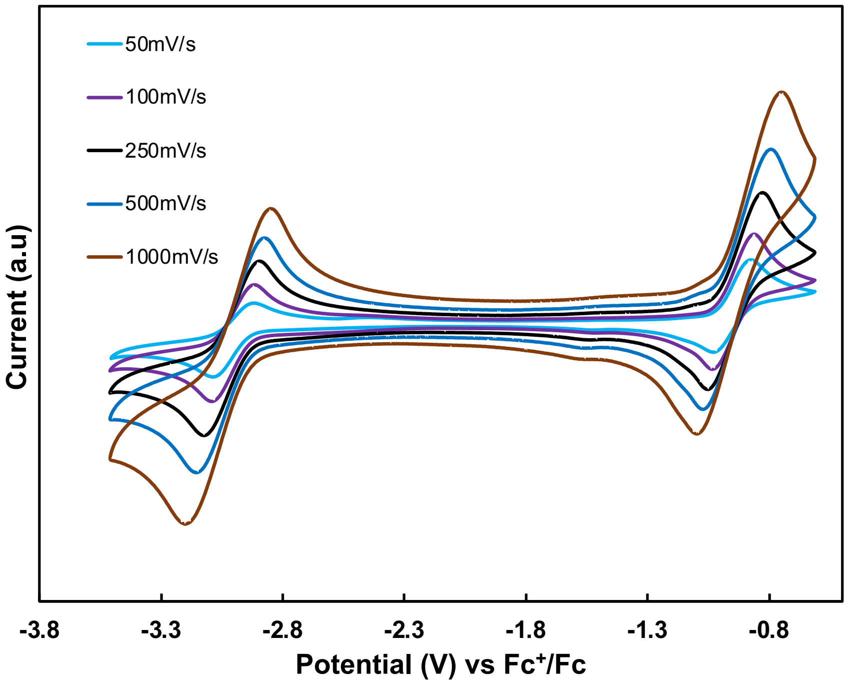

Figure S29. Cyclic voltammogram of **1** in THF at room temperature with varying scan rates.

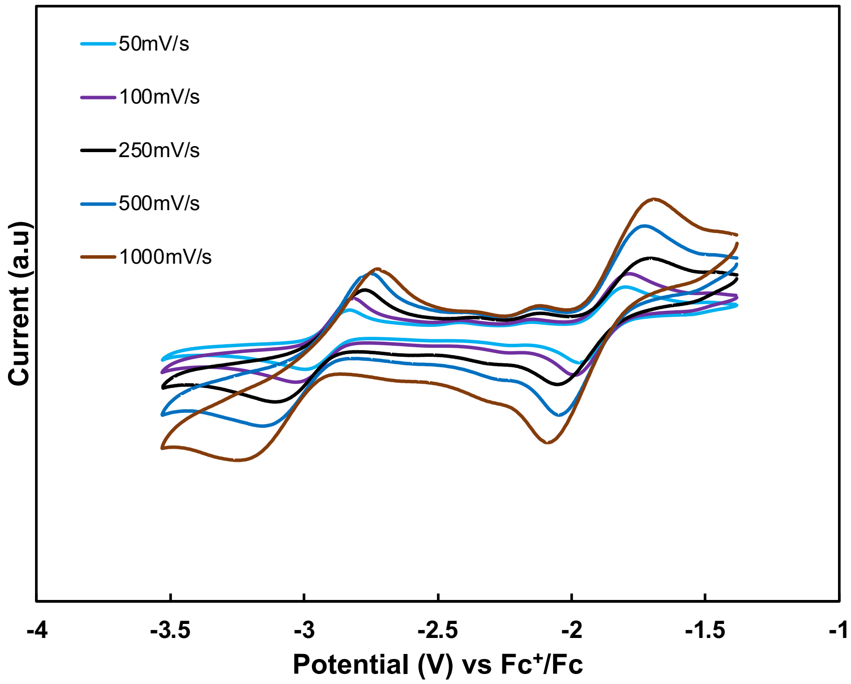

Figure S30. Cyclic voltammogram of $[(U(N(SiMe_3)_2)_3)_2(\mu\text{-}O)]$ in THF at room temperature with varying scan rates.

S5. SQUID Magnetometry Data

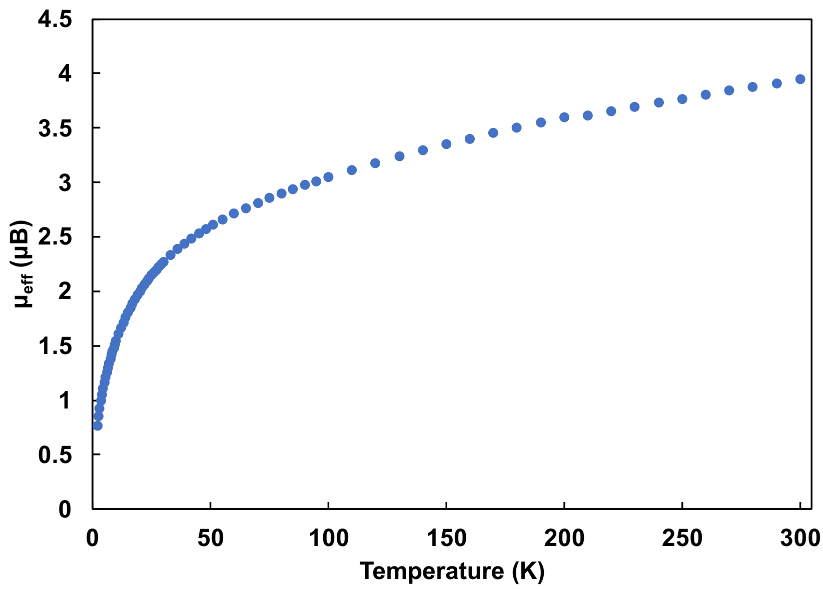

Figure S31. Plot of μ_{eff} per ion versus T data for **A** under an applied field of 1 T.

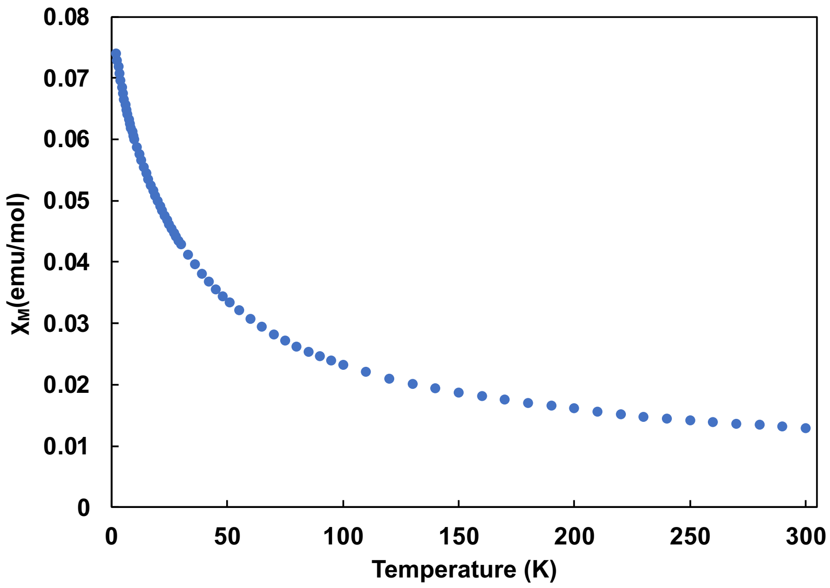

Figure S32. Plot of χ_M versus temperature data for **A** under an applied field of 1 T.

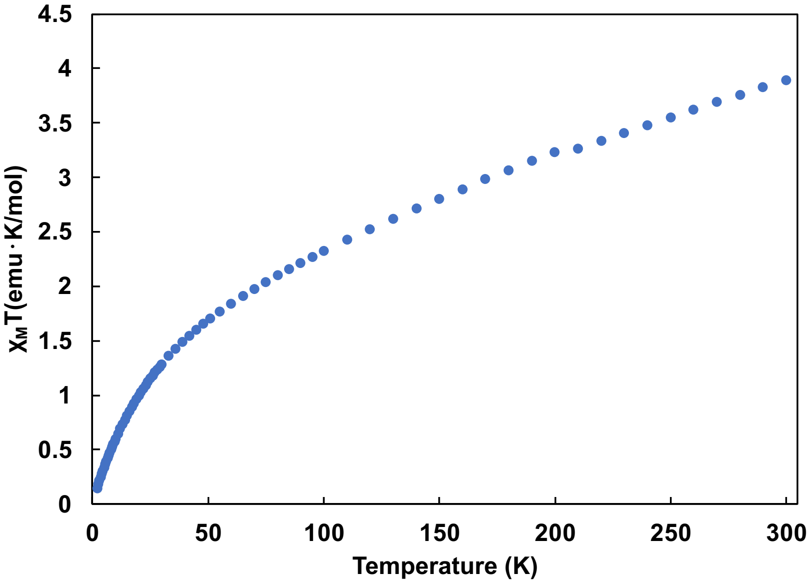

Figure S33. Plot of χ_MT versus temperature data for **A** under an applied field of 1 T.

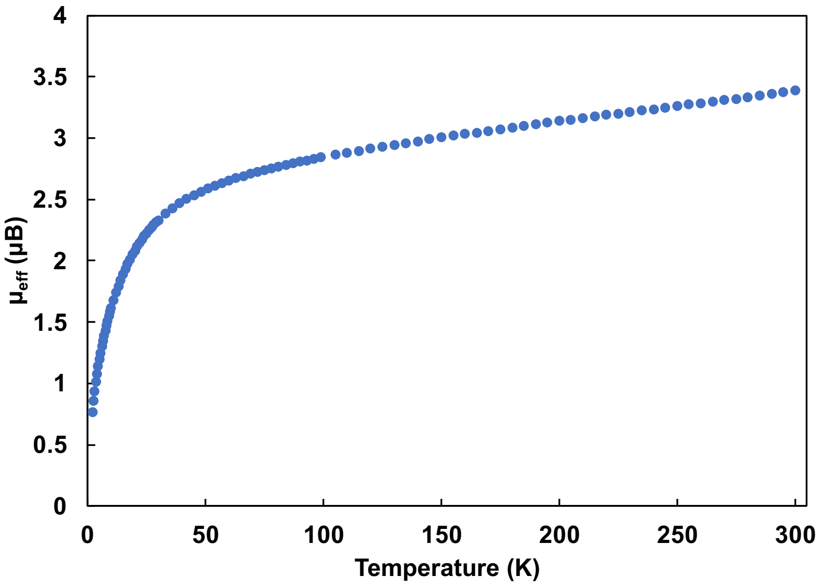

Figure S34. Plot of μ_{eff} per ion versus T data for **B** under an applied field of 1 T.

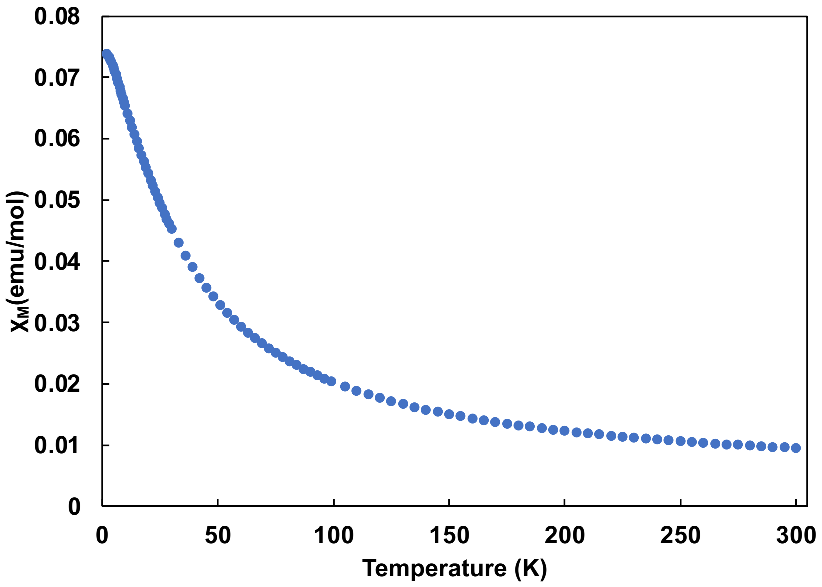

Figure S35. Plot of χ_M versus temperature data for **B** under an applied field of 1 T.

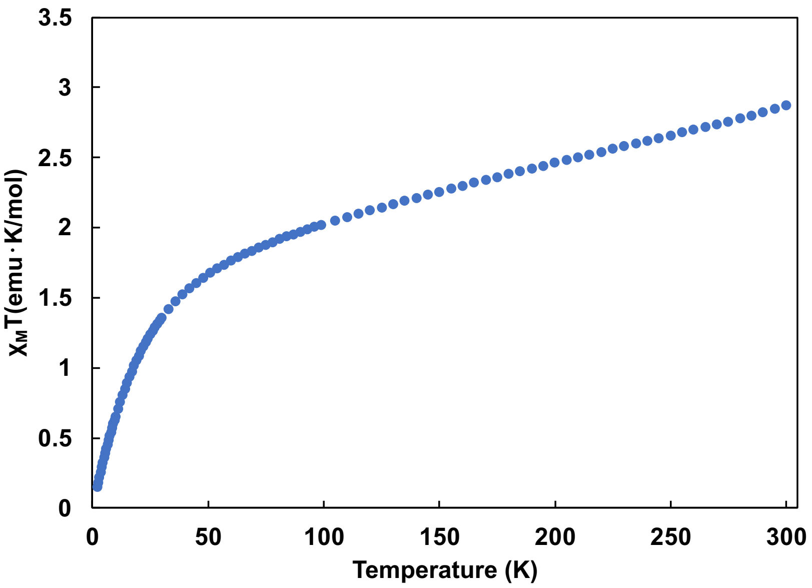

Figure S36. Plot of χ_MT versus temperature data for **B** under an applied field of 1 T.

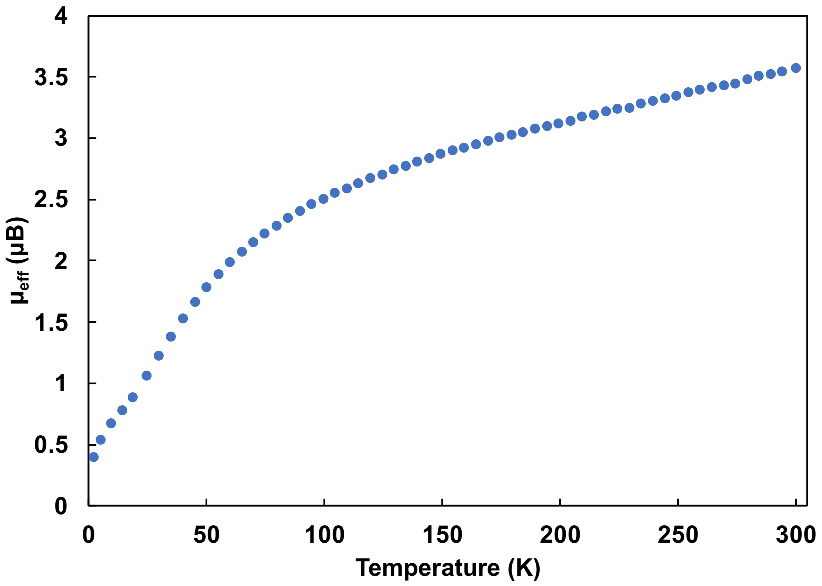

Figure S37. Plot of μ_{eff} per ion versus T data for **1** under an applied field of 1 T.

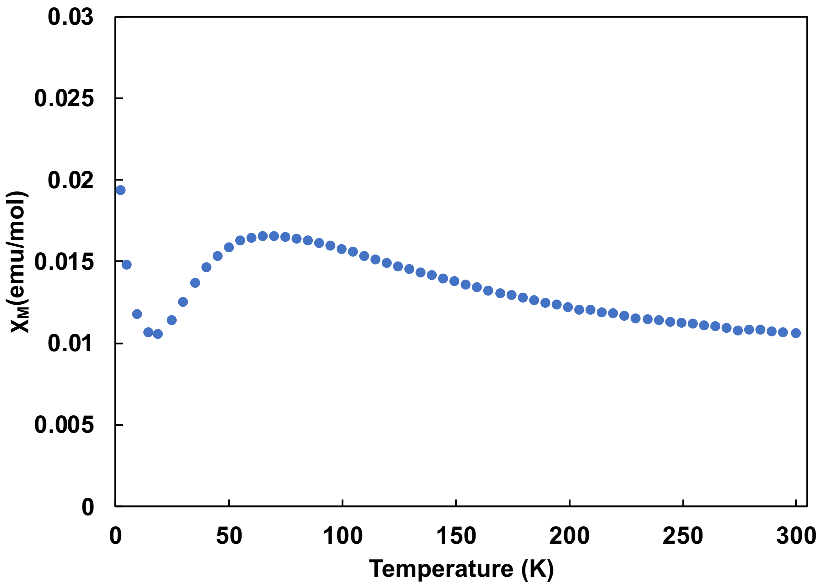

Figure S38. Plot of χ_M versus temperature data for **1** under an applied field of 1 T.

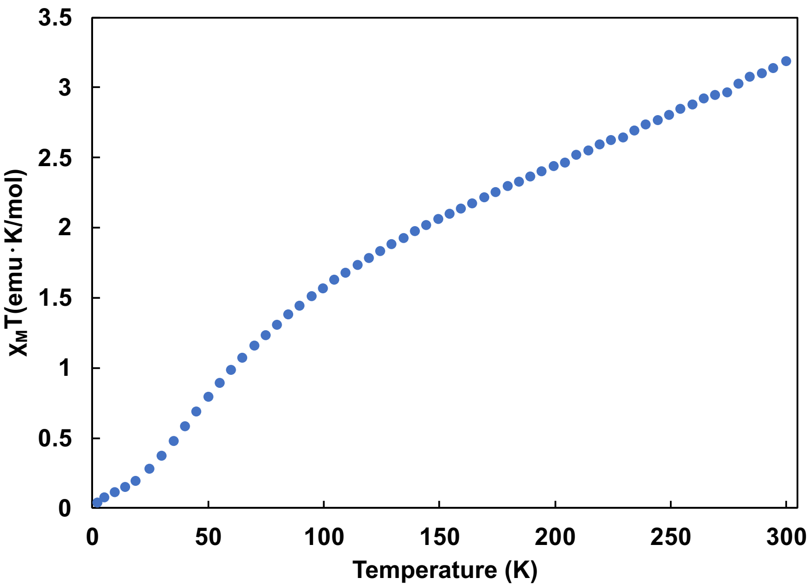

Figure S39. Plot of χ_MT versus temperature data for **1** under an applied field of 1 T.

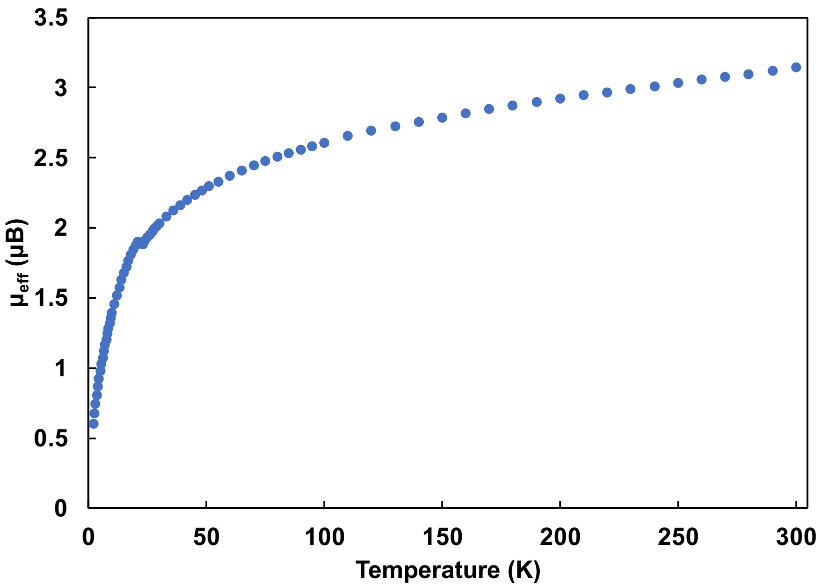

Figure S40. Plot of μ_{eff} per ion versus T data for **3** under an applied field of 1 T.

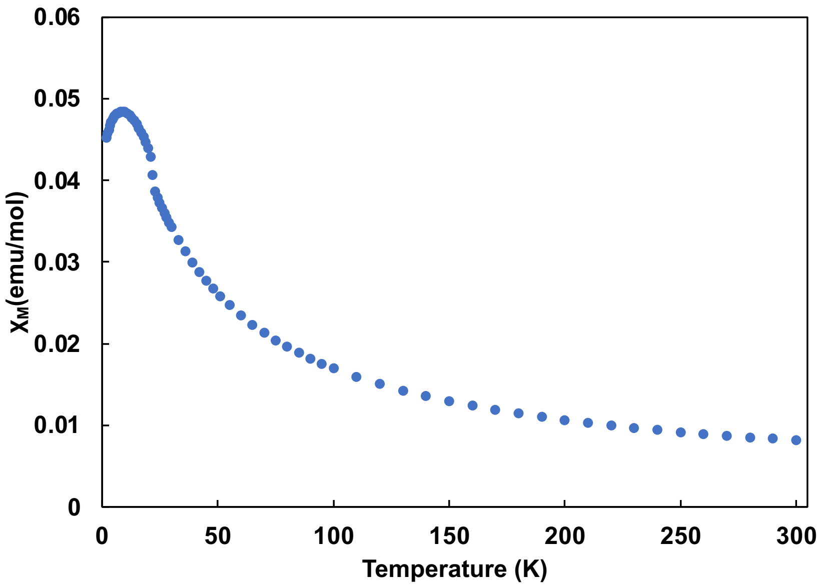

Figure S41. Plot of χ_M versus temperature data for **3** under an applied field of 1 T.

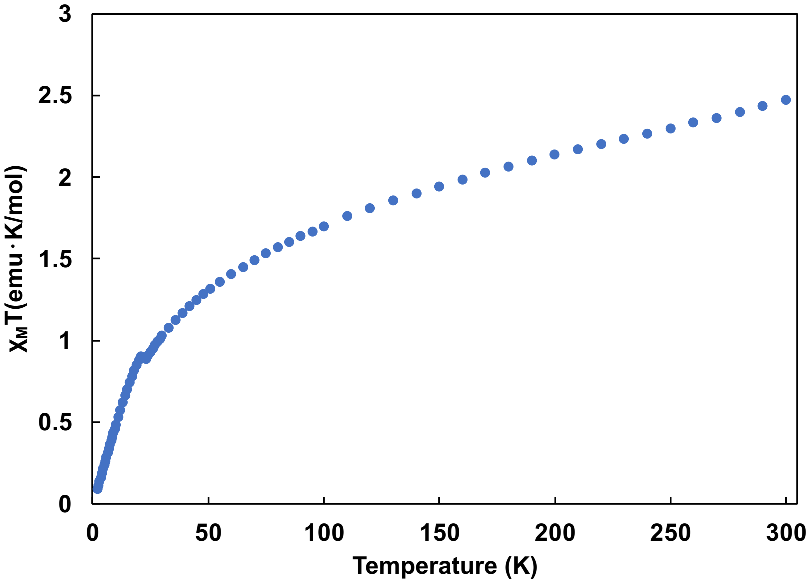

Figure S42. Plot of χ_MT versus temperature data for **3** under an applied field of 1 T.

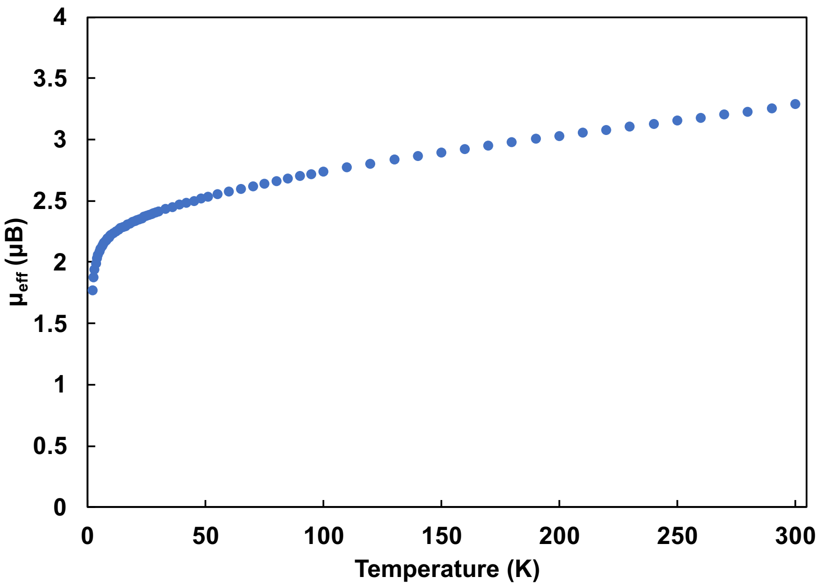

Figure S43. Plot of μ_{eff} per ion versus T data for **6** under an applied field of 1 T.

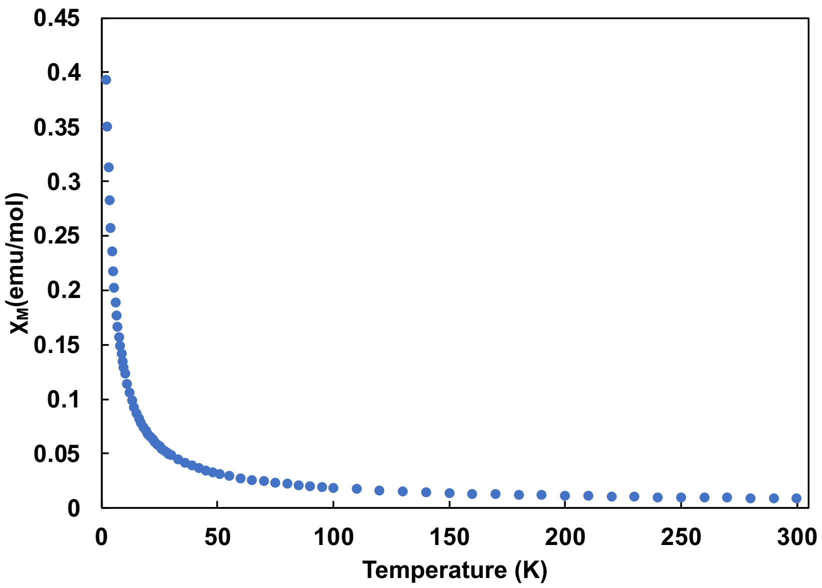

Figure S44. Plot of χ_M versus temperature data for **6** under an applied field of 1 T.

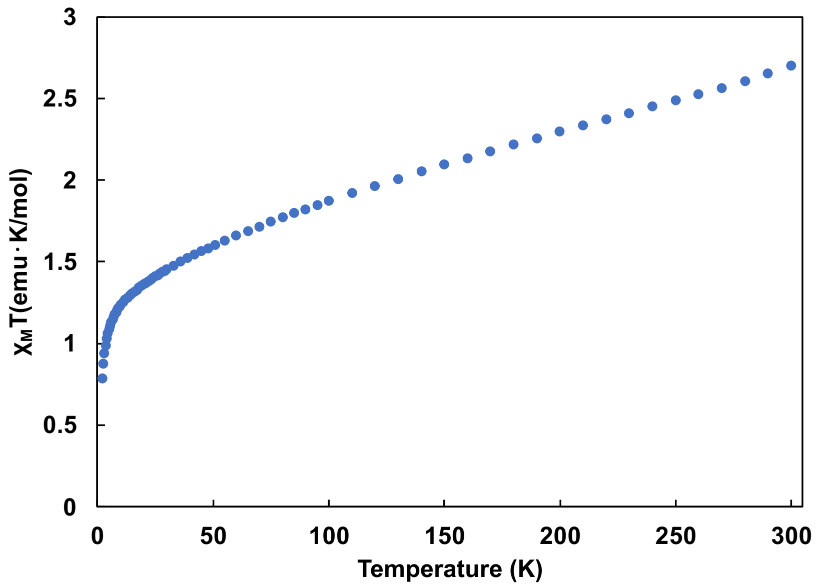

Figure S45. Plot of χ_MT versus temperature data for **6** under an applied field of 1 T.

S6. X-ray Crystallography Data

Table S1. Crystal data and structural refinement parameters for complexes **1**, **2**·$(C_6H_{14})_2$·$(THF)_2$, and **4**·$(C_{12}H_{10}N_2)$·$(C_{22}H_{44}KN_2O_7)$·$(THF)_4$.

	**1**	**2**·$(C_6H_{14})_2$·$(THF)_2$	**4**·$(C_{12}H_{10}N_2)$·$(C_{22}H_{44}KN_2O_7)$·$(THF)_4$
Formula	$C_{116}H_{190}CsNO_{14}U_2$	$C_{120}H_{202}LiO_{13}U_2$	$C_{156}H_{256}K_2N_6O_{25}U_2$
Crystal size (mm)	0.17×0.15×0.10	0.42×0.23×0.21	0.29×0.12×0.12
Crystal System	monoclinic	triclinic	triclinic
Space Group	$P2_1/c$	*P*-1	*P*-1
Volume (Å^3)	5858.50(19)	5890.70(12)	4127.8(4)
a (Å)	14.8121(3)	14.9084(2)	15.1585(8)
b (Å)	19.1272(4)	19.4995(2)	16.4391(10)
c (Å)	21.0703(4)	21.0862(2)	18.5969(10)
α (°)	90	102.1051(10)	77.156(5)
β (°)	101.0671(19)	97.5370(10)	66.138(5)
γ (°)	90	95.7018(11)	82.519(5)
Z	2	2	1
Formula Weight	2431.763	2335.80	3170.052
Density (g cm^{-3})	1.379	1.317	1.275
μ (mm^{-1})	10.549	8.110	6.436
F(000)	2477.5	2426	1653
Temperature (K)	173.04(14)	140.00(10)	140.00(10)
Total Reflections	25396	28682	31475
Unique Reflections	11310	28682	15234
R_{int}	0.0298	-	0.0866
R Indices [I > 2σ(I)]	R_1 = 0.0464	R_1 = 0.0407	R_1 = 0.0721
	wR_2 = 0.1175	wR_2 = 0.1130	wR_2 = 0.1962
Largest Diff. Peak	1.7075 and	1.756 and	2.6949 and
and Hole (e.A^{-3})	-1.9778	-1.913	-2.5288
GOF	1.0868	1.023	1.0220

F(000), structure factor evaluated in the zeroth-order case, h=k=l= 0; R(int) =∑|Fo2−Fo2(mean)| /∑[Fo2]; I, measured intensities; 'Largest diff. peak and hole', maximum and minimum electron density found in the final Fourier difference map; GOF, goodness of fit (= {∑ [w(Fo2−Fc2)2]/(n−p)}1/2, where n is the number of reflections and p is the total number of parameters refined).

Table S2. Crystal data and structural refinement parameters for complexes **5** and **6**.

	**5**	**6**
Formula	$C_{88}H_{148}K_2O_{12}S_2U_2$	$C_{120}H_{198}K_2N_4O_{18}SU_2$
Crystal size (mm)	0.34×0.24×0.20	0.13×0.08×0.04
Crystal System	triclinic	triclinic
Space Group	*P*-1	*P*-1
Volume (Å^3)	2627.79(5)	7113.18(10)
a (Å)	13.70485(13)	14.45571(11)
b (Å)	14.20424(13)	17.05206(17)
c (Å)	15.06289(17)	29.72010(19)
α (°)	93.7454(9)	79.8697(7)
β (°)	113.9243(10)	80.6323(6)
γ (°)	98.2448(8)	89.8128(7)
Z	1	2
Formula Weight	2016.44	2571.13
Density (g cm^{-3})	1.274	1.200
μ (mm^{-1})	10.062	7.447
F(000)	1026	2656
Temperature (K)	139.99(10)	139.99(10)
Total Reflections	50894	244318
Unique Reflections	10292	28387
R_{int}	0.0266	0.0760
R Indices [I > 2σ(I)]	R_1 = 0.0236	R_1 = 0.0517
	wR_2 = 0.0605	wR_2 = 0.1243
Largest Diff. Peak	1.405 and	3.183 and
and Hole (e.A^{-3})	-1.224	-1.385
GOF	1.056	1.070

F(000), structure factor evaluated in the zeroth-order case, h=k=l= 0; R(int) =∑|Fo2−Fo2(mean)| /∑[Fo2]; I, measured intensities; 'Largest diff. peak and hole', maximum and minimum electron density found in the final Fourier difference map; GOF, goodness of fit (= {∑ [w(Fo2−Fc2)2]/(n−p)}1/2, where n is the number of reflections and p is the total number of parameters refined).

S7. Computational Details

The optimization of three different spin states for uranium complexes were carried out by employing DFT hybrid functional (B3PW91)[11] along with small core pseudopotential Stuttgart basis set for uranium and silicon atoms with additional polarization functions for silicon atoms.[12] Pople basis sets (6-31G**) were employed for the rest of the atoms.[13] Dispersion corrections were included in our calculations by employing D3 version of Grimme's dispersion with Becke-Johnson damping.[14] To account for the solvation effects, SMD model using THF solvent has been included in the calculations.[15] All the calculations were performed using Gaussian 09 suite of programs.[16]

Computed natural charges for **$[^{Si}L_3UOU^{Si}L_3]^{2-}$**, **$[^{Si}L_3UOU^{Si}L_3]^{1-}$**, **$[^{Si}L_3UOU^{Si}L_3]$**, **$[^{Si}L_3UNU^{Si}L_3]^{3-}$**, **$[^{Si}L_3UNU^{Si}L_3]^{2-}$**, **$[^{Si}L_3UNU^{Si}L_3]^{1-}$**, **$[^{Si}L_3USU^{Si}L_3]^{2-}$**, **$[^{Si}L_3USU^{Si}L_3]^{1-}$**, **$[^{Si}L_3USU^{Si}L_3]$**

	Natural charges								
Atom label	**$[UOU]^{2-}$** S=3	**$[UOU]^{1-}$** S=5/2	**[UOU]** S=2	**$[UNU]^{3-}$** S=3	**$[UNU]^{2-}$** S=5/2	**[UNU]** S=2	**$[USU]^{2-}$** S=3	**$[USU]^{1-}$** S=5/2	**[USU]** S=2
U1	1.82124	2.08532	2.16329	1.59046	1.91077	2.05749	1.79053	2.06780	2.15181
O5/N5/S5	-1.16267	-1.08419	-1.06808	-1.51273	-1.39435	-1.35079	-1.14441	-0.93792	-0.85101
O6	-1.29294	-1.23600	-1.22221	-1.27834	-1.25575	-1.23950	-1.29067	-1.23085	-1.21359
O10	-1.29797	-1.23712	-1.21592	-1.28360	-1.26733	-1.25336	-1.29745	-1.23481	-1.22396
O14	-1.29455	-1.22286	-1.20311	-1.27824	-1.26065	-1.24535	-1.29042	-1.21981	-1.20966
U135	1.80001	1.89720	2.22493	1.59635	1.71184	2.01271	1.73066	1.74755	2.00816
O139	-1.29203	-1.28678	-1.22069	-1.28288	-1.27316	-1.23241	-1.29039	-1.28392	-1.20280
O143	-1.29055	-1.29308	-1.22606	-1.27901	-1.28392	-1.24162	-1.28254	-1.28627	-1.21264
O147	-1.28902	-1.29247	-1.22541	-1.27604	-1.27399	-1.23021	-1.28897	-1.27501	-1.21157

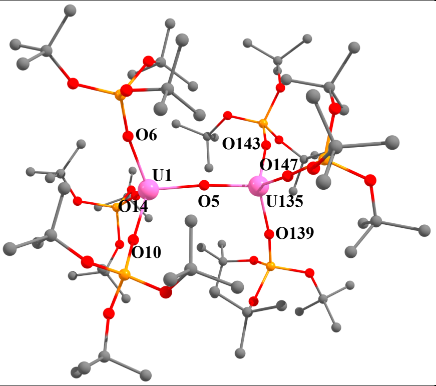

DFT computed spin densities on selected atoms in **$[^{Si}L_3UOU^{Si}L_3]^{2-}$**, **$[^{Si}L_3UOU^{Si}L_3]^{1-}$**, **$[^{Si}L_3UOU^{Si}L_3]$**, **$[^{Si}L_3UNU^{Si}L_3]^{3-}$**, **$[^{Si}L_3UNU^{Si}L_3]^{2-}$**, **$[^{Si}L_3UNU^{Si}L_3]^{1-}$**, **$[^{Si}L_3USU^{Si}L_3]^{2-}$**, **$[^{Si}L_3USU^{Si}L_3]^{1-}$**, **$[^{Si}L_3USU^{Si}L_3]$**

Atom label	**$[UOU]^{2-}$** S=3	**$[UOU]^{1-}$** S=5/2	**[UOU]** S=2	**$[UNU]^{3-}$** S=3	**$[UNU]^{2-}$** S=5/2	**$[UNU]^{1-}$** S=2	**$[USU]^{2-}$** S=3	**$[USU]^{1-}$** S=5/2	**[USU]** S=2
U1	3.11	2.14	2.11	3.12	2.21	2.14	3.11	2.20	2.13
O5/N5/S5	-0.01	-0.08	-0.06	-0.19	-0.18	-0.13	-0.07	-0.11	-0.08
O6	-0.03	-0.02	-0.02	-0.02	-0.02	-0.02	-0.03	-0.02	-0.03
O10	-0.03	-0.02	-0.03	-0.02	-0.02	-0.02	-0.03	-0.02	-0.02
O14	-0.03	-0.02	-0.02	-0.02	-0.02	-0.02	-0.03	-0.03	-0.03
U135	3.10	3.07	2.10	3.10	3.06	2.11	3.12	3.07	2.13
O139	-0.03	-0.03	-0.02	-0.02	-0.03	-0.02	-0.03	-0.03	-0.03
O143	-0.03	-0.03	-0.03	-0.03	-0.02	-0.02	-0.03	-0.03	-0.03
O147	-0.03	-0.03	-0.02	-0.02	-0.02	-0.02	-0.03	-0.03	-0.03

Computed Wiberg bond index for ground spin state of **[$^{Si}L_3UOU^{Si}L_3$]$^{2-}$**, **[$^{Si}L_3UOU^{Si}L_3$]$^{1-}$**, **[$^{Si}L_3UOU^{Si}L_3$]**, **[$^{Si}L_3UNU^{Si}L_3$]$^{3-}$**, **[$^{Si}L_3UNU^{Si}L_3$]$^{2-}$**, **[$^{Si}L_3UNU^{Si}L_3$]$^{1-}$**, **[$^{Si}L_3USU^{Si}L_3$]$^{2-}$**, **[$^{Si}L_3USU^{Si}L_3$]$^{1-}$**, **[$^{Si}L_3USU^{Si}L_3$]**

Atom label	Wiberg bond index	Atom label	Wiberg bond index	Atom label	Wiberg bond index	Atom label	Wiberg bond index
U1	0.0000	U1	0.0000	U1	0.0000	U1	0.0000
O5/N5/S5	0.6812<br>1.0474<br>0.7852<br>1.1145<br>1.5861<br>1.2448<br>0.6677<br>1.0799<br>0.8210	O6	0.4744<br>0.6598<br>0.7176<br>0.4374<br>0.5422<br>0.6219<br>0.5051<br>0.6887<br>0.7475	O10	0.4643<br>0.6532<br>0.7292<br>0.4353<br>0.5174<br>0.5904<br>0.4708<br>0.6737<br>0.7408	O14	0.4670<br>0.6465<br>0.7238<br>0.4262<br>0.5293<br>0.6039<br>0.5069<br>0.6831<br>0.7305
Atom label	Wiberg bond index	Atom label	Wiberg bond index	Atom label	Wiberg bond index	Atom label	Wiberg bond index
U135	0.0000	U135	0.0000	U135	0.0000	U135	0.0000
O5/N5/S5	0.7239<br>0.4903<br>0.7743<br>1.1113<br>0.8241<br>1.2284<br>0.7381<br>0.6152<br>0.9824	O139	0.4682<br>0.5088<br>0.7083<br>0.4366<br>0.4960<br>0.6466<br>0.5035<br>0.5357<br>0.7677	O143	0.4806<br>0.5101<br>0.7062<br>0.4462<br>0.4757<br>0.6226<br>0.5127<br>0.5177<br>0.7343	O147	0.4578<br>0.5051<br>0.7140<br>0.4272<br>0.4702<br>0.6131<br>0.4819<br>0.5201<br>0.7250

Bonding orbitals from NBO analysis for ground spin state (Alpha spin orbitals, UOU core) of [$^{Si}L_3UOU^{Si}L_3$]$^{2-}$, (S=3)

(0.93210) BD (1) O 5- U135
(92.07%)   0.9595* O 5 s(0.04%)p99.99(99.96%)d 0.03(0.00%)
(7.93%)   0.2817* U135 s(0.01%)p 1.00(0.43%)d99.99(63.90%)f83.13(35.63%)g 0.06(0.03%)
(0.93113) BD (2) O 5- U135
(92.33%)   0.9609* O 5 s(0.30%)p99.99(99.70%)d 0.00(0.00%)
(7.67%)   0.2769* U135 s(0.09%)p 6.82(0.58%)d99.99(65.32%)f99.99(33.98%)g 0.35(0.03%)

Bonding orbitals from NBO analysis for ground spin state (Alpha spin orbitals, UOU core) of [$^{Si}L_3UOU^{Si}L_3$]$^{1-}$, (s=5/2)

(0.98237) BD (1) U 1- O 5
(10.45%)   0.3233* U 1 s(0.30%)p 3.45(1.04%)d99.99(72.33%) f86.45(26.04%)g 0.94(0.28%)
(89.55%)   0.9463* O 5 s(45.10%)p 1.22(54.86%)d 0.00(0.04%)
(0.95272) BD (2) U 1- O 5
(10.50%)   0.3240* U 1 s(0.10%)p 8.23(0.81%)d99.99(45.24%) f99.99(53.77%)g 0.91(0.09%)
(89.50%)   0.9460* O 5 s(0.04%)p99.99(99.94%)d 0.29(0.01%)
(0.94999) BD (3) U 1- O 5
(10.42%)   0.3228* U 1 s(0.04%)p11.38(0.47%)d99.99(50.19%) f99.99(49.20%)g 2.13(0.09%)
(89.58%)   0.9465* O 5 s(0.00%)p 1.00(99.99%)d 0.00(0.01%)

Bonding orbitals from NBO analysis for ground spin state (Alpha spin orbitals, UOU core) of [$^{Si}L_3UOU^{Si}L_3$], (s=2)

(0.97777) BD (1) U 1- O 5
(8.53%)   0.2921* U 1 s(1.28%)p 0.57(0.73%)d60.55(77.54%) f15.75(20.17%)g 0.23(0.29%)
(91.47%)   0.9564* O 5 s(49.67%)p 1.01(50.32%)d 0.00(0.02%)
(0.97207) BD (1) O 5- U135
(93.02%)   0.9645* O 5 s(50.11%)p 1.00(49.87%)d 0.00(0.01%)
(6.98%)   0.2642* U135 s(1.79%)p 1.43(2.56%)d36.24(64.99%) f15.50(27.80%)g 1.59(2.85%)

Bonding orbitals from NBO analysis for ground spin state (Alpha spin orbitals, UNU core) of [$^{Si}L_3UNU^{Si}L_3$]$^{3-}$, (S=3)

(0.97486) BD (1) U 1- N 5
(14.47%)   0.3804* U 1 s(9.21%)p 0.17(1.58%)d 8.43(77.62%) f 1.25(11.54%)g 0.01(0.06%)
(85.53%)   0.9248* N 5 s(49.94%)p 1.00(50.04%)d 0.00(0.02%)
(0.97323) BD (1) N 5- U135
(86.12%)   0.9280* N 5 s(49.95%)p 1.00(50.04%)d 0.00(0.02%)

(13.88%)   0.3726* U135 s(6.38%)p 0.28(1.77%)d13.08(83.39%) f 1.32(8.41%)g 0.01(0.06%)

Bonding orbitals from NBO analysis for ground spin state (Alpha spin orbitals, UNU core) of $[^{Si}L_3UNU^{Si}L_3]^{2-}$, (S=5/2)

(0.98530) BD (1) U 1- N 5
(21.27%)   0.4612* U 1 s(0.14%)p 5.78(0.79%)d99.99(53.36%) f99.99(45.66%)g 0.34(0.05%)
(78.73%)   0.8873* N 5 s(40.46%)p 1.47(59.48%)d 0.00(0.06%)
(0.91884) BD (2) U 1- N 5
(19.69%)   0.4437* U 1 s(0.01%)p 1.00(0.24%)d99.99(52.90%) f99.99(46.83%)g 0.10(0.03%)
(80.31%)   0.8962* N 5 s(0.08%)p99.99(99.91%)d 0.20(0.02%)
(0.91461) BD (3) U 1- N 5
(19.04%)   0.4363* U 1 s(0.01%)p 1.00(0.29%)d99.99(54.10%) f99.99(45.58%)g 0.10(0.03%)
(80.96%)   0.8998* N 5 s(0.03%)p99.99(99.95%)d 0.48(0.02%)
(0.96932) BD (1) N 5- U135
(88.21%)   0.9392* N 5 s(59.43%)p 0.68(40.56%)d 0.00(0.01%)
(11.79%)   0.3433* U135 s(12.74%)p 0.07(0.84%)d 6.23(79.38%) f 0.55(6.99%)g 0.00(0.05%)

Bonding orbitals from NBO analysis for ground spin state (Alpha spin orbitals, UNU core) of $[^{Si}L_3UNU^{Si}L_3]^{1-}$, (S=2)

(0.97191) BD (1) U 1- N 5
(16.37%)   0.4046* U 1 s(1.27%)p 0.41(0.52%)d43.27(55.04%) f33.52(42.63%)g 0.42(0.53%)
(83.63%)   0.9145* N 5 s(48.26%)p 1.07(51.71%)d 0.00(0.02%)
(0.97221) BD (1) N 5- U135
(84.19%)   0.9176* N 5 s(51.56%)p 0.94(48.42%)d 0.00(0.02%)
(15.81%)   0.3976* U135 s(3.18%)p 0.20(0.62%)d16.55(52.63%) f13.59(43.23%)g 0.11(0.34%)

Bonding orbitals from NBO analysis for ground spin state (Alpha spin orbitals, USU core) of $[^{Si}L_3USU^{Si}L_3]^{2-}$, (S=3)

(0.98251) BD (1) U 1- S 5
(7.68%)   0.2770* U 1 s(6.60%)p 0.04(0.28%)d11.53(76.07%) f 2.58(17.04%)g 0.00(0.02%)
(92.32%)   0.9609* S 5 s(49.59%)p 1.02(50.39%)d 0.00(0.02%)
(0.92814) BD (2) U 1- S 5
(6.38%)   0.2525* U 1 s(0.52%)p 1.42(0.75%)d99.99(76.50%) f42.35(22.22%)g 0.01(0.01%)
(93.62%)   0.9676* S 5 s(0.02%)p99.99(99.97%)d 0.45(0.01%)
(0.92244) BD (3) U 1- S 5
(5.32%)   0.2307* U 1 s(0.04%)p20.21(0.89%)d99.99(82.08%) f99.99(16.97%)g 0.26(0.01%)
(94.68%)   0.9730* S 5 s(0.01%)p 1.00(99.99%)d 0.00(0.01%)
(0.97046) BD (1) S 5- U135
(91.78%)   0.9580* S 5 s(50.37%)p 0.99(49.61%)d 0.00(0.02%)
(8.22%)   0.2866* U135 s(17.39%)p 0.02(0.39%)d 3.97(69.07%) f 0.75(13.11%)g 0.00(0.04%)

Bonding orbitals from NBO analysis for ground spin state (Alpha spin orbitals, USU core) of $[^{Si}L_3USU^{Si}L_3]^{1-}$, (S=5/2)

(0.96106) BD (1) U 1- S 5
(11.18%)   0.3343* U 1 s(4.12%)p 0.68(2.80%)d11.52(47.42%) f10.95(45.09%)g 0.14(0.57%)
(88.82%)   0.9425* S 5 s(32.11%)p 2.11(67.83%)d 0.00(0.06%)
(0.93761) BD (2) U 1- S 5
(10.93%)   0.3305* U 1 s(0.97%)p 0.42(0.41%)d39.55(38.49%) f61.72(60.07%)g 0.06(0.06%)
(89.07%)   0.9438* S 5 s(0.04%)p99.99(99.93%)d 0.66(0.03%)
(0.95602) BD (1) S 5- U135
(92.56%)   0.9621* S 5 s(67.82%)p 0.47(32.15%)d 0.00(0.03%)
(7.44%)   0.2728* U135 s(15.49%)p 0.02(0.34%)d 3.66(56.72%) f 1.77(27.44%)g 0.00(0.02%)

Bonding orbitals from NBO analysis for ground spin state (Alpha spin orbitals, USU core) of $[^{Si}L_3USU^{Si}L_3]$, (S=2)

(0.96204) BD (1) U 1- S 5
(9.33%)   0.3054* U 1 s(2.26%)p 0.52(1.17%)d22.12(49.94%) f20.58(46.47%)g 0.07(0.16%)
(90.67%)   0.9522* S 5 s(42.88%)p 1.33(57.07%)d 0.00(0.05%)
(0.95509) BD (1) S 5- U135
(90.93%)   0.9536* S 5 s(51.45%)p 0.94(48.50%)d 0.00(0.05%)
(9.07%)   0.3012* U135 s(5.65%)p 0.41(2.31%)d11.15(63.01%) f 5.04(28.46%)g 0.10(0.57%)

DFT computed NBO second order perturbation analysis for UOU core in **$[^{Si}L_3UOU^{Si}L_3]^{2-}$**

Donor NBO	Acceptor NBO	E(2)<br>kcal/mol
(0.91911) LP (1) O 5<br>s(1.47%)p67.21(98.53%)d 0.00(0.00%)	(0.11237) LV (1) U 1<br>s(0.77%)p 0.98(0.75%)d99.99(<br>78.92%)f25.47(19.54%)g 0.03(0.02%)	13.56
(0.91172) LP (2) O 5<br>s(98.17%)p 0.02(1.81%)d 0.00(0.03%)	(0.11237) LV (1) U 1<br>s(0.77%)p 0.98(0.75%)d99.99(<br>78.92%)f25.47(19.54%)g 0.03(0.02%)	35.40
(0.93210) BD (1) O 5- U135<br>(92.07%) 0.9595* O 5 s(0.04%)p99.99(<br>99.96%)d 0.03(0.00%)<br>(7.93%) 0.2817* U135 s(0.01%)p 1.00(<br>0.43%)d99.99(63.90%)f83.13(35.63%)g<br>0.06(0.03%)	(0.09545) LV (3) U 1<br>s(0.13%)p 5.13(0.65%)d99.99(<br>94.56%)f36.86(4.66%)g 0.06(0.01%)	9.71
(0.93113) BD (2) O 5- U135<br>(92.33%) 0.9609* O 5 s(0.30%)p99.99(<br>99.70%)d 0.00(0.00%)<br>(7.67%) 0.2769* U135 s(0.09%)p 6.82(<br>0.58%)d99.99(65.32%)f99.99(33.98%)g<br>0.35(0.03%)	(0.09774) LV (2) U 1<br>s(0.13%)p 3.75(0.48%)d99.99(<br>94.64%)f37.02(4.74%)g 0.04(0.01%)	12.15

DFT computed NBO second order perturbation analysis for UOU core in **$[^{Si}L_3UOU^{Si}L_3]^{1-}$**

Donor NBO	Acceptor NBO	E(2)<br>kcal/mol
(0.92156) LP (1) O 5<br>s(54.84%)p 0.82(45.16%)d 0.00(0.00%)	(0.11318) LV (1) U135<br>s(0.19%)p 3.71(0.69%)d99.99(<br>58.12%)f99.99(40.97%)g 0.16(0.03%)	5.37
(0.92156) LP (1) O 5<br>s(54.84%)p 0.82(45.16%)d 0.00(0.00%)	(0.09342) LV (2) U135<br>s(0.27%)p 2.58(0.70%)d99.99(<br>62.84%)f99.99(36.15%)g 0.12(0.03%)	7.90
(0.92156) LP (1) O 5<br>s(54.84%)p 0.82(45.16%)d 0.00(0.00%)	(0.07832) LV (3) U135<br>s(0.04%)p25.77(0.99%)d99.99(<br>58.82%)f99.99(40.12%)g 0.89(0.03%)	12.13
(0.98237) BD (1) U 1- O 5<br>(10.45%) 0.3233* U 1 s(0.30%)p 3.45(<br>1.04%)d99.99(72.33%) f86.45(26.04%)g<br>0.94(0.28%)<br>(89.55%) 0.9463* O 5 s(45.10%)p 1.22(<br>54.86%)d 0.00(0.04%)	(0.07832) LV (3) U135<br>s(0.04%)p25.77(0.99%)d99.99(<br>58.82%)f99.99(40.12%)g 0.89(0.03%)	3.58
(0.95272) BD (2) U 1- O 5<br>(10.50%) 0.3240* U 1 s(0.10%)p 8.23(<br>0.81%)d99.99(45.24%) f99.99(53.77%)g<br>0.91(0.09%)<br>(89.50%) 0.9460* O 5 s(0.04%)p99.99(<br>99.94%)d 0.29(0.01%)	(0.06266) LV (8) U135<br>s(0.10%)p 2.53(0.25%)d99.99(<br>57.16%)f99.99(42.44%)g 0.45(0.04%)	5.66
(0.94999) BD (3) U 1- O 5<br>(10.42%) 0.3228* U 1 s(0.04%)p11.38(<br>0.47%)d99.99(50.19%) f99.99(49.20%)g<br>2.13(0.09%)<br>(89.58%) 0.9465* O 5 s(0.00%)p 1.00(<br>99.99%)d 0.00(0.01%)	(0.07014) LV (6) U135<br>s(0.17%)p 2.04(0.36%)d99.99(<br>57.31%)f99.99(42.13%)g 0.20(0.03%)	8.17

DFT computed NBO second order perturbation analysis for UOU core in **$[^{Si}L_3UOU^{Si}L_3]$**

Donor NBO	Acceptor NBO	E(2)<br>kcal/mol
(0.85610) LP (1) O 5<br>s(0.14%)p99.99(99.86%)d 0.00(0.00%)	(0.04169) LV (3) U 1<br>s(17.06%)p 0.10(1.64%)d 2.02(34.52%)f<br>2.72(46.43%)g 0.02(0.35%)	4.37
(0.85610) LP (1) O 5<br>s(0.14%)p99.99(99.86%)d 0.00(0.00%)	(0.07796) LV (2) U135	14.27

	s(0.20%)p24.49(4.87%)d99.99(33.88%)f99.99(59.73%)g 6.67(1.33%)	
(0.97777) BD (1) U 1- O 5<br>(8.53%) 0.2921* U 1 s(1.28%)p 0.57(0.73%)d60.55(77.54%) f15.75(20.17%)g 0.23(0.29%)<br>(91.47%) 0.9564* O 5 s(49.67%)p 1.01(50.32%)d 0.00(0.02%)	(0.03761) LV (6) U135<br>s(89.67%)p 0.01(0.69%)d 0.05(4.16%)f 0.06(4.94%)g 0.01(0.54%)	4.65
(0.97207) BD (1) O 5- U135<br>(93.02%) 0.9645* O 5 s(50.11%)p 1.00(49.87%)d 0.00(0.01%)<br>(6.98%) 0.2642* U135 s(1.79%)p 1.43(2.56%)d36.24(64.99%) f15.50(27.80%)g 1.59(2.85%)	(0.03615) LV (4) U 1<br>s(76.56%)p 0.00(0.23%)d 0.14(10.72%)f 0.16(12.44%)g 0.00(0.05%)	3.26

DFT computed NBO second order perturbation analysis for UNU core in **$[^{Si}L_3UNU^{Si}L_3]^{3-}$**

Donor NBO	Acceptor NBO	E(2) kcal/mol
(0.97486) BD (1) U 1- N 5<br>(14.47%) 0.3804* U 1 s(9.21%)p 0.17(1.58%)d 8.43(77.62%) f 1.25(11.54%)g 0.01(0.06%)<br>(85.53%) 0.9248* N 5 s(49.94%)p 1.00(50.04%)d 0.00(0.02%)	(0.05238) LV (3) U135<br>s(86.19%)p 0.01(0.90%)d 0.07(6.10%)f 0.08(6.81%)g 0.00(0.00%)	1.96
(0.97323) BD (1) N 5- U135<br>(86.12%) 0.9280* N 5 s(49.95%)p 1.00(50.04%)d 0.00(0.02%)<br>(13.88%) 0.3726* U135 s(6.38%)p 0.28(1.77%)d13.08(83.39%) f 1.32(8.41%)g 0.01(0.06%)	(0.05712) LV (3) U 1<br>s(81.82%)p 0.01(0.80%)d 0.12(9.91%)f 0.09(7.47%)g 0.00(0.01%)	1.12

DFT computed NBO second order perturbation analysis for UNU core in **$[^{Si}L_3UNU^{Si}L_3]^{2-}$**

Donor NBO	Acceptor NBO	E(2) kcal/mol
(0.91884) BD (2) U 1- N 5<br>(19.69%) 0.4437* U 1 s(0.01%)p 1.00(0.24%)d99.99(52.90%) f99.99(46.83%)g 0.10(0.03%)<br>(80.31%) 0.8962* N 5 s(0.08%)p99.99(99.91%)d 0.20(0.02%)	(0.10979) LV (1) U135<br>s(0.00%)p 1.00(0.40%)d99.99(87.08%)f31.24(12.51%)g 0.01(0.00%)	3.49
(0.91884) BD (2) U 1- N 5<br>(19.69%) 0.4437* U 1 s(0.01%)p 1.00(0.24%)d99.99(52.90%) f99.99(46.83%)g 0.10(0.03%)<br>(80.31%) 0.8962* N 5 s(0.08%)p99.99(99.91%)d 0.20(0.02%)	(0.10315) LV (2) U135<br>s(0.00%)p 1.00(0.47%)d99.99(86.08%)f28.74(13.44%)g 0.01(0.01%)	8.97
(0.91461) BD (3) U 1- N 5<br>(19.04%) 0.4363* U 1 s(0.01%)p 1.00(0.29%)d99.99(54.10%) f99.99(45.58%)g 0.10(0.03%)<br>(80.96%) 0.8998* N 5 s(0.03%)p99.99(99.95%)d 0.48(0.02%)	(0.10979) LV (1) U135<br>s(0.00%)p 1.00(0.40%)d99.99(87.08%)f31.24(12.51%)g 0.01(0.00%)	10.77
(0.96932) BD (1) N 5- U135<br>(88.21%) 0.9392* N 5 s(59.43%)p 0.68(40.56%)d 0.00(0.01%)<br>(11.79%) 0.3433* U135 s(12.74%)p 0.07(0.84%)d 6.23(79.38%) f 0.55(6.99%)g 0.00(0.05%)	(0.10879) LV (1) U 1<br>s(6.69%)p 0.10(0.65%)d 8.91(59.67%)f 4.93(32.98%)g 0.00(0.01%)	3.68
(0.96932) BD (1) N 5- U135<br>(88.21%) 0.9392* N 5 s(59.43%)p 0.68(40.56%)d 0.00(0.01%)	(0.09140) LV (3) U 1<br>s(10.01%)p 0.09(0.88%)d 6.40(64.02%)f 2.51(25.08%)g 0.00(0.01%)	3.60

(11.79%) 0.3433* U135 s(12.74%)p 0.07(0.84%)d 6.23(79.38%) f 0.55(6.99%)g 0.00(0.05%)		

DFT computed NBO second order perturbation analysis for UNU core in **[^{Si}L$_3$UNU^{Si}L$_3$]$^{1-}$**

Donor NBO	Acceptor NBO	E(2) kcal/mol
(0.73817) LP (2) N 5 s(0.04%)p99.99(99.96%)d 0.01(0.00%)	(0.09355) LV (1) U 1 s(1.89%)p 0.61(1.14%)d24.63(46.53%)f26.44(49.95%)g 0.26(0.49%)	8.06
(0.73817) LP (2) N 5 s(0.04%)p99.99(99.96%)d 0.01(0.00%)	(0.09152) LV (2) U 1 s(1.10%)p 1.25(1.38%)d37.63(41.38%)f50.72(55.78%)g 0.33(0.37%)	5.26
(0.73817) LP (2) N 5 s(0.04%)p99.99(99.96%)d 0.01(0.00%)	(0.09322) LV (1) U135 s(0.03%)p14.73(0.48%)d99.99(42.57%)f99.99(56.82%)g 3.10(0.10%)	10.73
(0.97191) BD (1) U 1- N 5 (16.37%) 0.4046* U 1 s(1.27%)p 0.41(0.52%)d43.27(55.04%) f33.52(42.63%)g 0.42(0.53%) (83.63%) 0.9145* N 5 s(48.26%)p 1.07(51.71%)d 0.00(0.02%)	(0.03325) LV (5) U135 s(76.92%)p 0.00(0.17%)d 0.11(8.12%)f 0.19(14.52%)g 0.00(0.26%)	6.41
(0.97221) BD (1) N 5- U135 (84.19%) 0.9176* N 5 s(51.56%)p 0.94(48.42%)d 0.00(0.02%) (15.81%) 0.3976* U135 s(3.18%)p 0.20(0.62%)d16.55(52.63%) f13.59(43.23%)g 0.11(0.34%)	(0.03516) LV (6) U 1 s(83.61%)p 0.01(0.57%)d 0.08(6.32%)f 0.11(9.06%)g 0.01(0.44%)	5.72

DFT computed NBO second order perturbation analysis for USU core in **[^{Si}L$_3$USU^{Si}L$_3$]$^{2-}$**

Donor NBO	Acceptor NBO	E(2) kcal/mol
(0.92814) BD (2) U 1- S 5 (6.38%) 0.2525* U 1 s(0.52%)p 1.42(0.75%)d99.99(76.50%) f42.35(22.22%)g 0.01(0.01%) (93.62%) 0.9676* S 5 s(0.02%)p99.99(99.97%)d 0.45(0.01%)	(0.07973) LV (4) U135 s(14.28%)p 0.05(0.65%)d 4.18(59.70%)f 1.78(25.35%)g 0.00(0.02%)	5.28
(0.92814) BD (2) U 1- S 5 (6.38%) 0.2525* U 1 s(0.52%)p 1.42(0.75%)d99.99(76.50%) f42.35(22.22%)g 0.01(0.01%) (93.62%) 0.9676* S 5 s(0.02%)p99.99(99.97%)d 0.45(0.01%)	(0.05457) LV (6) U135 s(10.48%)p 0.02(0.22%)d 3.42(35.89%)f 5.09(53.38%)g 0.00(0.02%)	2.51
(0.92244) BD (3) U 1- S 5 (5.32%) 0.2307* U 1 s(0.04%)p20.21(0.89%)d99.99(82.08%) f99.99(16.97%)g 0.26(0.01%) (94.68%) 0.9730* S 5 s(0.01%)p 1.00(99.99%)d 0.00(0.01%)	(0.09935) LV (1) U135 s(0.12%)p 6.63(0.79%)d99.99(88.23%)f90.67(10.85%)g 0.13(0.02%)	7.56
(0.92244) BD (3) U 1- S 5 (5.32%) 0.2307* U 1 s(0.04%)p20.21(0.89%)d99.99(82.08%) f99.99(16.97%)g 0.26(0.01%) (94.68%) 0.9730* S 5 s(0.01%)p 1.00(99.99%)d 0.00(0.01%)	(0.07973) LV (4) U135 s(14.28%)p 0.05(0.65%)d 4.18(59.70%)f 1.78(25.35%)g 0.00(0.02%)	2.65
(0.97046) BD (1) S 5- U135 (91.78%) 0.9580* S 5 s(50.37%)p 0.99(49.61%)d 0.00(0.02%)	(0.04261) LV (5) U 1 s(49.65%)p 0.01(0.36%)d 0.23(11.45%)f 0.78(38.52%)g 0.00(0.02%)	2.68

(8.22%) 0.2866* U135 s(17.39%)p<br>0.02(0.39%)d 3.97(69.07%) f 0.75(<br>13.11%)g 0.00(0.04%)		

DFT computed NBO second order perturbation analysis for USU core in $\mathbf{[^{Si}L_3USU^{Si}L_3]^{1-}}$

Donor NBO	Acceptor NBO	E(2)<br>kcal/mol
(0.84703) LP (1) S 5<br>s(0.02%)p99.99(99.94%)d 2.74(0.04%)	(0.09499) LV (1) U135<br>s(0.19%)p 2.76(0.51%)d99.99(<br>90.85%)f45.42(8.44%)g 0.06(0.01%)	10.56
(0.96106) BD (1) U 1- S 5<br>(11.18%) 0.3343* U 1 s(4.12%)p 0.68(<br>2.80%)d11.52(47.42%) f10.95(45.09%)g<br>0.14(0.57%)<br>(88.82%) 0.9425* S 5 s(32.11%)p 2.11(<br>67.83%)d 0.00(0.06%)	(0.04759) LV (7) U135<br>s(58.34%)p 0.01(0.49%)d 0.32(18.73%)f<br>0.38(22.42%)g 0.00(0.03%)	6.72
(0.96106) BD (1) U 1- S 5<br>(11.18%) 0.3343* U 1 s(4.12%)p 0.68(<br>2.80%)d11.52(47.42%) f10.95(45.09%)g<br>0.14(0.57%)<br>(88.82%) 0.9425* S 5 s(32.11%)p 2.11(<br>67.83%)d 0.00(0.06%)	(0.04499) LV (8) U135<br>s(15.35%)p 0.02(0.34%)d 1.44(22.16%)f<br>4.05(62.12%)g 0.00(0.04%)	3.00
(0.95602) BD (1) S 5- U135<br>(92.56%) 0.9621* S 5 s(67.82%)p 0.47(<br>32.15%)d 0.00(0.03%)<br>(7.44%) 0.2728* U135 s(15.49%)p<br>0.02(0.34%)d 3.66(56.72%) f 1.77(<br>27.44%)g 0.00(0.02%)	(0.04087) LV (7) U 1<br>s(84.68%)p 0.08(6.41%)d 0.02(1.53%)f<br>0.08(7.10%)g 0.00(0.28%)	5.07

DFT computed NBO second order perturbation analysis for USU core in $\mathbf{[^{Si}L_3USU^{Si}L_3]}$

Donor NBO	Acceptor NBO	E(2)<br>kcal/mol
(0.83467) LP (1) S 5<br>s(3.48%)p27.76(96.49%)d 0.01(0.03%)	(0.08661) LV (3) U135<br>s(2.66%)p 0.54(1.43%)d14.21(<br>37.77%)f21.72(57.73%)g 0.16(0.41%)	2.81
(0.81677) LP (2) S 5<br>s(2.18%)p44.79(97.78%)d 0.02(0.04%)	(0.10525) LV (1) U 1<br>s(0.37%)p 0.74(0.27%)d99.99(<br>44.96%)f99.99(54.30%)g 0.26(0.10%)	7.59
(0.96204) BD (1) U 1- S 5<br>(9.33%) 0.3054* U 1 s(2.26%)p 0.52(<br>1.17%)d22.12(49.94%) f20.58(46.47%)g<br>0.07(0.16%)<br>(90.67%) 0.9522* S 5 s(42.88%)p 1.33(<br>57.07%)d 0.00(0.05%)	(0.03816) LV (5) U135<br>s(72.42%)p 0.03(2.24%)d 0.12(8.78%)f<br>0.22(16.25%)g 0.00(0.30%)	6.09
(0.95509) BD (1) S 5- U135<br>(90.93%) 0.9536* S 5 s(51.45%)p 0.94(<br>48.50%)d 0.00(0.05%)<br>(9.07%) 0.3012* U135 s(5.65%)p 0.41(<br>2.31%)d11.15(63.01%) f 5.04(28.46%)g<br>0.10(0.57%)	(0.03844) LV (5) U 1<br>s(89.45%)p 0.01(1.33%)d 0.03(2.61%)f<br>0.07(6.57%)g 0.00(0.04%)	6.38

DFT computed MO’s (Alpha spin orbitals) for ground state spin

	$[^{Si}L_3UOU^{Si}L_3]^{2-}$<br>S=3	$[^{Si}L_3UOU^{Si}L_3]^{1-}$<br>S=5/2	$[^{Si}L_3UOU^{Si}L_3]$<br>S=2
HOMO-5	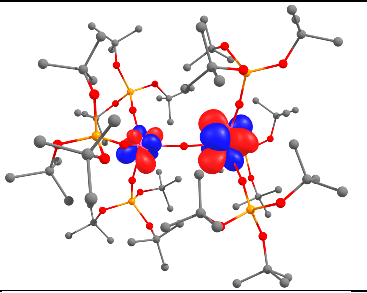		
HOMO-4	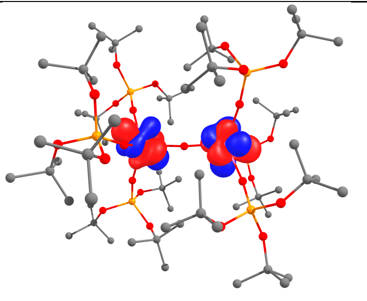	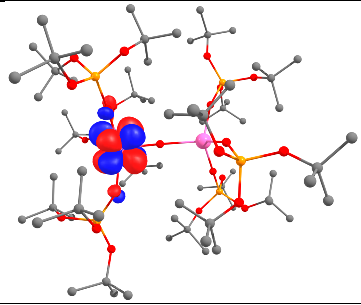	
HOMO-3	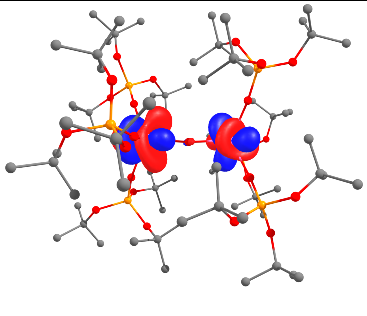	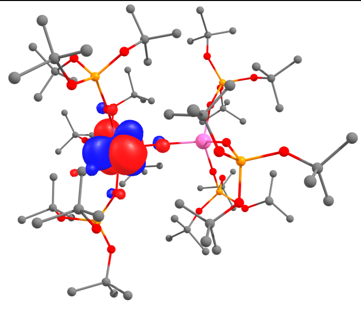	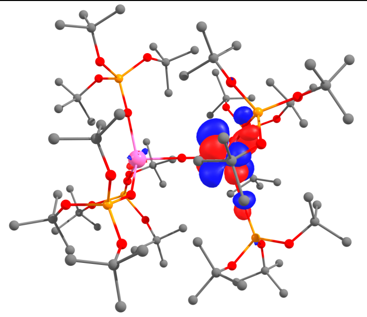
HOMO-2	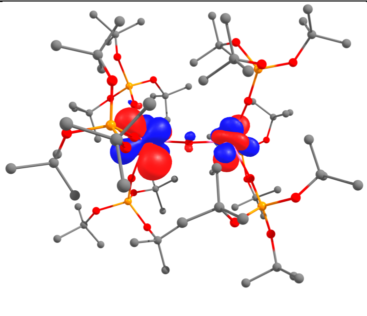	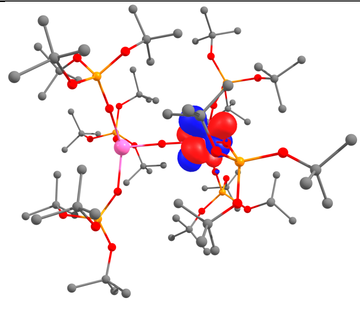	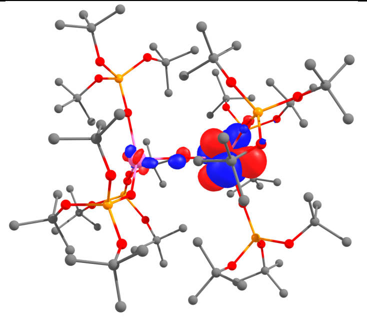
HOMO-1	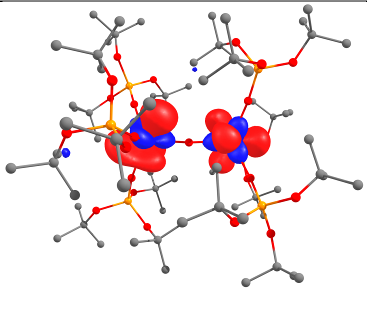	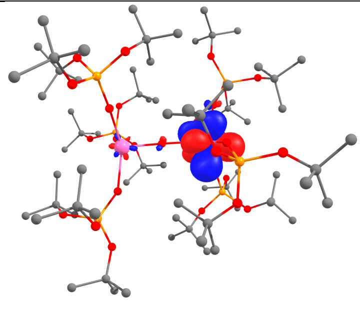	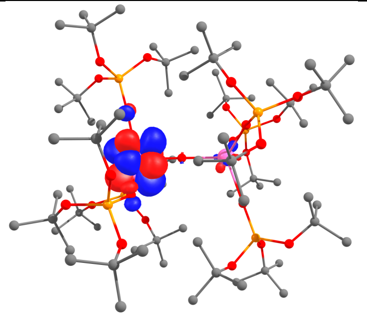

HOMO	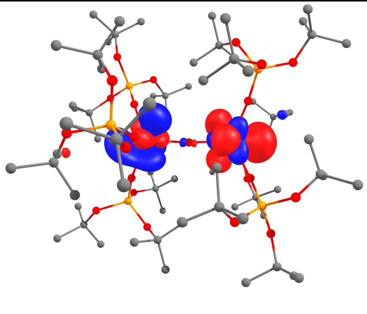	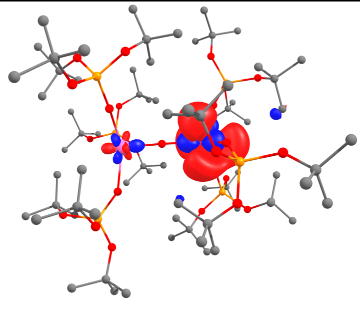	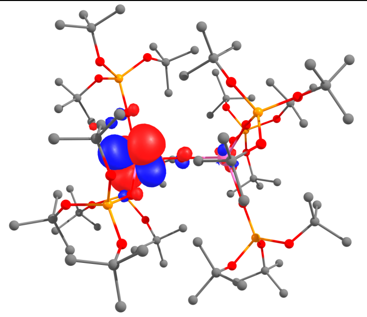
LUMO	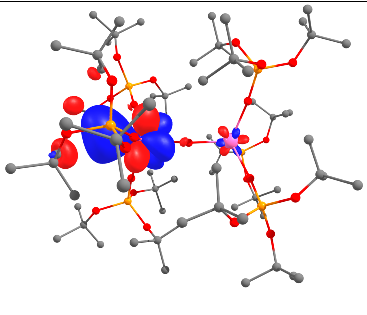	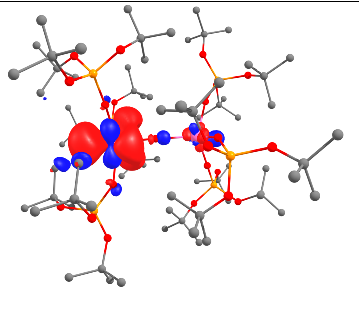	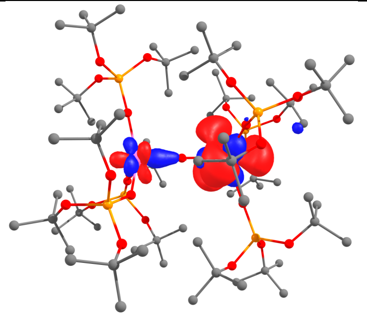

	[$^{Si}L_3UNU^{Si}L_3$]$^{3-}$<br>S=3	[$^{Si}L_3UNU^{Si}L_3$]$^{2-}$<br>S=5/2	[$^{Si}L_3UNU^{Si}L_3$]$^{1-}$<br>S=2
HOMO-5	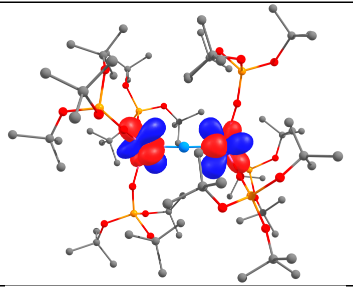		
HOMO-4	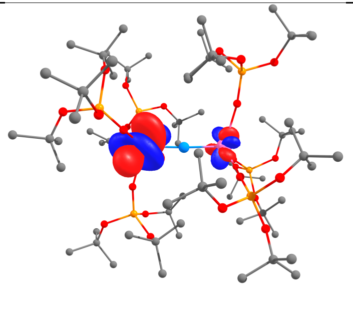	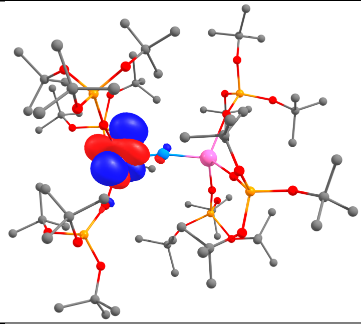	
HOMO-3	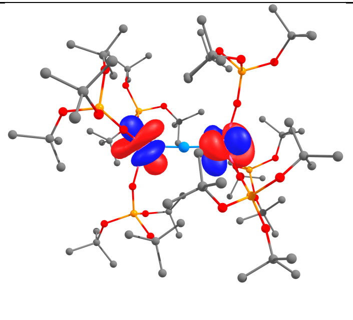	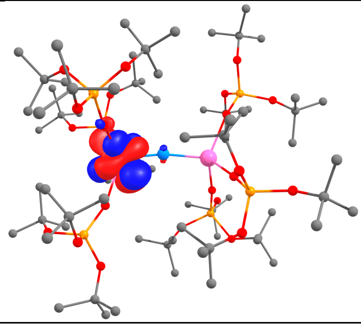	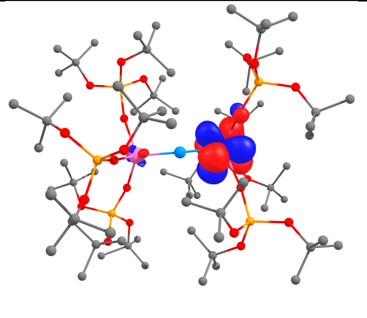

HOMO-2	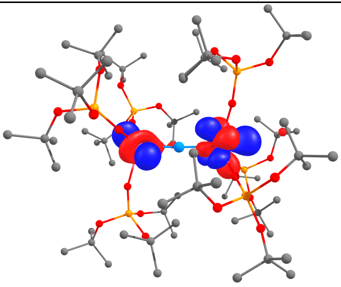	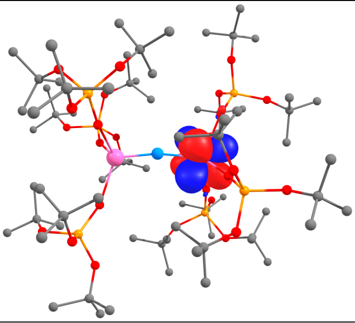	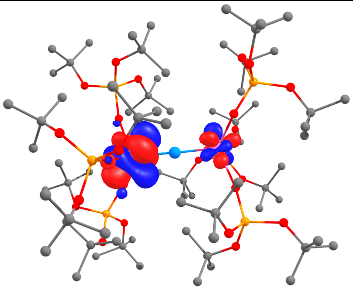
HOMO-1	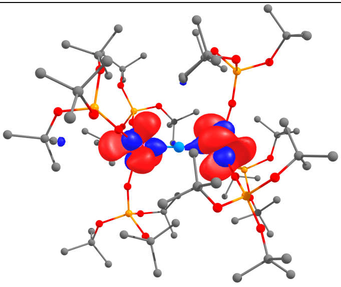	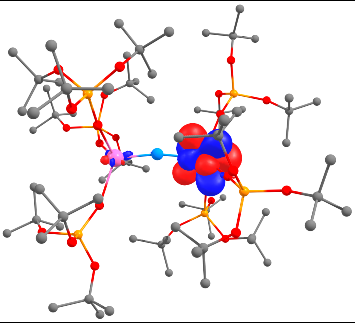	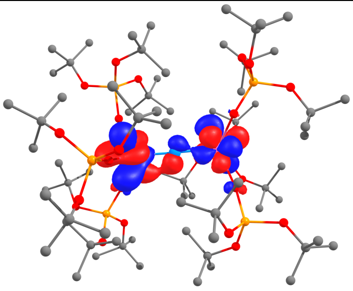
HOMO	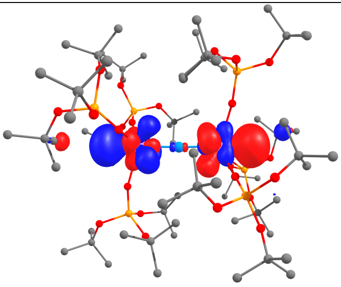	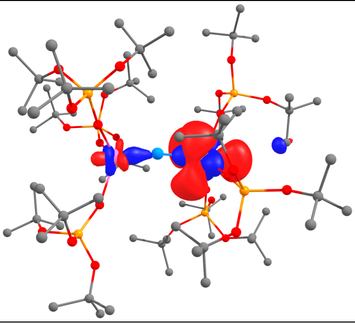	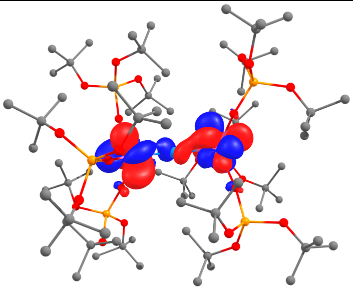
LUMO	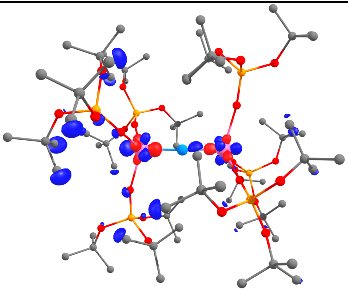	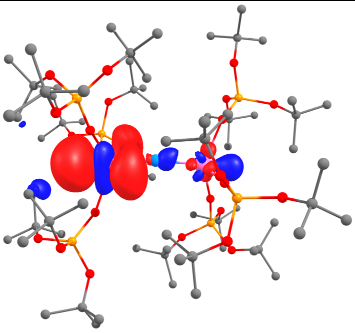	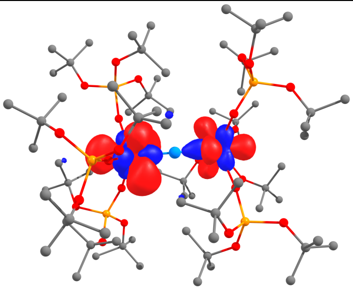
	**[$^{Si}L_3USU^{Si}L_3$]$^{2-}$**<br>S=3	**[$^{Si}L_3USU^{Si}L_3$]$^{1-}$**<br>S=5/2	**[$^{Si}L_3USU^{Si}L_3$]**<br>S=2
HOMO-5	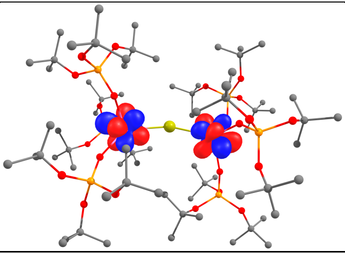		
HOMO-4	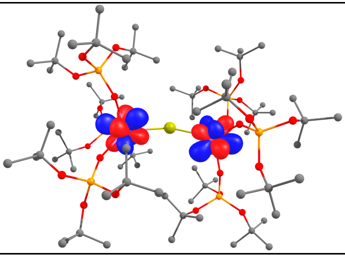	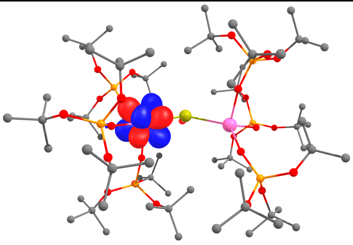	

HOMO-3	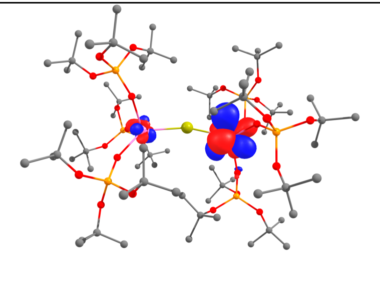	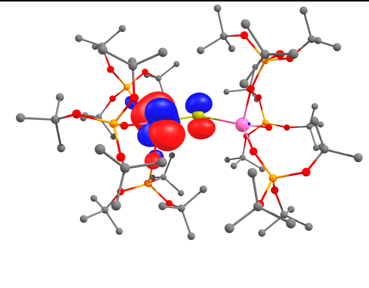	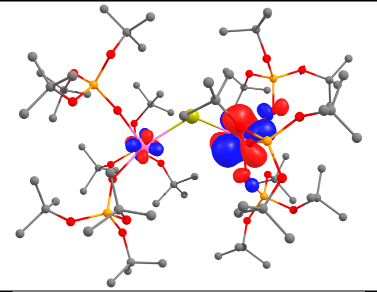
HOMO-2	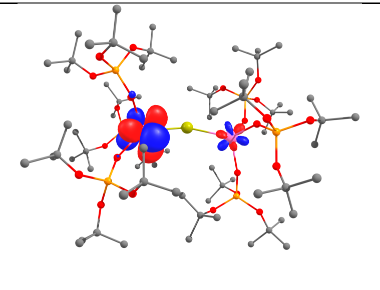	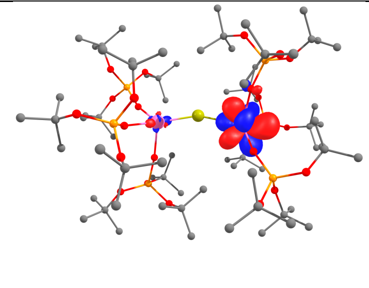	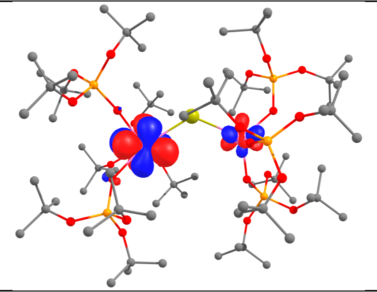
HOMO-1	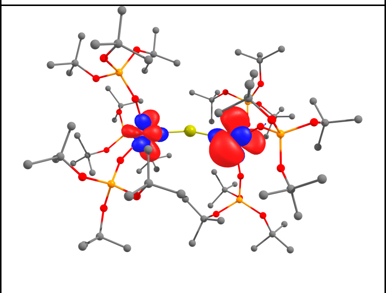	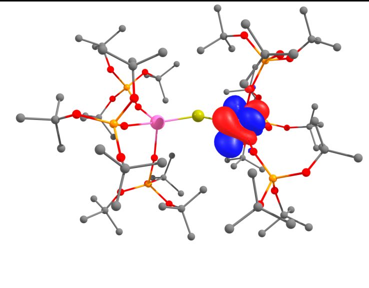	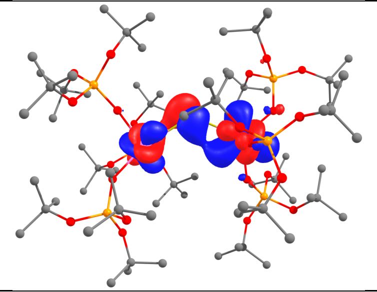
HOMO	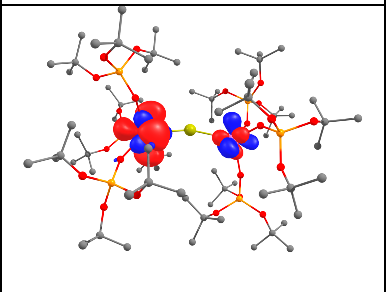	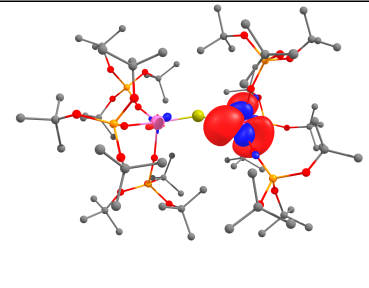	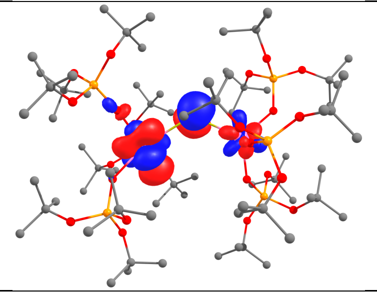
LUMO	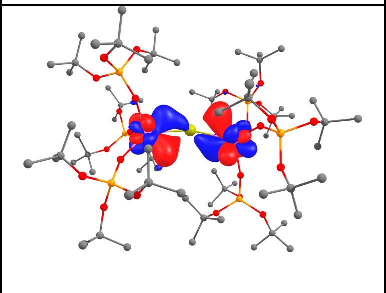	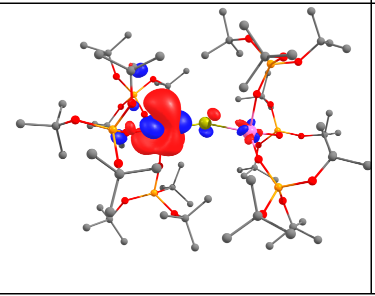	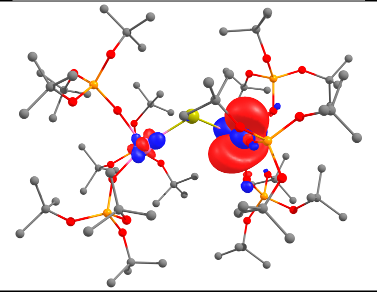

Computed natural charges for **[$^{Ar}L_3UOU^{Ar}L_3$]$^{2-}$**, **[$^{Ar}L_3UOU^{Ar}L_3$]$^{1-}$**, **[$^{Ar}L_3UNU^{Ar}L_3$]$^{3-}$**, **[$^{Ar}L_3UNU^{Ar}L_3$]$^{2-}$**, **[$^{Ar}L_3USU^{Ar}L_3$]$^{2-}$**, **[$^{Ar}L_3USU^{Ar}L_3$]$^{1-}$**

	Natural charges								
Atom label	**[UOU]$^{2-}$** S=3	**[UOU]$^{1-}$** S=5/2	**[UOU]** S=2	**[UNU]$^{3-}$** S=3	**[UNU]$^{2-}$** S=5/2	**[UNU]$^{1-}$** S=2	**[USU]$^{2-}$** S=3	**[USU]$^{1-}$** S=5/2	**[USU]** S=2
U1	1.68732	1.98126	2.03408	1.50285	1.78660	1.90865	1.50125	1.64898	1.73752
O2	-0.81819	-0.78771	-0.78200	-0.79391	-0.78740	-0.78180	-0.81093	-0.75302	-0.74989
O3	-0.81828	-0.78733	-0.78062	-0.79516	-0.78629	-0.78117	-0.81204	-0.75164	-0.74923
O4	-0.82491	-0.79423	-0.78996	-0.79779	-0.78851	-0.78976	-0.82267	-0.78092	-0.78255
O5/N5/S5	-1.14755	-1.06431	-1.04504	-1.50820	-1.38416	-1.34296	-0.89945	-0.73229	-0.66862
U111	1.68737	1.76429	2.03940	1.50252	1.63459	1.90365	1.48462	1.57674	1.74692
O112	-0.81821	-0.82814	-0.78349	-0.79447	-0.80944	-0.78594	-0.80470	-0.81442	-0.75104
O113	-0.81831	-0.82709	-0.78371	-0.79203	-0.80922	-0.78690	-0.80431	-0.81545	-0.75246
O114	-0.82492	-0.83512	-0.78899	-0.79934	-0.81463	-0.78909	-0.82033	-0.83263	-0.78495

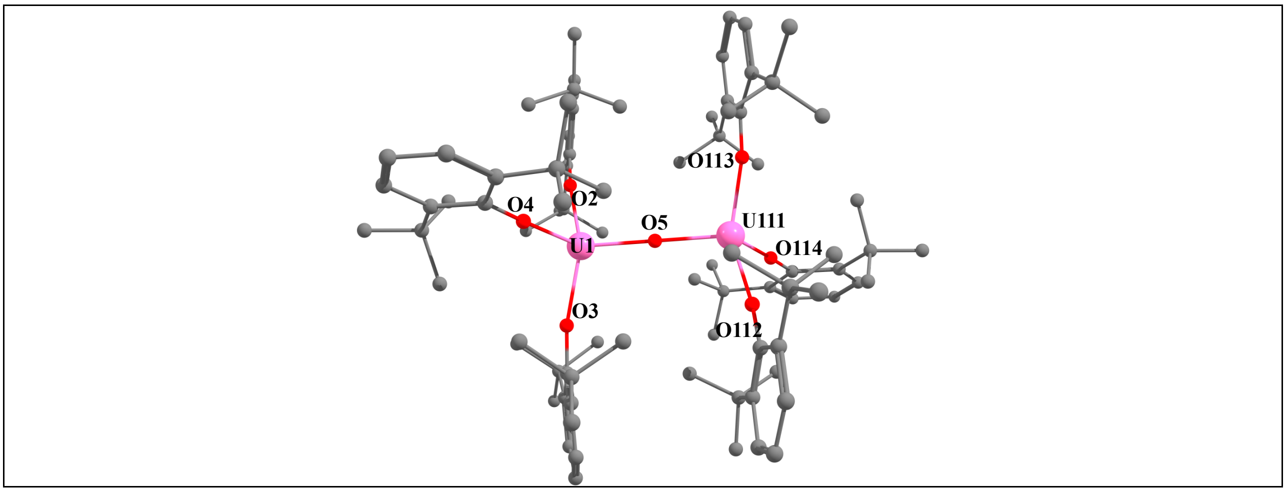

DFT computed spin densities for selected atoms in **$[^{Ar}L_3UOU^{Ar}L_3]^{2-}$**, **$[^{Ar}L_3UOU^{Ar}L_3]^{1-}$**, **$[^{Ar}L_3UNU^{Ar}L_3]^{3-}$**, **$[^{Ar}L_3UNU^{Ar}L_3]^{2-}$**, **$[^{Ar}L_3USU^{Ar}L_3]^{2-}$**, **$[^{Ar}L_3USU^{Ar}L_3]^{1-}$**

Atom label	[UOU]$^{2-}$ S=3	[UOU]$^{1-}$ S=5/2	[UOU] S=2	[UNU]$^{3-}$ S=3	[UNU]$^{2-}$ S=5/2	[UNU]$^{1-}$ S=2	[USU]$^{2-}$ S=3	[USU]$^{1-}$ S=5/2	[USU] S=2
U1	3.08	2.14	2.12	3.10	2.16	2.13	3.13	2.2	2.16
O2	-0.02	-0.02	-0.03	-0.02	-0.01	-0.02	-0.02	-0.03	-0.03
O3	-0.02	-0.02	-0.03	-0.02	-0.01	-0.02	-0.02	-0.03	-0.04
O4	-0.02	-0.02	-0.02	-0.02	-0.02	-0.02	-0.03	-0.02	-0.02
O5/N5/S5	-0.10	-0.08	-0.06	-0.19	-0.17	-0.14	-0.17	-0.16	-0.10
U111	3.08	3.06	2.12	3.10	3.07	2.13	3.12	3.06	2.16
O112	-0.02	-0.02	-0.03	-0.02	-0.02	-0.02	-0.02	-0.02	-0.04
O113	-0.02	-0.02	-0.02	-0.02	-0.02	-0.02	-0.02	-0.02	-0.03
O114	-0.02	-0.03	-0.02	-0.02	-0.02	-0.02	-0.03	-0.03	-0.02

Computed Wiberg bond index for ground spin state of **$[^{Ar}L_3UOU^{Ar}L_3]^{2-}$**, **$[^{Ar}L_3UOU^{Ar}L_3]^{1-}$**, **$[^{Ar}L_3UOU^{Ar}L_3]$**, **$[^{Ar}L_3UNU^{Ar}L_3]^{3-}$**, **$[^{Ar}L_3UNU^{Ar}L_3]^{2-}$**, **$[^{Ar}L_3UNU^{Ar}L_3]^{1-}$**, **$[^{Ar}L_3USU^{Ar}L_3]^{2-}$**, **$[^{Ar}L_3USU^{Ar}L_3]^{1-}$**, **$[^{Ar}L_3USU^{Ar}L_3]$**

Atom label	Wiberg bond index	Atom label	Wiberg bond index	Atom label	Wiberg bond index	Atom label	Wiberg bond index
U1	0.0000	U1	0.0000	U1	0.0000	U1	0.0000
O2	0.4607<br>0.6366<br>0.7111<br>0.4293<br>0.5254<br>0.6254<br>0.4936<br>0.7356<br>0.7806	O3	0.4581<br>0.6443<br>0.7140<br>0.4215<br>0.5276<br>0.6259<br>0.4898<br>0.7434<br>0.7882	O4	0.4893<br>0.6672<br>0.7206<br>0.4337<br>0.5655<br>0.6371<br>0.5203<br>0.7061<br>0.7328	O5/N5/S5	0.7074<br>1.0663<br>0.7877<br>1.0995<br>1.6119<br>1.2155<br>0.8584<br>1.2982<br>1.0344
Atom label	Wiberg bond index	Atom label	Wiberg bond index	Atom label	Wiberg bond index	Atom label	Wiberg bond index
U111	0.0000	U111	0.0000	U111	0.0000	U111	0.0000
O5/N5/S5	0.7074<br>0.4818<br>0.7847<br>1.1095<br>0.7815<br>1.2327<br>0.8861<br>0.6679<br>1.0071	O112	0.4607<br>0.4978<br>0.7093<br>0.4207<br>0.4622<br>0.6067<br>0.4919<br>0.5076<br>0.8892	O113	0.4581<br>0.4941<br>0.7098<br>0.4145<br>0.4631<br>0.6005<br>0.4869<br>0.5083<br>0.8854	O114	0.4893<br>0.5212<br>0.7211<br>0.4400<br>0.4761<br>0.6352<br>0.5281<br>0.5501<br>0.8560

Bonding orbitals from NBO analysis for ground spin state (Alpha spin orbitals, UOU core) of $[^{Ar}L_3UOU^{Ar}L_3]^{2-}$, (S=3)

(0.92436) BD (1) U 1- O 5
(7.65%) 0.2766* U 1 s(0.05%)p 8.95(0.41%)d99.99(65.46%)f99.99(34.05%)g 0.53(0.02%)
(92.35%) 0.9610* O 5 s(0.00%)p 1.00(100.00%)d 0.00(0.00%)
(0.95250) BD (1) O 5- U111
(94.61%) 0.9727* O 5 s(99.95%)p 0.00(0.02%)d 0.00(0.02%)
(5.39%) 0.2321* U111 s(7.99%)p 0.04(0.33%)d 9.50(75.86%)f 1.98(15.80%)g 0.00(0.03%)
(0.92377) BD (2) O 5- U111
(92.53%) 0.9619* O 5 s(0.02%)p99.99(99.98%)d 0.00(0.00%)
(7.47%) 0.2734* U111 s(0.01%)p 1.00(0.38%)d99.99(62.33%)f99.21(37.26%)g 0.06(0.02%)

Bonding orbitals from NBO analysis for ground spin state (Alpha spin orbitals, UOU core) of $[^{Ar}L_3UOU^{Ar}L_3]^{1-}$, (S=5/2)

(0.98841) BD (1) U 1- O 5
(10.68%) 0.3269* U 1 s(0.18%)p 4.11(0.76%)d99.99(46.70%)f99.99(52.25%)g 0.60(0.11%)
(89.32%) 0.9451* O 5 s(46.82%)p 1.13(53.14%)d 0.00(0.04%)
(0.94954) BD (2) U 1- O 5
(10.69%) 0.3269* U 1 s(0.07%)p 4.71(0.34%)d99.99(54.23%)f99.99(45.27%)g 1.18(0.09%)
(89.31%) 0.9451* O 5 s(0.03%)p99.99(99.96%)d 0.62(0.02%)
(0.94697) BD (3) U 1- O 5
(10.47%) 0.3236* U 1 s(0.01%)p 1.00(0.32%)d99.99(55.25%)f99.99(44.34%)g 0.27(0.08%)
(89.53%) 0.9462* O 5 s(0.00%)p 1.00(99.98%)d 0.00(0.01%)
(0.97879) BD (1) O 5- U111
(82.72%) 0.9095* O 5 s(53.11%)p 0.88(46.89%)d 0.00(0.01%)
(17.28%) 0.4156* U111 s(0.15%)p 0.60(0.09%)d99.99(21.93%)f99.99(77.81%)g 0.14(0.02%)

Bonding orbitals from NBO analysis for ground spin state (Alpha spin orbitals, UOU core) of $[^{Ar}L_3UOU^{Ar}L_3]$, (S=2)

(0.98350) BD (1) U 1- O 5
(8.90%) 0.2984* U 1 s(0.56%)p 0.66(0.37%)d95.00(53.56%) f80.56(45.42%)g 0.14(0.08%)
(91.10%) 0.9544* O 5 s(50.21%)p 0.99(49.77%)d 0.00(0.01%)
(0.98378) BD (1) O 5- U111
(91.03%) 0.9541* O 5 s(49.76%)p 1.01(50.23%)d 0.00(0.01%)
(8.97%) 0.2994* U111 s(0.49%)p 0.74(0.36%)d99.99(53.90%) f92.05(45.18%)g 0.15(0.07%)

Bonding orbitals from NBO analysis for ground spin state (Alpha spin orbitals, UNU core) of $[^{Ar}L_3UNU^{Ar}L_3]^{3-}$, (S=3)

(0.97205) BD (1) U 1- N 5
(13.36%) 0.3654* U 1 s(7.60%)p 0.18(1.36%)d 9.66(73.36%)f 2.32(17.62%)g 0.01(0.06%)
(86.64%) 0.9308* N 5 s(49.92%)p 1.00(50.07%)d 0.00(0.01%)
(0.86875) BD (2) U 1- N 5
(13.87%) 0.3725* U 1 s(0.41%)p 0.78(0.32%)d99.99(66.88%)f78.08(32.37%)g 0.04(0.02%)
(86.13%) 0.9280* N 5 s(1.80%)p54.61(98.20%)d 0.00(0.00%)
(0.96806) BD (1) N 5- U111
(86.46%) 0.9298* N 5 s(47.99%)p 1.08(52.00%)d 0.00(0.02%)
(13.54%) 0.3679* U111 s(8.16%)p 0.21(1.75%)d 9.72(79.25%)f 1.32(10.78%)g 0.01(0.06%)
(0.86833) BD (2) N 5- U111
(86.19%) 0.9284* N 5 s(0.27%)p99.99(99.73%)d 0.00(0.00%)
(13.81%) 0.3716* U111 s(0.06%)p 4.62(0.29%)d99.99(65.15%)f99.99(34.49%)g 0.24(0.01%)

Bonding orbitals from NBO analysis for ground spin state (Alpha spin orbitals, UNU core) of $[^{Ar}L_3UNU^{Ar}L_3]^{2-}$, (S=5/2)

(0.98454) BD (1) U 1- N 5
(20.47%) 0.4525* U 1 s(0.26%)p 2.62(0.68%)d99.99(50.41%)f99.99(48.60%)g 0.18(0.05%)
(79.53%) 0.8918* N 5 s(42.39%)p 1.36(57.56%)d 0.00(0.06%)
(0.91792) BD (2) U 1- N 5
(19.93%) 0.4465* U 1 s(0.01%)p 1.00(0.11%)d99.99(54.40%)f99.99(45.45%)g 0.23(0.03%)
(80.07%) 0.8948* N 5 s(0.12%)p99.99(99.86%)d 0.14(0.02%)
(0.91604) BD (3) U 1- N 5
(19.61%) 0.4429* U 1 s(0.00%)p 1.00(0.14%)d99.99(51.82%)f99.99(48.01%)g 0.17(0.02%)
(80.39%) 0.8966* N 5 s(0.04%)p99.99(99.95%)d 0.44(0.02%)
(0.96509) BD (1) N 5- U111

(88.66%)   0.9416* N 5 s(57.48%)p 0.74(42.52%)d 0.00(0.00%)
(11.34%)   0.3368* U111 s(11.11%)p 0.09(0.97%)d 7.09(78.77%)f 0.82(9.10%)g 0.00(0.05%)

Bonding orbitals from NBO analysis for ground spin state (Alpha spin orbitals, UNU core) of [$^{Ar}L_3UNU^{Ar}L_3]^-$, (S=2)
(0.96964) BD (1) U 1- N 5
(14.08%)   0.3752* U 1 s(5.93%)p 0.16(0.94%)d10.71(63.57%) f 4.97(29.49%)g 0.01(0.07%)
(85.92%)   0.9270* N 5 s(54.15%)p 0.85(45.84%)d 0.00(0.01%)
(0.98035) BD (1) N 5- U111
(82.71%)   0.9094* N 5 s(45.68%)p 1.19(54.29%)d 0.00(0.02%)
(17.29%)   0.4159* U111 s(0.67%)p 0.73(0.49%)d78.10(52.45%) f69.00(46.34%)g 0.07(0.05%)

Bonding orbitals from NBO analysis for ground spin state (Alpha spin orbitals, USU core) of [$^{Ar}L_3USU^{Ar}L_3]^{2-}$, (S=3)
(0.97918) BD (1) U 1- S 5
(11.14%)   0.3338* U 1 s(19.02%)p 0.03(0.54%)d 3.15(59.90%)f 1.08(20.51%)g 0.00(0.02%)
(88.86%)   0.9426* S 5 s(50.37%)p 0.99(49.62%)d 0.00(0.02%)
(0.97792) BD (1) S 5- U111
(88.92%)   0.9430* S 5 s(49.54%)p 1.02(50.44%)d 0.00(0.01%)
(11.08%)   0.3329* U111 s(25.75%)p 0.04(0.91%)d 2.53(65.22%)f 0.31(8.09%)g 0.00(0.03%)

Bonding orbitals from NBO analysis for ground spin state (Alpha spin orbitals, USU core) of [$^{Ar}L_3USU^{Ar}L_3]^{1-}$, (S=5/2)
(0.98212) BD (1) U 1- S 5
(15.83%)   0.3979* U 1 s(5.64%)p 0.07(0.38%)d12.83(72.42%)f 3.82(21.55%)g 0.00(0.02%)
(84.17%)   0.9174* S 5 s(45.81%)p 1.18(54.16%)d 0.00(0.03%)
(0.94026) BD (2) U 1- S 5
(14.49%)   0.3807* U 1 s(0.03%)p 9.37(0.26%)d99.99(59.00%)f99.99(40.71%)g 0.26(0.01%)
(85.51%)   0.9247* S 5 s(0.02%)p99.99(99.97%)d 0.61(0.01%)
(0.93534) BD (3) U 1- S 5
(13.13%)   0.3623* U 1 s(0.07%)p 2.18(0.15%)d99.99(44.38%)f99.99(55.38%)g 0.20(0.01%)
(86.87%)   0.9321* S 5 s(0.22%)p99.99(99.78%)d 0.03(0.01%)
(0.97899) BD (1) S 5- U111
(89.82%)   0.9477* S 5 s(53.93%)p 0.85(46.06%)d 0.00(0.01%)
(10.18%)   0.3191* U111 s(12.01%)p 0.03(0.42%)d 6.75(81.04%)f 0.54(6.51%)g 0.00(0.01%)

Bonding orbitals from NBO analysis for ground spin state (Alpha spin orbitals, USU core) of [$^{Ar}L_3USU^{Ar}L_3$], (S=2)
(0.97062) BD (1) U 1- S 5
(13.41%)   0.3662* U 1 s(20.48%)p 0.02(0.50%)d 3.07(62.96%) f 0.78(16.01%)g 0.00(0.05%)
(86.59%)   0.9305* S 5 s(49.97%)p 1.00(50.01%)d 0.00(0.03%)
(0.97243) BD (1) S 5- U111
(86.47%)   0.9299* S 5 s(49.65%)p 1.01(50.32%)d 0.00(0.02%)
(13.53%)   0.3678* U111 s(11.51%)p 0.03(0.40%)d 6.56(75.54%) f 1.09(12.52%)g 0.00(0.03%)

DFT computed NBO second order perturbation analysis for UOU core in **[$^{Ar}L_3UOU^{Ar}L_3]^{2-}$**

Donor NBO	Acceptor NBO	E(2) kcal/mol
(0.92966) LP (1) O 5<br>s(0.00%)p 1.00(100.00%)d 0.00(0.00%)	(0.10854) LV (1) U 1<br>s(2.02%)p 0.46(0.92%)d44.28(89.65%)f 3.64(7.38%)g 0.01(0.02%)	4.47
(0.92436) BD (1) U 1- O 5<br>(7.65%) 0.2766* U 1 s(0.05%)p 8.95(0.41%)d99.99(65.46%)f99.99(34.05%)g 0.53(0.02%)<br>(92.35%) 0.9610* O 5 s(0.00%)p 1.00(100.00%)d 0.00(0.00%)	(0.09728) LV (2) U111<br>s(0.05%)p 5.88(0.31%)d99.99(87.80%)f99.99(11.84%)g 0.16(0.01%)	8.51
(0.95250) BD (1) O 5- U111<br>(94.61%) 0.9727* O 5 s(99.95%)p 0.00(0.02%)d 0.00(0.02%)	(0.10854) LV (1) U 1<br>s(2.02%)p 0.46(0.92%)d44.28(89.65%)f 3.64(7.38%)g 0.01(0.02%)	39.60

(5.39%) 0.2321* U111 s(7.99%)p 0.04(0.33%)d 9.50(75.86%)f 1.98(15.80%)g 0.00(0.03%)		
(0.92377) BD (2) O 5- U111<br>(92.53%) 0.9619* O 5 s(0.02%)p99.99(99.98%)d 0.00(0.00%)<br>(7.47%) 0.2734* U111 s(0.01%)p 1.00(0.38%)d99.99(62.33%)f99.21(37.26%)g 0.06(0.02%)	(0.10606) LV (2) U 1<br>s(0.04%)p 2.39(0.10%)d99.99(93.78%)f99.99(6.07%)g 0.03(0.00%)	13.78

DFT computed NBO second order perturbation analysis for UOU core in **$[^{Ar}L_3UOU^{Ar}L_3]^{1-}$**

Donor NBO	Acceptor NBO	E(2) kcal/mol
(0.98841) BD (1) U 1- O 5<br>(10.68%) 0.3269* U 1 s(0.18%)p 4.11(0.76%)d99.99(46.70%)f99.99(52.25%)g 0.60(0.11%)<br>(89.32%) 0.9451* O 5 s(46.82%)p 1.13(53.14%)d 0.00(0.04%)	(0.20431) LV (1) U111<br>s(7.00%)p 0.09(0.66%)d 9.23(64.58%)f 3.96(27.72%)g 0.01(0.04%)	5.30
(0.94954) BD (2) U 1- O 5<br>(10.69%) 0.3269* U 1 s(0.07%)p 4.71(0.34%)d99.99(54.23%)f99.99(45.27%)g 1.18(0.09%)<br>(89.31%) 0.9451* O 5 s(0.03%)p99.99(99.96%)d 0.62(0.02%)	(0.08544) LV (4) U111<br>s(0.05%)p 4.62(0.23%)d99.99(66.53%)f99.99(33.18%)g 0.25(0.01%)	3.62
(0.94697) BD (3) U 1- O 5<br>(10.47%) 0.3236* U 1 s(0.01%)p 1.00(0.32%)d99.99(55.25%)f99.99(44.34%)g 0.27(0.08%)<br>(89.53%) 0.9462* O 5 s(0.00%)p 1.00(99.98%)d 0.00(0.01%)	(0.08566) LV (3) U111<br>s(0.02%)p 9.89(0.21%)d99.99(69.07%)f99.99(30.68%)g 0.54(0.01%)	3.30
(0.97879) BD (1) O 5- U111<br>(82.72%) 0.9095* O 5 s(53.11%)p 0.88(46.89%)d 0.00(0.01%)<br>(17.28%) 0.4156* U111 s(0.15%)p 0.60(0.09%)d99.99(21.93%)f99.99(77.81%)g 0.14(0.02%)	(0.20431) LV (1) U111<br>s(7.00%)p 0.09(0.66%)d 9.23(64.58%)f 3.96(27.72%)g 0.01(0.04%)	10.56

DFT computed NBO second order perturbation analysis for UOU core in **$[^{Ar}L_3UOU^{Ar}L_3]$**

Donor NBO	Acceptor NBO	E(2) kcal/mol
(0.85318) LP (1) O 5<br>s(0.00%)p 1.00(100.00%)d 0.00(0.00%)	(0.06331) LV (2) U 1<br>s(0.15%)p 1.25(0.19%)d96.63(14.30%)f99.99(85.28%)g 0.62(0.09%)	4.48
(0.85318) LP (1) O 5<br>s(0.00%)p 1.00(100.00%)d 0.00(0.00%)	(0.05793) LV (3) U 1<br>s(0.80%)p 0.40(0.32%)d29.20(23.36%)f94.27(75.40%)g 0.15(0.12%)	7.50
(0.85318) LP (1) O 5<br>s(0.00%)p 1.00(100.00%)d 0.00(0.00%)	(0.06173) LV (2) U111<br>s(0.02%)p 6.54(0.13%)d99.99(8.84%)f99.99(90.92%)g 4.37(0.09%)	4.17
(0.85318) LP (1) O 5<br>s(0.00%)p 1.00(100.00%)d 0.00(0.00%)	(0.05715) LV (3) U111<br>s(0.06%)p 7.67(0.48%)d99.99(26.30%)f99.99(73.04%)g 1.85(0.12%)	8.50
(0.85217) LP (2) O 5<br>s(0.00%)p 1.00(100.00%)d 0.00(0.00%)	(0.13481) LV (1) U111<br>s(0.00%)p 0.00(0.01%)d 1.00(95.46%)f 0.05(4.52%)g 0.00(0.00%)	10.21

DFT computed NBO second order perturbation analysis for UNU core in **[$^{Ar}L_3UNU^{Ar}L_3$]$^{3-}$**

Donor NBO	Acceptor NBO	E(2) kcal/mol
(0.97205) BD (1) U 1- N 5<br>(13.36%) 0.3654* U 1 s(7.60%)p 0.18(1.36%)d 9.66(73.36%)f 2.32(17.62%)g 0.01(0.06%)<br>(86.64%) 0.9308* N 5 s(49.92%)p 1.00(50.07%)d 0.00(0.01%)	(0.01052) LV (8) U111<br>s(11.31%)p 0.84(9.55%)d 2.11(23.90%)f 4.88(55.20%)g 0.00(0.05%)	4.34
(0.96806) BD (1) N 5- U111<br>(86.46%) 0.9298* N 5 s(47.99%)p 1.08(52.00%)d 0.00(0.02%)<br>(13.54%) 0.3679* U111 s(8.16%)p 0.21(1.75%)d 9.72(79.25%)f 1.32(10.78%)g 0.01(0.06%)	(0.01712) LV (8) U 1<br>s(7.51%)p 0.18(1.35%)d 3.41(25.64%)f 8.70(65.37%)g 0.02(0.12%)	4.55
(0.86833) BD (2) N 5- U111<br>(86.19%) 0.9284* N 5 s(0.27%)p99.99(99.73%)d 0.00(0.00%)<br>(13.81%) 0.3716* U111 s(0.06%)p 4.62(0.29%)d99.99(65.15%)f99.99(34.49%)g 0.24(0.01%)	(0.14159) LV (1) U 1<br>s(0.04%)p 3.54(0.16%)d99.99(79.99%)f99.99(19.80%)g 0.11(0.00%)	23.24

DFT computed NBO second order perturbation analysis for UNU core in **[$^{Ar}L_3UNU^{Ar}L_3$]$^{2-}$**

Donor NBO	Acceptor NBO	E(2) kcal/mol
(0.91792) BD (2) U 1- N 5<br>(19.93%) 0.4465* U 1 s(0.01%)p 1.00(0.11%)d99.99(54.40%)f99.99(45.45%)g 0.23(0.03%)<br>(80.07%) 0.8948* N 5 s(0.12%)p99.99(99.86%)d 0.14(0.02%)	(0.09999) LV (2) U111<br>s(0.03%)p 5.77(0.15%)d99.99(89.70%)f99.99(10.13%)g 0.15(0.00%)	7.25
(0.91604) BD (3) U 1- N 5<br>(19.61%) 0.4429* U 1 s(0.00%)p 1.00(0.14%)d99.99(51.82%)f99.99(48.01%)g 0.17(0.02%)<br>(80.39%) 0.8966* N 5 s(0.04%)p99.99(99.95%)d 0.44(0.02%)	(0.10994) LV (1) U111<br>s(0.01%)p 1.00(0.06%)d99.99(93.98%)f92.41(5.95%)g 0.01(0.00%)	8.98
(0.96509) BD (1) N 5- U111<br>(88.66%) 0.9416* N 5 s(57.48%)p 0.74(42.52%)d 0.00(0.00%)<br>(11.34%) 0.3368* U111 s(11.11%)p 0.09(0.97%)d 7.09(78.77%)f 0.82(9.10%)g 0.00(0.05%)	(0.10833) LV (2) U 1<br>s(2.01%)p 0.16(0.32%)d33.58(67.45%)f15.04(30.22%)g 0.00(0.01%)	5.67
(0.96509) BD (1) N 5- U111<br>(88.66%) 0.9416* N 5 s(57.48%)p 0.74(42.52%)d 0.00(0.00%)<br>(11.34%) 0.3368* U111 s(11.11%)p 0.09(0.97%)d 7.09(78.77%)f 0.82(9.10%)g 0.00(0.05%)	(0.04880) LV (6) U 1<br>s(77.49%)p 0.02(1.78%)d 0.06(4.57%)f 0.21(16.15%)g 0.00(0.01%)	3.15

DFT computed NBO second order perturbation analysis for UNU core in **[$^{Ar}L_3UNU^{Ar}L_3$]$^{1-}$**

Donor NBO	Acceptor NBO	E(2) kcal/mol
(0.73688) LP (1) N 5<br>s(0.14%)p99.99(99.86%)d 0.00(0.00%)	(0.05823) LV (3) U 1<br>s(52.46%)p 0.01(0.54%)d 0.39(20.56%)f 0.50(26.27%)g 0.00(0.17%)	3.52
(0.73688) LP (1) N 5<br>s(0.14%)p99.99(99.86%)d 0.00(0.00%)	(0.09225) LV (2) U111<br>s(1.15%)p 0.72(0.82%)d27.56(31.58%)f57.90(66.35%)g 0.09(0.10%)	23.52
(0.73171) LP (2) N 5<br>s(0.00%)p 1.00(100.00%)d 0.00(0.00%)	(0.13811) LV (1) U 1	23.26

	s(0.01%)p 1.00(0.09%)d99.99(85.90%)f99.99(14.00%)g 0.08(0.01%)	
(0.73171) LP (2) N 5<br>s(0.00%)p 1.00(100.00%)d 0.00(0.00%)	(0.13569) LV (1) U111<br>s(0.00%)p 1.00(0.08%)d99.99(86.86%)f99.99(13.05%)g 0.09(0.01%)	22.59
(0.73171) LP (2) N 5<br>s(0.00%)p 1.00(100.00%)d 0.00(0.00%)	(0.06531) LV (3) U111<br>s(0.02%)p 7.44(0.12%)d99.99(5.88%)f99.99(93.90%)g 5.37(0.08%)	4.13
(0.96964) BD (1) U 1- N 5<br>(14.08%) 0.3752* U 1 s(5.93%)p 0.16(0.94%)d10.71(63.57%) f 4.97(29.49%)g 0.01(0.07%)<br>(85.92%) 0.9270* N 5 s(54.15%)p 0.85(45.84%)d 0.00(0.01%)	(0.03896) LV (4) U111<br>s(88.00%)p 0.01(0.69%)d 0.08(6.77%)f 0.05(4.53%)g 0.00(0.01%)	4.91

DFT computed NBO second order perturbation analysis for USU core in **[$^{Ar}L_3USU^{Ar}L_3$]$^{2-}$**

Donor NBO	Acceptor NBO	E(2) kcal/mol
(0.84555) LP (1) S 5<br>s(0.05%)p99.99(99.95%)d 0.01(0.00%)	(0.11911) LV (1) U 1<br>s(0.07%)p 0.25(0.02%)d99.99(95.75%)f56.13(4.15%)g 0.01(0.00%)	13.14
(0.84555) LP (1) S 5<br>s(0.05%)p99.99(99.95%)d 0.01(0.00%)	(0.12209) LV (1) U111<br>s(0.00%)p 1.00(0.03%)d99.99(95.54%)f99.99(4.43%)g 0.04(0.00%)	14.39

DFT computed NBO second order perturbation analysis for USU core in **[$^{Ar}L_3USU^{Ar}L_3$]$^{1-}$**

Donor NBO	Acceptor NBO	E(2) kcal/mol
(0.94026) BD (2) U 1- S 5<br>(14.49%) 0.3807* U 1 s(0.03%)p 9.37(0.26%)d99.99(59.00%)f99.99(40.71%)g 0.26(0.01%)<br>(85.51%) 0.9247* S 5 s(0.02%)p99.99(99.97%)d 0.61(0.01%)	(0.09313) LV (3) U111<br>s(0.00%)p 1.00(0.45%)d99.99(93.58%)f13.19(5.95%)g 0.04(0.02%)	4.32
(0.93534) BD (3) U 1- S 5<br>(13.13%) 0.3623* U 1 s(0.07%)p 2.18(0.15%)d99.99(44.38%)f99.99(55.38%)g 0.20(0.01%)<br>(86.87%) 0.9321* S 5 s(0.22%)p99.99(99.78%)d 0.03(0.01%)	(0.11402) LV (1) U111<br>s(0.03%)p 0.21(0.01%)d99.99(97.93%)f80.09(2.03%)g 0.11(0.00%)	7.48
(0.97899) BD (1) S 5- U111<br>(89.82%) 0.9477* S 5 s(53.93%)p 0.85(46.06%)d 0.00(0.01%)<br>(10.18%) 0.3191* U111 s(12.01%)p 0.03(0.42%)d 6.75(81.04%)f 0.54(6.51%)g 0.00(0.01%)	(0.04398) LV (4) U 1<br>s(87.20%)p 0.00(0.09%)d 0.09(7.94%)f 0.05(4.71%)g 0.00(0.06%)	4.83

DFT computed NBO second order perturbation analysis for USU core in **[$^{Ar}L_3USU^{Ar}L_3$]**

Donor NBO	Acceptor NBO	E(2) kcal/mol
(0.97062) BD (1) U 1- S 5<br>(13.41%) 0.3662* U 1 s(20.48%)p 0.02(0.50%)d 3.07(62.96%) f 0.78(16.01%)g 0.00(0.05%)<br>(86.59%) 0.9305* S 5 s(49.97%)p 1.00(50.01%)d 0.00(0.03%)	(0.04787) LV (2) U111<br>s(67.45%)p 0.00(0.12%)d 0.37(24.81%)f 0.11(7.53%)g 0.00(0.10%)	4.62
(0.97062) BD (1) U 1- S 5<br>(13.41%) 0.3662* U 1 s(20.48%)p 0.02(0.50%)d 3.07(62.96%) f 0.78(16.01%)g 0.00(0.05%)	(0.04101) BD*(1) S 5- U111<br>(13.53%) 0.3678* S 5 s(49.65%)p 1.01(50.32%)d 0.00(0.02%)	2.51

(86.59%) 0.9305* S 5 s(49.97%)p 1.00(50.01%)d 0.00(0.03%)	(86.47%) -0.9299* U111 s(11.51%)p 0.03(0.40%)d 6.56(75.54%)f 1.09(12.52%)g 0.00(0.03%)	
(0.97243) BD (1) S 5- U111<br>(86.47%) 0.9299* S 5 s(49.65%)p 1.01(50.32%)d 0.00(0.02%)<br>(13.53%) 0.3678* U111 s(11.51%)p 0.03(0.40%)d 6.56(75.54%) f 1.09(12.52%)g 0.00(0.03%)	(0.03386) BD*(1) U 1- S 5<br>(86.59%) 0.9305* U 1 s(20.48%)p 0.02(0.50%)d 3.07(62.96%)f 0.78(16.01%)g 0.00(0.05%)	2.67

DFT computed MOs (Alpha spin orbitals) for ground spin state

	**$[^{Ar}L_3UOU^{Ar}L_3]^{2-}$**<br>S=3	**$[^{Ar}L_3UOU^{Ar}L_3]^{1-}$**<br>S=5/2	**$[^{Ar}L_3UOU^{Ar}L_3]$**<br>S=2
HOMO-5	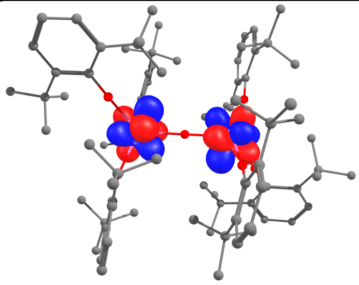		
HOMO-4	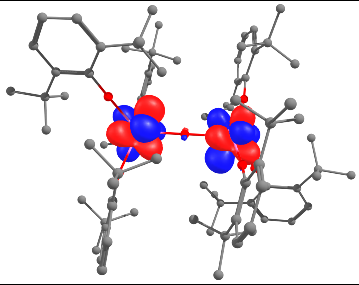	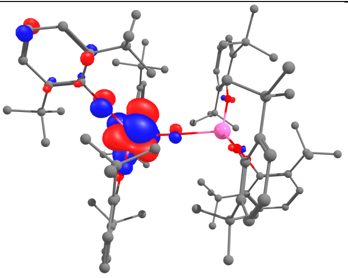	
HOMO-3	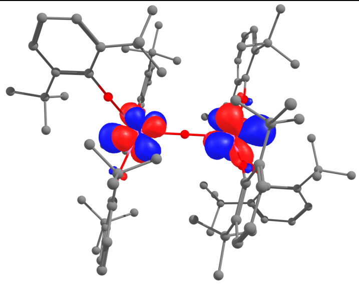	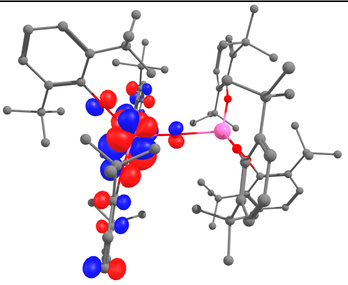	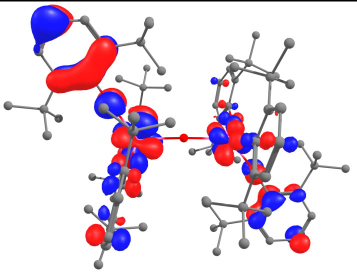
HOMO-2	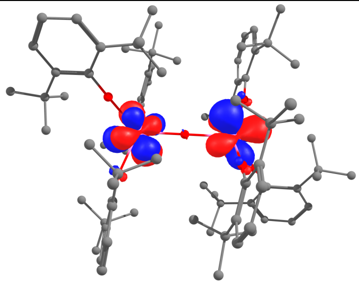	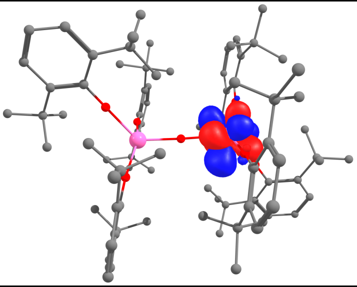	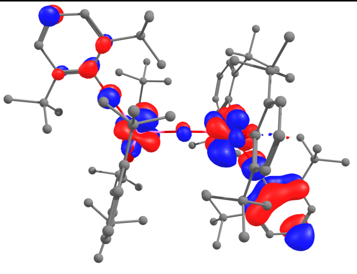
HOMO-1	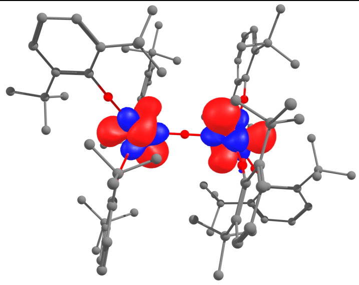	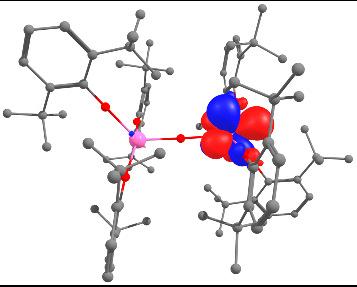	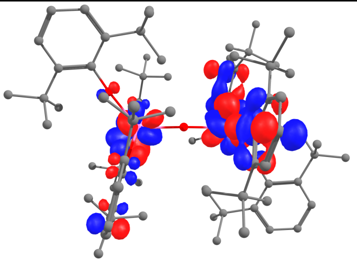

HOMO	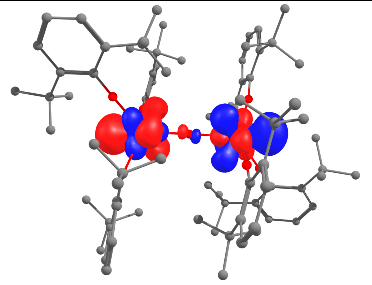	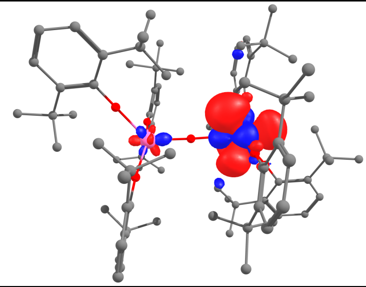	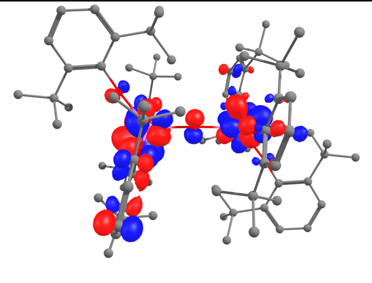
LUMO	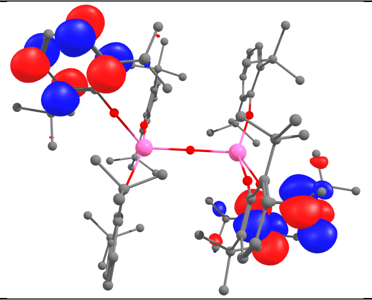	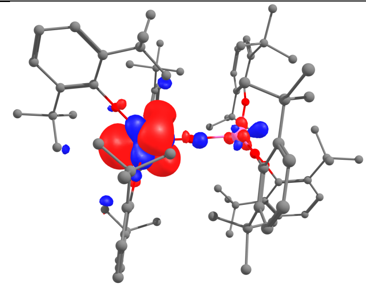	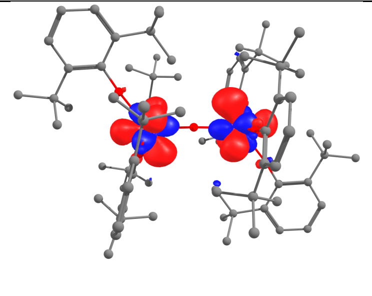

	**[$^{Ar}L_3UNU^{Ar}L_3$]$^{3-}$** S=3	**[$^{Ar}L_3UNU^{Ar}L_3$]$^{2-}$** S=5/2	**[$^{Ar}L_3UNU^{Ar}L_3$]$^{-}$** S=2
HOMO-5	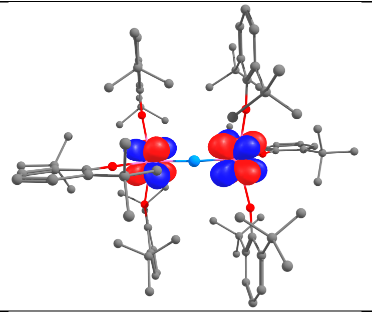		
HOMO-4	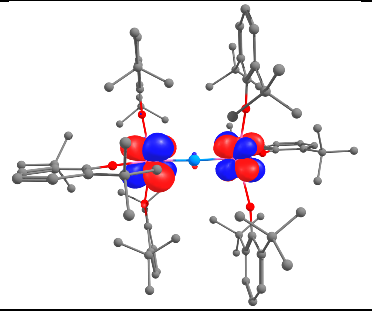	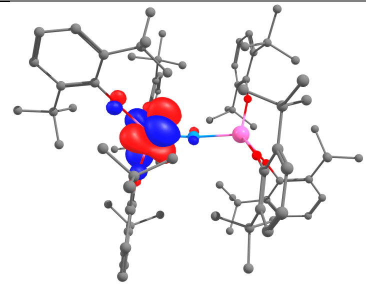	
HOMO-3	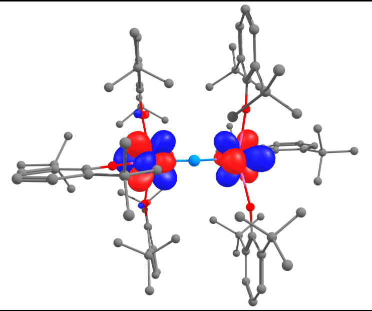	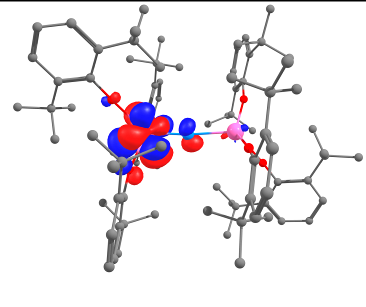	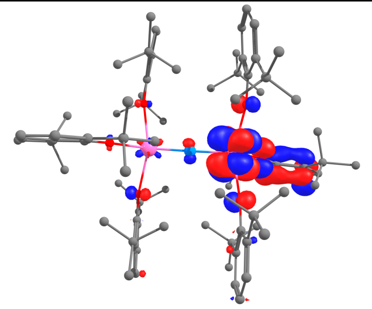
HOMO-2	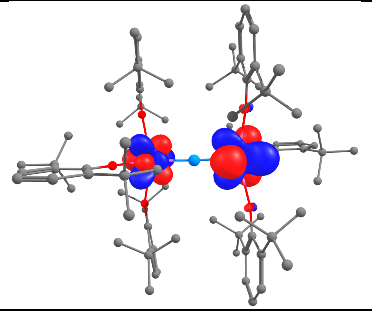	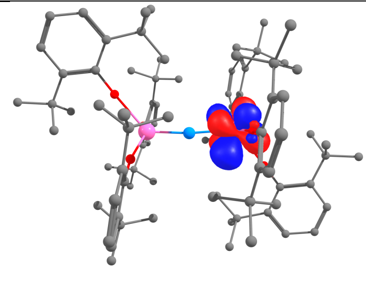	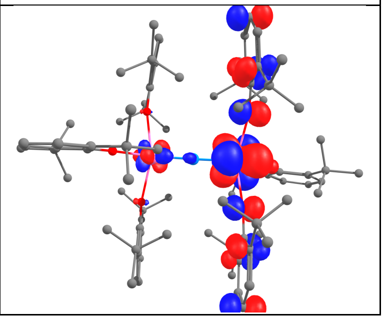

HOMO-1	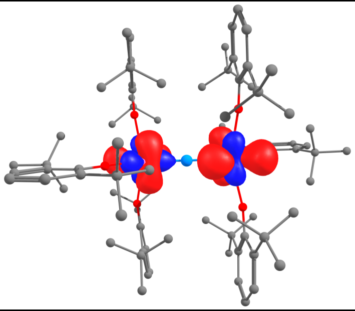	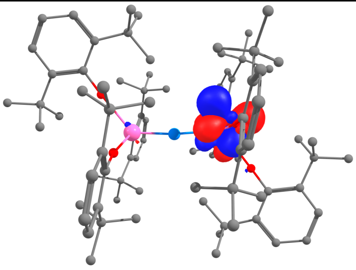	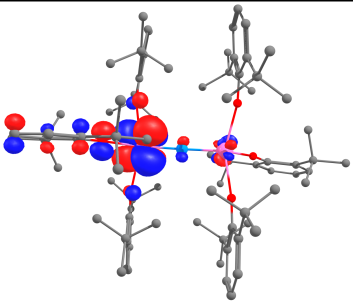
HOMO	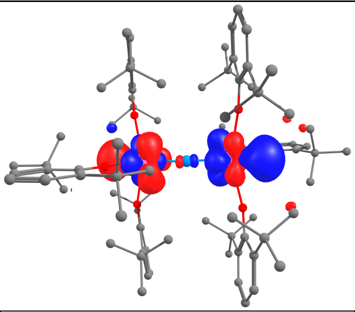	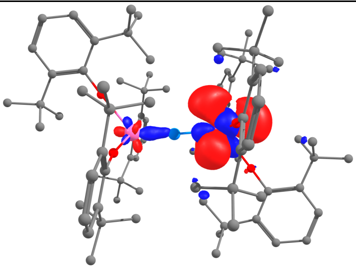	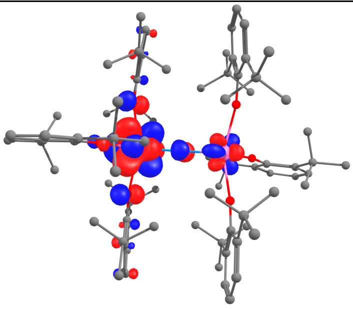
LUMO	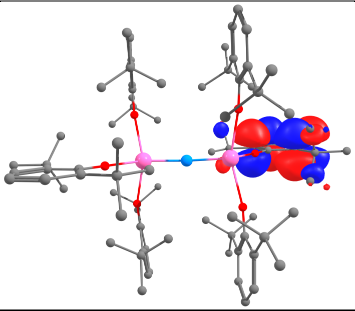	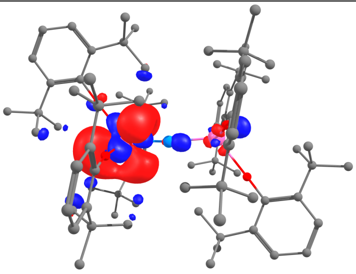	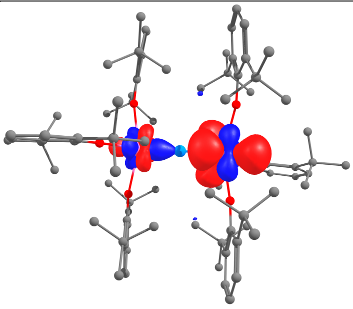
	**$[^{Ar}L_3USU^{Ar}L_3]^{2-}$**<br>S=3	**$[^{Ar}L_3USU^{Ar}L_3]^{1-}$**<br>S=5/2	**$[^{Ar}L_3USU^{Ar}L_3]$**<br>S=2
HOMO-5	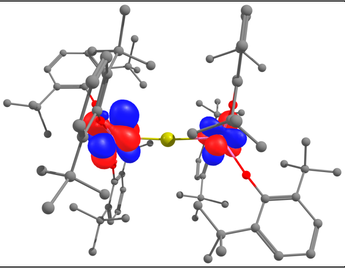		
HOMO-4	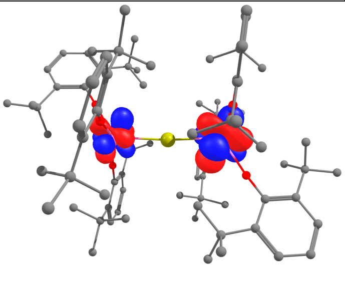	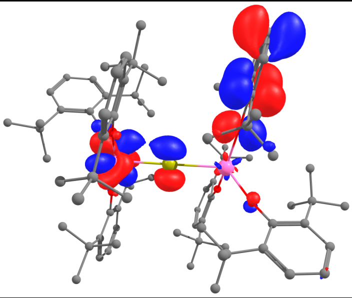	
HOMO-3	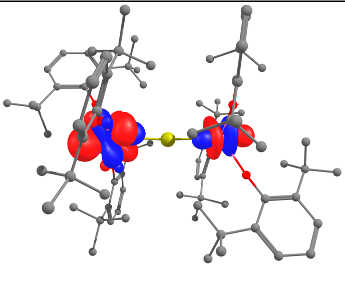	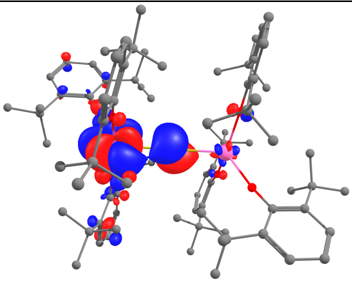	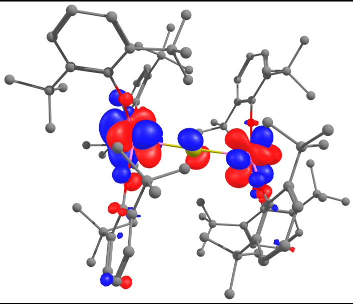

HOMO-2	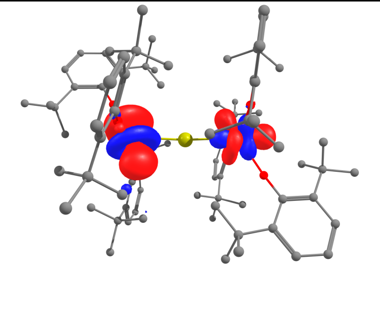	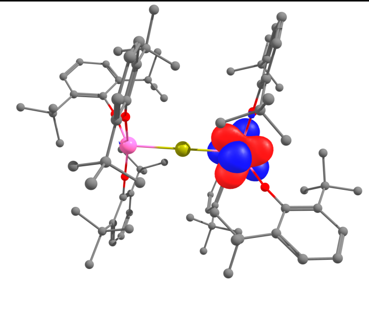	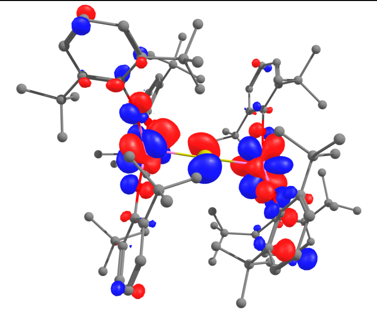
HOMO-1	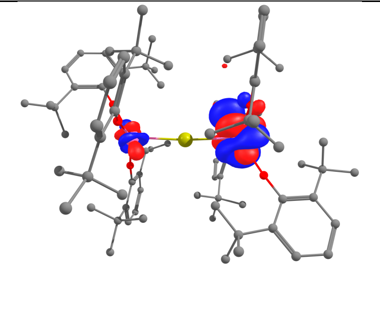	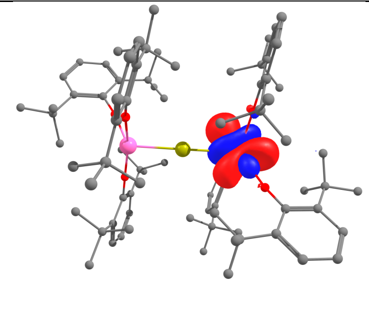	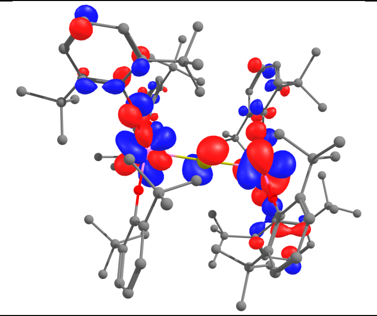
HOMO	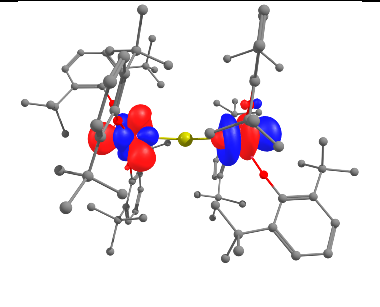	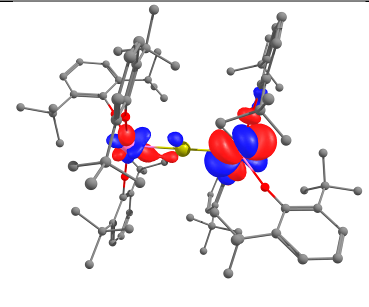	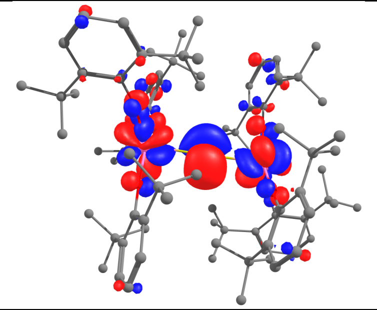
LUMO	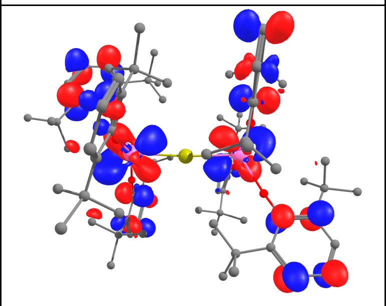	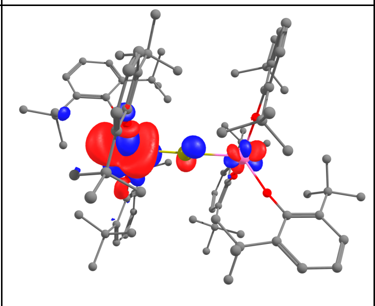	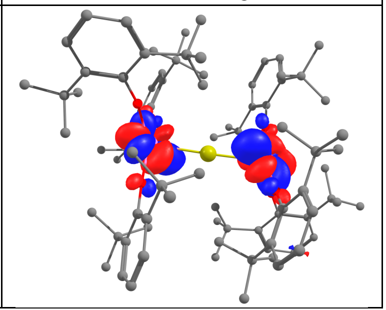

<u>Optimized geometries</u>

$[^{Si}L_3UOU^{Si}L_3]^{2-}$ E = -5700.29774208

U	11.841498000	17.740456000	7.846554000
Si	9.479854000	19.233628000	10.436058000
Si	12.663006000	14.202536000	8.998977000
Si	10.865591000	17.782077000	4.208511000
O	13.588270000	18.808035000	8.358215000
O	10.299081000	18.602958000	9.194932000
O	9.655857000	20.895431000	10.596594000
O	9.858162000	18.635365000	11.951999000
O	7.869141000	18.830458000	10.199608000
O	12.314749000	15.608587000	8.293768000
O	14.088179000	14.138246000	9.879270000
O	11.485526000	13.710538000	10.094956000
O	12.847942000	13.060851000	7.789322000
O	11.411533000	18.117129000	5.686330000
O	11.754091000	16.656931000	3.337642000
O	10.931891000	19.185953000	3.297874000
O	9.299567000	17.163300000	4.205616000

C	9.860874000	21.838528000	9.535838000
C	8.999975000	21.479760000	8.326802000
H	9.315008000	20.518531000	7.913563000
H	9.089578000	22.240224000	7.545376000
H	7.947120000	21.404036000	8.616632000
C	11.341284000	21.859412000	9.172734000
H	11.555998000	22.567934000	8.370378000
H	11.675710000	20.870641000	8.846281000
H	11.938117000	22.143018000	10.043698000
C	9.435758000	23.192525000	10.099508000
H	8.377665000	23.177572000	10.382997000
H	9.584855000	23.986116000	9.360114000
H	10.024407000	23.436890000	10.989793000
C	11.020679000	18.944791000	12.736309000
C	12.233234000	19.238714000	11.858667000
H	12.419611000	18.426567000	11.155248000
H	13.130239000	19.370223000	12.469497000
H	12.079969000	20.150295000	11.278179000
C	10.703772000	20.156648000	13.610383000
H	10.468053000	21.014007000	12.974685000
H	11.558730000	20.411251000	14.246825000
H	9.842861000	19.950900000	14.256040000
C	11.265077000	17.711395000	13.601056000
H	10.385937000	17.500364000	14.219470000
H	12.124321000	17.860329000	14.262220000
H	11.462298000	16.838454000	12.973970000
C	6.795622000	18.902701000	11.138375000
C	5.521312000	18.988312000	10.300700000
H	5.523595000	19.898803000	9.692506000
H	4.628682000	18.998495000	10.935431000
H	5.455557000	18.129215000	9.625062000
C	6.913877000	20.129902000	12.041875000
H	7.822013000	20.072351000	12.646633000
H	6.051026000	20.191503000	12.714290000
H	6.958724000	21.046160000	11.447179000
C	6.796584000	17.625138000	11.978183000
H	6.680309000	16.748636000	11.332676000
H	5.975987000	17.629170000	12.704766000
H	7.745687000	17.538307000	12.512892000
C	14.348686000	14.808053000	11.123755000
C	15.857656000	15.014290000	11.193294000
H	16.386683000	14.065203000	11.055780000
H	16.144557000	15.436933000	12.160863000
H	16.173899000	15.711835000	10.414937000
C	13.654973000	16.163056000	11.179078000
H	13.955007000	16.788534000	10.333927000
H	13.924883000	16.688275000	12.098680000
H	12.568073000	16.047218000	11.151207000
C	13.871453000	13.909142000	12.262351000
H	12.799603000	13.722237000	12.161261000
H	14.062181000	14.376932000	13.234432000
H	14.396196000	12.947758000	12.235536000
C	10.071800000	13.907420000	9.977452000
C	9.615675000	13.829864000	8.520588000
H	9.885543000	12.864002000	8.082663000
H	10.091295000	14.618125000	7.931668000
H	8.529492000	13.951467000	8.452900000
C	9.430847000	12.782874000	10.788204000
H	9.787499000	12.809837000	11.823102000
H	9.689412000	11.807279000	10.363529000
H	8.339670000	12.877983000	10.797367000
C	9.711353000	15.261821000	10.580317000
H	10.182808000	16.078865000	10.029617000

H	10.042639000	15.317234000	11.621451000
H	8.629382000	15.425096000	10.558721000
C	13.418687000	11.755194000	7.875677000
C	14.939825000	11.876854000	7.787256000
H	15.416849000	10.890140000	7.795087000
H	15.311152000	12.459035000	8.633614000
H	15.221708000	12.389559000	6.863049000
C	12.877360000	10.990260000	6.669512000
H	13.158559000	11.498764000	5.741798000
H	11.784672000	10.935631000	6.711475000
H	13.274444000	9.969883000	6.637660000
C	13.014714000	11.049884000	9.169793000
H	13.431931000	10.037256000	9.198455000
H	11.926575000	10.979107000	9.248534000
H	13.383227000	11.601132000	10.038605000
C	11.819398000	15.242154000	3.577984000
C	11.733382000	14.928824000	5.066701000
H	10.773142000	15.257259000	5.474099000
H	12.528980000	15.427476000	5.623344000
H	11.825922000	13.855313000	5.251330000
C	10.674280000	14.572436000	2.820385000
H	9.717900000	14.956665000	3.183907000
H	10.696963000	13.485641000	2.958618000
H	10.749009000	14.783590000	1.748024000
C	13.167020000	14.785964000	3.026861000
H	13.983329000	15.249508000	3.586511000
H	13.264839000	15.067213000	1.972998000
H	13.271086000	13.698466000	3.105216000
C	10.835490000	19.330047000	1.879728000
C	12.226244000	19.122683000	1.283352000
H	12.218058000	19.260490000	0.196227000
H	12.577108000	18.113545000	1.512940000
H	12.926302000	19.841554000	1.718570000
C	9.839563000	18.341646000	1.274501000
H	9.752442000	18.504812000	0.194724000
H	8.851545000	18.463906000	1.726028000
H	10.167979000	17.313587000	1.444744000
C	10.361011000	20.761225000	1.638558000
H	9.377235000	20.918868000	2.092474000
H	10.286894000	20.976753000	0.567235000
H	11.060815000	21.474270000	2.084845000
C	8.208142000	17.574797000	5.036282000
C	6.942873000	17.264677000	4.238950000
H	6.928938000	17.839962000	3.307494000
H	6.044890000	17.514763000	4.813809000
H	6.905007000	16.200475000	3.983893000
C	8.276784000	19.067279000	5.356222000
H	9.208479000	19.304231000	5.875715000
H	7.441041000	19.360315000	5.999770000
H	8.235107000	19.661687000	4.438592000
C	8.232218000	16.750136000	6.321707000
H	8.236101000	15.681389000	6.086478000
H	7.361901000	16.967647000	6.949645000
H	9.127326000	16.980560000	6.907357000
U	15.420560000	19.792194000	8.612623000
Si	17.701214000	18.223446000	5.963613000
Si	14.578393000	23.245167000	7.295720000
Si	16.477592000	19.910304000	12.237619000
O	16.853854000	18.914138000	7.150182000
O	17.648455000	16.542939000	5.969322000
O	17.254690000	18.686261000	4.421963000
O	19.284034000	18.757230000	6.109523000
O	15.028293000	21.896978000	8.056867000

O	13.093852000	23.202464000	6.523587000
O	15.660357000	23.714506000	6.098144000
O	14.432781000	24.451728000	8.449320000
O	15.877280000	19.526393000	10.793259000
O	15.950776000	21.342736000	12.929502000
O	16.022007000	18.713634000	13.320417000
O	18.150020000	20.089699000	12.232835000
C	17.358274000	15.695642000	7.092432000
C	18.118753000	16.160309000	8.332232000
H	17.773295000	17.150208000	8.639491000
H	17.960224000	15.464193000	9.160104000
H	19.192113000	16.214562000	8.131951000
C	15.855565000	15.692480000	7.356469000
H	15.595342000	15.048451000	8.199237000
H	15.503305000	16.702613000	7.582392000
H	15.312968000	15.329843000	6.478227000
C	17.829225000	14.303980000	6.679517000
H	18.907620000	14.304798000	6.486879000
H	17.617605000	13.572427000	7.465678000
H	17.318700000	13.986550000	5.764567000
C	16.147172000	18.214998000	3.645333000
C	14.913030000	17.976988000	4.510635000
H	14.670533000	18.870064000	5.086322000
H	14.043891000	17.718549000	3.900046000
H	15.084788000	17.161393000	5.216645000
C	16.560484000	16.925230000	2.937084000
H	16.805111000	16.160168000	3.678079000
H	15.751648000	16.555846000	2.297793000
H	17.442568000	17.097531000	2.311029000
C	15.874674000	19.314724000	2.623385000
H	16.782726000	19.532459000	2.050721000
H	15.088628000	19.011049000	1.926363000
H	15.557006000	20.232580000	3.126044000
C	20.451248000	18.307439000	5.421919000
C	21.102720000	17.197861000	6.246729000
H	20.437425000	16.332061000	6.296967000
H	22.052523000	16.879942000	5.802122000
H	21.298081000	17.545190000	7.266257000
C	20.137494000	17.790600000	4.018059000
H	19.628300000	18.555890000	3.428563000
H	21.063694000	17.503129000	3.507882000
H	19.486357000	16.913763000	4.069181000
C	21.375834000	19.521192000	5.340176000
H	21.570600000	19.915679000	6.342695000
H	22.333941000	19.260286000	4.877754000
H	20.910017000	20.314408000	4.746812000
C	12.762136000	22.450499000	5.344384000
C	11.266802000	22.169428000	5.425705000
H	10.701309000	23.103443000	5.513922000
H	10.927699000	21.626184000	4.539288000
H	11.053714000	21.547045000	6.297556000
C	13.517027000	21.128641000	5.288108000
H	13.328040000	20.541443000	6.190128000
H	13.170486000	20.542013000	4.433528000
H	14.594723000	21.289985000	5.196059000
C	13.090791000	23.308044000	4.124320000
H	14.157177000	23.547088000	4.118751000
H	12.840243000	22.779672000	3.197839000
H	12.523655000	24.245210000	4.149812000
C	17.075815000	23.496796000	6.068139000
C	17.693850000	23.616537000	7.460877000
H	17.466380000	24.591840000	7.902646000
H	17.304092000	22.836302000	8.119569000

H	18.782434000	23.509196000	7.404590000
C	17.636351000	24.584128000	5.153538000
H	17.171757000	24.526478000	4.163700000
H	17.432885000	25.576933000	5.568572000
H	18.719517000	24.475537000	5.032820000
C	17.348053000	22.115301000	5.480243000
H	16.932830000	21.329091000	6.114184000
H	16.898569000	22.028920000	4.486354000
H	18.423424000	21.934215000	5.383823000
C	13.803346000	25.727060000	8.321559000
C	12.298870000	25.557643000	8.532690000
H	11.785321000	26.525319000	8.498553000
H	11.890032000	24.910493000	7.753523000
H	12.102811000	25.096383000	9.505242000
C	14.402111000	26.593417000	9.427468000
H	14.215709000	26.141662000	10.406929000
H	15.484922000	26.686104000	9.294699000
H	13.965496000	27.597967000	9.423022000
C	14.077875000	26.353727000	6.955050000
H	13.614005000	27.344045000	6.889576000
H	15.152570000	26.461027000	6.785524000
H	13.668724000	25.726068000	6.159223000
C	15.611486000	22.559150000	12.251932000
C	14.219241000	22.409297000	11.643869000
H	14.210462000	21.591689000	10.919670000
H	13.485088000	22.184212000	12.424009000
H	13.919997000	23.321477000	11.120331000
C	16.640562000	22.897968000	11.175441000
H	16.678192000	22.111701000	10.416415000
H	16.367161000	23.826466000	10.666289000
H	17.636922000	23.006543000	11.613954000
C	15.608206000	23.640859000	13.329167000
H	14.890748000	23.392548000	14.118389000
H	16.600324000	23.731743000	13.783993000
H	15.331762000	24.611997000	12.905471000
C	16.029458000	18.757316000	14.747229000
C	14.779560000	19.495201000	15.227900000
H	14.723226000	19.507372000	16.322160000
H	14.791912000	20.522692000	14.857511000
H	13.883570000	19.000162000	14.842399000
C	17.288466000	19.442767000	15.278039000
H	17.297275000	19.431296000	16.373568000
H	18.186449000	18.932517000	14.918638000
H	17.328657000	20.482736000	14.942841000
C	15.990751000	17.300195000	15.203687000
H	16.875364000	16.763988000	14.845206000
H	15.962365000	17.228358000	16.296337000
H	15.102869000	16.801527000	14.802438000
C	19.084553000	19.272393000	11.522981000
C	20.413023000	19.426469000	12.261042000
H	20.320236000	19.071548000	13.292731000
H	21.205663000	18.853498000	11.767966000
H	20.713381000	20.479114000	12.287560000
C	18.650716000	17.807520000	11.524209000
H	17.680834000	17.697372000	11.033300000
H	19.380210000	17.189098000	10.991445000
H	18.561767000	17.436016000	12.549114000
C	19.217244000	19.792554000	10.092388000
H	19.482555000	20.854659000	10.097392000
H	19.989411000	19.245094000	9.541920000
H	18.276121000	19.679002000	9.547422000

[$^{Si}L_3UOU^{Si}L_3$]$^{1-}$ E = -5700.25035695

U	11.807121000	17.460628000	7.775487000
Si	9.507686000	19.183216000	10.210655000
Si	12.724480000	14.154576000	9.375691000
Si	10.814701000	17.727481000	4.326302000
O	13.444722000	18.520900000	8.157611000
O	10.422875000	18.398391000	9.103726000
O	9.708358000	20.835483000	10.151030000
O	9.818608000	18.741890000	11.783460000
O	7.942965000	18.717004000	9.879963000
O	12.354759000	15.604921000	8.727909000
O	14.225960000	14.072964000	10.085827000
O	11.628947000	13.715172000	10.558628000
O	12.732746000	13.073706000	8.108073000
O	11.309266000	18.228735000	5.797423000
O	11.849351000	16.645042000	3.583544000
O	10.754023000	19.072419000	3.352654000
O	9.354466000	16.913207000	4.418839000
C	9.990078000	21.656932000	9.004688000
C	9.268811000	21.127585000	7.768865000
H	9.653702000	20.145132000	7.488111000
H	9.414966000	21.801010000	6.919435000
H	8.195135000	21.037716000	7.958755000
C	11.498523000	21.685578000	8.793237000
H	11.779051000	22.333516000	7.959966000
H	11.877221000	20.680829000	8.589079000
H	11.996998000	22.056499000	9.692591000
C	9.473286000	23.046906000	9.361911000
H	8.390220000	23.025332000	9.521797000
H	9.692783000	23.759831000	8.561239000
H	9.948827000	23.402898000	10.281008000
C	10.955356000	19.132583000	12.579218000
C	12.223426000	19.197427000	11.733830000
H	12.385431000	18.253043000	11.212560000
H	13.097699000	19.389271000	12.360791000
H	12.162849000	19.992446000	10.987248000
C	10.658499000	20.487299000	13.216865000
H	10.495043000	21.237738000	12.440318000
H	11.493289000	20.804930000	13.850502000
H	9.759431000	20.427659000	13.839754000
C	11.089826000	18.059029000	13.653375000
H	10.138086000	17.923580000	14.177129000
H	11.849030000	18.344143000	14.387038000
H	11.382702000	17.103491000	13.210183000
C	6.778570000	18.921981000	10.691698000
C	5.594641000	18.884134000	9.729648000
H	5.670788000	19.691849000	8.994685000
H	4.648332000	18.995764000	10.269033000
H	5.572846000	17.931705000	9.190499000
C	6.829276000	20.267351000	11.413056000
H	7.669439000	20.301786000	12.111887000
H	5.906155000	20.427154000	11.980049000
H	6.946022000	21.086207000	10.698372000
C	6.689434000	17.777295000	11.699089000
H	6.627873000	16.817652000	11.175897000
H	5.801912000	17.879750000	12.333122000
H	7.579402000	17.772117000	12.333233000
C	14.660020000	14.779784000	11.265953000
C	16.157359000	15.004371000	11.094752000
H	16.667781000	14.057119000	10.892994000
H	16.584233000	15.443539000	12.000907000
H	16.345540000	15.689552000	10.264813000

C	13.955312000	16.124203000	11.399522000
H	14.097295000	16.719519000	10.494239000
H	14.369647000	16.693321000	12.235741000
H	12.882366000	15.988293000	11.561462000
C	14.370082000	13.891992000	12.472759000
H	13.296883000	13.693326000	12.538600000
H	14.696817000	14.375392000	13.399552000
H	14.896144000	12.935851000	12.380210000
C	10.210443000	13.943948000	10.544491000
C	9.637922000	13.850286000	9.130490000
H	9.853546000	12.874835000	8.685355000
H	10.068981000	14.622567000	8.488168000
H	8.552665000	13.990956000	9.152831000
C	9.609473000	12.849955000	11.422128000
H	10.046610000	12.885299000	12.425042000
H	9.809451000	11.861462000	10.996206000
H	8.525497000	12.974691000	11.513141000
C	9.937329000	15.318576000	11.144824000
H	10.411094000	16.106531000	10.557361000
H	10.328659000	15.368484000	12.164543000
H	8.863870000	15.526092000	11.182924000
C	13.220730000	11.725906000	8.091842000
C	14.718729000	11.761329000	7.799506000
H	15.126770000	10.748000000	7.717080000
H	15.242177000	12.288488000	8.600810000
H	14.904703000	12.286501000	6.858217000
C	12.470327000	11.031722000	6.958595000
H	12.654184000	11.543889000	6.008557000
H	11.392727000	11.043295000	7.151088000
H	12.792935000	9.990584000	6.855438000
C	12.948777000	11.018034000	9.417123000
H	13.299200000	9.981608000	9.370430000
H	11.878996000	11.013126000	9.642393000
H	13.469355000	11.519419000	10.237754000
C	12.063900000	15.269426000	3.940125000
C	12.038936000	15.094411000	5.455416000
H	11.049281000	15.350990000	5.851259000
H	12.819305000	15.718725000	5.905272000
H	12.251544000	14.068588000	5.761547000
C	10.984236000	14.415359000	3.281880000
H	9.997007000	14.721797000	3.635109000
H	11.134151000	13.355474000	3.513236000
H	11.018251000	14.537944000	2.194422000
C	13.443736000	14.903884000	3.404236000
H	14.213972000	15.514420000	3.881259000
H	13.489796000	15.071704000	2.323852000
H	13.667256000	13.850297000	3.600821000
C	10.655143000	19.119913000	1.921171000
C	12.057379000	18.962087000	1.338713000
H	12.038217000	19.029626000	0.245663000
H	12.474973000	17.994599000	1.627081000
H	12.711278000	19.751352000	1.721536000
C	9.723633000	18.030757000	1.393989000
H	9.629800000	18.107491000	0.305674000
H	8.727634000	18.126750000	1.835160000
H	10.111690000	17.037701000	1.635646000
C	10.092294000	20.500034000	1.596192000
H	9.100649000	20.624804000	2.042601000
H	10.005640000	20.642256000	0.514121000
H	10.747415000	21.280941000	1.994482000
C	8.184612000	17.293151000	5.157512000
C	7.020539000	16.587380000	4.467876000
H	6.955092000	16.900254000	3.421279000

H	6.072689000	16.823378000	4.961975000
H	7.162081000	15.502021000	4.493082000
C	7.985681000	18.805710000	5.129632000
H	8.852655000	19.315883000	5.556715000
H	7.104499000	19.086747000	5.715251000
H	7.849581000	19.160170000	4.104079000
C	8.315873000	16.795163000	6.596168000
H	8.616655000	15.743056000	6.607152000
H	7.364749000	16.884100000	7.129090000
H	9.038674000	17.391743000	7.161906000
U	15.263027000	19.695412000	8.502223000
Si	17.607848000	18.058625000	6.121106000
Si	14.666027000	23.231349000	7.205007000
Si	16.319267000	19.971456000	12.123788000
O	16.855444000	18.591589000	7.456541000
O	17.328008000	16.433318000	5.814806000
O	17.179234000	18.830896000	4.703593000
O	19.233353000	18.372214000	6.344149000
O	14.910294000	21.732139000	7.773135000
O	13.278056000	23.462548000	6.301350000
O	15.920974000	23.734930000	6.213936000
O	14.494282000	24.251995000	8.516091000
O	15.669304000	19.741160000	10.657795000
O	15.682396000	21.270623000	12.965158000
O	16.002442000	18.619871000	13.054737000
O	17.968237000	20.263981000	12.072216000
C	17.157470000	15.406184000	6.802438000
C	18.153786000	15.589214000	7.945068000
H	17.957437000	16.524067000	8.476178000
H	18.077977000	14.764229000	8.660085000
H	19.177013000	15.618800000	7.558631000
C	15.722840000	15.449827000	7.318946000
H	15.550744000	14.712878000	8.105419000
H	15.500689000	16.439542000	7.726040000
H	15.019860000	15.248395000	6.504595000
C	17.424800000	14.090750000	6.077357000
H	18.451529000	14.063765000	5.697589000
H	17.281385000	13.239236000	6.749787000
H	16.743414000	13.979397000	5.227634000
C	16.006041000	18.591122000	3.908893000
C	14.807391000	18.217012000	4.776042000
H	14.604912000	18.996261000	5.513398000
H	13.909228000	18.087597000	4.168346000
H	14.996017000	17.283150000	5.310393000
C	16.318399000	17.473046000	2.917149000
H	16.569164000	16.557510000	3.458503000
H	15.460729000	17.277798000	2.264944000
H	17.170692000	17.751272000	2.287852000
C	15.735241000	19.900034000	3.175615000
H	16.600496000	20.183596000	2.567360000
H	14.865568000	19.805627000	2.518668000
H	15.541451000	20.699583000	3.893557000
C	20.292050000	18.284896000	5.385264000
C	20.093147000	17.108918000	4.430585000
H	19.177845000	17.236337000	3.847376000
H	20.937600000	17.038496000	3.736510000
H	20.016954000	16.168704000	4.983136000
C	20.352070000	19.597064000	4.605206000
H	20.521503000	20.435940000	5.287507000
H	21.165730000	19.580839000	3.871456000
H	19.406200000	19.759757000	4.082835000
C	21.567204000	18.088852000	6.201494000
H	21.516853000	17.154094000	6.769212000

H	22.449632000	18.053156000	5.553955000
H	21.692274000	18.912495000	6.911780000
C	12.859211000	22.818541000	5.088072000
C	11.435890000	23.311146000	4.846458000
H	11.419214000	24.400509000	4.736445000
H	11.015056000	22.862981000	3.941012000
H	10.794822000	23.040798000	5.690396000
C	12.875183000	21.305602000	5.261758000
H	12.234650000	21.001530000	6.091471000
H	12.514694000	20.795870000	4.364994000
H	13.888115000	20.962519000	5.479871000
C	13.768507000	23.240909000	3.936328000
H	14.802069000	22.952364000	4.137723000
H	13.444315000	22.771760000	3.001137000
H	13.743948000	24.327913000	3.805887000
C	17.316730000	23.462397000	6.405175000
C	17.694288000	23.513257000	7.884403000
H	17.445123000	24.487586000	8.313950000
H	17.155454000	22.743193000	8.440808000
H	18.767825000	23.340352000	8.010683000
C	18.055663000	24.553991000	5.635447000
H	17.750405000	24.549482000	4.584095000
H	17.827682000	25.539929000	6.053582000
H	19.139012000	24.400513000	5.681030000
C	17.636773000	22.091125000	5.817525000
H	17.059085000	21.309086000	6.313444000
H	17.396529000	22.064593000	4.751184000
H	18.697935000	21.852690000	5.934564000
C	13.956148000	25.577026000	8.542389000
C	12.434308000	25.479902000	8.641663000
H	11.978532000	26.474342000	8.705515000
H	12.039663000	24.963082000	7.763983000
H	12.151154000	24.914238000	9.534286000
C	14.528727000	26.233537000	9.796029000
H	14.257960000	25.655883000	10.685222000
H	15.620607000	26.283959000	9.737671000
H	14.142082000	27.250999000	9.916950000
C	14.363349000	26.372523000	7.302828000
H	13.975525000	27.395278000	7.362932000
H	15.452100000	26.419846000	7.213589000
H	13.964756000	25.903763000	6.399624000
C	15.350889000	22.546513000	12.390755000
C	13.985768000	22.437618000	11.717632000
H	14.024995000	21.697339000	10.916141000
H	13.226709000	22.126027000	12.440910000
H	13.687957000	23.395537000	11.282194000
C	16.407338000	22.987505000	11.381368000
H	16.462480000	22.276186000	10.554337000
H	16.147038000	23.961173000	10.959587000
H	17.392443000	23.048353000	11.851621000
C	15.295195000	23.520591000	13.562947000
H	14.559657000	23.184818000	14.301356000
H	16.271088000	23.584317000	14.055341000
H	15.011989000	24.522933000	13.225307000
C	16.122479000	18.488313000	14.475787000
C	14.813156000	18.956946000	15.106612000
H	14.836673000	18.852131000	16.196896000
H	14.633308000	20.004844000	14.853339000
H	13.983805000	18.358323000	14.720014000
C	17.300395000	19.292845000	15.022765000
H	17.393545000	19.136359000	16.102775000
H	18.234066000	18.985926000	14.544566000
H	17.157309000	20.361442000	14.840223000

C	16.337758000	17.000590000	14.740452000
H	17.270988000	16.660067000	14.281164000
H	16.388101000	16.796987000	15.815369000
H	15.513866000	16.417566000	14.318235000
C	18.928232000	19.678080000	11.184994000
C	20.266554000	19.758595000	11.915892000
H	20.231471000	19.177383000	12.843177000
H	21.077572000	19.367035000	11.293002000
H	20.497365000	20.797453000	12.173502000
C	18.592173000	18.221102000	10.869927000
H	17.635106000	18.146530000	10.348161000
H	19.362676000	17.783981000	10.227273000
H	18.530579000	17.633172000	11.789902000
C	18.978149000	20.511638000	9.906402000
H	19.206435000	21.554712000	10.144028000
H	19.738276000	20.134407000	9.215680000
H	18.015410000	20.481298000	9.389394000

[$^{Si}L_3UOU^{Si}L_3$] E = -5700.16858310

U	11.698017000	17.365310000	7.758159000
Si	9.489743000	19.100721000	10.238552000
Si	12.568318000	14.068821000	9.344812000
Si	10.744921000	17.670387000	4.323972000
O	13.362319000	18.535941000	8.150746000
O	10.362111000	18.318509000	9.079245000
O	9.727713000	20.744521000	10.184954000
O	9.873955000	18.624940000	11.780117000
O	7.919053000	18.651653000	9.946202000
O	12.292874000	15.571229000	8.733190000
O	14.043779000	13.906502000	10.087123000
O	11.424022000	13.664286000	10.483255000
O	12.562029000	13.049088000	8.034561000
O	11.211703000	18.155299000	5.824241000
O	11.813031000	16.606943000	3.612298000
O	10.710140000	19.031252000	3.380690000
O	9.291508000	16.855545000	4.399553000
C	9.938915000	21.580369000	9.033577000
C	9.154226000	21.064345000	7.831422000
H	9.522405000	20.084669000	7.519588000
H	9.258549000	21.748375000	6.984318000
H	8.091457000	20.973641000	8.073896000
C	11.433843000	21.610166000	8.741083000
H	11.669388000	22.272900000	7.906011000
H	11.788503000	20.605494000	8.494557000
H	11.979305000	21.963021000	9.620191000
C	9.443730000	22.964965000	9.436012000
H	8.372706000	22.937838000	9.660356000
H	9.611400000	23.686400000	8.630692000
H	9.972577000	23.312005000	10.328872000
C	11.035633000	19.007834000	12.544460000
C	12.268847000	19.121711000	11.651759000
H	12.439199000	18.190370000	11.108916000
H	13.159112000	19.330922000	12.250061000
H	12.149653000	19.930085000	10.926628000
C	10.743789000	20.337417000	13.234101000
H	10.549960000	21.112736000	12.489304000
H	11.592771000	20.642662000	13.854666000
H	9.863914000	20.245764000	13.879336000
C	11.225961000	17.901343000	13.574943000
H	10.310688000	17.766618000	14.159936000
H	12.040646000	18.150967000	14.260591000
H	11.465894000	16.954482000	13.084884000
C	6.773372000	18.872639000	10.786614000

C	5.569731000	18.854556000	9.849937000
H	5.642105000	19.662651000	9.115060000
H	4.638029000	18.979915000	10.410806000
H	5.521063000	17.903375000	9.310530000
C	6.863705000	20.215208000	11.507918000
H	7.722635000	20.238507000	12.184513000
H	5.959169000	20.384683000	12.100956000
H	6.968449000	21.034988000	10.792548000
C	6.691485000	17.728219000	11.793703000
H	6.603950000	16.769793000	11.272432000
H	5.819547000	17.844006000	12.446309000
H	7.593668000	17.708609000	12.410253000
C	14.467879000	14.546277000	11.308906000
C	15.987017000	14.622911000	11.228389000
H	16.410437000	13.630102000	11.045927000
H	16.403289000	15.011995000	12.161673000
H	16.291134000	15.288332000	10.416955000
C	13.878861000	15.947454000	11.417768000
H	14.163340000	16.550722000	10.552383000
H	14.248045000	16.452135000	12.314087000
H	12.787531000	15.905980000	11.467816000
C	14.023112000	13.677089000	12.480858000
H	12.934417000	13.581339000	12.483505000
H	14.343052000	14.115619000	13.432034000
H	14.458929000	12.675943000	12.399772000
C	10.010440000	13.926587000	10.446330000
C	9.472921000	13.912327000	9.016509000
H	9.667013000	12.951433000	8.531643000
H	9.942704000	14.697863000	8.417765000
H	8.393249000	14.090175000	9.018392000
C	9.362540000	12.809789000	11.258192000
H	9.773125000	12.791015000	12.272464000
H	9.550712000	11.836919000	10.793451000
H	8.280008000	12.957496000	11.326314000
C	9.760219000	15.278575000	11.101907000
H	10.261258000	16.080903000	10.558180000
H	10.134323000	15.276333000	12.129216000
H	8.691599000	15.508110000	11.128467000
C	12.984048000	11.675767000	7.977753000
C	14.490345000	11.647820000	7.733482000
H	14.847576000	10.618871000	7.619280000
H	15.014870000	12.110652000	8.573048000
H	14.734515000	12.200543000	6.821714000
C	12.236425000	11.067922000	6.795104000
H	12.485231000	11.597833000	5.869987000
H	11.155232000	11.136581000	6.950945000
H	12.501141000	10.013566000	6.666578000
C	12.632449000	10.935413000	9.265358000
H	12.939953000	9.887111000	9.192869000
H	11.555625000	10.967779000	9.451472000
H	13.143537000	11.382382000	10.122858000
C	12.018969000	15.221091000	3.938559000
C	11.975360000	15.014817000	5.449106000
H	10.981147000	15.262370000	5.840570000
H	12.757521000	15.621673000	5.920225000
H	12.178965000	13.982738000	5.737980000
C	10.946991000	14.383030000	3.249548000
H	9.954891000	14.678843000	3.597835000
H	11.096465000	13.318773000	3.458826000
H	10.993411000	14.529849000	2.165895000
C	13.404819000	14.867866000	3.410750000
H	14.169012000	15.477252000	3.899387000
H	13.458321000	15.046865000	2.332703000

H	13.630745000	13.813282000	3.597808000
C	10.653558000	19.100482000	1.943571000
C	12.073237000	18.954183000	1.402700000
H	12.086380000	19.046873000	0.311722000
H	12.483073000	17.980041000	1.679748000
H	12.715167000	19.734751000	1.822203000
C	9.739704000	18.016706000	1.378328000
H	9.674100000	18.108807000	0.289424000
H	8.732219000	18.104776000	1.794353000
H	10.123754000	17.020379000	1.613706000
C	10.097818000	20.483944000	1.625163000
H	9.094638000	20.602049000	2.046540000
H	10.040279000	20.640662000	0.543405000
H	10.742184000	21.259110000	2.050868000
C	8.108746000	17.228569000	5.126773000
C	6.953206000	16.544024000	4.403950000
H	6.905637000	16.881293000	3.364216000
H	5.999745000	16.775132000	4.888992000
H	7.088328000	15.457718000	4.407220000
C	7.919756000	18.742068000	5.127891000
H	8.783683000	19.240457000	5.574783000
H	7.034290000	19.015350000	5.710426000
H	7.794715000	19.118324000	4.108845000
C	8.221114000	16.695279000	6.553497000
H	8.510197000	15.640632000	6.539596000
H	7.266975000	16.782833000	7.081237000
H	8.946693000	17.271599000	7.138474000
U	15.054751000	19.710821000	8.453154000
Si	17.449962000	17.960006000	6.327230000
Si	14.494276000	23.190978000	7.102042000
Si	16.358672000	20.007763000	11.939878000
O	16.536605000	18.458297000	7.606914000
O	17.191976000	16.353925000	5.979258000
O	17.095400000	18.797413000	4.936819000
O	19.011913000	18.316870000	6.752286000
O	14.725895000	21.675960000	7.708451000
O	13.101654000	23.376060000	6.217775000
O	15.749555000	23.609372000	6.090768000
O	14.348327000	24.188645000	8.420309000
O	15.607163000	19.768707000	10.490559000
O	15.695147000	21.258533000	12.808764000
O	16.131175000	18.618390000	12.819209000
O	17.968744000	20.375299000	11.734606000
C	17.020034000	15.278086000	6.920996000
C	18.000573000	15.423973000	8.080067000
H	17.791131000	16.332477000	8.650547000
H	17.921425000	14.569745000	8.758715000
H	19.028863000	15.477114000	7.711263000
C	15.579991000	15.290473000	7.419873000
H	15.399309000	14.494248000	8.143429000
H	15.353036000	16.242851000	7.903730000
H	14.889334000	15.155064000	6.582466000
C	17.307668000	14.002237000	6.137894000
H	18.335724000	14.007325000	5.761815000
H	17.174432000	13.120867000	6.772401000
H	16.630853000	13.918624000	5.281696000
C	15.976174000	18.565684000	4.057232000
C	14.731975000	18.160046000	4.841715000
H	14.444843000	18.940647000	5.550071000
H	13.884851000	18.000492000	4.171547000
H	14.907254000	17.234067000	5.394509000
C	16.362987000	17.474742000	3.063183000
H	16.575607000	16.542630000	3.591875000

H	15.554302000	17.298919000	2.346431000
H	17.257435000	17.771645000	2.505910000
C	15.741620000	19.890548000	3.341947000
H	16.637643000	20.190115000	2.789258000
H	14.909575000	19.809483000	2.636491000
H	15.505943000	20.673822000	4.065439000
C	20.188907000	18.245704000	5.928800000
C	20.106630000	17.090503000	4.934308000
H	19.272424000	17.229640000	4.240797000
H	21.030025000	17.033788000	4.348926000
H	19.966224000	16.137974000	5.451847000
C	20.336057000	19.574220000	5.192448000
H	20.413150000	20.397228000	5.909155000
H	21.236604000	19.576891000	4.569240000
H	19.464962000	19.749026000	4.555800000
C	21.348817000	18.031177000	6.895253000
H	21.229524000	17.084865000	7.432122000
H	22.303227000	18.007068000	6.359549000
H	21.385701000	18.840964000	7.630453000
C	12.669658000	22.725657000	5.007186000
C	11.237413000	23.201578000	4.793031000
H	11.207030000	24.290523000	4.685613000
H	10.808336000	22.751242000	3.893028000
H	10.612791000	22.922020000	5.646384000
C	12.711204000	21.215309000	5.188624000
H	12.093177000	20.905603000	6.033024000
H	12.338012000	20.694816000	4.303715000
H	13.735423000	20.889080000	5.378370000
C	13.556276000	23.159535000	3.843520000
H	14.595583000	22.875896000	4.020999000
H	13.216541000	22.693450000	2.912828000
H	13.520966000	24.246443000	3.718412000
C	17.159966000	23.484558000	6.344137000
C	17.488044000	23.826267000	7.793601000
H	17.134530000	24.828906000	8.047181000
H	17.014576000	23.116477000	8.475299000
H	18.569257000	23.785485000	7.958180000
C	17.829232000	24.475864000	5.397939000
H	17.560908000	24.250575000	4.361011000
H	17.504631000	25.496633000	5.623020000
H	18.919017000	24.429875000	5.490688000
C	17.599305000	22.061497000	6.017915000
H	17.091274000	21.338025000	6.658693000
H	17.365890000	21.810682000	4.981295000
H	18.675609000	21.944055000	6.168600000
C	13.823755000	25.526723000	8.451412000
C	12.303257000	25.442706000	8.561486000
H	11.860759000	26.441961000	8.634541000
H	11.893088000	24.937117000	7.684389000
H	12.021743000	24.876511000	9.453986000
C	14.414206000	26.174192000	9.699726000
H	14.137231000	25.604475000	10.591870000
H	15.506163000	26.208019000	9.636098000
H	14.043262000	27.197362000	9.817557000
C	14.234222000	26.309780000	7.206565000
H	13.861776000	27.337713000	7.266883000
H	15.323044000	26.343130000	7.110301000
H	13.820819000	25.848655000	6.305583000
C	15.325352000	22.556237000	12.302490000
C	13.918716000	22.452993000	11.723377000
H	13.911290000	21.741773000	10.895157000
H	13.214192000	22.103260000	12.482785000
H	13.579303000	23.420447000	11.343648000

C	16.306694000	23.031431000	11.237384000
H	16.275673000	22.370187000	10.368467000
H	16.036104000	24.031565000	10.892492000
H	17.327640000	23.046096000	11.626147000
C	15.348075000	23.489026000	13.507070000
H	14.670212000	23.122838000	14.284617000
H	16.356667000	23.545632000	13.928478000
H	15.034350000	24.498305000	13.222183000
C	16.341239000	18.450279000	14.233700000
C	15.063707000	18.873090000	14.953803000
H	15.156633000	18.729041000	16.035448000
H	14.850989000	19.926131000	14.753403000
H	14.220659000	18.273907000	14.598092000
C	17.533934000	19.270772000	14.718974000
H	17.704874000	19.089864000	15.785105000
H	18.440264000	18.998404000	14.171605000
H	17.354127000	20.340657000	14.579640000
C	16.607380000	16.963382000	14.440699000
H	17.502388000	16.654426000	13.892088000
H	16.758747000	16.741188000	15.501866000
H	15.760637000	16.370628000	14.082841000
C	18.884243000	19.813395000	10.778200000
C	20.271243000	19.974383000	11.391827000
H	20.347194000	19.397467000	12.318893000
H	21.043841000	19.622245000	10.700822000
H	20.465661000	21.026237000	11.623697000
C	18.592969000	18.337023000	10.525468000
H	17.602103000	18.199298000	10.088154000
H	19.322116000	17.926908000	9.821879000
H	18.645526000	17.769315000	11.458595000
C	18.782806000	20.621672000	9.487038000
H	19.063558000	21.663975000	9.662155000
H	19.414520000	20.200012000	8.701852000
H	17.756471000	20.610769000	9.114715000

[$^{Si}L_3UNU^{Si}L_3$]$^{3-}$ E = -5679.78327838

U	11.696131000	17.711271000	7.791339000
Si	9.381311000	19.340565000	10.390771000
Si	12.511973000	14.202257000	9.119296000
Si	10.892525000	17.550244000	4.081511000
N	13.454896000	18.687408000	8.301126000
O	10.108311000	18.583659000	9.179119000
O	9.614114000	21.006289000	10.444116000
O	9.777731000	18.849507000	11.947537000
O	7.731619000	19.012059000	10.265581000
O	12.060175000	15.549653000	8.383600000
O	14.015980000	14.177796000	9.864016000
O	11.445885000	13.728863000	10.342393000
O	12.604123000	12.975618000	7.968354000
O	11.294381000	18.036975000	5.551180000
O	11.955452000	16.511436000	3.291592000
O	10.823473000	18.896141000	3.073956000
O	9.419651000	16.725399000	4.003001000
C	9.854819000	21.894214000	9.348587000
C	9.039107000	21.484425000	8.122908000
H	9.389393000	20.525782000	7.731387000
H	9.124645000	22.236552000	7.332541000
H	7.981636000	21.383031000	8.389080000
C	11.347088000	21.910999000	9.040655000
H	11.591312000	22.604786000	8.233785000
H	11.706225000	20.919279000	8.748655000
H	11.910427000	22.217038000	9.926665000
C	9.401749000	23.270999000	9.834245000

H	8.328875000	23.269074000	10.057521000
H	9.597122000	24.037137000	9.076518000
H	9.938830000	23.545806000	10.748020000
C	10.995010000	19.159846000	12.641569000
C	12.178500000	19.247495000	11.683233000
H	12.236991000	18.344363000	11.077126000
H	13.121325000	19.356524000	12.225108000
H	12.081603000	20.091857000	10.998160000
C	10.804098000	20.479814000	13.387531000
H	10.587107000	21.274337000	12.669515000
H	11.706172000	20.741761000	13.951688000
H	9.968319000	20.407619000	14.093723000
C	11.201840000	18.015094000	13.629118000
H	10.321772000	17.901137000	14.272047000
H	12.074482000	18.197785000	14.262853000
H	11.361284000	17.076797000	13.092183000
C	6.711724000	19.213610000	11.237893000
C	5.400391000	19.275474000	10.455233000
H	5.414786000	20.116686000	9.754144000
H	4.541310000	19.397275000	11.124441000
H	5.261899000	18.356341000	9.876514000
C	6.913909000	20.512809000	12.018873000
H	7.853297000	20.478593000	12.575356000
H	6.091573000	20.664896000	12.727262000
H	6.953588000	21.369814000	11.341541000
C	6.701866000	18.020574000	12.196123000
H	6.543222000	17.091952000	11.638088000
H	5.904709000	18.113703000	12.943175000
H	7.666100000	17.955737000	12.706111000
C	14.405569000	14.914077000	11.033663000
C	15.877706000	15.270138000	10.855847000
H	16.474986000	14.371789000	10.662446000
H	16.266030000	15.760871000	11.752654000
H	15.991305000	15.960543000	10.016695000
C	13.604354000	16.198223000	11.185481000
H	13.716522000	16.830809000	10.297766000
H	13.959714000	16.764027000	12.051287000
H	12.542737000	15.977505000	11.323449000
C	14.201239000	14.000894000	12.241123000
H	13.145632000	13.725667000	12.317350000
H	14.505053000	14.499181000	13.168381000
H	14.792655000	13.084292000	12.134161000
C	10.035473000	13.973055000	10.360777000
C	9.416236000	13.788395000	8.974892000
H	9.618344000	12.783280000	8.590687000
H	9.832669000	14.520958000	8.278095000
H	8.330843000	13.930796000	9.018992000
C	9.457742000	12.944752000	11.332129000
H	9.924444000	13.048352000	12.317486000
H	9.646778000	11.928081000	10.970764000
H	8.376131000	13.075151000	11.447575000
C	9.776851000	15.388914000	10.872093000
H	10.211097000	16.131955000	10.199768000
H	10.216640000	15.517448000	11.865367000
H	8.703352000	15.591942000	10.948833000
C	13.243159000	11.707625000	8.066139000
C	14.740904000	11.882924000	7.810056000
H	15.258663000	10.916434000	7.812813000
H	15.171613000	12.522152000	8.583960000
H	14.903056000	12.360401000	6.839019000
C	12.618649000	10.844756000	6.970220000
H	12.763108000	11.312639000	5.990941000
H	11.542134000	10.735135000	7.137623000

H	13.068542000	9.846018000	6.945782000
C	13.011590000	11.064287000	9.433635000
H	13.487240000	10.078019000	9.479340000
H	11.942323000	10.944263000	9.628885000
H	13.433266000	11.691716000	10.222555000
C	12.233746000	15.150234000	3.644379000
C	12.195965000	14.961083000	5.153985000
H	11.197861000	15.159758000	5.552850000
H	12.891049000	15.651350000	5.635869000
H	12.472775000	13.945924000	5.449065000
C	11.207006000	14.251913000	2.955307000
H	10.201694000	14.529156000	3.281747000
H	11.383617000	13.197343000	3.196012000
H	11.262514000	14.369931000	1.866981000
C	13.638465000	14.858460000	3.123644000
H	14.369277000	15.487030000	3.637923000
H	13.701817000	15.059867000	2.048693000
H	13.907461000	13.810268000	3.294855000
C	10.831245000	18.942868000	1.650890000
C	12.279253000	18.859600000	1.166111000
H	12.340389000	18.927978000	0.073636000
H	12.718647000	17.913944000	1.491163000
H	12.861451000	19.680067000	1.596352000
C	10.000930000	17.813526000	1.040126000
H	9.983482000	17.898850000	-0.052485000
H	8.972486000	17.849155000	1.409248000
H	10.425337000	16.843337000	1.309552000
C	10.229271000	20.296540000	1.275915000
H	9.198978000	20.371584000	1.638960000
H	10.225062000	20.441756000	0.189892000
H	10.809336000	21.106207000	1.730411000
C	8.233238000	17.003720000	4.750031000
C	7.069000000	16.610984000	3.840693000
H	7.063954000	17.229259000	2.936495000
H	6.108389000	16.740009000	4.351568000
H	7.159644000	15.562717000	3.536601000
C	8.128318000	18.482198000	5.125103000
H	8.965464000	18.770830000	5.766771000
H	7.194392000	18.676498000	5.663273000
H	8.150783000	19.109336000	4.228544000
C	8.239289000	16.138384000	6.008163000
H	8.335868000	15.079796000	5.744643000
H	7.318684000	16.270732000	6.587656000
H	9.084959000	16.413683000	6.651536000
U	15.246253000	19.671853000	8.646569000
Si	17.585291000	18.199979000	5.961963000
Si	14.611059000	23.267862000	7.501318000
Si	16.360394000	19.735034000	12.269383000
O	16.764120000	18.810338000	7.195484000
O	17.352718000	16.549296000	5.716166000
O	17.286828000	18.872542000	4.452947000
O	19.221684000	18.498305000	6.236824000
O	14.872896000	21.852404000	8.199182000
O	13.193471000	23.443768000	6.614188000
O	15.809884000	23.718832000	6.402286000
O	14.491250000	24.437442000	8.704703000
O	15.683922000	19.363612000	10.868156000
O	15.796763000	21.119284000	13.044107000
O	16.051717000	18.488292000	13.359545000
O	18.027293000	19.992708000	12.217041000
C	17.106998000	15.584745000	6.747415000
C	17.983219000	15.873111000	7.964495000
H	17.696515000	16.829647000	8.408056000

H	17.870760000	15.090729000	8.720613000
H	19.036934000	15.924077000	7.672553000
C	15.628203000	15.604571000	7.119340000
H	15.399162000	14.885242000	7.908736000
H	15.314564000	16.594175000	7.471611000
H	15.017739000	15.354172000	6.246454000
C	17.481182000	14.233574000	6.141056000
H	18.539828000	14.216341000	5.858101000
H	17.298369000	13.421870000	6.852789000
H	16.884308000	14.043409000	5.242739000
C	16.138046000	18.614158000	3.632607000
C	14.902423000	18.297991000	4.469189000
H	14.688574000	19.090388000	5.187918000
H	14.024361000	18.160742000	3.833592000
H	15.046579000	17.376532000	5.036422000
C	16.469042000	17.440123000	2.711353000
H	16.684952000	16.554382000	3.314418000
H	15.632123000	17.218211000	2.040643000
H	17.348535000	17.669720000	2.098720000
C	15.919339000	19.882017000	2.811783000
H	16.825294000	20.134727000	2.249583000
H	15.097566000	19.747989000	2.101451000
H	15.672453000	20.722938000	3.464482000
C	20.305799000	18.381213000	5.321107000
C	21.555455000	18.187411000	6.179099000
H	21.476169000	17.264525000	6.763082000
H	22.458699000	18.129403000	5.561548000
H	21.666455000	19.022791000	6.878044000
C	20.132913000	17.190542000	4.377391000
H	19.235891000	17.317510000	3.767579000
H	20.999894000	17.101099000	3.712903000
H	20.028566000	16.261088000	4.943147000
C	20.410814000	19.678662000	4.518121000
H	20.564484000	20.527730000	5.192249000
H	21.248258000	19.643975000	3.811302000
H	19.482843000	19.839669000	3.964152000
C	12.884137000	22.790889000	5.374529000
C	11.364209000	22.678157000	5.312353000
H	10.896472000	23.653989000	5.484202000
H	11.044395000	22.304472000	4.334090000
H	11.009202000	21.974195000	6.068325000
C	13.496830000	21.398080000	5.302170000
H	13.184849000	20.775596000	6.146836000
H	13.182087000	20.899017000	4.381264000
H	14.588537000	21.453559000	5.309352000
C	13.405061000	23.663514000	4.232882000
H	14.485189000	23.793881000	4.334965000
H	13.190079000	23.204687000	3.261088000
H	12.931312000	24.651616000	4.257658000
C	17.206797000	23.417105000	6.461431000
C	17.720759000	23.388975000	7.900758000
H	17.506027000	24.334780000	8.408373000
H	17.241764000	22.577041000	8.453000000
H	18.803729000	23.225274000	7.916439000
C	17.899351000	24.532644000	5.678870000
H	17.505743000	24.581089000	4.658002000
H	17.727700000	25.502956000	6.157127000
H	18.980069000	24.362045000	5.623077000
C	17.454725000	22.071815000	5.785922000
H	16.943848000	21.258936000	6.308445000
H	17.097555000	22.093676000	4.752386000
H	18.522839000	21.833486000	5.770084000
C	13.969840000	25.759149000	8.608617000

C	12.445186000	25.699213000	8.718022000
H	12.006545000	26.703924000	8.711656000
H	12.040494000	25.127374000	7.879942000
H	12.154305000	25.200939000	9.647616000
C	14.555763000	26.523341000	9.795121000
H	14.275501000	26.037134000	10.734880000
H	15.648878000	26.542590000	9.733459000
H	14.192187000	27.556646000	9.819718000
C	14.381276000	26.434021000	7.299874000
H	13.996363000	27.459361000	7.259222000
H	15.470277000	26.466877000	7.208888000
H	13.985927000	25.877906000	6.446620000
C	15.509287000	22.376812000	12.417064000
C	14.113600000	22.311896000	11.803429000
H	14.080599000	21.525140000	11.047386000
H	13.366067000	22.087847000	12.570427000
H	13.856663000	23.255729000	11.313975000
C	16.537979000	22.719874000	11.341355000
H	16.511493000	21.976349000	10.539548000
H	16.303003000	23.688304000	10.890884000
H	17.546801000	22.746745000	11.762511000
C	15.556996000	23.412398000	13.538055000
H	14.839160000	23.157957000	14.325326000
H	16.556492000	23.447550000	13.985127000
H	15.312929000	24.410812000	13.159485000
C	16.175698000	18.504048000	14.778121000
C	14.906977000	19.114748000	15.374328000
H	14.949807000	19.135159000	16.469507000
H	14.780096000	20.133120000	14.999566000
H	14.034864000	18.526448000	15.075362000
C	17.405296000	19.290996000	15.232305000
H	17.503640000	19.250519000	16.323102000
H	18.312688000	18.878572000	14.783338000
H	17.321647000	20.337614000	14.928054000
C	16.306856000	17.041247000	15.199277000
H	17.210936000	16.599101000	14.767767000
H	16.359962000	16.944331000	16.289414000
H	15.444307000	16.468933000	14.843311000
C	18.976678000	19.374633000	11.347765000
C	20.311847000	19.426632000	12.090389000
H	20.256821000	18.849664000	13.019989000
H	21.120782000	19.015012000	11.476833000
H	20.564476000	20.461077000	12.347056000
C	18.602195000	17.923807000	11.047158000
H	17.638740000	17.882193000	10.534293000
H	19.356455000	17.453505000	10.407833000
H	18.524199000	17.348809000	11.975075000
C	19.060878000	20.185688000	10.055945000
H	19.334827000	21.222668000	10.276858000
H	19.805190000	19.766790000	9.370636000
H	18.091015000	20.190558000	9.541936000

$[^{Si}L_3UNU^{Si}L_3]^{2-}$ E = -5679.77853291

U	11.882915000	17.692643000	7.767753000
Si	9.644113000	19.146537000	10.432374000
Si	12.304201000	14.016880000	8.767713000
Si	10.733254000	17.743910000	4.178705000
N	13.521680000	18.604154000	8.323188000
O	10.347873000	18.568823000	9.095558000
O	9.871389000	20.792765000	10.654230000
O	10.127526000	18.452305000	11.871676000
O	8.017037000	18.769808000	10.282461000
O	12.065311000	15.516961000	8.222895000

O	13.685276000	13.756188000	9.679179000
O	11.037217000	13.468168000	9.724245000
O	12.483899000	13.011006000	7.442593000
O	11.293042000	18.102307000	5.649881000
O	11.409285000	16.398037000	3.448849000
O	11.080813000	19.026967000	3.162226000
O	9.080556000	17.438945000	4.174842000
C	10.023788000	21.780957000	9.623533000
C	9.076900000	21.495963000	8.458944000
H	9.334438000	20.547718000	7.981443000
H	9.137738000	22.289858000	7.708439000
H	8.042661000	21.435673000	8.812766000
C	11.476349000	21.795498000	9.159899000
H	11.641572000	22.518693000	8.358785000
H	11.778374000	20.811281000	8.790298000
H	12.139413000	22.053780000	9.991760000
C	9.662114000	23.112170000	10.276765000
H	8.623466000	23.101380000	10.624592000
H	9.782226000	23.938178000	9.568583000
H	10.310447000	23.299225000	11.138867000
C	11.318217000	18.738786000	12.624148000
C	12.490814000	19.101277000	11.717750000
H	12.714034000	18.305062000	11.005577000
H	13.392696000	19.289990000	12.305905000
H	12.271324000	20.004267000	11.144042000
C	11.003992000	19.886386000	13.581445000
H	10.730887000	20.777395000	13.010366000
H	11.867951000	20.119180000	14.212433000
H	10.164619000	19.618366000	14.232909000
C	11.624728000	17.461754000	13.399155000
H	10.772755000	17.180084000	14.027397000
H	12.499685000	17.598760000	14.041561000
H	11.829997000	16.640612000	12.707973000
C	6.983113000	18.908865000	11.257998000
C	5.680422000	18.985464000	10.464661000
H	5.686574000	19.858988000	9.804543000
H	4.813787000	19.060270000	11.130314000
H	5.563946000	18.091625000	9.843154000
C	7.162425000	20.173873000	12.096083000
H	8.093095000	20.129640000	12.667316000
H	6.329480000	20.283555000	12.799225000
H	7.199939000	21.060887000	11.458684000
C	6.984936000	17.676057000	12.162395000
H	6.812764000	16.770282000	11.572249000
H	6.197328000	17.743957000	12.921458000
H	7.953512000	17.585294000	12.660095000
C	14.072101000	14.366955000	10.919415000
C	15.500713000	13.893080000	11.168265000
H	15.531978000	12.801747000	11.257253000
H	15.904525000	14.329853000	12.087081000
H	16.146722000	14.186899000	10.337278000
C	14.020557000	15.886291000	10.802498000
H	14.635085000	16.243834000	9.973383000
H	14.376241000	16.362268000	11.720744000
H	12.995933000	16.218882000	10.620123000
C	13.160511000	13.883849000	12.045856000
H	12.129432000	14.194761000	11.868725000
H	13.493468000	14.290631000	13.006819000
H	13.175863000	12.790637000	12.108461000
C	9.701685000	13.982080000	9.792185000
C	9.118622000	14.180388000	8.394310000
H	9.175375000	13.251200000	7.818501000
H	9.673626000	14.954205000	7.861622000

H	8.069323000	14.487871000	8.454740000
C	8.900911000	12.926786000	10.551168000
H	9.349351000	12.745955000	11.533439000
H	8.892063000	11.980727000	9.999475000
H	7.865677000	13.252174000	10.698891000
C	9.702111000	15.300175000	10.560623000
H	10.347115000	16.032454000	10.073788000
H	10.059047000	15.152466000	11.584057000
H	8.698170000	15.729559000	10.610731000
C	13.082807000	11.716396000	7.368686000
C	14.600178000	11.881160000	7.275244000
H	15.096127000	10.909787000	7.167828000
H	14.974107000	12.373787000	8.175436000
H	14.858860000	12.498411000	6.409687000
C	12.536812000	11.085654000	6.089758000
H	12.782103000	11.710272000	5.224876000
H	11.447457000	10.991067000	6.145645000
H	12.963163000	10.090011000	5.927255000
C	12.707042000	10.862470000	8.578891000
H	13.136613000	9.858635000	8.489478000
H	11.620428000	10.769303000	8.661339000
H	13.083660000	11.320859000	9.496768000
C	11.645280000	15.116993000	4.055686000
C	12.999177000	15.143028000	4.757629000
H	13.010068000	15.910298000	5.533204000
H	13.798956000	15.363841000	4.045260000
H	13.196927000	14.187904000	5.249594000
C	10.545410000	14.753286000	5.048414000
H	10.502373000	15.487146000	5.855758000
H	10.762374000	13.785838000	5.507502000
H	9.569218000	14.721965000	4.557688000
C	11.666121000	14.115886000	2.904517000
H	12.436050000	14.389931000	2.175820000
H	10.698441000	14.099739000	2.392298000
H	11.880964000	13.106262000	3.270030000
C	11.086436000	19.042435000	1.732779000
C	12.436150000	18.512471000	1.251109000
H	12.503632000	18.533300000	0.157611000
H	12.576372000	17.485852000	1.597970000
H	13.241403000	19.129384000	1.659412000
C	9.943707000	18.206827000	1.157356000
H	9.950056000	18.257340000	0.062976000
H	8.977350000	18.572361000	1.515731000
H	10.046450000	17.160214000	1.455964000
C	10.921641000	20.506667000	1.333691000
H	9.967931000	20.899759000	1.700159000
H	10.947982000	20.623344000	0.244905000
H	11.729024000	21.107118000	1.763893000
C	8.095523000	18.087601000	4.987152000
C	6.771781000	17.899092000	4.250055000
H	6.815401000	18.374694000	3.264791000
H	5.941591000	18.339822000	4.811924000
H	6.566166000	16.833244000	4.105686000
C	8.405364000	19.573884000	5.153492000
H	9.369385000	19.701420000	5.650932000
H	7.636661000	20.063079000	5.760540000
H	8.447792000	20.069957000	4.179499000
C	8.038705000	17.403314000	6.352572000
H	7.892664000	16.325893000	6.232381000
H	7.216701000	17.800641000	6.957007000
H	8.962958000	17.572288000	6.910051000
U	15.311271000	19.768865000	8.588913000
Si	17.763274000	18.132920000	6.165820000

Si	14.518302000	23.216668000	7.123564000
Si	16.560393000	19.888107000	12.109776000
O	16.830023000	18.921523000	7.214547000
O	17.710392000	16.457633000	6.295730000
O	17.440957000	18.480862000	4.560421000
O	19.335689000	18.670780000	6.380707000
O	14.938414000	21.841749000	7.843168000
O	13.014237000	23.241406000	6.387010000
O	15.592943000	23.674373000	5.910641000
O	14.443368000	24.410751000	8.299786000
O	16.133299000	19.367615000	10.649686000
O	15.474110000	20.900997000	12.892773000
O	16.690358000	18.547667000	13.108222000
O	18.005852000	20.754678000	12.106191000
C	17.270018000	15.662454000	7.407699000
C	17.767609000	16.247173000	8.728107000
H	17.309908000	17.220672000	8.913450000
H	17.516273000	15.588968000	9.564437000
H	18.854030000	16.373815000	8.706807000
C	15.747727000	15.577557000	7.388735000
H	15.371149000	14.921625000	8.177215000
H	15.302983000	16.566491000	7.534758000
H	15.404380000	15.182156000	6.428182000
C	17.872366000	14.277561000	7.182611000
H	18.966774000	14.321839000	7.199424000
H	17.542849000	13.579700000	7.959215000
H	17.561680000	13.881636000	6.210288000
C	16.288807000	18.085032000	3.808906000
C	15.026074000	18.117647000	4.664878000
H	14.925076000	19.084544000	5.158041000
H	14.138361000	17.942791000	4.051834000
H	15.056112000	17.352235000	5.442228000
C	16.527117000	16.681620000	3.252289000
H	16.666009000	15.976768000	4.075312000
H	15.679043000	16.354226000	2.640787000
H	17.427923000	16.664439000	2.628677000
C	16.175089000	19.094399000	2.669523000
H	17.123813000	19.162409000	2.126534000
H	15.392552000	18.798789000	1.964372000
H	15.929405000	20.086263000	3.060693000
C	20.548944000	18.122181000	5.868099000
C	21.094299000	17.121304000	6.885978000
H	20.404322000	16.279417000	6.985220000
H	22.072469000	16.734656000	6.578440000
H	21.202764000	17.597076000	7.865653000
C	20.348610000	17.434442000	4.517485000
H	19.926504000	18.130127000	3.788776000
H	21.306892000	17.061686000	4.138739000
H	19.663945000	16.587766000	4.618921000
C	21.503665000	19.305640000	5.719045000
H	21.627147000	19.814651000	6.680612000
H	22.489549000	18.981268000	5.368533000
H	21.100093000	20.027574000	5.001644000
C	12.623443000	22.514926000	5.211128000
C	11.146947000	22.177096000	5.386051000
H	10.556585000	23.084823000	5.551725000
H	10.764084000	21.661035000	4.500976000
H	11.018365000	21.515261000	6.245698000
C	13.413939000	21.222100000	5.051045000
H	13.320290000	20.604017000	5.948236000
H	13.023217000	20.644853000	4.208507000
H	14.474109000	21.428681000	4.882585000
C	12.838752000	23.429840000	4.007793000

H	13.897280000	23.692699000	3.930028000
H	12.526654000	22.939133000	3.079699000
H	12.258640000	24.352525000	4.119634000
C	17.007282000	23.439013000	5.907955000
C	17.604583000	23.594797000	7.306416000
H	17.411211000	24.596149000	7.703546000
H	17.166627000	22.859338000	7.985842000
H	18.687746000	23.436451000	7.276868000
C	17.592884000	24.490193000	4.967796000
H	17.144910000	24.402415000	3.972366000
H	17.389277000	25.497688000	5.345764000
H	18.677167000	24.369993000	4.870051000
C	17.277065000	22.034953000	5.371326000
H	16.847098000	21.276021000	6.028474000
H	16.838335000	21.922468000	4.375204000
H	18.352564000	21.844073000	5.292640000
C	13.813910000	25.689022000	8.240382000
C	12.324185000	25.516227000	8.537691000
H	11.812034000	26.484646000	8.572274000
H	11.864168000	24.897268000	7.763892000
H	12.191350000	25.019227000	9.503331000
C	14.477775000	26.527546000	9.331163000
H	14.355353000	26.047237000	10.307380000
H	15.549873000	26.629987000	9.134453000
H	14.038247000	27.529549000	9.382306000
C	14.012635000	26.350093000	6.877077000
H	13.552052000	27.344094000	6.863514000
H	15.077042000	26.457292000	6.650675000
H	13.554451000	25.744791000	6.091046000
C	15.177066000	22.265217000	12.558550000
C	15.211332000	22.484417000	11.051592000
H	16.208907000	22.293086000	10.646347000
H	14.501219000	21.819498000	10.552212000
H	14.935213000	23.506372000	10.781135000
C	16.190952000	23.167812000	13.259360000
H	17.199831000	22.919822000	12.920877000
H	15.990742000	24.222977000	13.042199000
H	16.144132000	23.025333000	14.344657000
C	13.768323000	22.528454000	13.082864000
H	13.046924000	21.888124000	12.569015000
H	13.712044000	22.322934000	14.157023000
H	13.480877000	23.572093000	12.915639000
C	16.710694000	18.495338000	14.533959000
C	15.271207000	18.555286000	15.044506000
H	15.232163000	18.475088000	16.136805000
H	14.810905000	19.497392000	14.738114000
H	14.693404000	17.730421000	14.616554000
C	17.537360000	19.635830000	15.126812000
H	17.578710000	19.551127000	16.218466000
H	18.558967000	19.614283000	14.737511000
H	17.094138000	20.601218000	14.869638000
C	17.340168000	17.149210000	14.887003000
H	18.366565000	17.095828000	14.509633000
H	17.360694000	16.994105000	15.971143000
H	16.767805000	16.334722000	14.431340000
C	19.129267000	20.529774000	11.246041000
C	20.336177000	21.101983000	11.986712000
H	20.490385000	20.573301000	12.933169000
H	21.246810000	21.007378000	11.385506000
H	20.178945000	22.162406000	12.210004000
C	19.334157000	19.039966000	10.974660000
H	18.463118000	18.626003000	10.461421000
H	20.214446000	18.884009000	10.342265000

H	19.477182000	18.493938000	11.912305000
C	18.907904000	21.285306000	9.937026000
H	18.745376000	22.349683000	10.134150000
H	19.769061000	21.183017000	9.268493000
H	18.029608000	20.891854000	9.415780000

[$^{Si}L_3UNU^{Si}L_3$]$^{1-}$ E = -5679.73720103

U	11.933579000	17.679133000	7.840535000
Si	9.690164000	19.229619000	10.405859000
Si	12.343360000	14.019276000	8.809464000
Si	10.737139000	17.766383000	4.308084000
N	13.658452000	18.693967000	8.207963000
O	10.443862000	18.637141000	9.084503000
O	9.993168000	20.855991000	10.639090000
O	10.123879000	18.482520000	11.829669000
O	8.069825000	18.916101000	10.175763000
O	12.109071000	15.542498000	8.285898000
O	13.725702000	13.763712000	9.711778000
O	11.083294000	13.477521000	9.765346000
O	12.521289000	13.060850000	7.457418000
O	11.280403000	18.111641000	5.801977000
O	11.498301000	16.479301000	3.565948000
O	11.023641000	19.090819000	3.335141000
O	9.107861000	17.381542000	4.301632000
C	10.116314000	21.852498000	9.608820000
C	9.117367000	21.594111000	8.483186000
H	9.336878000	20.649064000	7.980895000
H	9.163440000	22.395546000	7.739949000
H	8.098089000	21.545853000	8.877926000
C	11.548500000	21.838577000	9.085594000
H	11.695762000	22.560673000	8.280652000
H	11.806065000	20.848710000	8.699284000
H	12.246604000	22.080389000	9.891971000
C	9.807317000	23.182678000	10.288085000
H	8.784992000	23.184380000	10.680310000
H	9.908002000	24.011547000	9.580911000
H	10.494962000	23.353052000	11.122613000
C	11.303853000	18.736559000	12.613735000
C	12.499948000	19.081035000	11.730482000
H	12.732499000	18.265758000	11.043146000
H	13.388323000	19.270411000	12.337413000
H	12.296956000	19.979006000	11.141941000
C	10.997918000	19.882202000	13.575040000
H	10.754888000	20.786173000	13.011189000
H	11.855831000	20.087793000	14.223278000
H	10.142526000	19.625606000	14.209117000
C	11.566025000	17.446987000	13.383310000
H	10.696529000	17.184305000	13.994735000
H	12.432297000	17.558653000	14.041973000
H	11.761369000	16.623943000	12.691692000
C	7.003227000	19.045973000	11.124254000
C	5.741031000	19.262806000	10.294191000
H	5.824157000	20.184618000	9.709502000
H	4.856416000	19.338153000	10.935159000
H	5.594741000	18.429432000	9.599750000
C	7.224594000	20.230285000	12.062998000
H	8.132923000	20.091000000	12.654655000
H	6.378212000	20.329359000	12.750931000
H	7.323780000	21.160910000	11.498336000
C	6.906262000	17.746944000	11.921355000
H	6.726458000	16.902635000	11.248338000
H	6.084735000	17.790356000	12.644893000
H	7.841411000	17.570888000	12.459016000

C	14.100680000	14.337283000	10.974387000
C	15.536309000	13.879729000	11.208588000
H	15.585331000	12.786810000	11.254166000
H	15.929203000	14.285148000	12.145989000
H	16.180217000	14.216788000	10.392683000
C	14.021107000	15.857867000	10.900897000
H	14.634804000	16.247006000	10.085893000
H	14.363852000	16.314895000	11.833311000
H	12.988115000	16.170059000	10.727941000
C	13.195900000	13.804847000	12.083007000
H	12.160066000	14.105185000	11.915765000
H	13.523749000	14.184721000	13.056470000
H	13.228537000	12.710972000	12.109799000
C	9.728789000	13.943508000	9.818415000
C	9.157064000	14.121836000	8.414415000
H	9.241266000	13.192070000	7.843503000
H	9.698183000	14.905522000	7.881881000
H	8.100793000	14.405295000	8.462564000
C	8.957019000	12.862052000	10.569784000
H	9.399254000	12.697596000	11.557534000
H	8.986659000	11.916345000	10.019030000
H	7.909841000	13.152158000	10.704133000
C	9.679567000	15.258618000	10.590369000
H	10.273272000	16.028475000	10.094825000
H	10.068475000	15.125752000	11.603861000
H	8.653413000	15.626627000	10.666932000
C	13.105646000	11.757058000	7.353893000
C	14.624248000	11.908489000	7.271705000
H	15.108434000	10.935077000	7.136500000
H	15.001832000	12.368050000	8.187734000
H	14.893550000	12.548118000	6.425881000
C	12.557685000	11.165064000	6.058348000
H	12.820991000	11.801791000	5.207870000
H	11.466772000	11.087052000	6.104789000
H	12.969069000	10.166162000	5.879868000
C	12.713585000	10.881476000	8.542235000
H	13.134765000	9.876520000	8.431917000
H	11.625888000	10.795272000	8.615582000
H	13.089984000	11.312693000	9.473532000
C	11.734912000	15.172576000	4.118542000
C	13.085895000	15.181492000	4.824566000
H	13.097944000	15.933024000	5.616671000
H	13.888370000	15.417313000	4.120625000
H	13.281261000	14.215824000	5.295584000
C	10.633887000	14.762563000	5.089966000
H	10.581895000	15.460815000	5.928138000
H	10.854449000	13.778203000	5.508662000
H	9.660218000	14.748095000	4.594860000
C	11.765853000	14.220822000	2.927119000
H	12.529874000	14.535358000	2.208899000
H	10.796918000	14.213296000	2.417599000
H	11.994684000	13.199812000	3.249551000
C	11.039757000	19.131223000	1.901475000
C	12.437732000	18.732749000	1.432863000
H	12.518347000	18.782761000	0.341457000
H	12.661366000	17.714082000	1.759216000
H	13.180101000	19.409701000	1.865246000
C	9.983483000	18.206808000	1.298649000
H	9.995761000	18.282740000	0.206031000
H	8.984805000	18.475584000	1.653419000
H	10.179751000	17.167350000	1.573953000
C	10.745752000	20.579538000	1.523301000
H	9.754254000	20.877184000	1.879046000

H	10.777132000	20.714107000	0.437004000
H	11.487730000	21.245284000	1.973198000
C	8.081046000	17.956729000	5.119762000
C	6.779281000	17.740987000	4.352463000
H	6.817310000	18.256830000	3.387648000
H	5.923068000	18.124499000	4.916915000
H	6.620711000	16.674165000	4.164046000
C	8.322163000	19.447391000	5.347563000
H	9.276710000	19.604477000	5.854973000
H	7.528634000	19.874149000	5.969431000
H	8.343019000	19.984125000	4.394928000
C	8.034856000	17.209759000	6.451506000
H	7.939716000	16.134359000	6.278218000
H	7.185421000	17.541068000	7.057177000
H	8.941786000	17.392330000	7.033357000
U	15.308662000	19.831173000	8.586281000
Si	17.760769000	18.170733000	6.252538000
Si	14.454092000	23.169049000	6.983383000
Si	16.500590000	19.785663000	12.048538000
O	16.805380000	18.943563000	7.323449000
O	17.673606000	16.506982000	6.372584000
O	17.426723000	18.561192000	4.668106000
O	19.307074000	18.730181000	6.524248000
O	14.848156000	21.730260000	7.627166000
O	12.946833000	23.232039000	6.278316000
O	15.537844000	23.633248000	5.795256000
O	14.419842000	24.264783000	8.240733000
O	16.038151000	19.245379000	10.586545000
O	15.412315000	20.817490000	12.794170000
O	16.630096000	18.451069000	13.036436000
O	17.924382000	20.666239000	11.984407000
C	17.252753000	15.695698000	7.483770000
C	17.775755000	16.263137000	8.799814000
H	17.315497000	17.228800000	9.014198000
H	17.545919000	15.590771000	9.630683000
H	18.860084000	16.396680000	8.758889000
C	15.730575000	15.619167000	7.489871000
H	15.368093000	14.939930000	8.264639000
H	15.290778000	16.604514000	7.673159000
H	15.368552000	15.253305000	6.525349000
C	17.848378000	14.314666000	7.226756000
H	18.942543000	14.359728000	7.220265000
H	17.535672000	13.608057000	8.001992000
H	17.516432000	13.930906000	6.256924000
C	16.285545000	18.149405000	3.898741000
C	15.019469000	18.181577000	4.748424000
H	14.895563000	19.165462000	5.201336000
H	14.136767000	17.969570000	4.140499000
H	15.068215000	17.440812000	5.548466000
C	16.541769000	16.746453000	3.352532000
H	16.670734000	16.041896000	4.177022000
H	15.705624000	16.415943000	2.727102000
H	17.451941000	16.734176000	2.743333000
C	16.176672000	19.150888000	2.753440000
H	17.131042000	19.225802000	2.222133000
H	15.407215000	18.839439000	2.041218000
H	15.912849000	20.142216000	3.132433000
C	20.541600000	18.221685000	6.002946000
C	21.106355000	17.218592000	7.006257000
H	20.441060000	16.355115000	7.089533000
H	22.094461000	16.863757000	6.693637000
H	21.202156000	17.679654000	7.994116000
C	20.358887000	17.552564000	4.641534000

H	19.925809000	18.249409000	3.920551000
H	21.326733000	17.207758000	4.261946000
H	19.695908000	16.686497000	4.724679000
C	21.463285000	19.432038000	5.877427000
H	21.573114000	19.927671000	6.847429000
H	22.457191000	19.136267000	5.525640000
H	21.045745000	20.154730000	5.169171000
C	12.529701000	22.528751000	5.091517000
C	11.049186000	22.221594000	5.280368000
H	10.478292000	23.141765000	5.443778000
H	10.651501000	21.703437000	4.404103000
H	10.914431000	21.568992000	6.145961000
C	13.295827000	21.223576000	4.911296000
H	13.178641000	20.585699000	5.790420000
H	12.899620000	20.672701000	4.054966000
H	14.361022000	21.414548000	4.755105000
C	12.759230000	23.459995000	3.904761000
H	13.822739000	23.701313000	3.824302000
H	12.432901000	22.994077000	2.969346000
H	12.200128000	24.392540000	4.036384000
C	16.955897000	23.402558000	5.796264000
C	17.542242000	23.513745000	7.202965000
H	17.349620000	24.501893000	7.630928000
H	17.102179000	22.760904000	7.860909000
H	18.624507000	23.352221000	7.175132000
C	17.546493000	24.484413000	4.896731000
H	17.103671000	24.431128000	3.896936000
H	17.343571000	25.478624000	5.308008000
H	18.630650000	24.364011000	4.801247000
C	17.224264000	22.018659000	5.213100000
H	16.763836000	21.240008000	5.823270000
H	16.814201000	21.952051000	4.201382000
H	18.298159000	21.814243000	5.159079000
C	13.902502000	25.600158000	8.241280000
C	12.406028000	25.534769000	8.538021000
H	11.974320000	26.539242000	8.609035000
H	11.894328000	24.984734000	7.744505000
H	12.234748000	25.017023000	9.486144000
C	14.639178000	26.326647000	9.363696000
H	14.469331000	25.823136000	10.320980000
H	15.716345000	26.336153000	9.168877000
H	14.293522000	27.361673000	9.453726000
C	14.153249000	26.299732000	6.907092000
H	13.781937000	27.329346000	6.945072000
H	15.221400000	26.323479000	6.675746000
H	13.638719000	25.778434000	6.095290000
C	15.120375000	22.169123000	12.400518000
C	15.108156000	22.296976000	10.880910000
H	16.097593000	22.078558000	10.464620000
H	14.357384000	21.613288000	10.468145000
H	14.833078000	23.299236000	10.548105000
C	16.161857000	23.102070000	13.012528000
H	17.159598000	22.832093000	12.660378000
H	15.955061000	24.142934000	12.741228000
H	16.145701000	23.022224000	14.104559000
C	13.733046000	22.480011000	12.951886000
H	12.986655000	21.820474000	12.503690000
H	13.713011000	22.339293000	14.037057000
H	13.453921000	23.515723000	12.732145000
C	16.684816000	18.412246000	14.468629000
C	15.258730000	18.495859000	15.009409000
H	15.248030000	18.427969000	16.102680000
H	14.801069000	19.441034000	14.707999000

H	14.659648000	17.673430000	14.607281000
C	17.538393000	19.550738000	15.023422000
H	17.601622000	19.480542000	16.114564000
H	18.551716000	19.509606000	14.614500000
H	17.103045000	20.519969000	14.765662000
C	17.307564000	17.063431000	14.817232000
H	18.322294000	16.992521000	14.412845000
H	17.356059000	16.922861000	15.902021000
H	16.712165000	16.250320000	14.390220000
C	19.069792000	20.446667000	11.150876000
C	20.254040000	21.042733000	11.907189000
H	20.395563000	20.525773000	12.861437000
H	21.176034000	20.951418000	11.323751000
H	20.080719000	22.103372000	12.115435000
C	19.294107000	18.958781000	10.899043000
H	18.435326000	18.525746000	10.382576000
H	20.180768000	18.805889000	10.275858000
H	19.435743000	18.424884000	11.843254000
C	18.859228000	21.192662000	9.835129000
H	18.661218000	22.252032000	10.022698000
H	19.735849000	21.111012000	9.185543000
H	18.011461000	20.773148000	9.288298000

$[^{Si}L_3USU^{Si}L_3]^{2-}$ E = -5635.31482310

U	11.526219000	17.695952000	8.224022000
Si	8.904670000	19.381456000	10.198676000
Si	11.747237000	14.251691000	9.661376000
Si	9.760659000	17.667440000	4.890091000
S	13.592056000	19.350367000	7.669257000
O	10.301024000	18.709385000	9.735990000
O	8.923058000	21.053815000	10.103938000
O	8.469989000	19.056167000	11.782750000
O	7.691961000	18.720735000	9.259889000
O	11.298745000	15.578140000	8.859550000
O	13.231574000	14.356613000	10.432744000
O	10.659508000	13.813589000	10.864037000
O	11.906488000	13.027908000	8.535690000
O	10.324817000	17.639909000	6.405849000
O	10.859254000	18.120912000	3.716586000
O	8.533364000	18.798326000	4.831117000
O	9.198359000	16.168838000	4.385539000
C	9.492390000	21.846767000	9.050273000
C	9.213136000	21.233991000	7.679377000
H	9.714606000	20.270871000	7.557886000
H	9.578963000	21.894871000	6.888392000
H	8.140204000	21.080259000	7.534470000
C	10.993307000	21.985892000	9.285000000
H	11.442563000	22.643529000	8.536075000
H	11.493368000	21.016615000	9.215029000
H	11.184334000	22.410499000	10.276412000
C	8.809430000	23.208275000	9.156995000
H	7.730583000	23.110482000	8.996131000
H	9.209241000	23.903779000	8.411691000
H	8.969270000	23.639646000	10.150798000
C	9.112318000	19.562607000	12.959338000
C	10.617749000	19.716458000	12.748904000
H	11.062578000	18.764816000	12.449769000
H	11.095329000	20.054287000	13.674836000
H	10.829908000	20.449714000	11.966104000
C	8.481536000	20.908409000	13.313519000
H	8.640260000	21.615936000	12.496123000
H	8.917855000	21.319472000	14.230741000
H	7.403279000	20.793066000	13.468195000

C	8.839464000	18.536035000	14.055181000
H	7.761792000	18.391179000	14.183675000
H	9.260769000	18.862055000	15.012147000
H	9.283925000	17.571880000	13.791055000
C	6.277216000	18.822683000	9.431217000
C	5.675623000	18.542342000	8.056974000
H	6.039423000	19.271055000	7.326762000
H	4.581651000	18.591123000	8.087532000
H	5.970566000	17.547891000	7.711884000
C	5.868605000	20.214488000	9.910894000
H	6.306002000	20.431500000	10.888998000
H	4.778447000	20.280010000	9.997112000
H	6.208694000	20.979456000	9.207725000
C	5.831835000	17.760564000	10.435456000
H	6.129170000	16.768034000	10.083621000
H	4.743547000	17.770624000	10.564501000
H	6.307489000	17.943361000	11.402095000
C	13.509457000	15.049206000	11.659196000
C	14.992388000	15.396689000	11.614477000
H	15.593598000	14.492117000	11.477230000
H	15.304203000	15.879197000	12.545624000
H	15.195802000	16.080213000	10.785563000
C	12.688022000	16.330265000	11.785032000
H	12.920778000	17.024494000	10.970642000
H	12.916525000	16.832197000	12.730331000
H	11.618293000	16.114786000	11.765951000
C	13.210070000	14.107842000	12.824806000
H	12.152264000	13.833968000	12.818352000
H	13.449947000	14.582330000	13.782770000
H	13.807046000	13.193822000	12.737069000
C	9.248842000	14.072520000	10.884467000
C	8.633331000	13.884397000	9.499652000
H	8.843503000	12.879682000	9.119559000
H	9.044689000	14.616719000	8.802123000
H	7.547199000	14.016654000	9.544877000
C	8.661949000	13.059164000	11.863916000
H	9.126392000	13.166459000	12.849641000
H	8.840522000	12.037972000	11.511253000
H	7.581942000	13.202852000	11.974714000
C	9.014853000	15.496011000	11.384416000
H	9.466495000	16.223803000	10.706405000
H	9.459581000	15.622133000	12.376570000
H	7.946567000	15.718648000	11.462149000
C	12.548714000	11.760318000	8.677140000
C	14.045101000	11.949629000	8.434511000
H	14.578947000	10.993568000	8.478441000
H	14.456785000	12.622231000	9.190799000
H	14.210717000	12.394757000	7.449031000
C	11.938436000	10.869406000	7.597818000
H	12.082702000	11.318296000	6.609910000
H	10.862487000	10.753022000	7.764239000
H	12.398698000	9.875564000	7.598250000
C	12.303012000	11.158623000	10.059719000
H	12.768988000	10.170027000	10.134430000
H	11.231874000	11.054236000	10.252580000
H	12.728099000	11.799746000	10.836261000
C	12.009605000	17.397179000	3.256544000
C	12.791495000	16.796005000	4.423484000
H	12.189105000	16.059819000	4.965419000
H	13.100734000	17.574540000	5.127402000
H	13.690484000	16.292160000	4.053100000
C	11.559356000	16.304775000	2.287850000
H	10.893772000	15.603866000	2.795565000

H	12.423027000	15.759076000	1.892112000
H	11.015337000	16.743915000	1.444402000
C	12.865221000	18.429413000	2.528770000
H	13.189026000	19.206757000	3.225219000
H	12.288504000	18.899885000	1.725295000
H	13.751302000	17.962000000	2.087191000
C	8.037211000	19.530404000	3.710532000
C	8.986378000	20.695491000	3.431401000
H	8.616090000	21.322653000	2.612450000
H	9.975827000	20.313186000	3.169823000
H	9.084074000	21.315132000	4.328146000
C	7.902664000	18.633226000	2.480647000
H	7.480139000	19.193600000	1.639341000
H	7.247842000	17.783181000	2.694519000
H	8.881997000	18.248689000	2.184433000
C	6.666605000	20.053661000	4.135146000
H	5.993614000	19.221454000	4.364711000
H	6.213224000	20.657851000	3.342058000
H	6.762113000	20.675277000	5.030809000
C	8.576889000	15.165291000	5.197968000
C	7.797813000	14.278710000	4.228545000
H	7.035162000	14.862256000	3.702304000
H	7.300755000	13.459558000	4.759044000
H	8.471511000	13.845487000	3.481889000
C	7.625829000	15.798513000	6.211869000
H	8.173679000	16.449786000	6.897471000
H	7.117674000	15.026981000	6.799742000
H	6.868526000	16.397409000	5.697352000
C	9.663232000	14.356134000	5.900163000
H	10.367098000	13.953156000	5.164083000
H	9.230652000	13.513517000	6.449249000
H	10.217410000	14.973169000	6.610740000
U	16.127233000	20.081206000	8.140437000
Si	18.316558000	17.511336000	6.432030000
Si	15.765903000	23.349255000	6.732339000
Si	17.701132000	20.419526000	11.425246000
O	17.560562000	18.609683000	7.345208000
O	17.497232000	16.053237000	6.329562000
O	18.559536000	17.961233000	4.837051000
O	19.845640000	17.310710000	7.079453000
O	16.440361000	21.887225000	6.924268000
O	14.157805000	23.377047000	6.295555000
O	16.566937000	24.231397000	5.543701000
O	15.830732000	24.132596000	8.207480000
O	16.842111000	20.805888000	10.112073000
O	16.799443000	20.170957000	12.815045000
O	18.544104000	19.015913000	11.088179000
O	18.779324000	21.623608000	11.878996000
C	16.867584000	15.333005000	7.399605000
C	17.559072000	15.603868000	8.733900000
H	17.469500000	16.658744000	9.007158000
H	17.103245000	15.000158000	9.523497000
H	18.621604000	15.351868000	8.673887000
C	15.398968000	15.744731000	7.464778000
H	14.864626000	15.203386000	8.252925000
H	15.306011000	16.816634000	7.665918000
H	14.899009000	15.543000000	6.511603000
C	17.003591000	13.856652000	7.037006000
H	18.060499000	13.579445000	6.963960000
H	16.531968000	13.224091000	7.794440000
H	16.529469000	13.652487000	6.071604000
C	17.545733000	18.098487000	3.830324000
C	16.260433000	18.678799000	4.412740000

H	16.448630000	19.651575000	4.874958000
H	15.514013000	18.814406000	3.626413000
H	15.834652000	18.015507000	5.170383000
C	17.279309000	16.726506000	3.212952000
H	16.917143000	16.039378000	3.981111000
H	16.531532000	16.794983000	2.415114000
H	18.200623000	16.315542000	2.785692000
C	18.127561000	19.052788000	2.791239000
H	19.058916000	18.649536000	2.379481000
H	17.424412000	19.210201000	1.966570000
H	18.345736000	20.022236000	3.249407000
C	21.003464000	16.742672000	6.465830000
C	21.916799000	16.324770000	7.616129000
H	21.429665000	15.562235000	8.232307000
H	22.860217000	15.913996000	7.240710000
H	22.142082000	17.185538000	8.253296000
C	20.646044000	15.527267000	5.611638000
H	19.969560000	15.813873000	4.802186000
H	21.549443000	15.090182000	5.172247000
H	20.151899000	14.762447000	6.217312000
C	21.678012000	17.817026000	5.612603000
H	21.918590000	18.690058000	6.228379000
H	22.605895000	17.442577000	5.165692000
H	21.002187000	18.134766000	4.814750000
C	13.572639000	22.934453000	5.062995000
C	12.146941000	22.515582000	5.407438000
H	11.597617000	23.356153000	5.845084000
H	11.612325000	22.181624000	4.512589000
H	12.166007000	21.690785000	6.125530000
C	14.330413000	21.743545000	4.484021000
H	14.305974000	20.906522000	5.187331000
H	13.865722000	21.427264000	3.544659000
H	15.372737000	22.001768000	4.276479000
C	13.582465000	24.110874000	4.088863000
H	14.611499000	24.427639000	3.897662000
H	13.111750000	23.837698000	3.137917000
H	13.033430000	24.959374000	4.511663000
C	17.968669000	24.137589000	5.256861000
C	18.789022000	24.146670000	6.546893000
H	18.578514000	25.048661000	7.128826000
H	18.544847000	23.274374000	7.158980000
H	19.861035000	24.121064000	6.322769000
C	18.296078000	25.366006000	4.412457000
H	17.682817000	25.379266000	3.505361000
H	18.092725000	26.282469000	4.976019000
H	19.350405000	25.368603000	4.116127000
C	18.236044000	22.856722000	4.465752000
H	17.960997000	21.988616000	5.069512000
H	17.642684000	22.847987000	3.545387000
H	19.294319000	22.778655000	4.193146000
C	15.036365000	25.226608000	8.672099000
C	13.670743000	24.695760000	9.105297000
H	13.059292000	25.492034000	9.544989000
H	13.146909000	24.275965000	8.244972000
H	13.796391000	23.902947000	9.848564000
C	15.784995000	25.800956000	9.872102000
H	15.902603000	25.036220000	10.645001000
H	16.780772000	26.145048000	9.574808000
H	15.239826000	26.647549000	10.303276000
C	14.884616000	26.289454000	7.585011000
H	14.318426000	27.148301000	7.962068000
H	15.865611000	26.639790000	7.250851000
H	14.355210000	25.876554000	6.722392000

C	15.397634000	20.437265000	12.976696000
C	14.586776000	19.452788000	12.137505000
H	14.758873000	19.612007000	11.070019000
H	14.874655000	18.426182000	12.379854000
H	13.515260000	19.567987000	12.323810000
C	15.078258000	21.877902000	12.581460000
H	15.324261000	22.039621000	11.529070000
H	14.013312000	22.089676000	12.725597000
H	15.656997000	22.580770000	13.190479000
C	15.115294000	20.216916000	14.460555000
H	15.348632000	19.185631000	14.745878000
H	15.729754000	20.887812000	15.070117000
H	14.061681000	20.407220000	14.689501000
C	19.144794000	18.082467000	11.984603000
C	18.072346000	17.088253000	12.424628000
H	18.490505000	16.306468000	13.068712000
H	17.286044000	17.614931000	12.971588000
H	17.624432000	16.614968000	11.547338000
C	19.754132000	18.783888000	13.197402000
H	20.258630000	18.057990000	13.844719000
H	20.484415000	19.534388000	12.882702000
H	18.976946000	19.284297000	13.782578000
C	20.231079000	17.380105000	11.173698000
H	21.008983000	18.093376000	10.883315000
H	20.696878000	16.572962000	11.749466000
H	19.801926000	16.960321000	10.260343000
C	19.543659000	22.421233000	10.964374000
C	20.675539000	23.020221000	11.795929000
H	21.313383000	22.228150000	12.202248000
H	21.297585000	23.689004000	11.191512000
H	20.266499000	23.592734000	12.635066000
C	20.119108000	21.576634000	9.827047000
H	19.320796000	21.138730000	9.221401000
H	20.741986000	22.193859000	9.170809000
H	20.730866000	20.759691000	10.220221000
C	18.642870000	23.522402000	10.409648000
H	18.232243000	24.115043000	11.233619000
H	19.200788000	24.194045000	9.748561000
H	17.811431000	23.089473000	9.849642000

$[^{Si}L_3USU^{Si}L_3]^{1-}$ E = -5635.25847439

U	11.814864000	17.238274000	8.201527000
Si	9.442673000	19.505201000	9.959801000
Si	11.574950000	13.970502000	9.852863000
Si	10.023001000	16.898813000	5.151096000
S	14.006031000	17.977581000	9.406689000
O	10.577849000	18.573463000	9.234798000
O	9.791595000	21.121037000	9.764027000
O	9.279225000	19.247519000	11.592221000
O	8.001695000	19.070154000	9.257874000
O	11.457499000	15.533963000	9.379674000
O	13.066742000	13.555022000	10.447905000
O	10.458387000	13.625387000	11.041516000
O	11.365166000	13.075297000	8.465519000
O	10.296085000	16.787727000	6.762136000
O	11.436695000	16.719675000	4.281105000
O	9.483170000	18.445099000	4.882640000
O	8.946848000	15.752476000	4.607781000
C	10.260521000	21.749146000	8.557298000
C	9.603554000	21.139631000	7.321709000
H	9.892030000	20.095956000	7.174004000
H	9.904557000	21.700855000	6.432723000
H	8.513851000	21.179409000	7.401483000

C	11.777027000	21.612611000	8.482042000
H	12.175471000	22.123667000	7.602795000
H	12.068019000	20.558619000	8.436527000
H	12.245710000	22.047398000	9.369083000
C	9.864066000	23.216679000	8.684021000
H	8.774433000	23.316538000	8.725351000
H	10.232850000	23.791840000	7.829686000
H	10.282528000	23.646918000	9.598591000
C	10.267410000	19.399426000	12.627911000
C	11.513172000	18.590466000	12.285351000
H	11.246344000	17.551273000	12.083656000
H	12.222887000	18.606823000	13.117698000
H	12.018761000	18.979429000	11.398389000
C	10.608775000	20.876425000	12.804770000
H	11.065427000	21.277055000	11.897740000
H	11.304240000	21.008614000	13.639524000
H	9.703548000	21.456273000	13.012855000
C	9.604682000	18.854080000	13.889147000
H	8.685396000	19.407386000	14.107638000
H	10.275468000	18.945198000	14.749494000
H	9.348699000	17.798443000	13.760019000
C	6.671204000	19.344302000	9.713926000
C	5.788110000	19.256386000	8.472774000
H	6.093098000	20.004917000	7.734565000
H	4.736553000	19.426536000	8.726315000
H	5.880037000	18.268606000	8.011312000
C	6.574303000	20.735530000	10.337071000
H	7.209911000	20.806803000	11.224372000
H	5.542518000	20.946361000	10.637624000
H	6.889786000	21.501743000	9.623502000
C	6.283244000	18.272149000	10.730268000
H	6.379330000	17.278699000	10.281389000
H	5.248168000	18.402409000	11.064778000
H	6.945069000	18.326962000	11.598110000
C	13.664290000	13.932027000	11.702780000
C	15.168688000	13.889306000	11.460629000
H	15.472230000	12.896004000	11.113662000
H	15.719097000	14.116919000	12.378420000
H	15.446625000	14.623905000	10.699438000
C	13.234653000	15.334760000	12.118764000
H	13.489639000	16.068284000	11.348136000
H	13.737517000	15.623327000	13.047798000
H	12.155307000	15.369202000	12.293125000
C	13.246301000	12.902803000	12.749411000
H	12.158642000	12.907137000	12.863004000
H	13.701964000	13.126723000	13.719981000
H	13.560809000	11.898538000	12.446510000
C	9.109943000	14.128657000	11.112034000
C	8.460160000	14.130014000	9.731210000
H	8.506413000	13.133941000	9.283115000
H	8.966866000	14.835381000	9.068296000
H	7.410174000	14.431712000	9.802251000
C	8.375278000	13.176414000	12.049176000
H	8.870621000	13.146417000	13.024913000
H	8.369228000	12.161873000	11.638042000
H	7.339015000	13.497085000	12.196940000
C	9.135852000	15.541120000	11.688184000
H	9.664995000	16.219571000	11.016828000
H	9.639615000	15.543780000	12.659767000
H	8.119444000	15.921591000	11.826137000
C	11.727206000	11.703965000	8.243257000
C	13.217978000	11.638623000	7.915938000
H	13.519835000	10.614005000	7.673370000

H	13.805016000	11.989561000	8.767842000
H	13.443310000	12.276968000	7.055859000
C	10.897442000	11.248696000	7.047065000
H	11.103216000	11.882646000	6.179710000
H	9.829235000	11.313207000	7.275112000
H	11.132956000	10.212699000	6.782510000
C	11.407638000	10.848866000	9.467478000
H	11.658107000	9.800336000	9.275189000
H	10.344044000	10.908290000	9.715390000
H	11.984563000	11.185254000	10.333451000
C	12.192854000	15.512458000	4.072985000
C	12.388818000	14.752285000	5.383603000
H	11.439344000	14.450454000	5.829785000
H	12.942775000	15.356601000	6.112056000
H	12.981350000	13.849676000	5.207042000
C	11.474684000	14.636010000	3.050062000
H	10.487191000	14.351346000	3.418523000
H	12.055416000	13.730768000	2.844704000
H	11.345975000	15.181362000	2.109704000
C	13.539661000	15.973177000	3.529107000
H	14.040234000	16.619083000	4.253921000
H	13.400993000	16.539136000	2.603040000
H	14.188907000	15.118095000	3.318638000
C	9.498569000	19.195301000	3.657719000
C	10.857846000	19.882570000	3.550697000
H	10.901672000	20.540941000	2.677028000
H	11.652722000	19.136373000	3.468847000
H	11.039028000	20.482764000	4.446352000
C	9.244145000	18.293776000	2.451917000
H	9.206949000	18.888900000	1.533399000
H	8.292110000	17.766278000	2.560276000
H	10.041729000	17.553327000	2.347184000
C	8.380712000	20.224410000	3.790307000
H	7.413916000	19.723803000	3.904497000
H	8.336470000	20.867102000	2.905123000
H	8.544602000	20.854926000	4.668066000
C	7.899807000	15.110640000	5.360372000
C	6.904054000	14.605822000	4.321571000
H	6.473767000	15.443473000	3.763695000
H	6.088543000	14.054789000	4.801167000
H	7.399886000	13.938155000	3.609784000
C	7.225048000	16.097377000	6.309868000
H	7.936537000	16.469555000	7.050250000
H	6.399083000	15.610936000	6.838962000
H	6.823308000	16.949592000	5.753125000
C	8.516464000	13.944517000	6.125959000
H	8.986642000	13.244921000	5.428200000
H	7.752260000	13.401150000	6.691186000
H	9.276654000	14.298341000	6.825860000
U	15.554121000	20.000496000	8.392807000
Si	17.954922000	18.230610000	6.104901000
Si	14.812773000	23.174960000	6.819391000
Si	17.486332000	20.418416000	11.264731000
O	16.733651000	18.865823000	6.961103000
O	17.865821000	16.560636000	6.008476000
O	18.049621000	18.747124000	4.517173000
O	19.372114000	18.729469000	6.828483000
O	14.867495000	21.564186000	7.000589000
O	13.419260000	23.728733000	6.073152000
O	16.050378000	23.787626000	5.873930000
O	14.821006000	23.825998000	8.356324000
O	17.043990000	20.874534000	9.767994000
O	16.218504000	20.340835000	12.352456000

O	18.097648000	18.868356000	11.157263000
O	18.623081000	21.434964000	11.956802000
C	17.315414000	15.659365000	6.978260000
C	17.733855000	16.049635000	8.393109000
H	17.357505000	17.040653000	8.658866000
H	17.340010000	15.330826000	9.118308000
H	18.824747000	16.059228000	8.474447000
C	15.795356000	15.661015000	6.836453000
H	15.336151000	14.944718000	7.526047000
H	15.403848000	16.654954000	7.067319000
H	15.516469000	15.386835000	5.814780000
C	17.876832000	14.283648000	6.627635000
H	18.968663000	14.282542000	6.709146000
H	17.478428000	13.518543000	7.302158000
H	17.610657000	14.011735000	5.600879000
C	17.064033000	18.591059000	3.486137000
C	15.688910000	19.030762000	3.986731000
H	15.727484000	20.050866000	4.373638000
H	14.952249000	18.989099000	3.177921000
H	15.347265000	18.386323000	4.800578000
C	17.030670000	17.136854000	3.018845000
H	16.738517000	16.476505000	3.837450000
H	16.325829000	17.015058000	2.189615000
H	18.023140000	16.827203000	2.674703000
C	17.523865000	19.493615000	2.344516000
H	18.523167000	19.199459000	2.007533000
H	16.838439000	19.424963000	1.493384000
H	17.565662000	20.535553000	2.673124000
C	20.708161000	18.639551000	6.322648000
C	21.615150000	18.660117000	7.549368000
H	21.400205000	17.804213000	8.196571000
H	22.670404000	18.622077000	7.258894000
H	21.446540000	19.573030000	8.127461000
C	20.920344000	17.351050000	5.529973000
H	20.263106000	17.321762000	4.656440000
H	21.957171000	17.286089000	5.182751000
H	20.705286000	16.476645000	6.149634000
C	20.974185000	19.857958000	5.439790000
H	20.821951000	20.777898000	6.013290000
H	22.003472000	19.853306000	5.063994000
H	20.286843000	19.858294000	4.590120000
C	13.115235000	23.624138000	4.672798000
C	11.593184000	23.629172000	4.580123000
H	11.186922000	24.530788000	5.049435000
H	11.263004000	23.602799000	3.536656000
H	11.179919000	22.757188000	5.093463000
C	13.677466000	22.332040000	4.081970000
H	13.319413000	21.464912000	4.641611000
H	13.372465000	22.225239000	3.036003000
H	14.769890000	22.337419000	4.120135000
C	13.700704000	24.839708000	3.956992000
H	14.784095000	24.863658000	4.096073000
H	13.479017000	24.806068000	2.884554000
H	13.275726000	25.762779000	4.365149000
C	17.443207000	23.447003000	5.903431000
C	17.966776000	23.405409000	7.335158000
H	17.796776000	24.361507000	7.838063000
H	17.477441000	22.620392000	7.918093000
H	19.041660000	23.197745000	7.340166000
C	18.143795000	24.552381000	5.118008000
H	17.759597000	24.594147000	4.093563000
H	17.970325000	25.525304000	5.589065000
H	19.223414000	24.375717000	5.074221000

C	17.648721000	22.100363000	5.220384000
H	17.147390000	21.300271000	5.767035000
H	17.242842000	22.131361000	4.204899000
H	18.711319000	21.851028000	5.157161000
C	14.520724000	25.167166000	8.759234000
C	13.012043000	25.271109000	8.969040000
H	12.728584000	26.269724000	9.319672000
H	12.493132000	25.064911000	8.029913000
H	12.686332000	24.538765000	9.713353000
C	15.262403000	25.373515000	10.075680000
H	14.939902000	24.628461000	10.807934000
H	16.340744000	25.260671000	9.926904000
H	15.069084000	26.370436000	10.485495000
C	14.988241000	26.186371000	7.722126000
H	14.780536000	27.202966000	8.072758000
H	16.062777000	26.094484000	7.543947000
H	14.469193000	26.038058000	6.771550000
C	15.226102000	21.334276000	12.639454000
C	14.261218000	21.467182000	11.460199000
H	14.766048000	21.911381000	10.595936000
H	13.864131000	20.486792000	11.178199000
H	13.420421000	22.117380000	11.721481000
C	15.877655000	22.680895000	12.941101000
H	16.399841000	23.061953000	12.060843000
H	15.116781000	23.412654000	13.233191000
H	16.604030000	22.587691000	13.753203000
C	14.488392000	20.814370000	13.869311000
H	14.033528000	19.843569000	13.654592000
H	15.184677000	20.690608000	14.705173000
H	13.699481000	21.508369000	14.175711000
C	18.260852000	17.905955000	12.206782000
C	16.948505000	17.138690000	12.352087000
H	17.023151000	16.369364000	13.128335000
H	16.141693000	17.828869000	12.611671000
H	16.690324000	16.658178000	11.405001000
C	18.649030000	18.576467000	13.523504000
H	18.822171000	17.821432000	14.298037000
H	19.564122000	19.162502000	13.400393000
H	17.854424000	19.245168000	13.864900000
C	19.379211000	16.974698000	11.747766000
H	20.316011000	17.529603000	11.632533000
H	19.540280000	16.170045000	12.473114000
H	19.126497000	16.526084000	10.783611000
C	19.596444000	22.216080000	11.250392000
C	20.678742000	22.550122000	12.272665000
H	21.159071000	21.635203000	12.634461000
H	21.448663000	23.192163000	11.831587000
H	20.244061000	23.071625000	13.131483000
C	20.195671000	21.428538000	10.085194000
H	19.423041000	21.173681000	9.355449000
H	20.968004000	22.019708000	9.581337000
H	20.652053000	20.501884000	10.445841000
C	18.916849000	23.489707000	10.750162000
H	18.536281000	24.073161000	11.594546000
H	19.615905000	24.117053000	10.186781000
H	18.078806000	23.225425000	10.102269000

[$^{Si}L_3USU^{Si}L_3$] E = -5635.16781290

U	11.819725000	17.252536000	8.123002000
Si	9.433808000	19.356175000	10.043312000
Si	11.727902000	13.937666000	9.747519000
Si	10.016213000	16.909494000	5.079506000
S	14.011361000	18.180553000	9.369428000

O	10.553694000	18.403507000	9.292978000
O	9.780120000	20.961288000	9.780300000
O	9.350673000	19.143450000	11.683389000
O	7.978705000	18.875027000	9.415810000
O	11.716810000	15.510223000	9.249798000
O	13.187340000	13.477255000	10.383747000
O	10.572164000	13.675587000	10.911164000
O	11.498490000	13.069408000	8.352781000
O	10.316026000	16.817001000	6.697473000
O	11.423707000	16.711122000	4.205831000
O	9.501222000	18.462141000	4.813985000
O	8.929353000	15.767031000	4.571041000
C	10.183468000	21.566214000	8.539250000
C	9.483503000	20.916134000	7.349285000
H	9.772274000	19.870597000	7.215341000
H	9.741179000	21.451379000	6.431584000
H	8.397877000	20.950465000	7.471547000
C	11.697700000	21.447512000	8.408687000
H	12.055846000	21.938019000	7.501029000
H	11.999935000	20.395362000	8.388663000
H	12.192902000	21.915101000	9.264589000
C	9.776482000	23.032098000	8.645699000
H	8.688883000	23.121701000	8.730995000
H	10.101987000	23.588415000	7.761609000
H	10.228774000	23.489616000	9.530382000
C	10.373074000	19.356152000	12.675767000
C	11.655206000	18.642210000	12.264631000
H	11.455316000	17.589560000	12.055694000
H	12.398032000	18.696463000	13.065602000
H	12.093473000	19.079335000	11.364544000
C	10.612278000	20.853204000	12.848859000
H	10.975617000	21.292769000	11.917883000
H	11.348351000	21.033441000	13.638373000
H	9.681101000	21.359402000	13.123495000
C	9.812536000	18.752857000	13.958793000
H	8.869567000	19.239014000	14.228609000
H	10.517537000	18.881540000	14.786165000
H	9.623455000	17.683643000	13.826500000
C	6.658754000	19.206126000	9.876288000
C	5.754859000	19.065220000	8.656114000
H	6.056877000	19.768762000	7.873891000
H	4.710843000	19.264835000	8.918185000
H	5.824155000	18.052706000	8.247915000
C	6.602586000	20.632113000	10.418443000
H	7.251395000	20.741253000	11.292367000
H	5.579986000	20.880769000	10.720327000
H	6.922359000	21.350175000	9.658414000
C	6.269044000	18.204819000	10.960332000
H	6.324325000	17.184646000	10.568357000
H	5.247126000	18.385116000	11.310866000
H	6.953406000	18.290196000	11.808258000
C	13.739180000	13.836023000	11.667225000
C	15.248921000	13.703757000	11.506064000
H	15.512398000	12.688689000	11.191798000
H	15.761904000	13.917303000	12.448585000
H	15.609853000	14.405691000	10.749045000
C	13.365703000	15.267052000	12.042098000
H	13.711546000	15.977445000	11.286320000
H	13.820662000	15.535747000	13.000810000
H	12.281526000	15.367420000	12.143780000
C	13.211122000	12.848684000	12.704109000
H	12.121430000	12.912847000	12.760801000
H	13.629070000	13.063784000	13.693208000

H	13.485415000	11.824420000	12.431327000
C	9.244792000	14.237655000	10.965898000
C	8.620974000	14.285923000	9.575203000
H	8.615204000	13.291743000	9.121077000
H	9.176947000	14.963268000	8.922428000
H	7.590163000	14.649439000	9.630788000
C	8.451203000	13.306779000	11.875604000
H	8.923297000	13.242457000	12.861009000
H	8.408015000	12.299056000	11.450415000
H	7.427685000	13.672295000	12.006138000
C	9.331246000	15.636610000	11.565069000
H	9.893476000	16.304903000	10.911031000
H	9.827735000	15.599298000	12.539487000
H	8.333789000	16.061824000	11.703890000
C	11.772639000	11.676647000	8.124778000
C	13.251325000	11.533075000	7.773089000
H	13.495192000	10.494468000	7.525646000
H	13.870527000	11.849160000	8.616186000
H	13.495614000	12.160379000	6.910025000
C	10.895995000	11.272030000	6.944308000
H	11.120871000	11.892750000	6.072227000
H	9.837821000	11.396712000	7.192432000
H	11.067365000	10.224457000	6.676761000
C	11.425906000	10.842626000	9.355381000
H	11.604315000	9.780915000	9.156532000
H	10.374240000	10.970584000	9.626518000
H	12.043372000	11.137314000	10.208757000
C	12.144330000	15.482425000	3.982538000
C	12.318682000	14.717921000	5.291089000
H	11.362776000	14.431614000	5.733341000
H	12.873099000	15.323639000	6.017226000
H	12.900744000	13.807216000	5.123854000
C	11.402142000	14.633839000	2.954713000
H	10.408745000	14.370042000	3.322302000
H	11.960252000	13.716023000	2.743219000
H	11.287623000	15.188368000	2.018022000
C	13.503581000	15.907792000	3.442680000
H	14.020076000	16.536349000	4.171612000
H	13.384655000	16.480230000	2.518005000
H	14.129232000	15.035287000	3.232902000
C	9.436860000	19.175335000	3.565649000
C	10.771747000	19.891563000	3.379449000
H	10.765508000	20.510877000	2.476740000
H	11.582349000	19.162041000	3.298066000
H	10.968617000	20.537144000	4.239451000
C	9.155690000	18.234476000	2.397075000
H	9.069004000	18.805548000	1.466937000
H	8.221568000	17.688568000	2.554924000
H	9.965937000	17.509989000	2.277157000
C	8.299818000	20.179226000	3.719891000
H	7.351048000	19.658781000	3.884017000
H	8.204923000	20.798014000	2.821930000
H	8.481994000	20.835206000	4.575423000
C	7.859816000	15.147828000	5.313737000
C	6.831029000	14.727021000	4.270082000
H	6.427141000	15.603684000	3.754035000
H	6.000875000	14.189677000	4.739512000
H	7.289838000	14.070203000	3.524327000
C	7.241398000	16.127213000	6.307525000
H	7.970646000	16.428341000	7.062342000
H	6.391181000	15.661032000	6.815428000
H	6.883517000	17.022551000	5.790030000
C	8.438282000	13.929906000	6.026012000

H	8.866324000	13.235440000	5.296897000
H	7.661752000	13.401118000	6.587952000
H	9.223910000	14.229738000	6.722885000
U	15.548062000	20.081413000	8.451381000
Si	17.779976000	18.126499000	6.241468000
Si	14.738813000	23.138455000	6.844989000
Si	17.453008000	20.414920000	11.341740000
O	16.580245000	18.899921000	7.082707000
O	17.493400000	16.495342000	6.153362000
O	17.931589000	18.656853000	4.677277000
O	19.165974000	18.533305000	7.049545000
O	14.768777000	21.503850000	7.089604000
O	13.334902000	23.646540000	6.122275000
O	15.974673000	23.661300000	5.870144000
O	14.800337000	23.751821000	8.387117000
O	16.999756000	20.836484000	9.806202000
O	16.165536000	20.415313000	12.390035000
O	17.975972000	18.845844000	11.216601000
O	18.610384000	21.428609000	11.961842000
C	17.044463000	15.588602000	7.176588000
C	17.504003000	16.038825000	8.557883000
H	17.037300000	16.983605000	8.847133000
H	17.228847000	15.288790000	9.304940000
H	18.589394000	16.165908000	8.574689000
C	15.523819000	15.520916000	7.096914000
H	15.122835000	14.796843000	7.813051000
H	15.098864000	16.500857000	7.334992000
H	15.217909000	15.219166000	6.091394000
C	17.656774000	14.235847000	6.829269000
H	18.749451000	14.285817000	6.869673000
H	17.318165000	13.469511000	7.533674000
H	17.364224000	13.931030000	5.819505000
C	16.996220000	18.517756000	3.589790000
C	15.612503000	18.985872000	4.033842000
H	15.656625000	20.009451000	4.411827000
H	14.905389000	18.951505000	3.199485000
H	15.226805000	18.348735000	4.834872000
C	16.964506000	17.066128000	3.119695000
H	16.626904000	16.405662000	3.919832000
H	16.295545000	16.960157000	2.259723000
H	17.966031000	16.745909000	2.814982000
C	17.532160000	19.410704000	2.476054000
H	18.542204000	19.097407000	2.193849000
H	16.891708000	19.347733000	1.590723000
H	17.574070000	20.453233000	2.800331000
C	20.525568000	18.367837000	6.607462000
C	21.361191000	18.273035000	7.878710000
H	21.064582000	17.400780000	8.469437000
H	22.425506000	18.184444000	7.638397000
H	21.216362000	19.165518000	8.492932000
C	20.683840000	17.101227000	5.770458000
H	20.079536000	17.157089000	4.860235000
H	21.730437000	16.971731000	5.476653000
H	20.374420000	16.219590000	6.338366000
C	20.907210000	19.603519000	5.797342000
H	20.775266000	20.504971000	6.403996000
H	21.953084000	19.553437000	5.476507000
H	20.270255000	19.681550000	4.912334000
C	13.001736000	23.533881000	4.721874000
C	11.479389000	23.555145000	4.666589000
H	11.094834000	24.476487000	5.114597000
H	11.126160000	23.498721000	3.632494000
H	11.067974000	22.706192000	5.218455000

C	13.543195000	22.227909000	4.145467000
H	13.181471000	21.372925000	4.722084000
H	13.220692000	22.106051000	3.107073000
H	14.636388000	22.222903000	4.164779000
C	13.587561000	24.735243000	3.986592000
H	14.674747000	24.747178000	4.094078000
H	13.337651000	24.695148000	2.921113000
H	13.184832000	25.666541000	4.397464000
C	17.383540000	23.374975000	5.927367000
C	17.892827000	23.389956000	7.362923000
H	17.690640000	24.351312000	7.841249000
H	17.427020000	22.605941000	7.966049000
H	18.972726000	23.214184000	7.378550000
C	18.050588000	24.483056000	5.119514000
H	17.674377000	24.484945000	4.091751000
H	17.839917000	25.460184000	5.564958000
H	19.135363000	24.341148000	5.089977000
C	17.636005000	22.019399000	5.282316000
H	17.171845000	21.210584000	5.849497000
H	17.223192000	22.007614000	4.270507000
H	18.707299000	21.810616000	5.220162000
C	14.509261000	25.099467000	8.809490000
C	13.008740000	25.191427000	9.067939000
H	12.735722000	26.185762000	9.436746000
H	12.455752000	24.994675000	8.146183000
H	12.709957000	24.452612000	9.817324000
C	15.297445000	25.303596000	10.096852000
H	14.995642000	24.567579000	10.845784000
H	16.369536000	25.186797000	9.911693000
H	15.122850000	26.303980000	10.505276000
C	14.940827000	26.113041000	7.753657000
H	14.743165000	27.129537000	8.108444000
H	16.009682000	26.023937000	7.542193000
H	14.389387000	25.966122000	6.820725000
C	15.219664000	21.458158000	12.679233000
C	14.250931000	21.597734000	11.504384000
H	14.755002000	22.033937000	10.634469000
H	13.840213000	20.618594000	11.236559000
H	13.418406000	22.259356000	11.760667000
C	15.930385000	22.779043000	12.949940000
H	16.458530000	23.128283000	12.060297000
H	15.202284000	23.543941000	13.238859000
H	16.658876000	22.667974000	13.757474000
C	14.480281000	20.988763000	13.926650000
H	13.999067000	20.024942000	13.742598000
H	15.180819000	20.869970000	14.759095000
H	13.713575000	21.712153000	14.219781000
C	18.114660000	17.875240000	12.272453000
C	16.783642000	17.140519000	12.402578000
H	16.836540000	16.370735000	13.179372000
H	15.988761000	17.846060000	12.657436000
H	16.523342000	16.663200000	11.454710000
C	18.504103000	18.542414000	13.589193000
H	18.658771000	17.784753000	14.364273000
H	19.431796000	19.110084000	13.473476000
H	17.718659000	19.222889000	13.929248000
C	19.220281000	16.928115000	11.820019000
H	20.167233000	17.467671000	11.717697000
H	19.359025000	16.119640000	12.545100000
H	18.970944000	16.486339000	10.852379000
C	19.608815000	22.177316000	11.244417000
C	20.701433000	22.483501000	12.262398000
H	21.158889000	21.557472000	12.624632000

H	21.484996000	23.103301000	11.814793000
H	20.283407000	23.019360000	13.120185000
C	20.174203000	21.358817000	10.086490000
H	19.395174000	21.129401000	9.355246000
H	20.969643000	21.913645000	9.578345000
H	20.593369000	20.418299000	10.455361000
C	18.961813000	23.464879000	10.741209000
H	18.604604000	24.065539000	11.583070000
H	19.676362000	24.065611000	10.169278000
H	18.112209000	23.226473000	10.097986000

$[^{Ar}L_3UOU^{Ar}L_3]^{2-}$ E = -4757.59421105

U	6.889053000	9.491593000	8.822621000
O	7.411566000	7.657233000	7.588705000
O	7.482750000	11.591713000	9.476111000
O	7.500085000	8.545337000	10.742803000
O	4.831868000	9.740485000	8.353382000
C	7.617749000	12.877950000	9.745035000
C	7.603490000	6.648155000	6.758252000
C	7.084289000	5.346746000	7.060556000
C	5.082598000	11.961793000	11.406231000
H	4.614179000	11.289707000	12.131749000
H	5.261761000	11.401920000	10.486807000
H	4.366835000	12.754911000	11.176765000
C	7.059356000	13.436121000	10.940840000
C	9.257017000	7.752311000	12.139510000
C	6.358952000	5.053659000	8.380729000
C	8.319059000	6.848492000	5.534206000
C	8.312936000	13.736206000	8.834009000
C	8.436976000	5.783917000	4.637654000
H	8.963266000	5.930166000	3.700786000
C	10.028477000	12.099335000	7.982543000
H	10.784537000	12.519141000	8.655678000
H	10.544645000	11.715724000	7.095173000
H	9.548200000	11.265991000	8.495955000
C	7.235574000	4.330705000	6.113472000
H	6.827735000	3.347413000	6.319376000
C	9.005776000	13.176558000	7.585142000
C	7.860323000	7.937942000	11.860824000
C	7.317962000	11.449898000	12.468109000
H	8.250325000	11.879460000	12.852723000
H	7.560840000	10.756559000	11.666616000
H	6.854711000	10.878278000	13.280001000
C	7.962085000	12.595946000	6.621635000
H	7.377819000	11.805054000	7.094719000
H	8.448961000	12.184853000	5.730377000
H	7.262108000	13.372151000	6.298797000
C	8.342200000	15.109118000	9.088342000
H	8.845097000	15.770902000	8.391587000
C	7.276903000	5.377761000	9.569187000
H	8.217250000	4.819593000	9.494850000
H	6.794956000	5.091990000	10.510806000
H	7.507140000	6.439059000	9.618906000
C	6.368196000	12.559179000	11.990844000
C	10.227691000	7.520562000	9.815475000
H	11.005669000	7.880311000	9.131769000
H	9.259308000	7.677380000	9.339660000
H	10.366954000	6.441218000	9.940586000
C	11.758904000	7.951636000	11.693038000
H	11.942706000	6.879385000	11.822200000
H	11.960437000	8.452245000	12.646533000
H	12.490267000	8.322731000	10.966509000
C	5.957917000	3.576895000	8.510147000

H	5.247898000	3.271954000	7.733587000
H	5.462192000	3.429164000	9.476122000
H	6.822104000	2.903229000	8.477941000
C	5.058146000	5.862784000	8.463516000
H	5.230874000	6.933210000	8.345232000
H	4.568921000	5.697420000	9.429027000
H	4.363758000	5.547408000	7.681167000
C	10.344200000	8.241235000	11.168310000
C	6.890558000	7.463110000	12.793270000
C	5.357901000	7.582740000	12.662417000
C	9.628141000	7.109983000	13.320701000
H	10.678789000	6.962786000	13.543444000
C	4.840800000	8.233533000	11.380334000
H	5.171101000	9.266118000	11.283161000
H	3.748108000	8.239738000	11.410581000
H	5.138348000	7.681017000	10.489822000
C	7.126582000	14.818411000	11.129953000
H	6.681731000	15.255750000	12.016665000
C	7.893815000	4.532903000	4.905135000
H	7.991709000	3.722479000	4.187382000
C	9.776103000	14.254518000	6.810936000
H	9.116924000	15.037417000	6.420876000
H	10.275191000	13.791316000	5.952361000
H	10.548587000	14.728857000	7.426198000
C	4.727607000	6.178694000	12.725375000
H	5.057737000	5.567411000	11.879670000
H	3.635018000	6.256648000	12.677382000
H	4.981398000	5.643207000	13.645295000
C	5.948477000	13.358510000	13.232797000
H	5.205902000	14.128239000	12.996890000
H	6.801221000	13.835403000	13.729822000
H	5.486616000	12.674633000	13.953642000
C	10.256493000	9.763050000	10.970201000
H	11.054509000	10.099389000	10.297802000
H	10.381247000	10.284889000	11.925415000
H	9.301647000	10.071061000	10.545297000
C	9.712538000	8.184210000	3.865077000
H	10.553062000	7.483085000	3.912026000
H	10.123081000	9.181711000	3.672208000
H	9.088993000	7.917775000	3.005132000
C	8.687534000	6.646957000	14.236396000
H	9.003349000	6.151980000	15.151086000
C	8.912693000	8.216623000	5.174913000
C	7.742147000	15.663628000	10.212828000
H	7.771346000	16.736736000	10.383078000
C	4.820296000	8.421439000	13.837325000
H	5.071957000	7.985840000	14.809395000
H	3.728190000	8.499122000	13.778969000
H	5.231640000	9.435394000	13.807527000
C	7.342448000	6.832012000	13.959806000
H	6.610141000	6.473530000	14.678382000
C	9.880073000	8.701337000	6.266607000
H	10.698717000	7.985567000	6.400550000
H	9.380436000	8.817524000	7.228972000
H	10.317191000	9.664686000	5.981755000
C	7.775888000	9.228918000	4.975520000
H	8.178960000	10.227512000	4.776516000
H	7.125423000	9.282475000	5.850461000
H	7.153753000	8.940353000	4.123244000
U	2.774692000	9.989177000	7.884156000
O	2.251728000	11.823146000	9.118472000
O	2.180439000	7.889176000	7.230776000
O	2.163445000	10.935578000	5.964091000

C	2.045605000	6.602879000	6.961956000
C	2.059861000	12.832347000	9.948805000
C	2.579103000	14.133722000	9.646410000
C	4.580912000	7.518979000	5.300886000
H	5.049289000	8.191076000	4.575355000
H	4.401701000	8.078871000	6.220289000
H	5.296727000	6.725911000	5.530391000
C	2.604165000	6.044629000	5.766270000
C	0.406477000	11.728915000	4.567600000
C	3.304419000	14.426773000	8.326218000
C	1.344360000	12.632151000	11.172920000
C	1.350432000	5.744647000	7.873009000
C	1.226597000	13.696778000	12.069430000
H	0.700375000	13.550601000	13.006344000
C	-0.365140000	7.381550000	8.724338000
H	-1.121272000	6.961730000	8.051284000
H	-0.881238000	7.765262000	9.611703000
H	0.115167000	8.214832000	8.210845000
C	2.427974000	15.149820000	10.593464000
H	2.835892000	16.133058000	10.387471000
C	0.657509000	6.304323000	9.121802000
C	1.803179000	11.543266000	4.846240000
C	2.345614000	8.030736000	4.238851000
H	1.413161000	7.601151000	3.854482000
H	2.102896000	8.724281000	5.040215000
H	2.808779000	8.602132000	3.426755000
C	1.701162000	6.884923000	10.085357000
H	2.285472000	7.675806000	9.612305000
H	1.214248000	7.296041000	10.976579000
H	2.401120000	6.108714000	10.408227000
C	1.321228000	4.371719000	7.618762000
H	0.818307000	3.709960000	8.315521000
C	2.386527000	14.102473000	7.137773000
H	1.446174000	14.660646000	7.212010000
H	2.868490000	14.388112000	6.196124000
H	2.156303000	13.041173000	7.088196000
C	3.295368000	6.921505000	4.716244000
C	-0.564439000	11.959763000	6.891675000
H	-1.342482000	11.599687000	7.575133000
H	0.403894000	11.802841000	7.367550000
H	-0.703795000	13.039137000	6.766945000
C	-2.095410000	11.529172000	5.013800000
H	-2.279262000	12.601437000	4.884827000
H	-2.296858000	11.028735000	4.060196000
H	-2.826797000	11.157900000	5.740212000
C	3.705255000	15.903584000	8.196651000
H	4.415251000	16.208720000	8.973156000
H	4.200927000	16.051291000	7.230647000
H	2.840959000	16.577112000	8.228818000
C	4.605334000	13.617811000	8.243570000
H	4.432736000	12.547363000	8.361881000
H	5.094620000	13.783198000	7.278091000
H	5.299607000	13.933336000	9.025964000
C	-0.680726000	11.239555000	5.538573000
C	2.772919000	12.018295000	3.913869000
C	4.305571000	11.898412000	4.044541000
C	0.035328000	12.371595000	3.386606000
H	-1.015328000	12.518829000	3.163928000
C	4.822676000	11.247324000	5.326466000
H	4.492203000	10.214775000	5.423515000
H	5.915366000	11.240913000	5.296118000
H	4.525349000	11.799775000	6.217093000
C	2.536988000	4.662323000	5.577228000

H	2.981902000	4.224955000	4.690564000
C	1.769821000	14.947742000	11.801862000
H	1.672070000	15.758195000	12.519601000
C	-0.112888000	5.226401000	9.895994000
H	0.546256000	4.443560000	10.286230000
H	-0.612111000	5.689679000	10.754452000
H	-0.885265000	4.751990000	9.280657000
C	4.936143000	13.302340000	3.981743000
H	4.606230000	13.913559000	4.827578000
H	6.028721000	13.224129000	4.029633000
H	4.682408000	13.838037000	3.061933000
C	3.715206000	6.122106000	3.474374000
H	4.457743000	5.352374000	3.710387000
H	2.862502000	5.645211000	2.977283000
H	4.177157000	6.805942000	2.753549000
C	-0.592858000	9.717681000	5.736175000
H	-1.390992000	9.380995000	6.408258000
H	-0.717331000	9.196169000	4.780744000
H	0.361936000	9.409658000	6.161181000
C	-0.049214000	11.296733000	12.842229000
H	-0.889630000	11.997987000	12.795213000
H	-0.459912000	10.299305000	13.035147000
H	0.574342000	11.563117000	13.702178000
C	0.975914000	12.834895000	2.471028000
H	0.660090000	13.330146000	1.556491000
C	0.750624000	11.264111000	11.532386000
C	1.921360000	3.817149000	6.494346000
H	1.892203000	2.744029000	6.324175000
C	4.842880000	11.059792000	2.869436000
H	4.591160000	11.495593000	1.897470000
H	5.934983000	10.981901000	2.927633000
H	4.431355000	10.045909000	2.899135000
C	2.321004000	12.649716000	2.747520000
H	3.053293000	13.008350000	2.029001000
C	-0.216821000	10.779414000	10.440742000
H	-1.035216000	11.495420000	10.306549000
H	0.282883000	10.662825000	9.478463000
H	-0.654292000	9.816290000	10.725801000
C	1.887354000	10.251759000	11.731865000
H	1.484215000	9.253213000	11.930998000
H	2.537793000	10.198043000	10.856919000
H	2.509527000	10.540389000	12.584095000

$[{}^{Ar}L_3UOU{}^{Ar}L_3]^{1-}$ E = -4757.53528917

U	6.955528000	9.491259000	8.826826000
O	7.375709000	7.677985000	7.663096000
O	7.383823000	11.525273000	9.509688000
O	7.553468000	8.571354000	10.673479000
O	5.042411000	9.747720000	8.290058000
C	7.606507000	12.815441000	9.795663000
C	7.533125000	6.661245000	6.804389000
C	6.981349000	5.381146000	7.105389000
C	5.070402000	11.973089000	11.477754000
H	4.586595000	11.308327000	12.198563000
H	5.220706000	11.424842000	10.547423000
H	4.379023000	12.791633000	11.263919000
C	7.103448000	13.372371000	11.008420000
C	9.286601000	7.879432000	12.147967000
C	6.278539000	5.089572000	8.435006000
C	8.234002000	6.865300000	5.580359000
C	8.326994000	13.632780000	8.877848000
C	8.293477000	5.814123000	4.661866000
H	8.806627000	5.958417000	3.718444000

C	9.840961000	11.887286000	7.835567000
H	10.613663000	12.154159000	8.563247000
H	10.336583000	11.585965000	6.907639000
H	9.312591000	11.018134000	8.229434000
C	7.072726000	4.377273000	6.137622000
H	6.634685000	3.406580000	6.336744000
C	8.907440000	13.077357000	7.571307000
C	7.898119000	8.023480000	11.847206000
C	7.304247000	11.390151000	12.532382000
H	8.243207000	11.798801000	12.921957000
H	7.541688000	10.697203000	11.729647000
H	6.829581000	10.823944000	13.339913000
C	7.760656000	12.670158000	6.638159000
H	7.081709000	11.959651000	7.113087000
H	8.150388000	12.223406000	5.718288000
H	7.165067000	13.543972000	6.360866000
C	8.467411000	14.993016000	9.167793000
H	9.000782000	15.634450000	8.476482000
C	7.231105000	5.379964000	9.604943000
H	8.153492000	4.797832000	9.500381000
H	6.766316000	5.095119000	10.554307000
H	7.496221000	6.432149000	9.662776000
C	6.380423000	12.521928000	12.057967000
C	10.281119000	7.532223000	9.851535000
H	11.055494000	7.867216000	9.152547000
H	9.311814000	7.633917000	9.363629000
H	10.438336000	6.464364000	10.036491000
C	11.789781000	8.070301000	11.717886000
H	11.973469000	7.007157000	11.905033000
H	11.978100000	8.622324000	12.644720000
H	12.526861000	8.405398000	10.980192000
C	5.850701000	3.620016000	8.560481000
H	5.116988000	3.337238000	7.798329000
H	5.374061000	3.474978000	9.536145000
H	6.700481000	2.930406000	8.503641000
C	4.992291000	5.919456000	8.526363000
H	5.170880000	6.986377000	8.388945000
H	4.507678000	5.769813000	9.495891000
H	4.292550000	5.603926000	7.750410000
C	10.379935000	8.325784000	11.164952000
C	6.908850000	7.596687000	12.771103000
C	5.379349000	7.680126000	12.595836000
C	9.643201000	7.313793000	13.372235000
H	10.690244000	7.196730000	13.624772000
C	4.894580000	8.303095000	11.287611000
H	5.197668000	9.345995000	11.198538000
H	3.802925000	8.288765000	11.274090000
H	5.229466000	7.732381000	10.422627000
C	7.274517000	14.741550000	11.227992000
H	6.878442000	15.187951000	12.132338000
C	7.705956000	4.582524000	4.918209000
H	7.755498000	3.784687000	4.182197000
C	9.748226000	14.118412000	6.816765000
H	9.158326000	14.988902000	6.513344000
H	10.144079000	13.661837000	5.903211000
H	10.600482000	14.467085000	7.409871000
C	4.780825000	6.263039000	12.663232000
H	5.143197000	5.645245000	11.836782000
H	3.688510000	6.316497000	12.591278000
H	5.027598000	5.751813000	13.598302000
C	5.992672000	13.334919000	13.300766000
H	5.290346000	14.140910000	13.063547000
H	6.864100000	13.766241000	13.805944000

H	5.494339000	12.669778000	14.014209000
C	10.288832000	9.836879000	10.897699000
H	11.068773000	10.138867000	10.189856000
H	10.439570000	10.401496000	11.823539000
H	9.324403000	10.141875000	10.491570000
C	9.675215000	8.159480000	3.919740000
H	10.471126000	7.407560000	3.933038000
H	10.143185000	9.135038000	3.748538000
H	9.021391000	7.956739000	3.065445000
C	8.687199000	6.890713000	14.289988000
H	8.990049000	6.453375000	15.237417000
C	8.901641000	8.203131000	5.245132000
C	7.937551000	15.557223000	10.320033000
H	8.052278000	16.619799000	10.515990000
C	4.781353000	8.515711000	13.742698000
H	5.001719000	8.088879000	14.725383000
H	3.692044000	8.564926000	13.639083000
H	5.168489000	9.538399000	13.726173000
C	7.345688000	7.035764000	13.979429000
H	6.604948000	6.703609000	14.700780000
C	9.920922000	8.570930000	6.333309000
H	10.675318000	7.783363000	6.431496000
H	9.450908000	8.690322000	7.309572000
H	10.435882000	9.502310000	6.077663000
C	7.836302000	9.296171000	5.094011000
H	8.306944000	10.269782000	4.927161000
H	7.186907000	9.365594000	5.969182000
H	7.187918000	9.082033000	4.240043000
U	2.862468000	10.007246000	7.813821000
O	2.317921000	11.725694000	9.152480000
O	2.274439000	7.928011000	7.187551000
O	2.150492000	10.944894000	5.961085000
C	2.117408000	6.637397000	6.912972000
C	2.090067000	12.750979000	9.968380000
C	2.597426000	14.053671000	9.662284000
C	4.646198000	7.542090000	5.238662000
H	5.110562000	8.221119000	4.516975000
H	4.476103000	8.088078000	6.167856000
H	5.359432000	6.743956000	5.454680000
C	2.670116000	6.078878000	5.718091000
C	0.379100000	11.714392000	4.570347000
C	3.328835000	14.356439000	8.348738000
C	1.351829000	12.552778000	11.176285000
C	1.414904000	5.787890000	7.822661000
C	1.207863000	13.622408000	12.063381000
H	0.664529000	13.477541000	12.990165000
C	-0.320292000	7.411803000	8.633436000
H	-1.048015000	6.981556000	7.936748000
H	-0.869029000	7.787098000	9.503878000
H	0.162048000	8.251772000	8.134733000
C	2.419946000	15.075423000	10.599306000
H	2.818345000	16.061650000	10.391311000
C	0.704274000	6.349143000	9.059751000
C	1.776906000	11.544065000	4.834799000
C	2.398317000	8.057129000	4.187482000
H	1.465289000	7.622223000	3.811944000
H	2.155258000	8.753033000	4.986630000
H	2.852586000	8.625973000	3.369084000
C	1.726788000	6.943294000	10.036719000
H	2.324805000	7.727017000	9.567503000
H	1.219824000	7.367672000	10.909372000
H	2.415787000	6.169663000	10.387916000
C	1.387159000	4.413644000	7.573845000

H	0.880968000	3.755314000	8.270732000
C	2.409140000	14.045117000	7.158833000
H	1.476869000	14.615717000	7.233780000
H	2.895086000	14.324403000	6.217730000
H	2.161837000	12.987911000	7.106037000
C	3.354282000	6.951741000	4.661224000
C	-0.577005000	11.916727000	6.906580000
H	-1.348458000	11.543082000	7.589692000
H	0.394459000	11.765390000	7.377078000
H	-0.725338000	12.996014000	6.793872000
C	-2.116042000	11.488454000	5.035305000
H	-2.310572000	12.559722000	4.915043000
H	-2.319257000	10.992351000	4.080106000
H	-2.837940000	11.104674000	5.764445000
C	3.732952000	15.833424000	8.229120000
H	4.435174000	16.135080000	9.014022000
H	4.237878000	15.983195000	7.268318000
H	2.869648000	16.507787000	8.254188000
C	4.630909000	13.552133000	8.260402000
H	4.469849000	12.481065000	8.385852000
H	5.108588000	13.718048000	7.290434000
H	5.330846000	13.871776000	9.036041000
C	-0.694657000	11.208984000	5.546915000
C	2.737829000	12.015671000	3.896158000
C	4.271813000	11.909616000	4.015800000
C	-0.006244000	12.348953000	3.389486000
H	-1.059426000	12.487039000	3.174547000
C	4.801602000	11.251366000	5.288528000
H	4.474578000	10.215682000	5.375986000
H	5.893360000	11.244753000	5.246638000
H	4.514282000	11.807426000	6.181672000
C	2.605860000	4.695560000	5.533803000
H	3.049594000	4.256164000	4.647918000
C	1.745700000	14.874981000	11.797731000
H	1.628005000	15.689357000	12.507688000
C	-0.070182000	5.271469000	9.830387000
H	0.587324000	4.493247000	10.232221000
H	-0.580566000	5.737925000	10.680263000
H	-0.833850000	4.791731000	9.208820000
C	4.887148000	13.320509000	3.961970000
H	4.556662000	13.921634000	4.814696000
H	5.980644000	13.254399000	3.998572000
H	4.618723000	13.859436000	3.048762000
C	3.764103000	6.149609000	3.417968000
H	4.508066000	5.380088000	3.649398000
H	2.906893000	5.672341000	2.929810000
H	4.219710000	6.831894000	2.691810000
C	-0.586425000	9.686318000	5.724457000
H	-1.368279000	9.332420000	6.406149000
H	-0.722366000	9.175932000	4.764834000
H	0.380071000	9.382063000	6.125233000
C	-0.072990000	11.225420000	12.827483000
H	-0.916884000	11.919611000	12.750826000
H	-0.481698000	10.226849000	13.017903000
H	0.525014000	11.503927000	13.701455000
C	0.924743000	12.813733000	2.465409000
H	0.598417000	13.302751000	1.551443000
C	0.760275000	11.185781000	11.539199000
C	1.992413000	3.854712000	6.454990000
H	1.965231000	2.780870000	6.290369000
C	4.814935000	11.087250000	2.832092000
H	4.557531000	11.529919000	1.865276000
H	5.908167000	11.021933000	2.887749000

H	4.414832000	10.068821000	2.851519000
C	2.272907000	12.638396000	2.729745000
H	2.996585000	12.998207000	2.003709000
C	-0.173164000	10.681447000	10.429029000
H	-1.003033000	11.380279000	10.278321000
H	0.350983000	10.580789000	9.478786000
H	-0.595916000	9.709440000	10.703581000
C	1.905924000	10.193670000	11.776022000
H	1.514850000	9.195069000	11.996769000
H	2.559227000	10.132412000	10.904177000
H	2.515581000	10.512561000	12.626220000

[$^{Ar}L_3UOU^{Ar}L_3$] E = -4757.44740174

U	6.962168000	9.439317000	8.906895000
O	7.441491000	7.704773000	7.715843000
O	7.378269000	11.471321000	9.496257000
O	7.674049000	8.573261000	10.703990000
O	4.933652000	9.702535000	8.387246000
C	7.692143000	12.751552000	9.791741000
C	7.587531000	6.677894000	6.850826000
C	7.015759000	5.409945000	7.152911000
C	5.155725000	12.019718000	11.513424000
H	4.668064000	11.361464000	12.237025000
H	5.265527000	11.480820000	10.572304000
H	4.487265000	12.863292000	11.326700000
C	7.243638000	13.326942000	11.013965000
C	9.406942000	7.901953000	12.190227000
C	6.341444000	5.110778000	8.494702000
C	8.286819000	6.879382000	5.628884000
C	8.449263000	13.522818000	8.867754000
C	8.299065000	5.839088000	4.695738000
H	8.806814000	5.978067000	3.749333000
C	9.836553000	11.707429000	7.784370000
H	10.679801000	11.950097000	8.438204000
H	10.237001000	11.340250000	6.835852000
H	9.292023000	10.890022000	8.262056000
C	7.061511000	4.416799000	6.170655000
H	6.603581000	3.455044000	6.366270000
C	8.961453000	12.944388000	7.545569000
C	8.019074000	8.032713000	11.890822000
C	7.381115000	11.356382000	12.546470000
H	8.329436000	11.736392000	12.941152000
H	7.606398000	10.660721000	11.742779000
H	6.889025000	10.799489000	13.349433000
C	7.763655000	12.613609000	6.648748000
H	7.067955000	11.925452000	7.133110000
H	8.096017000	12.172705000	5.705381000
H	7.205829000	13.524108000	6.417126000
C	8.690627000	14.866747000	9.167659000
H	9.256700000	15.474808000	8.472590000
C	7.319915000	5.391954000	9.644707000
H	8.236201000	4.806142000	9.514660000
H	6.876428000	5.102816000	10.602313000
H	7.596173000	6.440987000	9.705594000
C	6.493574000	12.517104000	12.074817000
C	10.407358000	7.530523000	9.903719000
H	11.181050000	7.859251000	9.201468000
H	9.438550000	7.624405000	9.414071000
H	10.567866000	6.465957000	10.103978000
C	11.910242000	8.099189000	11.766241000
H	12.098204000	7.039243000	11.965877000
H	12.093309000	8.663306000	12.686674000
H	12.647131000	8.429138000	11.026305000

C	5.919049000	3.639843000	8.622650000
H	5.166302000	3.358972000	7.879022000
H	5.469803000	3.488141000	9.610077000
H	6.769381000	2.954419000	8.538517000
C	5.054782000	5.935974000	8.609750000
H	5.220395000	7.002333000	8.448825000
H	4.594597000	5.798645000	9.592706000
H	4.339469000	5.606610000	7.853917000
C	10.501213000	8.342200000	11.205939000
C	7.029696000	7.610962000	12.813953000
C	5.500068000	7.682292000	12.640584000
C	9.764520000	7.348370000	13.419795000
H	10.811582000	7.239795000	13.674426000
C	5.012193000	8.307004000	11.335784000
H	5.307241000	9.354878000	11.256838000
H	3.921579000	8.286930000	11.320745000
H	5.342295000	7.729894000	10.472143000
C	7.513133000	14.678973000	11.243060000
H	7.164160000	15.141690000	12.158157000
C	7.674085000	4.624604000	4.942245000
H	7.686350000	3.837781000	4.193258000
C	9.835186000	13.939023000	6.767439000
H	9.283075000	14.839074000	6.479587000
H	10.180052000	13.460536000	5.844655000
H	10.721145000	14.241844000	7.335230000
C	4.913064000	6.260278000	12.701053000
H	5.284839000	5.646722000	11.876116000
H	3.820603000	6.304427000	12.625297000
H	5.160343000	5.751013000	13.636562000
C	6.153281000	13.351085000	13.317539000
H	5.492987000	14.192595000	13.083196000
H	7.047975000	13.736695000	13.817926000
H	5.625311000	12.712877000	14.034105000
C	10.404380000	9.849642000	10.921735000
H	11.188464000	10.146943000	10.216953000
H	10.544955000	10.424861000	11.842388000
H	9.442824000	10.142686000	10.501407000
C	9.783239000	8.114622000	3.976324000
H	10.527489000	7.311961000	3.961307000
H	10.313707000	9.059882000	3.820089000
H	9.105133000	7.975002000	3.128218000
C	8.809723000	6.926022000	14.338375000
H	9.114189000	6.497123000	15.288910000
C	9.034656000	8.180194000	5.315898000
C	8.219391000	15.453743000	10.333104000
H	8.412545000	16.503099000	10.537489000
C	4.890987000	8.508451000	13.788439000
H	5.117767000	8.083042000	14.769679000
H	3.801067000	8.540657000	13.685655000
H	5.262232000	9.536903000	13.774388000
C	7.468118000	7.059936000	14.026538000
H	6.728999000	6.727252000	14.748755000
C	10.091345000	8.433720000	6.400678000
H	10.763436000	7.573545000	6.482677000
H	9.642108000	8.590670000	7.380575000
H	10.696084000	9.311370000	6.152454000
C	8.058974000	9.358201000	5.204712000
H	8.602698000	10.278319000	4.973342000
H	7.488851000	9.521050000	6.123105000
H	7.334160000	9.183974000	4.404829000
U	2.921160000	10.007061000	7.810278000
O	2.464160000	11.683843000	9.090081000
O	2.405498000	8.013132000	7.172942000

O	2.152210000	10.894025000	6.044104000
C	2.243685000	6.700738000	6.892668000
C	2.212099000	12.710509000	9.933900000
C	2.695041000	14.014474000	9.628071000
C	4.807690000	7.578330000	5.234632000
H	5.287954000	8.254547000	4.521258000
H	4.656248000	8.113131000	6.173937000
H	5.500010000	6.761243000	5.441187000
C	2.814915000	6.151027000	5.710826000
C	0.415854000	11.729988000	4.651905000
C	3.410536000	14.337028000	8.314657000
C	1.487100000	12.478646000	11.135638000
C	1.524541000	5.869167000	7.795301000
C	1.323375000	13.542030000	12.028378000
H	0.786778000	13.380848000	12.955368000
C	-0.237602000	7.485088000	8.542604000
H	-0.927685000	7.046913000	7.814509000
H	-0.828245000	7.852228000	9.387445000
H	0.244899000	8.336148000	8.062386000
C	2.500460000	15.028597000	10.569914000
H	2.877817000	16.022871000	10.364789000
C	0.778115000	6.431717000	9.008739000
C	1.804695000	11.547831000	4.915978000
C	2.556041000	8.117061000	4.168262000
H	1.632042000	7.677387000	3.777765000
H	2.288909000	8.812187000	4.959326000
H	3.019247000	8.687011000	3.357278000
C	1.772651000	7.016876000	10.017359000
H	2.399554000	7.793527000	9.574487000
H	1.242641000	7.445494000	10.872552000
H	2.444975000	6.238248000	10.388459000
C	1.501432000	4.493153000	7.552729000
H	0.981554000	3.839009000	8.241870000
C	2.466809000	14.036778000	7.141151000
H	1.551119000	14.631124000	7.229907000
H	2.939360000	14.296112000	6.189134000
H	2.183059000	12.988614000	7.101834000
C	3.507328000	7.013439000	4.652160000
C	-0.583103000	11.764601000	6.979317000
H	-1.336051000	11.308684000	7.631388000
H	0.393981000	11.631800000	7.442649000
H	-0.778193000	12.841464000	6.943249000
C	-2.083666000	11.458790000	5.054880000
H	-2.282792000	12.534289000	5.001740000
H	-2.266159000	11.023277000	4.066913000
H	-2.814245000	11.024058000	5.745265000
C	3.803243000	15.817242000	8.203730000
H	4.508172000	16.117647000	8.986175000
H	4.299755000	15.976519000	7.240391000
H	2.935199000	16.484184000	8.238945000
C	4.719373000	13.544874000	8.218377000
H	4.579387000	12.472800000	8.358837000
H	5.186116000	13.708869000	7.244421000
H	5.422161000	13.878049000	8.985916000
C	-0.669832000	11.153243000	5.571846000
C	2.788059000	12.046801000	4.025062000
C	4.317574000	11.900532000	4.145272000
C	0.051114000	12.437702000	3.506464000
H	-0.997417000	12.595982000	3.286020000
C	4.806374000	11.160756000	5.387891000
H	4.419893000	10.142357000	5.402059000
H	5.894835000	11.087255000	5.358666000
H	4.553338000	11.704555000	6.301046000

C	2.755310000	4.765854000	5.533167000
H	3.211429000	4.323392000	4.656240000
C	1.833952000	14.804889000	11.766696000
H	1.701640000	15.611619000	12.482189000
C	-0.020911000	5.355408000	9.757180000
H	0.622274000	4.577117000	10.180328000
H	-0.554785000	5.826038000	10.589583000
H	-0.766582000	4.878233000	9.113144000
C	4.976325000	13.291255000	4.151367000
H	4.655573000	13.877118000	5.016924000
H	6.065543000	13.185571000	4.197670000
H	4.738954000	13.864981000	3.251410000
C	3.911131000	6.203436000	3.411849000
H	4.652754000	5.432737000	3.644477000
H	3.050425000	5.729320000	2.927873000
H	4.367766000	6.882029000	2.683418000
C	-0.549128000	9.621300000	5.628844000
H	-1.346070000	9.206609000	6.255569000
H	-0.653436000	9.193222000	4.626486000
H	0.408110000	9.289648000	6.030205000
C	0.090915000	11.125227000	12.789947000
H	-0.768893000	11.797910000	12.705322000
H	-0.293511000	10.117448000	12.979903000
H	0.677824000	11.419722000	13.665552000
C	1.000430000	12.948685000	2.627904000
H	0.691114000	13.497379000	1.742822000
C	0.930691000	11.100796000	11.506009000
C	2.129119000	3.932072000	6.449433000
H	2.107521000	2.857613000	6.290788000
C	4.847576000	11.120047000	2.927902000
H	4.611868000	11.620348000	1.984546000
H	5.937905000	11.024764000	2.990806000
H	4.422213000	10.113455000	2.888622000
C	2.343243000	12.742609000	2.891988000
H	3.078622000	13.134505000	2.196347000
C	0.020419000	10.563339000	10.397227000
H	-0.822244000	11.239781000	10.223701000
H	0.556141000	10.465623000	9.452750000
H	-0.378673000	9.584360000	10.676964000
C	2.103921000	10.148173000	11.753618000
H	1.744307000	9.145775000	12.003627000
H	2.753299000	10.080508000	10.878425000
H	2.714403000	10.504874000	12.586735000

$[^{Ar}L_3UNU^{Ar}L_3]^{3-}$ E = -4737.09243300

U	5.733496000	7.911611000	9.142322000
O	3.647474000	7.632235000	8.194266000
O	8.024342000	7.939309000	8.743364000
O	5.767344000	5.773294000	10.008866000
N	5.468970000	9.641469000	10.285624000
C	2.455382000	7.630807000	7.652790000
C	1.314470000	7.181119000	8.400417000
C	0.052968000	7.247498000	7.805556000
H	-0.817368000	6.924855000	8.367081000
C	-0.133537000	7.718652000	6.508665000
H	-1.128668000	7.759069000	6.072505000
C	0.977482000	8.124026000	5.775605000
H	0.824694000	8.479450000	4.761584000
C	2.268355000	8.090644000	6.306284000
C	1.464669000	6.650305000	9.831687000
C	2.412772000	5.441023000	9.856352000
H	2.063496000	4.658366000	9.172208000
H	2.448577000	5.009971000	10.863558000

H	3.425574000	5.722075000	9.576979000
C	1.992446000	7.754557000	10.758232000
H	2.916864000	8.204524000	10.390604000
H	2.182409000	7.345408000	11.755616000
H	1.252544000	8.551230000	10.863790000
C	0.125136000	6.186796000	10.421646000
H	-0.600780000	7.004300000	10.490601000
H	0.295068000	5.819521000	11.440339000
H	-0.324157000	5.368373000	9.846528000
C	3.459838000	8.572547000	5.470294000
C	4.073755000	9.823688000	6.115882000
H	4.356018000	9.649105000	7.155959000
H	3.358063000	10.651018000	6.101362000
H	4.965210000	10.140157000	5.563764000
C	3.052081000	8.962305000	4.042765000
H	3.940706000	9.295574000	3.494212000
H	2.331976000	9.787604000	4.028321000
H	2.619297000	8.119789000	3.491072000
C	4.520398000	7.466698000	5.340434000
H	4.088258000	6.570143000	4.880646000
H	4.929159000	7.183786000	6.312980000
H	5.344893000	7.808833000	4.704888000
C	9.295450000	8.247600000	8.663938000
C	9.831419000	8.818306000	7.460227000
C	11.166029000	9.225372000	7.435753000
H	11.569563000	9.680855000	6.537416000
C	12.005338000	9.072133000	8.534869000
H	13.040067000	9.403249000	8.493127000
C	11.501333000	8.464987000	9.681491000
H	12.163974000	8.335178000	10.529996000
C	10.176973000	8.033784000	9.778102000
C	8.946703000	9.010186000	6.222417000
C	7.849566000	10.047143000	6.510581000
H	7.239626000	9.767272000	7.371478000
H	7.192049000	10.155574000	5.639801000
H	8.291266000	11.025597000	6.722987000
C	9.738571000	9.523909000	5.012243000
H	10.175209000	10.513046000	5.188476000
H	9.063460000	9.616121000	4.153656000
H	10.545365000	8.839178000	4.726265000
C	8.318939000	7.669414000	5.807515000
H	9.100556000	6.931725000	5.589744000
H	7.713426000	7.795245000	4.902060000
H	7.686796000	7.271626000	6.601920000
C	9.679068000	7.382358000	11.074047000
C	10.819558000	7.139644000	12.074172000
H	11.603286000	6.491141000	11.664384000
H	10.411751000	6.644326000	12.962562000
H	11.280607000	8.074732000	12.410234000
C	9.042969000	6.015318000	10.776216000
H	8.154011000	6.116778000	10.158535000
H	8.750933000	5.520275000	11.709547000
H	9.756146000	5.359982000	10.260990000
C	8.674664000	8.305831000	11.780516000
H	9.166090000	9.232602000	12.086540000
H	8.293207000	7.818054000	12.683851000
H	7.818767000	8.576874000	11.154481000
C	5.794150000	4.550207000	10.478248000
C	5.635564000	4.273794000	11.875498000
C	5.683062000	2.942912000	12.308059000
H	5.565259000	2.734037000	13.368570000
C	5.872918000	1.877294000	11.441178000
H	5.904260000	0.855771000	11.812006000

C	6.021509000	2.145600000	10.082415000
H	6.169128000	1.309431000	9.407411000
C	5.987197000	3.443238000	9.573605000
C	6.160164000	3.668739000	8.062476000
C	4.913106000	4.340196000	7.463165000
H	4.026185000	3.715323000	7.618040000
H	5.043273000	4.474348000	6.382626000
H	4.721024000	5.318072000	7.904737000
C	7.406135000	4.524496000	7.782586000
H	7.340138000	5.509486000	8.244619000
H	7.532317000	4.663295000	6.701670000
H	8.307222000	4.030260000	8.164393000
C	6.354415000	2.349643000	7.299772000
H	7.252772000	1.811169000	7.622039000
H	6.469826000	2.569541000	6.232539000
H	5.494733000	1.678104000	7.405373000
C	5.413760000	5.320349000	12.987998000
C	5.337458000	6.775568000	12.530138000
H	6.253401000	7.102183000	12.040915000
H	5.187314000	7.411305000	13.408357000
H	4.504729000	6.952126000	11.848507000
C	6.569881000	5.233899000	14.002222000
H	6.665664000	4.237778000	14.446670000
H	6.413379000	5.949641000	14.818671000
H	7.521985000	5.479951000	13.521706000
C	4.089617000	5.017766000	13.715381000
H	3.245529000	5.087696000	13.021470000
H	3.925037000	5.746875000	14.517741000
H	4.072814000	4.019546000	14.164802000
U	5.062347000	11.231253000	11.569084000
O	7.151122000	11.577555000	12.518367000
O	2.751826000	11.076530000	11.897414000
O	4.997782000	13.343131000	10.671563000
C	8.364055000	11.524037000	13.006887000
C	9.493363000	11.916092000	12.210991000
C	10.775277000	11.751862000	12.739108000
H	11.636510000	12.014562000	12.134217000
C	10.994565000	11.253186000	14.020583000
H	12.005966000	11.132735000	14.400904000
C	9.896181000	10.930167000	14.811815000
H	10.073612000	10.561543000	15.817158000
C	8.586240000	11.061152000	14.347297000
C	9.311137000	12.467957000	10.791547000
C	8.382509000	13.692962000	10.804112000
H	8.764203000	14.465124000	11.483142000
H	8.325054000	14.131849000	9.801292000
H	7.374314000	13.424057000	11.111085000
C	8.742595000	11.378637000	9.871123000
H	7.785483000	10.982064000	10.219917000
H	8.594408000	11.779916000	8.862789000
H	9.445683000	10.546878000	9.795095000
C	10.643156000	12.915885000	10.172213000
H	11.341309000	12.081445000	10.045803000
H	10.449901000	13.326178000	9.174300000
H	11.136070000	13.698387000	10.761735000
C	7.404645000	10.678124000	15.246531000
C	6.684678000	9.450743000	14.666545000
H	6.387622000	9.607236000	13.627838000
H	7.341713000	8.575850000	14.691558000
H	5.788954000	9.216538000	15.253023000
C	7.848491000	10.305049000	16.667758000
H	6.964576000	10.059975000	17.267955000
H	8.504597000	9.427965000	16.682037000

H	8.368474000	11.130350000	17.167867000
C	6.426110000	11.857088000	15.381614000
H	6.941048000	12.739061000	15.779965000
H	5.990164000	12.124536000	14.417188000
H	5.614743000	11.600678000	16.072762000
C	1.472858000	10.836110000	12.033915000
C	0.964243000	10.279180000	13.256848000
C	-0.387425000	9.941845000	13.341426000
H	-0.772083000	9.500559000	14.255164000
C	-1.266937000	10.145715000	12.282835000
H	-2.313585000	9.864446000	12.369366000
C	-0.786441000	10.738719000	11.118730000
H	-1.482120000	10.912487000	10.304797000
C	0.551351000	11.108130000	10.964580000
C	1.897489000	10.024445000	14.447119000
C	2.940210000	8.958125000	14.079159000
H	3.510952000	9.238287000	13.192268000
H	3.639156000	8.804840000	14.909500000
H	2.451588000	8.001494000	13.871192000
C	1.145847000	9.503754000	15.679746000
H	0.664479000	8.536987000	15.495398000
H	1.857463000	9.360798000	16.501150000
H	0.379990000	10.207492000	16.025913000
C	2.595253000	11.328362000	14.867647000
H	1.855456000	12.086100000	15.151798000
H	3.246554000	11.152106000	15.731860000
H	3.198635000	11.732368000	14.053105000
C	1.018822000	11.762903000	9.658642000
C	-0.148822000	12.048277000	8.703080000
H	-0.895610000	12.719673000	9.143805000
H	0.241964000	12.534868000	7.802306000
H	-0.653367000	11.130608000	8.381043000
C	1.702544000	13.106821000	9.953376000
H	2.607094000	12.969190000	10.541149000
H	1.981464000	13.608134000	9.019096000
H	1.025549000	13.774644000	10.500015000
C	1.977608000	10.831165000	8.904234000
H	1.459646000	9.916579000	8.602335000
H	2.338781000	11.324424000	7.996014000
H	2.846226000	10.542159000	9.499605000
C	4.911883000	14.560309000	10.190908000
C	5.050695000	14.830244000	8.790355000
C	4.936444000	16.153364000	8.346138000
H	5.041482000	16.357197000	7.283245000
C	4.694619000	17.216296000	9.203219000
H	4.610050000	18.231232000	8.822785000
C	4.567353000	16.954423000	10.565191000
H	4.382407000	17.788788000	11.233157000
C	4.671513000	15.665143000	11.086324000
C	4.537442000	15.450564000	12.603185000
C	5.830195000	14.849606000	13.179169000
H	6.683215000	15.507868000	12.978059000
H	5.741198000	14.740584000	14.266893000
H	6.051190000	13.869100000	12.757620000
C	3.341297000	14.540383000	12.924470000
H	3.434489000	13.560102000	12.456824000
H	3.260496000	14.393511000	14.008422000
H	2.406007000	14.994745000	12.577470000
C	4.295747000	16.768540000	13.354792000
H	3.369348000	17.262370000	13.040718000
H	4.206109000	16.555599000	14.425888000
H	5.122998000	17.476482000	13.229321000
C	5.321724000	13.787292000	7.685191000

C	5.454328000	12.338190000	8.149305000
H	4.541676000	11.967265000	8.613258000
H	5.659297000	11.710467000	7.277056000
H	6.271919000	12.198123000	8.854163000
C	4.169866000	13.822080000	6.662959000
H	4.036246000	14.810468000	6.210835000
H	4.359856000	13.108101000	5.852477000
H	3.225760000	13.540305000	7.139848000
C	6.637254000	14.141391000	6.965294000
H	7.479262000	14.104776000	7.664101000
H	6.835486000	13.417337000	6.165615000
H	6.617293000	15.139240000	6.514966000

$[^{Ar}L_3UNU^{Ar}L_3]^{2-}$ E = -4737.08007234

U	6.843095000	9.442996000	8.865274000
O	7.403039000	7.577961000	7.710205000
O	7.412716000	11.524297000	9.533146000
O	7.568511000	8.533448000	10.773758000
N	5.010645000	9.776052000	8.249184000
C	7.612161000	12.810721000	9.765926000
C	7.531431000	6.576182000	6.855120000
C	6.945377000	5.303051000	7.143299000
C	5.102501000	12.014786000	11.466464000
H	4.626547000	11.350194000	12.193187000
H	5.250565000	11.461738000	10.536749000
H	4.404560000	12.829259000	11.254793000
C	7.132513000	13.404737000	10.975840000
C	9.321252000	7.910330000	12.249941000
C	6.232598000	5.035934000	8.472891000
C	8.246230000	6.756509000	5.629642000
C	8.296695000	13.621627000	8.808370000
C	8.272208000	5.710401000	4.704790000
H	8.792937000	5.843664000	3.762985000
C	9.765969000	11.853895000	7.743769000
H	10.617428000	12.171430000	8.355549000
H	10.155702000	11.475357000	6.792974000
H	9.273876000	11.034845000	8.268105000
C	7.004782000	4.302872000	6.170590000
H	6.535690000	3.344811000	6.364190000
C	8.810951000	13.030354000	7.491723000
C	7.924415000	8.028780000	11.946572000
C	7.326215000	11.431970000	12.524596000
H	8.277966000	11.830853000	12.894083000
H	7.537040000	10.720471000	11.730121000
H	6.852328000	10.889526000	13.349834000
C	7.614238000	12.581641000	6.641887000
H	6.963229000	11.887312000	7.178207000
H	7.955184000	12.103175000	5.718904000
H	7.004147000	13.445417000	6.364546000
C	8.445354000	14.986716000	9.064967000
H	8.953088000	15.613873000	8.340419000
C	7.177171000	5.328197000	9.649997000
H	8.096096000	4.736993000	9.561018000
H	6.698825000	5.055657000	10.596936000
H	7.449884000	6.379547000	9.700640000
C	6.413796000	12.569945000	12.039821000
C	10.269603000	7.494752000	9.950173000
H	11.034161000	7.801300000	9.227106000
H	9.291176000	7.594131000	9.480520000
H	10.424756000	6.432470000	10.169116000
C	11.816584000	8.097994000	11.767825000
H	12.008835000	7.042308000	11.988074000
H	12.022063000	8.682038000	12.671573000

H	12.538438000	8.410186000	11.004856000
C	5.783553000	3.573733000	8.610583000
H	5.045048000	3.296677000	7.850678000
H	5.304975000	3.441689000	9.587502000
H	6.623305000	2.870923000	8.556868000
C	4.959227000	5.887517000	8.549032000
H	5.155488000	6.951914000	8.408156000
H	4.463266000	5.751169000	9.515823000
H	4.261645000	5.578436000	7.768436000
C	10.395046000	8.330556000	11.235227000
C	6.956405000	7.608198000	12.904898000
C	5.422846000	7.649465000	12.743547000
C	9.700350000	7.395618000	13.489052000
H	10.752651000	7.304783000	13.733461000
C	4.905645000	8.212479000	11.420967000
H	5.182320000	9.259393000	11.287233000
H	3.814062000	8.167908000	11.421336000
H	5.246285000	7.625897000	10.568225000
C	7.311492000	14.776769000	11.164428000
H	6.935865000	15.242558000	12.068839000
C	7.643937000	4.495009000	4.950295000
H	7.668663000	3.700785000	4.208624000
C	9.594884000	14.052119000	6.655524000
H	8.979269000	14.908067000	6.360092000
H	9.942064000	13.569730000	5.734902000
H	10.477074000	14.428212000	7.185565000
C	4.860019000	6.220601000	12.865913000
H	5.243127000	5.580576000	12.065296000
H	3.766414000	6.239131000	12.786538000
H	5.116061000	5.752309000	13.821062000
C	6.033489000	13.400243000	13.273110000
H	5.338805000	14.210382000	13.026347000
H	6.909064000	13.830607000	13.772884000
H	5.528825000	12.749350000	13.995744000
C	10.289976000	9.832509000	10.920886000
H	11.049960000	10.115400000	10.182870000
H	10.464953000	10.426468000	11.824540000
H	9.312579000	10.113624000	10.530723000
C	9.767172000	8.005706000	4.007904000
H	10.531311000	7.220909000	4.029704000
H	10.279117000	8.962003000	3.851974000
H	9.122590000	7.835639000	3.139099000
C	8.762415000	6.989300000	14.433990000
H	9.081358000	6.592138000	15.394114000
C	8.970038000	8.071242000	5.318313000
C	7.954269000	15.575327000	10.224223000
H	8.076632000	16.641457000	10.396935000
C	4.816538000	8.513805000	13.864865000
H	5.065390000	8.137429000	14.861678000
H	3.724281000	8.530114000	13.778712000
H	5.175242000	9.545276000	13.797038000
C	7.415793000	7.098735000	14.127081000
H	6.687500000	6.777979000	14.867123000
C	9.978041000	8.390036000	6.433554000
H	10.690938000	7.566464000	6.551184000
H	9.480269000	8.533044000	7.392024000
H	10.545353000	9.295594000	6.192476000
C	7.956182000	9.213366000	5.163084000
H	8.474743000	10.168032000	5.028217000
H	7.290281000	9.292620000	6.024772000
H	7.323829000	9.047715000	4.285721000
U	2.889468000	10.020194000	7.764070000
O	2.277873000	11.749749000	9.121168000

O	2.269481000	7.926771000	7.100601000
O	2.173243000	10.974138000	5.850352000
C	2.138651000	6.639625000	6.844024000
C	2.059698000	12.744930000	9.959424000
C	2.533086000	14.065760000	9.663606000
C	4.718147000	7.510592000	5.207750000
H	5.219699000	8.149108000	4.472930000
H	4.536708000	8.103746000	6.105798000
H	5.404648000	6.704689000	5.474832000
C	2.722447000	6.065976000	5.667518000
C	0.359185000	11.871673000	4.600204000
C	3.244617000	14.387450000	8.343933000
C	1.358022000	12.514697000	11.187115000
C	1.423261000	5.791387000	7.750150000
C	1.200993000	13.572502000	12.085958000
H	0.682580000	13.404271000	13.023682000
C	-0.330105000	7.428378000	8.498507000
H	-1.040585000	6.988464000	7.789522000
H	-0.900992000	7.813937000	9.350460000
H	0.166576000	8.262543000	8.003517000
C	2.345773000	15.073158000	10.613237000
H	2.717023000	16.071494000	10.409742000
C	0.683471000	6.369354000	8.962338000
C	1.767609000	11.668458000	4.804429000
C	2.498274000	8.050404000	4.124545000
H	1.559580000	7.633224000	3.741416000
H	2.263051000	8.760109000	4.914724000
H	2.974279000	8.602960000	3.306079000
C	1.682227000	6.974644000	9.959169000
H	2.312970000	7.733795000	9.492499000
H	1.150945000	7.431027000	10.801012000
H	2.343545000	6.198648000	10.357638000
C	1.418459000	4.413550000	7.524290000
H	0.905519000	3.759620000	8.221190000
C	2.328015000	14.050704000	7.158290000
H	1.386640000	14.608195000	7.226090000
H	2.808918000	14.323673000	6.212538000
H	2.098234000	12.989207000	7.120021000
C	3.428127000	6.930136000	4.615823000
C	-0.567502000	11.794072000	6.957477000
H	-1.312584000	11.320944000	7.608016000
H	0.418267000	11.623311000	7.390544000
H	-0.752308000	12.873807000	6.966861000
C	-2.122764000	11.522777000	5.066715000
H	-2.359103000	12.592668000	5.074614000
H	-2.319223000	11.133931000	4.061320000
H	-2.821718000	11.030112000	5.751758000
C	3.606685000	15.874878000	8.222886000
H	4.309662000	16.194028000	9.000353000
H	4.095438000	16.042199000	7.256480000
H	2.724980000	16.525045000	8.261127000
C	4.565694000	13.612863000	8.242003000
H	4.434763000	12.536309000	8.364654000
H	5.025297000	13.790103000	7.264829000
H	5.269272000	13.951683000	9.007666000
C	-0.685894000	11.235576000	5.530228000
C	2.693863000	12.232627000	3.874366000
C	4.227727000	12.064180000	3.892207000
C	-0.063062000	12.652379000	3.524769000
H	-1.122704000	12.820482000	3.368879000
C	4.801561000	11.252975000	5.052130000
H	4.398728000	10.242211000	5.084958000
H	5.885269000	11.173953000	4.923037000

H	4.624148000	11.731113000	6.016667000
C	2.679675000	4.679177000	5.506830000
H	3.150581000	4.229919000	4.639759000
C	1.694942000	14.844204000	11.820835000
H	1.566422000	15.648963000	12.540197000
C	-0.112222000	5.301334000	9.725418000
H	0.533364000	4.525784000	10.151405000
H	-0.642472000	5.776836000	10.558256000
H	-0.861267000	4.815755000	9.090121000
C	4.899604000	13.449160000	3.941605000
H	4.623137000	13.982191000	4.856722000
H	5.990238000	13.337869000	3.934335000
H	4.626328000	14.080357000	3.090359000
C	3.842112000	6.116398000	3.380999000
H	4.584563000	5.348623000	3.622864000
H	2.986734000	5.635089000	2.892549000
H	4.303263000	6.791132000	2.651093000
C	-0.529236000	9.705744000	5.539091000
H	-1.305404000	9.252738000	6.167045000
H	-0.642289000	9.304354000	4.525785000
H	0.443217000	9.394602000	5.919157000
C	0.037107000	11.118662000	12.865631000
H	-0.839110000	11.775028000	12.818422000
H	-0.321527000	10.102325000	13.063598000
H	0.647582000	11.420092000	13.723435000
C	0.836843000	13.229137000	2.632816000
H	0.482781000	13.836445000	1.803754000
C	0.833890000	11.120854000	11.553975000
C	2.056295000	3.843763000	6.428021000
H	2.047785000	2.766843000	6.280952000
C	4.659513000	11.351168000	2.596227000
H	4.357500000	11.900460000	1.698944000
H	5.750578000	11.240734000	2.569450000
H	4.219830000	10.349933000	2.540761000
C	2.191564000	13.001687000	2.816581000
H	2.891357000	13.439068000	2.109326000
C	-0.105468000	10.576331000	10.467568000
H	-0.961704000	11.245483000	10.326499000
H	0.405039000	10.479649000	9.509567000
H	-0.490302000	9.593713000	10.761020000
C	2.025435000	10.173831000	11.754046000
H	1.679558000	9.160537000	11.983776000
H	2.660180000	10.133737000	10.866983000
H	2.642996000	10.513961000	12.590083000

$[^{Ar}L_3UNU^{Ar}L_3]^{1-}$ E = -4737.03020664

U	6.843095000	9.442996000	8.865274000
O	7.403039000	7.577961000	7.710205000
O	7.412716000	11.524297000	9.533146000
O	7.568511000	8.533448000	10.773758000
N	5.010645000	9.776052000	8.249184000
C	7.612161000	12.810721000	9.765926000
C	7.531431000	6.576182000	6.855120000
C	6.945377000	5.303051000	7.143299000
C	5.102501000	12.014786000	11.466464000
H	4.626547000	11.350194000	12.193187000
H	5.250565000	11.461738000	10.536749000
H	4.404560000	12.829259000	11.254793000
C	7.132513000	13.404737000	10.975840000
C	9.321252000	7.910330000	12.249941000
C	6.232598000	5.035934000	8.472891000
C	8.246230000	6.756509000	5.629642000
C	8.296695000	13.621627000	8.808370000

C	8.272208000	5.710401000	4.704790000
H	8.792937000	5.843664000	3.762985000
C	9.765969000	11.853895000	7.743769000
H	10.617428000	12.171430000	8.355549000
H	10.155702000	11.475357000	6.792974000
H	9.273876000	11.034845000	8.268105000
C	7.004782000	4.302872000	6.170590000
H	6.535690000	3.344811000	6.364190000
C	8.810951000	13.030354000	7.491723000
C	7.924415000	8.028780000	11.946572000
C	7.326215000	11.431970000	12.524596000
H	8.277966000	11.830853000	12.894083000
H	7.537040000	10.720471000	11.730121000
H	6.852328000	10.889526000	13.349834000
C	7.614238000	12.581641000	6.641887000
H	6.963229000	11.887312000	7.178207000
H	7.955184000	12.103175000	5.718904000
H	7.004147000	13.445417000	6.364546000
C	8.445354000	14.986716000	9.064967000
H	8.953088000	15.613873000	8.340419000
C	7.177171000	5.328197000	9.649997000
H	8.096096000	4.736993000	9.561018000
H	6.698825000	5.055657000	10.596936000
H	7.449884000	6.379547000	9.700640000
C	6.413796000	12.569945000	12.039821000
C	10.269603000	7.494752000	9.950173000
H	11.034161000	7.801300000	9.227106000
H	9.291176000	7.594131000	9.480520000
H	10.424756000	6.432470000	10.169116000
C	11.816584000	8.097994000	11.767825000
H	12.008835000	7.042308000	11.988074000
H	12.022063000	8.682038000	12.671573000
H	12.538438000	8.410186000	11.004856000
C	5.783553000	3.573733000	8.610583000
H	5.045048000	3.296677000	7.850678000
H	5.304975000	3.441689000	9.587502000
H	6.623305000	2.870923000	8.556868000
C	4.959227000	5.887517000	8.549032000
H	5.155488000	6.951914000	8.408156000
H	4.463266000	5.751169000	9.515823000
H	4.261645000	5.578436000	7.768436000
C	10.395046000	8.330556000	11.235227000
C	6.956405000	7.608198000	12.904898000
C	5.422846000	7.649465000	12.743547000
C	9.700350000	7.395618000	13.489052000
H	10.752651000	7.304783000	13.733461000
C	4.905645000	8.212479000	11.420967000
H	5.182320000	9.259393000	11.287233000
H	3.814062000	8.167908000	11.421336000
H	5.246285000	7.625897000	10.568225000
C	7.311492000	14.776769000	11.164428000
H	6.935865000	15.242558000	12.068839000
C	7.643937000	4.495009000	4.950295000
H	7.668663000	3.700785000	4.208624000
C	9.594884000	14.052119000	6.655524000
H	8.979269000	14.908067000	6.360092000
H	9.942064000	13.569730000	5.734902000
H	10.477074000	14.428212000	7.185565000
C	4.860019000	6.220601000	12.865913000
H	5.243127000	5.580576000	12.065296000
H	3.766414000	6.239131000	12.786538000
H	5.116061000	5.752309000	13.821062000
C	6.033489000	13.400243000	13.273110000

H	5.338805000	14.210382000	13.026347000
H	6.909064000	13.830607000	13.772884000
H	5.528825000	12.749350000	13.995744000
C	10.289976000	9.832509000	10.920886000
H	11.049960000	10.115400000	10.182870000
H	10.464953000	10.426468000	11.824540000
H	9.312579000	10.113624000	10.530723000
C	9.767172000	8.005706000	4.007904000
H	10.531311000	7.220909000	4.029704000
H	10.279117000	8.962003000	3.851974000
H	9.122590000	7.835639000	3.139099000
C	8.762415000	6.989300000	14.433990000
H	9.081358000	6.592138000	15.394114000
C	8.970038000	8.071242000	5.318313000
C	7.954269000	15.575327000	10.224223000
H	8.076632000	16.641457000	10.396935000
C	4.816538000	8.513805000	13.864865000
H	5.065390000	8.137429000	14.861678000
H	3.724281000	8.530114000	13.778712000
H	5.175242000	9.545276000	13.797038000
C	7.415793000	7.098735000	14.127081000
H	6.687500000	6.777979000	14.867123000
C	9.978041000	8.390036000	6.433554000
H	10.690938000	7.566464000	6.551184000
H	9.480269000	8.533044000	7.392024000
H	10.545353000	9.295594000	6.192476000
C	7.956182000	9.213366000	5.163084000
H	8.474743000	10.168032000	5.028217000
H	7.290281000	9.292620000	6.024772000
H	7.323829000	9.047715000	4.285721000
U	3.054153000	10.001239000	7.801733000
O	2.442558000	11.730794000	9.158831000
O	2.434166000	7.907816000	7.138264000
O	2.337928000	10.955183000	5.888015000
C	2.303336000	6.620670000	6.881687000
C	2.224383000	12.725975000	9.997087000
C	2.697771000	14.046805000	9.701269000
C	4.882832000	7.491637000	5.245413000
H	5.384384000	8.130153000	4.510593000
H	4.701393000	8.084791000	6.143461000
H	5.569333000	6.685734000	5.512495000
C	2.887132000	6.047021000	5.705181000
C	0.523870000	11.852718000	4.637867000
C	3.409302000	14.368495000	8.381596000
C	1.522707000	12.495742000	11.224778000
C	1.587946000	5.772432000	7.787813000
C	1.365678000	13.553547000	12.123621000
H	0.847265000	13.385316000	13.061345000
C	-0.165420000	7.409423000	8.536170000
H	-0.875900000	6.969509000	7.827185000
H	-0.736307000	7.794982000	9.388123000
H	0.331261000	8.243588000	8.041180000
C	2.510458000	15.054203000	10.650900000
H	2.881708000	16.052539000	10.447405000
C	0.848156000	6.350399000	9.000001000
C	1.932294000	11.649503000	4.842092000
C	2.662959000	8.031449000	4.162208000
H	1.724265000	7.614269000	3.779079000
H	2.427736000	8.741154000	4.952387000
H	3.138964000	8.584005000	3.343742000
C	1.846912000	6.955689000	9.996832000
H	2.477655000	7.714840000	9.530162000
H	1.315630000	7.412072000	10.838675000

H	2.508230000	6.179693000	10.395301000
C	1.583144000	4.394595000	7.561953000
H	1.070204000	3.740665000	8.258853000
C	2.492700000	14.031749000	7.195953000
H	1.551325000	14.589240000	7.263753000
H	2.973603000	14.304718000	6.250201000
H	2.262919000	12.970252000	7.157684000
C	3.592812000	6.911181000	4.653486000
C	-0.402817000	11.775117000	6.995140000
H	-1.147899000	11.301989000	7.645679000
H	0.582952000	11.604356000	7.428207000
H	-0.587623000	12.854852000	7.004524000
C	-1.958079000	11.503822000	5.104378000
H	-2.194418000	12.573713000	5.112277000
H	-2.154538000	11.114976000	4.098983000
H	-2.657033000	11.011157000	5.789421000
C	3.771370000	15.855923000	8.260549000
H	4.474347000	16.175073000	9.038016000
H	4.260123000	16.023244000	7.294143000
H	2.889665000	16.506090000	8.298790000
C	4.730379000	13.593908000	8.279666000
H	4.599448000	12.517354000	8.402317000
H	5.189982000	13.771148000	7.302492000
H	5.433957000	13.932728000	9.045329000
C	-0.521209000	11.216621000	5.567891000
C	2.858548000	12.213672000	3.912029000
C	4.392412000	12.045225000	3.929870000
C	0.101623000	12.633424000	3.562432000
H	-0.958019000	12.801527000	3.406542000
C	4.966246000	11.234020000	5.089793000
H	4.563413000	10.223256000	5.122621000
H	6.049954000	11.154998000	4.960700000
H	4.788833000	11.712158000	6.054330000
C	2.844360000	4.660222000	5.544493000
H	3.315266000	4.210964000	4.677422000
C	1.859627000	14.825249000	11.858498000
H	1.731107000	15.630008000	12.577860000
C	0.052463000	5.282379000	9.763081000
H	0.698049000	4.506829000	10.189068000
H	-0.477787000	5.757881000	10.595919000
H	-0.696582000	4.796800000	9.127784000
C	5.064289000	13.430205000	3.979268000
H	4.787822000	13.963236000	4.894385000
H	6.154923000	13.318914000	3.971998000
H	4.791013000	14.061402000	3.128022000
C	4.006797000	6.097443000	3.418662000
H	4.749248000	5.329668000	3.660527000
H	3.151419000	5.616134000	2.930212000
H	4.467948000	6.772177000	2.688756000
C	-0.364551000	9.686789000	5.576754000
H	-1.140719000	9.233783000	6.204708000
H	-0.477604000	9.285399000	4.563448000
H	0.607902000	9.375647000	5.956820000
C	0.201792000	11.099707000	12.903294000
H	-0.674425000	11.756073000	12.856085000
H	-0.156842000	10.083370000	13.101261000
H	0.812267000	11.401137000	13.761098000
C	1.001528000	13.210182000	2.670479000
H	0.647466000	13.817490000	1.841417000
C	0.998575000	11.101899000	11.591638000
C	2.220980000	3.824808000	6.465684000
H	2.212470000	2.747888000	6.318615000
C	4.824198000	11.332213000	2.633890000

H	4.522185000	11.881505000	1.736607000
H	5.915263000	11.221779000	2.607113000
H	4.384515000	10.330978000	2.578424000
C	2.356249000	12.982732000	2.854244000
H	3.056042000	13.420113000	2.146989000
C	0.059217000	10.557376000	10.505231000
H	-0.797019000	11.226528000	10.364162000
H	0.569724000	10.460694000	9.547230000
H	-0.325617000	9.574758000	10.798683000
C	2.190120000	10.154876000	11.791709000
H	1.844243000	9.141582000	12.021439000
H	2.824865000	10.114782000	10.904646000
H	2.807681000	10.495006000	12.627746000

$[^{Ar}L_3USU^{Ar}L_3]^{2-}$ E = -4692.61324984

U	7.279922000	9.436830000	9.014524000
O	7.595327000	7.643495000	7.690769000
O	7.733362000	11.567661000	9.643744000
O	7.721050000	8.440202000	10.928677000
S	4.790702000	9.737330000	8.269279000
C	7.774647000	12.869573000	9.874098000
C	7.690309000	6.701922000	6.769159000
C	7.140531000	5.400802000	7.003460000
C	5.173489000	11.908211000	11.415433000
H	4.705747000	11.207364000	12.114352000
H	5.393815000	11.370685000	10.490278000
H	4.436399000	12.679222000	11.174597000
C	7.128064000	13.427825000	11.023834000
C	9.412061000	7.648878000	12.395704000
C	6.393138000	5.064908000	8.300811000
C	8.336286000	6.982609000	5.524214000
C	8.464130000	13.736990000	8.968836000
C	8.430757000	5.966036000	4.570872000
H	8.919051000	6.164185000	3.623028000
C	10.282989000	12.161905000	8.211602000
H	11.007463000	12.638481000	8.881856000
H	10.832319000	11.768096000	7.348673000
H	9.832224000	11.328033000	8.750254000
C	7.266085000	4.435777000	6.000987000
H	6.851015000	3.446821000	6.159686000
C	9.223615000	13.181041000	7.757683000
C	8.029184000	7.839840000	12.068113000
C	7.370067000	11.471790000	12.601400000
H	8.260266000	11.937276000	13.041024000
H	7.693245000	10.780994000	11.826443000
H	6.875695000	10.889923000	13.387267000
C	8.236517000	12.537170000	6.773115000
H	7.659920000	11.737926000	7.241916000
H	8.772791000	12.123437000	5.912295000
H	7.522620000	13.279697000	6.402755000
C	8.422005000	15.115419000	9.190438000
H	8.924575000	15.783455000	8.499623000
C	7.292785000	5.306282000	9.523871000
H	8.225277000	4.735995000	9.437211000
H	6.785680000	4.976395000	10.437371000
H	7.545790000	6.357471000	9.641508000
C	6.414443000	12.545304000	12.055006000
C	10.449013000	7.412935000	10.098240000
H	11.234289000	7.782127000	9.427279000
H	9.486762000	7.554423000	9.605332000
H	10.596584000	6.334839000	10.224081000
C	11.926239000	7.830653000	12.021246000
H	12.094551000	6.757079000	12.158601000

H	12.103955000	8.332061000	12.978890000
H	12.682336000	8.192721000	11.315892000
C	5.952431000	3.594423000	8.351763000
H	5.250654000	3.346664000	7.548147000
H	5.432135000	3.414640000	9.299160000
H	6.799044000	2.899238000	8.303896000
C	5.111918000	5.907843000	8.392003000
H	5.305150000	6.978992000	8.311662000
H	4.605465000	5.723825000	9.345858000
H	4.419047000	5.631768000	7.591626000
C	10.530056000	8.131368000	11.456283000
C	7.017359000	7.386861000	12.963866000
C	5.491194000	7.526356000	12.772720000
C	9.735629000	7.015159000	13.595254000
H	10.775942000	6.861855000	13.859105000
C	5.035742000	8.163037000	11.460869000
H	5.384968000	9.189145000	11.359229000
H	3.942253000	8.194437000	11.436547000
H	5.360282000	7.595206000	10.589524000
C	7.124067000	14.815861000	11.180078000
H	6.615906000	15.252791000	12.032148000
C	7.906709000	4.698150000	4.794400000
H	7.990652000	3.924455000	4.035494000
C	9.970966000	14.275209000	6.983617000
H	9.290674000	15.021243000	6.559484000
H	10.512629000	13.816754000	6.148633000
H	10.705145000	14.793922000	7.609840000
C	4.836163000	6.133905000	12.836420000
H	5.187543000	5.500174000	12.016110000
H	3.748138000	6.230165000	12.741778000
H	5.041609000	5.611647000	13.776153000
C	5.915911000	13.349859000	13.264506000
H	5.162182000	14.092837000	12.984049000
H	6.730715000	13.859021000	13.792640000
H	5.440374000	12.663180000	13.973871000
C	10.458031000	9.655798000	11.263608000
H	11.267378000	9.992556000	10.604987000
H	10.569694000	10.171512000	12.223663000
H	9.510876000	9.977099000	10.830061000
C	9.542626000	8.461515000	3.831874000
H	10.420547000	7.807384000	3.782697000
H	9.885172000	9.487400000	3.655427000
H	8.870114000	8.198321000	3.008817000
C	8.755483000	6.570134000	14.478371000
H	9.032188000	6.081433000	15.408930000
C	8.841619000	8.393409000	5.196747000
C	7.748399000	15.666575000	10.274082000
H	7.722874000	16.743410000	10.419579000
C	4.924656000	8.394555000	13.912050000
H	5.124535000	7.968905000	14.900645000
H	3.838075000	8.495639000	13.804052000
H	5.359144000	9.398736000	13.884158000
C	7.422176000	6.763456000	14.151952000
H	6.661842000	6.419116000	14.848096000
C	9.870921000	8.889680000	6.226935000
H	10.756200000	8.243253000	6.228760000
H	9.479849000	8.893894000	7.245168000
H	10.196263000	9.904734000	5.975273000
C	7.632634000	9.340534000	5.116460000
H	7.958719000	10.368718000	4.932753000
H	7.023292000	9.324455000	6.023690000
H	6.982492000	9.043145000	4.288343000
U	2.254728000	10.050817000	7.733347000

O	2.056551000	11.914039000	9.011021000
O	1.961655000	7.902546000	7.052621000
O	1.879906000	11.010006000	5.786623000
C	1.909780000	6.597878000	6.857721000
C	2.039805000	12.885920000	9.905051000
C	2.662307000	14.146095000	9.626147000
C	4.432063000	7.498498000	5.186496000
H	4.886341000	8.176537000	4.456412000
H	4.242704000	8.060993000	6.103769000
H	5.164821000	6.719150000	5.418500000
C	2.475317000	6.014855000	5.677878000
C	0.121642000	11.664976000	4.328858000
C	3.372901000	14.416917000	8.293963000
C	1.406759000	12.682101000	11.172874000
C	1.297134000	5.748934000	7.833722000
C	1.426502000	13.713980000	12.113395000
H	0.956054000	13.566159000	13.079455000
C	-0.370775000	7.379086000	8.825802000
H	-1.200826000	6.927398000	8.270768000
H	-0.773151000	7.788374000	9.759305000
H	0.013764000	8.200324000	8.219490000
C	2.652348000	15.128820000	10.618736000
H	3.135780000	16.080306000	10.428173000
C	0.712542000	6.329133000	9.126641000
C	1.519487000	11.530491000	4.621913000
C	2.198654000	7.966223000	4.096680000
H	1.257332000	7.524822000	3.748391000
H	1.969752000	8.695787000	4.869781000
H	2.648338000	8.498815000	3.251368000
C	1.849009000	6.935657000	9.963800000
H	2.423020000	7.682798000	9.410254000
H	1.450339000	7.399574000	10.872005000
H	2.553179000	6.155252000	10.267168000
C	1.292980000	4.368477000	7.623300000
H	0.841249000	3.716194000	8.362869000
C	2.418200000	14.182257000	7.111426000
H	1.509689000	14.786177000	7.223304000
H	2.900753000	14.478823000	6.173612000
H	2.124826000	13.138632000	7.023198000
C	3.154617000	6.874471000	4.605452000
C	-0.821470000	12.039013000	6.644245000
H	-1.556325000	11.693600000	7.381436000
H	0.171441000	11.968150000	7.088907000
H	-1.013625000	13.100721000	6.454075000
C	-2.374023000	11.480900000	4.821672000
H	-2.560234000	12.541800000	4.622879000
H	-2.588073000	10.915907000	3.908062000
H	-3.093616000	11.158429000	5.582571000
C	3.869206000	15.866404000	8.184281000
H	4.617518000	16.104959000	8.947581000
H	4.351194000	16.000429000	7.209371000
H	3.053404000	16.596095000	8.252296000
C	4.614873000	13.519324000	8.186874000
H	4.375840000	12.459125000	8.285081000
H	5.111414000	13.668989000	7.221078000
H	5.333053000	13.774834000	8.971076000
C	-0.950402000	11.230186000	5.340969000
C	2.487844000	11.949779000	3.663409000
C	4.023468000	11.890032000	3.815406000
C	-0.260405000	12.196615000	3.097700000
H	-1.313221000	12.300959000	2.860675000
C	4.541375000	11.334932000	5.140540000
H	4.257366000	10.295242000	5.292540000

H	5.635668000	11.376367000	5.145792000
H	4.191864000	11.910286000	5.995701000
C	2.441958000	4.625705000	5.537390000
H	2.882459000	4.170101000	4.657534000
C	2.045316000	14.931545000	11.854870000
H	2.052700000	15.717899000	12.605377000
C	0.047437000	5.258459000	10.002461000
H	0.760455000	4.495239000	10.331010000
H	-0.359778000	5.732513000	10.902741000
H	-0.780881000	4.757749000	9.488613000
C	4.597755000	13.312462000	3.673149000
H	4.221827000	13.962858000	4.469163000
H	5.691223000	13.285286000	3.751831000
H	4.345075000	13.775119000	2.714126000
C	3.582704000	6.048375000	3.384035000
H	4.330697000	5.290766000	3.640726000
H	2.735336000	5.552026000	2.896380000
H	4.042121000	6.717336000	2.647841000
C	-0.853378000	9.718427000	5.612149000
H	-1.587975000	9.419921000	6.369730000
H	-1.067445000	9.153691000	4.698207000
H	0.135047000	9.407867000	5.952396000
C	0.094155000	11.345647000	12.906219000
H	-0.713045000	12.084758000	12.957623000
H	-0.346419000	10.360569000	13.097647000
H	0.800126000	11.549088000	13.717994000
C	0.676993000	12.600351000	2.151130000
H	0.356693000	13.009234000	1.196344000
C	0.777284000	11.330989000	11.531469000
C	1.862903000	3.795740000	6.492109000
H	1.853157000	2.717679000	6.353799000
C	4.607072000	11.003548000	2.698898000
H	4.368330000	11.377497000	1.698117000
H	5.699010000	10.959075000	2.787244000
H	4.222324000	9.981972000	2.776417000
C	2.024655000	12.471922000	2.447798000
H	2.751408000	12.789046000	1.704632000
C	-0.309932000	10.937331000	10.517382000
H	-1.131425000	11.662375000	10.538604000
H	0.072874000	10.915581000	9.496602000
H	-0.723757000	9.953042000	10.763275000
C	1.886274000	10.268595000	11.603510000
H	1.459158000	9.278311000	11.793162000
H	2.486897000	10.224656000	10.690948000
H	2.571552000	10.500162000	12.424337000

$[^{Ar}L_3USU^{Ar}L_3]^{1-}$ E = -4692.54157845

U	7.467130000	9.449123000	8.875977000
O	7.621249000	7.635720000	7.711735000
O	7.671224000	11.455865000	9.649388000
O	7.744066000	8.428583000	10.728271000
S	5.049816000	9.801294000	8.317567000
C	7.722408000	12.777157000	9.898152000
C	7.750871000	6.642777000	6.815062000
C	7.179880000	5.363013000	7.070784000
C	5.271088000	11.776283000	11.579977000
H	4.811891000	11.090468000	12.297749000
H	5.453940000	11.232840000	10.651357000
H	4.545985000	12.566132000	11.360960000
C	7.200345000	13.287337000	11.120753000
C	9.521875000	7.732751000	12.155764000
C	6.425108000	5.040094000	8.364688000
C	8.454075000	6.889224000	5.602208000

C	8.295002000	13.656672000	8.935418000
C	8.542458000	5.859817000	4.661498000
H	9.067164000	6.029891000	3.729002000
C	9.943960000	12.117586000	7.777524000
H	10.793536000	12.557303000	8.310907000
H	10.301910000	11.764849000	6.804973000
H	9.626381000	11.252931000	8.362581000
C	7.300297000	4.383256000	6.080641000
H	6.859914000	3.407360000	6.244524000
C	8.831437000	13.160333000	7.587423000
C	8.124002000	7.873757000	11.892058000
C	7.530469000	11.292365000	12.632877000
H	8.467582000	11.739480000	12.983502000
H	7.764152000	10.589510000	11.838332000
H	7.100390000	10.726410000	13.465182000
C	7.666195000	12.608116000	6.752240000
H	7.106308000	11.814284000	7.253476000
H	8.029391000	12.229882000	5.791983000
H	6.946732000	13.404937000	6.546306000
C	8.314539000	15.025522000	9.218524000
H	8.738895000	15.714314000	8.498265000
C	7.330899000	5.287507000	9.580502000
H	8.263395000	4.718725000	9.490467000
H	6.830355000	4.958846000	10.496891000
H	7.579930000	6.339118000	9.694551000
C	6.550182000	12.383657000	12.172292000
C	10.471190000	7.365832000	9.843635000
H	11.221621000	7.700094000	9.119024000
H	9.487775000	7.434699000	9.376854000
H	10.649509000	6.303839000	10.041838000
C	12.017506000	7.929535000	11.664622000
H	12.212337000	6.867456000	11.844989000
H	12.224375000	8.481823000	12.587072000
H	12.733890000	8.270720000	10.909393000
C	5.978979000	3.571937000	8.427225000
H	5.271948000	3.321594000	7.629318000
H	5.461758000	3.404924000	9.378234000
H	6.822042000	2.872877000	8.383586000
C	5.146930000	5.885698000	8.434011000
H	5.346567000	6.955605000	8.366139000
H	4.616843000	5.693230000	9.372273000
H	4.478155000	5.617340000	7.613351000
C	10.592557000	8.175746000	11.145882000
C	7.161480000	7.428895000	12.837101000
C	5.624774000	7.491527000	12.711777000
C	9.915229000	7.164914000	13.367520000
H	10.969319000	7.052030000	13.590171000
C	5.088375000	8.092470000	11.415731000
H	5.387392000	9.129563000	11.286509000
H	3.995104000	8.071147000	11.445460000
H	5.400489000	7.530029000	10.538091000
C	7.249391000	14.667844000	11.333030000
H	6.846444000	15.078893000	12.250566000
C	7.966158000	4.616817000	4.884387000
H	8.040225000	3.833583000	4.134894000
C	9.453864000	14.290263000	6.752943000
H	8.722032000	15.062741000	6.497328000
H	9.827947000	13.872578000	5.811795000
H	10.298384000	14.766685000	7.262235000
C	5.047920000	6.066924000	12.815284000
H	5.389002000	5.446246000	11.981373000
H	3.953041000	6.107189000	12.776948000
H	5.328577000	5.564190000	13.745455000

C	6.129240000	13.161446000	13.427469000
H	5.375148000	13.924219000	13.207485000
H	6.978595000	13.641913000	13.926452000
H	5.681595000	12.460781000	14.140384000
C	10.498043000	9.686460000	10.880096000
H	11.210307000	9.983763000	10.102102000
H	10.738956000	10.250320000	11.787072000
H	9.501683000	10.018410000	10.581652000
C	9.823357000	8.271339000	3.953349000
H	10.655582000	7.559789000	3.937131000
H	10.238882000	9.271506000	3.789911000
H	9.162438000	8.051035000	3.109366000
C	8.986988000	6.734148000	14.308913000
H	9.317110000	6.297015000	15.247284000
C	9.074039000	8.256004000	5.293932000
C	7.796672000	15.538079000	10.399592000
H	7.820323000	16.607161000	10.592226000
C	5.062220000	8.335311000	13.871224000
H	5.316689000	7.922128000	14.851967000
H	3.969434000	8.381034000	13.802995000
H	5.444619000	9.359205000	13.828353000
C	7.637676000	6.869149000	14.031734000
H	6.919021000	6.528407000	14.770921000
C	10.116181000	8.640474000	6.357743000
H	10.897917000	7.876602000	6.418149000
H	9.699341000	8.734213000	7.362494000
H	10.591855000	9.591822000	6.099136000
C	7.959953000	9.306488000	5.176472000
H	8.387493000	10.306201000	5.054537000
H	7.274872000	9.314739000	6.028862000
H	7.342371000	9.098381000	4.298218000
U	2.414382000	10.005882000	7.787271000
O	2.140806000	11.739849000	9.206843000
O	2.227206000	7.890974000	7.041436000
O	1.967330000	11.005560000	5.908527000
C	2.164396000	6.587692000	6.797739000
C	1.953024000	12.702396000	10.102481000
C	2.435162000	14.024439000	9.845781000
C	4.695702000	7.581845000	5.198883000
H	5.159100000	8.275459000	4.489030000
H	4.472442000	8.128416000	6.116667000
H	5.430487000	6.809143000	5.440830000
C	2.761823000	6.048073000	5.616885000
C	0.179273000	11.885277000	4.611461000
C	3.153591000	14.378912000	8.538630000
C	1.281307000	12.424361000	11.332730000
C	1.520391000	5.714532000	7.726491000
C	1.120437000	13.456348000	12.261190000
H	0.614135000	13.258448000	13.199260000
C	-0.267555000	7.271675000	8.569351000
H	-1.023179000	6.779092000	7.947494000
H	-0.771139000	7.674400000	9.454684000
H	0.137254000	8.099284000	7.985663000
C	2.247942000	15.004832000	10.823790000
H	2.616305000	16.009135000	10.648231000
C	0.823196000	6.265105000	8.974836000
C	1.580056000	11.708128000	4.845499000
C	2.473438000	8.025836000	4.072010000
H	1.571152000	7.566936000	3.651110000
H	2.171608000	8.707559000	4.863956000
H	2.947626000	8.616182000	3.279846000
C	1.859237000	6.906092000	9.912023000
H	2.504956000	7.624144000	9.401966000

H	1.360977000	7.407713000	10.747521000
H	2.518405000	6.137760000	10.327737000
C	1.551153000	4.338676000	7.489462000
H	1.081636000	3.663387000	8.196242000
C	2.229777000	14.109163000	7.339998000
H	1.295911000	14.674569000	7.437564000
H	2.712876000	14.421956000	6.408224000
H	1.982605000	13.053981000	7.250776000
C	3.444032000	6.945945000	4.578091000
C	-0.803040000	11.781790000	6.944703000
H	-1.532806000	11.275681000	7.587593000
H	0.185242000	11.669793000	7.394073000
H	-1.032764000	12.852297000	6.954961000
C	-2.307527000	11.498581000	5.020021000
H	-2.554811000	12.565479000	5.029304000
H	-2.475716000	11.113538000	4.008561000
H	-3.015484000	10.993370000	5.686001000
C	3.550473000	15.860840000	8.473797000
H	4.252037000	16.134526000	9.269442000
H	4.053696000	16.050075000	7.519061000
H	2.684784000	16.530947000	8.526260000
C	4.456194000	13.574540000	8.419140000
H	4.296288000	12.499776000	8.502128000
H	4.934039000	13.776608000	7.454900000
H	5.158047000	13.867696000	9.206298000
C	-0.877751000	11.226559000	5.512628000
C	2.531757000	12.283080000	3.958130000
C	4.066590000	12.132198000	4.026651000
C	-0.223395000	12.664626000	3.527165000
H	-1.279739000	12.817689000	3.339301000
C	4.608418000	11.320992000	5.202398000
H	4.225613000	10.301791000	5.207125000
H	5.698666000	11.260096000	5.119188000
H	4.384465000	11.784003000	6.163272000
C	2.754296000	4.661876000	5.442181000
H	3.220794000	4.233826000	4.562348000
C	1.598062000	14.738921000	12.023521000
H	1.464728000	15.523416000	12.763853000
C	0.119729000	5.169937000	9.787041000
H	0.823527000	4.423755000	10.169963000
H	-0.374686000	5.626532000	10.651846000
H	-0.648114000	4.652441000	9.201888000
C	4.718755000	13.524702000	4.106497000
H	4.412566000	14.044782000	5.019301000
H	5.810241000	13.424785000	4.126293000
H	4.460717000	14.160843000	3.254410000
C	3.914197000	6.156290000	3.348265000
H	4.675181000	5.411170000	3.603189000
H	3.087745000	5.650145000	2.836109000
H	4.369236000	6.851170000	2.633717000
C	-0.703645000	9.698116000	5.508581000
H	-1.462525000	9.228785000	6.145302000
H	-0.824874000	9.302606000	4.494502000
H	0.280848000	9.387726000	5.860155000
C	0.065599000	10.948428000	13.022460000
H	-0.830166000	11.579190000	13.032975000
H	-0.254938000	9.917363000	13.208427000
H	0.706135000	11.245567000	13.859201000
C	0.697429000	13.256844000	2.668011000
H	0.360158000	13.862978000	1.831483000
C	0.803923000	11.009193000	11.677925000
C	2.170533000	3.802188000	6.366786000
H	2.185933000	2.727503000	6.205911000

C	4.547707000	11.432413000	2.741145000
H	4.276864000	11.988054000	1.838028000
H	5.639463000	11.328973000	2.754686000
H	4.116373000	10.429480000	2.660158000
C	2.049607000	13.051037000	2.889242000
H	2.762910000	13.502640000	2.205676000
C	-0.176880000	10.473436000	10.623572000
H	-1.063543000	11.113498000	10.558921000
H	0.269568000	10.441310000	9.629234000
H	-0.507737000	9.464375000	10.891910000
C	2.031340000	10.094539000	11.804840000
H	1.727035000	9.065980000	12.024615000
H	2.640359000	10.097416000	10.898078000
H	2.668350000	10.437064000	12.625332000

[^{Ar}L$_3$USU^{Ar}L$_3$] E = -4692.46060697

U	7.545629000	9.462903000	8.906604000
O	7.649900000	7.668541000	7.752173000
O	7.667182000	11.446462000	9.683158000
O	7.780005000	8.444763000	10.742764000
S	5.031269000	9.744768000	8.389537000
C	7.733680000	12.773250000	9.942767000
C	7.806621000	6.663110000	6.861258000
C	7.243386000	5.382413000	7.119702000
C	5.282739000	11.763414000	11.649485000
H	4.839922000	11.081601000	12.380551000
H	5.455721000	11.211319000	10.724590000
H	4.550281000	12.546860000	11.432854000
C	7.203238000	13.279976000	11.160659000
C	9.581034000	7.757996000	12.151961000
C	6.479245000	5.054588000	8.405987000
C	8.522778000	6.910703000	5.657969000
C	8.322172000	13.646186000	8.986298000
C	8.631620000	5.875969000	4.725048000
H	9.166591000	6.044719000	3.798618000
C	10.031176000	12.133137000	7.884983000
H	10.843151000	12.590852000	8.459416000
H	10.441128000	11.803025000	6.925173000
H	9.722818000	11.247630000	8.443419000
C	7.384964000	4.397632000	6.136926000
H	6.953087000	3.418456000	6.301735000
C	8.900070000	13.147834000	7.656237000
C	8.180157000	7.888712000	11.907673000
C	7.557331000	11.295502000	12.676325000
H	8.496115000	11.749620000	13.012161000
H	7.785496000	10.587858000	11.884149000
H	7.140680000	10.732799000	13.517298000
C	7.770998000	12.569039000	6.792273000
H	7.191313000	11.785805000	7.289208000
H	8.171282000	12.171387000	5.856011000
H	7.056734000	13.356108000	6.540742000
C	8.329963000	15.016385000	9.263491000
H	8.763471000	15.703997000	8.548199000
C	7.375423000	5.307296000	9.627071000
H	8.310521000	4.742621000	9.542680000
H	6.870390000	4.976006000	10.539470000
H	7.619652000	6.359501000	9.743158000
C	6.566048000	12.379068000	12.221550000
C	10.506465000	7.394180000	9.829246000
H	11.251618000	7.727220000	9.099588000
H	9.520002000	7.459573000	9.367858000
H	10.684393000	6.332157000	10.026452000
C	12.070823000	7.960707000	11.631173000

H	12.269666000	6.899290000	11.809702000
H	12.288542000	8.514670000	12.549890000
H	12.775892000	8.302907000	10.866049000
C	6.044054000	3.583150000	8.466267000
H	5.348235000	3.324155000	7.661371000
H	5.519471000	3.414064000	9.412703000
H	6.893191000	2.891507000	8.432690000
C	5.194183000	5.890136000	8.463863000
H	5.384174000	6.961909000	8.404698000
H	4.655412000	5.686918000	9.394827000
H	4.537500000	5.621283000	7.632816000
C	10.638750000	8.203106000	11.130428000
C	7.231998000	7.436779000	12.861738000
C	5.694045000	7.470670000	12.752441000
C	9.993282000	7.198942000	13.361429000
H	11.050613000	7.093366000	13.570202000
C	5.138463000	8.042964000	11.452846000
H	5.399566000	9.090605000	11.320365000
H	4.047149000	7.980010000	11.482834000
H	5.474840000	7.487118000	10.580291000
C	7.240425000	14.662140000	11.366044000
H	6.829113000	15.074432000	12.278894000
C	8.063226000	4.630199000	4.948424000
H	8.154080000	3.843263000	4.204930000
C	9.521613000	14.283044000	6.827802000
H	8.782916000	15.042003000	6.552902000
H	9.921688000	13.865472000	5.897636000
H	10.347711000	14.774869000	7.351789000
C	5.145845000	6.036326000	12.879864000
H	5.496817000	5.409249000	12.055194000
H	4.050264000	6.054167000	12.845934000
H	5.439610000	5.554189000	13.816316000
C	6.155304000	13.160595000	13.477854000
H	5.390584000	13.914511000	13.264337000
H	7.006931000	13.651485000	13.961800000
H	5.724995000	12.459225000	14.200545000
C	10.537132000	9.713475000	10.872276000
H	11.234599000	10.016665000	10.083822000
H	10.790656000	10.274309000	11.777464000
H	9.533917000	10.048703000	10.596966000
C	9.887644000	8.289353000	4.002244000
H	10.725351000	7.584606000	3.994056000
H	10.295942000	9.291388000	3.833651000
H	9.230575000	8.058059000	3.158365000
C	9.080068000	6.768628000	14.316787000
H	9.424885000	6.340399000	15.253770000
C	9.134581000	8.278569000	5.340988000
C	7.789681000	15.530665000	10.433242000
H	7.803293000	16.600699000	10.620490000
C	5.125606000	8.317912000	13.905961000
H	5.397241000	7.921283000	14.888681000
H	4.031338000	8.339844000	13.848068000
H	5.487504000	9.348395000	13.848031000
C	7.727137000	6.889796000	14.054876000
H	7.020260000	6.546826000	14.803811000
C	10.171404000	8.678310000	6.402477000
H	10.961025000	7.923573000	6.467764000
H	9.758213000	8.772837000	7.409394000
H	10.635994000	9.634066000	6.140537000
C	8.014804000	9.319251000	5.208379000
H	8.438521000	10.316142000	5.059892000
H	7.334632000	9.347035000	6.065155000
H	7.393209000	9.088508000	4.339372000

U	2.505196000	9.960376000	7.880981000
O	2.287751000	11.698767000	9.098305000
O	2.357080000	7.983100000	7.091466000
O	2.157030000	10.892220000	6.020553000
C	2.236771000	6.670960000	6.789729000
C	2.155612000	12.676145000	10.025853000
C	2.651949000	13.980826000	9.756466000
C	4.809322000	7.638448000	5.216160000
H	5.297845000	8.319013000	4.512631000
H	4.609249000	8.183315000	6.139777000
H	5.514824000	6.837364000	5.452050000
C	2.827277000	6.160211000	5.600524000
C	0.344681000	11.731659000	4.714562000
C	3.316636000	14.354826000	8.429206000
C	1.529627000	12.378214000	11.266779000
C	1.537790000	5.812258000	7.681643000
C	1.440019000	13.393964000	12.222531000
H	0.970288000	13.190751000	13.176842000
C	-0.215028000	7.395415000	8.594157000
H	-0.910405000	7.020098000	7.837280000
H	-0.794449000	7.663826000	9.483246000
H	0.215220000	8.315937000	8.196418000
C	2.537195000	14.943976000	10.763643000
H	2.919911000	15.941902000	10.590012000
C	0.831418000	6.324927000	8.944151000
C	1.747018000	11.592758000	4.939770000
C	2.596062000	8.157603000	4.088056000
H	1.681247000	7.722582000	3.670847000
H	2.317354000	8.846194000	4.881060000
H	3.078987000	8.737254000	3.294993000
C	1.862086000	6.855011000	9.948229000
H	2.501835000	7.641887000	9.542013000
H	1.358043000	7.242902000	10.838338000
H	2.532063000	6.051335000	10.265297000
C	1.514887000	4.444236000	7.394188000
H	1.003779000	3.764846000	8.064613000
C	2.356326000	14.083024000	7.261144000
H	1.419804000	14.634705000	7.398973000
H	2.801223000	14.414968000	6.318301000
H	2.117172000	13.028116000	7.162614000
C	3.542214000	7.052559000	4.581348000
C	-0.617878000	11.646224000	7.048356000
H	-1.316358000	11.126895000	7.713210000
H	0.381708000	11.585816000	7.482959000
H	-0.877086000	12.709675000	7.052579000
C	-2.139189000	11.346712000	5.147995000
H	-2.380839000	12.414459000	5.143478000
H	-2.323301000	10.944186000	4.146696000
H	-2.838149000	10.855807000	5.833339000
C	3.685071000	15.844065000	8.362467000
H	4.420301000	16.122709000	9.124459000
H	4.136808000	16.049657000	7.386126000
H	2.810252000	16.495620000	8.464251000
C	4.629746000	13.574310000	8.279198000
H	4.494808000	12.493733000	8.341917000
H	5.091031000	13.807278000	7.315467000
H	5.332377000	13.862217000	9.066774000
C	-0.703800000	11.075324000	5.625607000
C	2.686938000	12.174195000	4.049698000
C	4.225140000	12.074078000	4.107269000
C	-0.077676000	12.484817000	3.619080000
H	-1.136781000	12.609447000	3.431167000
C	4.787842000	11.288268000	5.286163000

H	4.430996000	10.260994000	5.296582000
H	5.877945000	11.255889000	5.201450000
H	4.551624000	11.752952000	6.241981000
C	2.765984000	4.781213000	5.380788000
H	3.226049000	4.364062000	4.493816000
C	1.944118000	14.664882000	11.987026000
H	1.869332000	15.435365000	12.749340000
C	0.060433000	5.209321000	9.667471000
H	0.720590000	4.411730000	10.021643000
H	-0.437978000	5.633163000	10.545602000
H	-0.711000000	4.765415000	9.029952000
C	4.831997000	13.487605000	4.171417000
H	4.507554000	14.011101000	5.074896000
H	5.925577000	13.419631000	4.195111000
H	4.558557000	14.102909000	3.309357000
C	3.996917000	6.269937000	3.341062000
H	4.734641000	5.498883000	3.586116000
H	3.159731000	5.799624000	2.813316000
H	4.475884000	6.966641000	2.644868000
C	-0.537837000	9.546583000	5.622734000
H	-1.309712000	9.084151000	6.246993000
H	-0.649496000	9.156266000	4.606256000
H	0.435392000	9.214013000	5.985003000
C	0.325157000	10.921731000	12.979145000
H	-0.546853000	11.581508000	13.038233000
H	-0.021519000	9.898739000	13.160178000
H	1.012687000	11.180661000	13.790108000
C	0.827107000	13.084971000	2.751155000
H	0.474122000	13.670563000	1.906953000
C	1.004371000	10.979078000	11.602739000
C	2.132993000	3.921323000	6.267569000
H	2.107099000	2.852853000	6.072921000
C	4.725952000	11.383966000	2.824094000
H	4.434055000	11.923903000	1.918567000
H	5.820564000	11.324517000	2.837412000
H	4.334433000	10.365203000	2.748368000
C	2.182545000	12.916980000	2.972186000
H	2.883054000	13.376800000	2.282245000
C	-0.058118000	10.523022000	10.591551000
H	-0.882054000	11.241926000	10.545843000
H	0.326787000	10.424960000	9.575318000
H	-0.468343000	9.552695000	10.888160000
C	2.187519000	10.005108000	11.661945000
H	1.836799000	8.983252000	11.826649000
H	2.804292000	10.024397000	10.758313000
H	2.846640000	10.269427000	12.491736000

S8. References

1 G. A. Bain and J. F. Berry, *J. Chem. Educ.*, 2008, **85**, 532.

2 D. E. Bergbreiter, *J. Am. Chem. Soc.*, 1978, **100**, 2126–2134.

3 A. G. Tskhovrebov, B. Vuichoud, E. Solari, R. Scopelliti and K. Severin, *J. Am. Chem. Soc.*, 2013, **135**, 9486–9492.

4 M. J. Monreal, R. K. Thomson, T. Cantat, N. E. Travia, B. L. Scott and J. L. Kiplinger, *Organometallics*, 2011, **30**, 2031–2038.

5 S. M. Mansell, N. Kaltsoyannis and P. L. Arnold, *J. Am. Chem. Soc.*, 2011, **133**, 9036–9051.

6 L. R. Avens, D. M. Barnhart, C. J. Burns, S. D. McKee and W. H. Smith, *Inorg. Chem.*, 1994, **33**, 4245–4254.

7 CrysAlisPro (Rigaku, V1.171.42.72a, 2022).

8 G. M. Sheldrick, *Acta Crystallogr A Found Adv*, 2015, **71**, 3–8.

9 O. V. Dolomanov, L. J. Bourhis, R. J. Gildea, J. A. K. Howard and H. Puschmann, *J Appl Crystallogr*, 2009, **42**, 339–341.

10 G. M. Sheldrick, *Acta Crystallogr C Struct Chem*, 2015, **71**, 3–8.

11 (a) A. D. Becke, *J. Chem. Phys.* 1993, **98**, 5648–5652; (b) K. Burke, J. P. Perdew, W. Yang, in Electronic Density Functional Theory: Recent Progress and New Directions, Eds: J. F. Dobson, G. Vignale, M. P. Das, Plenum, New York, 1998

12 (a) A. Moritz, X. Cao and M. Dolg, *Theor. Chem. Acc.,* 2007, **118**, 845–854; (b) A. Hollwarth, M. Bohme, S. Dapprich, A.W. Ehlers, A. Gobbi, V. Jonas, K.F. Kohler, R. Stegmann, A. Veldkamp, G. Frenking *J. Chem. Phys.,* 1993, **208**, 237–240.

13 (a) P. C. Hariharan and J. A. Pople, *Theor. Chim. Acta,* 1973, **28**, 213–222; (b) W. J. Hehre, R. Ditchfield and J. A. Pople, *J. Chem. Phys.,* 1972, **56**, 2257–2261.

14 S. Grimme, S. Ehrlich, L. Goerigk, *J. Comp. Chem.*, 2011, **32**, 1456–1465.

15 A. V. Marenich, C. J. Cramer, and D. G. Truhlar *J. Phys. Chem. B*, 2009, **113**, 6378–6396.

16 Gaussian 09, Revision D.01: M. J. Frisch, G. W. Trucks, H. B. Schlegel, G. E. Scuseria, M. A. Robb, J. R. Cheesman, G. Scalmani, V. Barone, B. Mennucci, G. A. Petersson, H. Nakatsuji, M. Caricato, X. Li, H. P. Hratchian, A. F. Izmaylov, J. Bloino, G. Zheng, J. L. Sonnenberg, M. Hada, M. Ehara, K. Toyota, R. Fukuda, J. Hasegawa, M. Ishida, T. Nakajima, Y. Honda, O. Kitao, H. Nakai, T. Vreven, J. A., Jr. Montgomery, J. E. Peralta, F. Ogliaro, M. Bearpark, J. J. Heyd, E. Brothers, K. N. Kudin, V. N. Staroverov, R. Kobayashi, J. Normand, K. Raghavachari, J. C. Burant, S. S. Iyengar, J. Tomasi, M. Cossi, N. Rega, M. J. Millam, M. Klene, J. E. Knox, J. B. Cross, V. Bakken, C. Adamo, J. Jaramillo, R. Gomperts, R. E. Stratmann, O. Yazyev, A. J. Austin, R. Cammi, C. Pomelli, J. W. Ochterski, R. L. Martin, K. Morokuma, V. G. Zakrzewski, G. A. Voth, P. Salvador, J. J. Dannenberg, S. Dapprich, A. D. Daniels, O. Farkas, J. B. Foresman, J. V. Ortiz, J. Cioslowski and D. J. Fox, Gaussian Inc., 2009, Wallingford CT.
